# Tuning the Efficiency of Iridium(III) Complexes for Energy Transfer (EnT) Catalysis through Ligand Design

**DOI:** 10.1002/chem.202403309

**Published:** 2025-02-25

**Authors:** Davide Ruggeri, Matteo Hoch, Davide Spataro, Luciano Marchiò, Stefano Protti, Daniele Cauzzi, Matteo Tegoni, Matteo Lanzi, Giovanni Maestri

**Affiliations:** ^1^ Department of Chemistry, Life Sciences and Environmental Sustainability Università di Parma Parco Area delle Scienze 17/A 43124 Parma Italy; ^2^ Photo Green Lab, Department of Chemistry Università di Pavia Via Taramelli 10 27100 Pavia Italy

**Keywords:** Catalysis, Energy transfer, Photocatalysis, Complexes

## Abstract

Photoactive Iridium(III) complexes are a popular synthetic tool. The impact of ligand design on their photoredox properties has been widely studied, but similar approaches to develop more potent photosensitizers are still absent. We report herein the preparation, characterization and catalytic application of a new family of Iridium(III) complexes that proved superior to their widely‐used commercial peers. The best results were observed when naphthyl pendants were installed to the ligands, which could stabilize the triplet intermediates involved in energy‐transfer reactions *via* radical‐π dispersion interactions.

## Graphical abstract

A family of heteroleptic Iridium(III) complexes, which can act as Energy Transfer (EnT) photocatalysts, has been prepared and tested in photochemical transformations. Our study explores ligand design and its impact on the photophysical properties of the complexes and their catalytic performance. Naphthyl pendants enhance triplet intermediate stabilization in energy‐transfer reactions via radical‐π dispersion interactions, leading to superior catalytic efficiency.

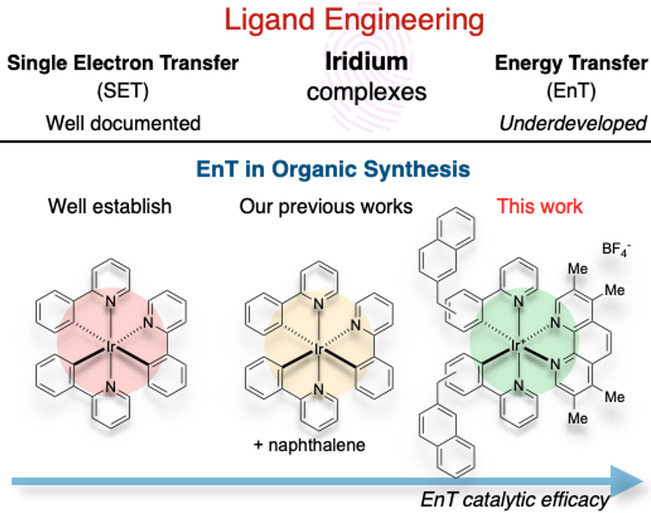

## Introduction

In recent decades, visible‐light driven processes have emerged as an essential tool in organic and material chemistry, enabling novel and unparalleled transformations.[^1^, ^2^, ^3^, ^4^] Activating small molecules with light is often limited by the need for intense UV light, which can reduce the reaction selectivity and functional groups tolerance.[^5^, ^6^, ^7^] A crucial role in harvesting the energy carried by low‐energetic visible photons is played by photocatalysts, triggering electron or energy transfer events that would otherwise be highly challenging.[^8^, ^9^, ^10^] Strategies involving single electron transfer (SET) are the most common, although, recent focus on energy transfer processes (EnT) led to the development of novel transformations, which are particularly useful to convert redox‐inert substrates.[^11^, ^12^, ^13^, ^14^, ^15^] Cyclization and isomerization reactions have particularly gained prominence by achieving significant level of molecular complexity through streamlined protocols.[^16^, ^17^, ^18^, ^19^, ^20^, ^21^, ^22^] Iridium complexes stand out among the expansive array of organic and metal‐based photosensitizers thanks to several distinguishing features.[^23^, ^24^]

**Figure 1:**
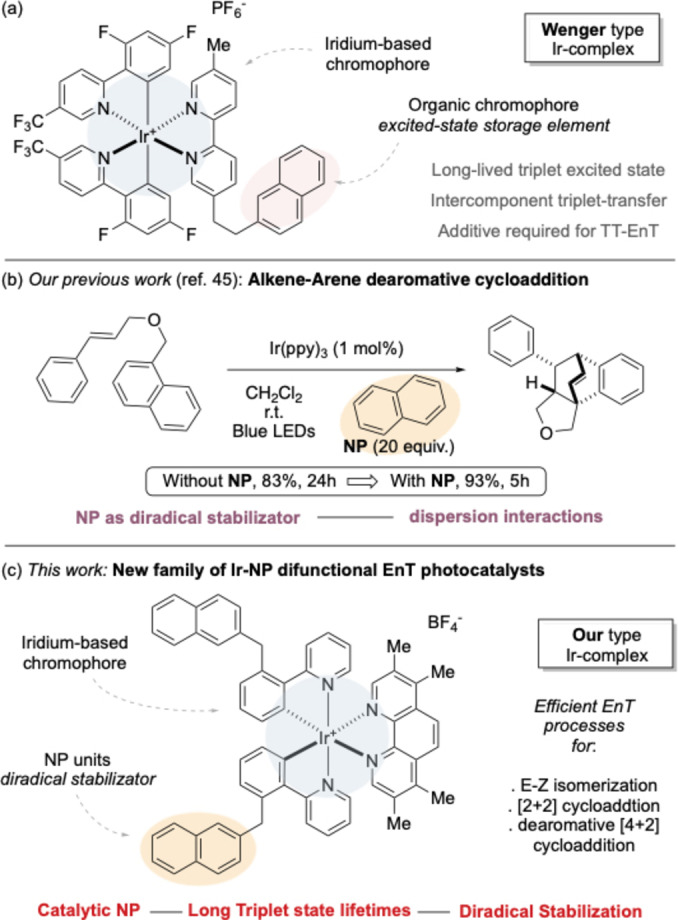
(a) Wenger type photocatalysts; (b) Our previous study on diradical stabilization; (c) This work.

Their long excited‐state lifetimes (Tt), chemical stability and the tunable nature of their optoelectronic properties rend them a particularly effective tool.[^25^, ^26^] Photoactive Ir(III) complexes usually have cyclometallated chelating ligands, which play a key role on determining the energetic level of the HOMO of the complex thanks to the strong σ‐donating capabilities of the carbon nucleus.[^27^, ^28^, ^29^, ^30^, ^31^] It is possible to tune this energetic level by adjusting the electronic effect of substituents on the ligands.[^32^, ^33^, ^34^, ^35^, ^36^] Conversely, the decoration of the N,N‐ligands, such as bipyridines (bpy), has a pronounced impact on the LUMO of the complex. Additional derivatization approaches include the insertion of the π‐conjugation to the ligands or the tethering with additional chromophores, that could act as complementary antennas.[^37^, ^38^, ^39^] Recent advancements in the field have been reported by Wenger, who designed a two‐component derivative consisting of a photoactive complex that functions as an antenna to harvest visible light and a 2‐naphthyl unit that serves as a triplet‐state storage element (Figure 1a).^[40]^ The two functional groups are tethered by an insulating spacer formed by two C(sp^3^) carbons. Once visible‐light excites the organometallic core, an intramolecular EnT can occur. The bifunctional compound could thus achieve a longer excited‐states lifetimes (up to 20 μs) by taking advantage of the long‐lifetime of sensitized naphthalenes. These compounds could trigger triplet‐triplet energy‐transfer annihilation (TT‐EnT) events, and the corresponding activation modes could lead to catalytic reactivities in the presence of 10 mol% of 2,5‐diphenyloxazole as annihilator.

Despite the extensive exploration of photocatalysts for SET transformations, there is a notable lack in understanding the correlation between the structural derivatization of Ir(III) complexes and their key properties for EnT processes where most of these methods rely on a few, commercially available photosensitizer. We recently demonstrated the beneficial role of naphthalene (**Naph**) and di‐**Naph**s derivatives on several EnT reactions, including *E‐Z* photoisomerization, [2+2] and [4+2] photocycloadditions and HAT/cyclization of allenamides (Figure 1b).[^41^, ^42^, ^43^] These additives are capable to stabilize the transient triplet intermediates of the freshly activated organic substrate through stacking‐like radical‐π dispersion interactions thus reducing the energetic cost of transition states.[^44^, ^45^] Adding **Naph** enhanced the rates and the yields of the reactions, while triggering otherwise silent transformations. However, the need for a large excess of **Naph** (5–20 equiv.) might limit the broad application of this methods.

Given these premises, we reasoned that it might have been possible to elicit similar stabilizing interactions at low **Naph** concentrations by designing Iridium complexes with **Naph** pendants for a direct activation of substrates. Because of the collisional nature of the EnT, the activation would occur at relatively close spatial proximity with respect to the π cloud of tethered naphthyl units, which might therefore more easily give rise to dispersion interactions with the resulting organic triplet intermediates. This hypothesis was coherent with the observation of an EnT between a photoexcited two‐chromophores adduct and 2,5‐diphenyl oxazole, which mediate a model TT‐EnT reactivities.^[40]^

We report here the preparation of Ir(III) photosensitizers in which the metal complex and pendant naphthyl units are spaced by saturated nuclei (Figure 1c). In contrast to the complex described by Wenger, the naphthyl arm of these species does not undergo excitation upon exposure to visible‐light. However, these complexes are competent for several EnT‐based transformations, since they can display excited‐states lifetimes and triplet energies (E_T_) that are superior to those of their popular commercial peers. Moreover, present results point out that the positive effect of **Naph** pendants can be still observed at low catalytic concentrations (1–2 mol%).

## Results and Discussion

**Scheme 1:**
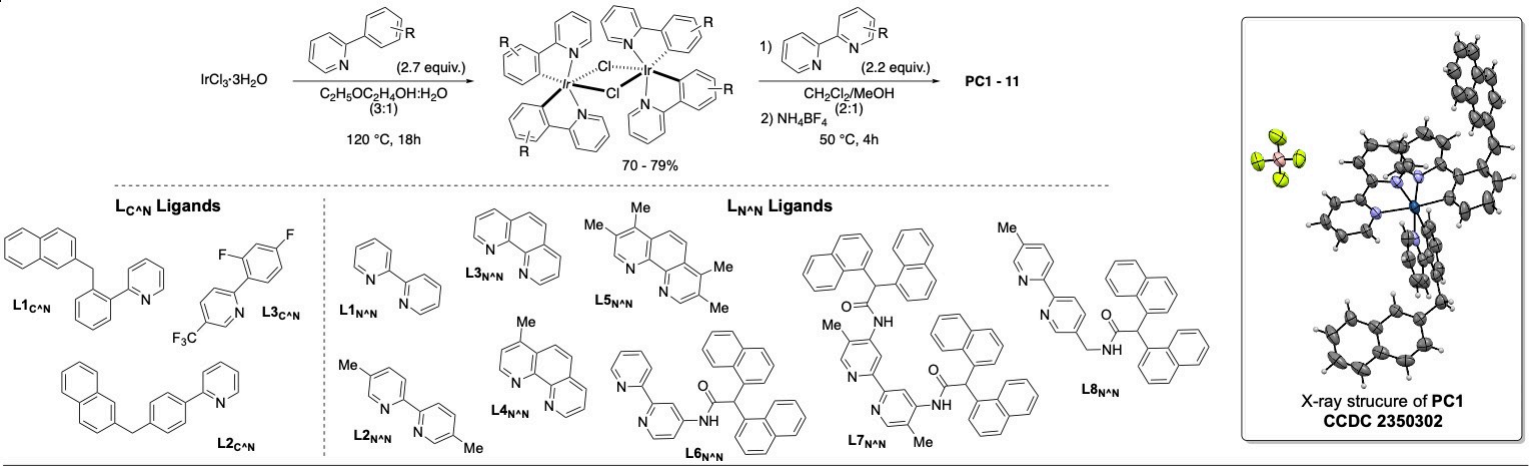
Convergent synthesis of heteroleptic iridium complexes **PC1**−**11** mixing phenylpyridines (**L_C N_**) with bipyridines and phenanthroline (**L_N N_**) ligands.

**Scheme 2:**
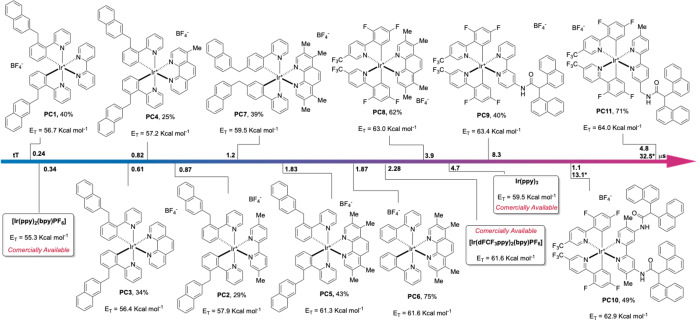
Iridium photosensitizers **PC1** – **PC11**, overall yield, E_T_ and Tt. The triplet‐state lifetimes (Tt) were measured by irradiating a CH_3_CN solution of **PC** at 375 nm (absorption 0.2; plotted on the central axis [μs]); Unless otherwise specified all the complexes present a mono‐exponentially phosphorescence emission decay; *PC10 and **PC11** present a bi‐exponential phosphorescence decay see supporting information.

We began our study with the preparation of C N ligands (ppy **L1_C N_** and **L2_C N_**) and N N ones (**L6_N N_**, **L7_N N_** and **L8_N N_**) that are variously decorated with flexible pendant **Naph** units (Scheme 1). Heteroleptic Ir‐complexes **PC1–11** have been assembled by utilizing IrCl_3_ 3H_2_O as metal source. The desired complexes were prepared in moderate to good overall yields over two steps. They were purified by column chromatography on silica gel and proved to be fully stable, either in solution or in the solid state. The effect of the ligand on the photophysical properties of the complex (Scheme 2) was compared by preparing photosensitizers that had either modified C N ligands or decorated N N ones. Complexes **PC1** to **PC5** were synthesized combining the use of ligand **L1_C N_** with various bipyridines or phenanthrolines as N N ligands (**L_N N_**).

The replacement of **L1_N N_** with **L2_N N_** led to a significant increment of the triplet lifetime (Tt) of the corresponding complex from 0.24 to 0.87 μs. The difference between the E_T_ of **PC1** and **PC2** is narrower (1.2 kcal/mol). An analogous scenario on both the E_T_ and the Tt has been observed replacing phenanthroline with a methyl‐substituted one (**L3_N N_** and **L4_N N_**, respectively). The presence of the tetra‐methyl substituted **L5_N N_** in **PC5** and **PC6** was accompanied by a further and significant elongation of the Tt (1.83 and 1.87 μs, respectively). In parallel, the use of this N N ligand had a larger effect on the E_T_ of these complexes, which was above 61 kcal/mol in both cases. Aiming to further extend both the Tt and the E_T_ of these iridium complexes, we tried to tune their HOMO‐LUMO gap with the introduction of electron withdrawing groups on the cyclometallated ligand. We thus combined **L3_C N_** with the commercially available **L5_N N_** and the newly prepared, **Naph**‐containing **L_N N_**, **L6_N N_**, **L7_N N_** and **L8_N N_**. The four corresponding complexes were prepared in moderate to very good overall yields (**PC8–11**). Interestingly, all of them displayed a higher E_T_ than that of [Ir(dF(CF_3_)ppy)_2_(dtbbpy)PF_6_], which is often the benchmark Ir(III) photosensitizer for EnT processes.[^10^, ^11^, ^12^, ^13^] Moreover, the introduction of a rigid **Naph**‐decorated pendants was accompanied by a peculiarly long Tt either for **PC9**, **PC10** and **PC11** (8.3, 14.2 and 37 μs respectively). In the specific case of **PC10** and **PC11**, a biexponential decay was observed (see Supporting Information).

With the new set of complexes in hand, we delved into the investigation of their catalytic properties using three different model EnT reactions. Our screening commenced with the benchmark *E‐Z* photoisomerization of model β‐styrene **A** (Table 1).[^46^, ^47^] Once the triplet state of the complex has been populated thanks to visible‐light photons, an EnT step can occur on substrates featuring a conjugated alkene unit, which is most often a styrene or 1,3‐diene fragment. This generates the corresponding triplet intermediate, that can relax giving either the starting *E‐* or the *Z‐* isomer.

**Table 1:** Evaluation of the photosensitizers in the isomerization reaction of **A**.

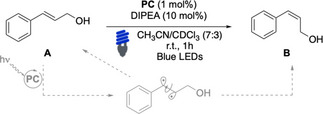			
Entry^[a]^	PC	**A**	**B**
1	Ir(ppy)_3_	64	36
2^[b]^	Ir(ppy)_3_	24	76
3	[Ir(dF(CF_3_)ppy)_2_(dtbbpy)PF_6_]	69	31
4^[c]^	[Ir(dF(CF_3_)ppy)_2_(bpy)PF_6_]	77	23
5	[Ir(ppy)_2_(bpy)PF_6_]	94	6
6	**PC1**	93	7
7	**PC2**	89	11
8	**PC3**	97	3
9	**PC4**	91	9
10	**PC5**	29	71
11	**PC6**	31	69
12	**PC7**	40	60
13	**PC8**	80	20
14	**PC9**	54	46
15	**PC10**	56	44
16	**PC11**	80	20

^[a]^Conditions:**A**(0.1 mmol), DIPEA (10 mol%),**PC**(1 mol%) in MeCN/CDCl_3_(7:3, 0.1 M), irradiated with blue LEDs (420–520 nm,λ_max_ emission 460 nm, copies of measured emission spectra in the SI) for 1 hour at 25 °C in 5 mm NMR tube under N_2_,**A**:**B** ratio was determined by ^**1**^ **H NMR** using 1,3,5‐trimethoxybenzene as internal standard;^[b]^ 5 equiv. Naphthalene were added;^[c]^ results from ref [41].

The less favorable sensitization of the latter compared to the former, allows to accumulate the *Z‐* isomer in solution until its concentration reaches a point where the rates of the two competing EnT pathways become identical, typically requiring prolonged irradiation times to achieve equilibrium. In the absence of **Naph**, the widely used Ir(ppy)_3_, afforded a partial conversion of the starting material **A** into the corresponding *Z‐*isomer, **B**, upon 1 hour of irradiation. The addition of 5 equiv. of **Naph** increased the rate of the reaction (Table 1, entries 1–2). Without **Naph**, other commercially available iridium complexes performed poorly under these conditions (Table 1, entries 3–5). We then tested the activity of **PC1** – **PC11**. The introduction of the **Naph** unit onto **PC1** – **PC4** complexes did not lead to better results (Table 1, entries 6–9). A higher conversion of **A** was observed employing complexes **PC5** – **PC7**, which feature the **L5_N N_** ligand (Table 1, entries 10–12). These results highlight the positive effect of the substituted phenanthroline ligand on the model reaction. In particular, the faster photoisomerization was achieved in the presence of **PC5**, which features two **Naph** pendants. The relative position of these groups plays a role on the isomerization of **A**, as shown by the result employing **PC7**, which proved to be less competent (Table 1, entry 12). The modification of the iridium complexes with the introduction of the fluorinated phenylpyridine **L3_C N_** (**PC8** – **11**) did not improve the isomerization (Table 1, entries 13–16) but showed that **Naph**‐containing complexes **PC9** – **11** gave higher conversion of **A** compared to their commercial peers. Despite a longer triplet‐state lifetime of **PC9**, **PC10** and **PC11**, these complexes performed poorly, probably due to the rigidity of the linkers. **PC5** and **PC7**, bearing **Naph**‐moieties at a correct structural distance from the metal center, showed high efficiency in the activation of the substrate, furthermore highlighting the positive and unparallel effect of naphthyl‐ moieties on the radical‐π dispersion stabilization.

We next examined the efficacy of these complexes in the [2+2] photocycloaddition of **C** to afford the fused bicyclic **D** with high diastereocontrol (Table 2). These EnT‐mediated reactions are widely employed for the preparation of highly functionalized cyclobutanes and they involve the initial sensitization of a conjugated alkene, through a photocatalytic pathway like the previously mentioned isomerization.[^48^, ^49^, ^50^] Once the triplet of the substrate has been generated, a radical 5‐*exo*‐trig cyclization is the most common reactivity observed for intramolecular substrates, and this step seals the first cycle of the final product. The resulting triplet could then relax, paving the way for an intramolecular radical recombination that eventually affords the product **D**. For our purposes, we prepared substrate **C**, which is a challenging diene for EnT cycloadditions. This is due to its flexible ethereal tether, which disfavors the first cyclization owing to a negative Thorpe‐Ingold effect, and to the presence of a terminal alkene unit that reduces the stability of the cyclic intermediate because of its primary radical character. As a result, the full conversion of similar substrates usually required long irradiation times.[^48^, ^49^, ^50^] Indeed, only moderate yields toward the desired product **D** were achieved in the presence of commercially available Iridium(III) photosensitizers upon four hours of irradiation (Table 2, entries 1, 3–5). The *Z‐* isomer of the substrate (**C’**) was the main product. However, the introduction of 20 equivalent of naphthalene delivered **D** in quantitative yield (Table 2, entry 2). Photosensitizers **PC1** – **PC4** were ineffective for this transformation (Table 2, entries 6–9). On the contrary, use of **PC5** led to the best isolated yield of **D** (86%). ^**1**^ **H NMR** analysis of the crude reaction mixture showed that the complete conversion of **C** occurred and only traces of **C′** were present (Table 2, entry 10). The significant role played by **Naph** units was underscored by comparing the outcome using **PC6** (Table 2, entry 11). The latter afforded the desired product in a 14 % lower yield. Additional attempts to modify the structure of ligands, do not led to higher substrate conversion or better product yields compared to commercial Iridium(III) complexes.

**Table 2:** Evaluation of the photosensitizers in the [2+2]‐reaction of **C**.

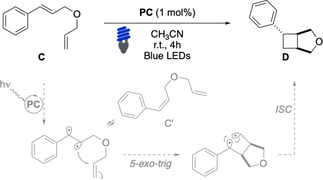				
Entry^[a]^	PC	**C**	**C’**	**D**
1	Ir(ppy)_3_	11	50	21
2^[b]^	Ir(ppy)_3_	0	0	99
3^[c]^	[Ir(dF(CF_3_)ppy)_2_(dtbbpy)PF_6_]	2	4	65
4	[Ir(dF(CF_3_)ppy)_2_(bpy)PF_6_]	16	35	49
5^[d]^	[Ir(ppy)_2_(bpy)PF_6_]	91	0	0
6	**PC1**	92	traces	0
7	**PC2**	38	38	7
8	**PC3**	70	11	traces
9	**PC4**	28	42	5
10^[c]^	**PC5**	0	traces	86
11	**PC6**	0	0	72
12	**PC7**	2	4	72
13	**PC8**	10	21	49
14	**PC9**	7	25	52
15^[c]^	**PC10**	0	0	63
16	**PC11**	12	43	27

^[a]^ Conditions: **C** (0.1 mmol), **PC** (1 mol%) in MeCN (0.05 M), irradiated with blue LEDs (420–520 nm, λ_max_ emission 460 nm, copies of measured emission spectra in the SI) for 4 hours at 25 °C in 5 mm NMR tube under N_2_, **C**:**C’** ratio and d.r. were determined by ^1^ **H NMR** using 1,3,5‐trimethoxybenzene as internal standard, d.r. 86:14;^[b]^ 20 equiv. of naphthalene were added;^[c]^ Isolated yield; ^[d]^ results from ref [41].

We then tested our recently developed dearomative alkene‐arene [4+2] photocycloaddition as recognized challenging transformation and thus prove the general applicability of present complexes (Table 3).[^17^, ^51^, ^52^, ^53^, ^54^, ^55^, ^56^] The activation of substrate **E** follows the aforementioned mechanism, involving a triplet intermediate and a reactive radical site attack with the generation of a quaternary carbon at the 1‐position of the naphthyl ring via 5‐*exo*‐trig cyclization. This step is the most challenging of the cascade because it causes the loss of the aromatic stabilization of a phenyl ring. The final product is formed upon inter‐system‐crossing (ISC) through intramolecular radical recombination, eventually delivering with high diastereocontrol a complex fused/bridged tetracyclic scaffold that has four contiguous stereocenters.[^5^, ^11^, ^28^, ^50^, ^57^, ^58^, ^59^] Likely owing to the inherent challenge of the dearomative transformation, a multitude of catalysts failed to provide decent reaction outcomes (Table 3, entries 1 and 3–9) in absence of **Naph** (20 equiv., Table 3, entry 2). On the contrary, the desired product **F** has been isolated in good to quantitative yields using **PC5** ‐ **PC11**. Complexes featuring electron‐rich C N ligands in combination with phenanthroline **L5_N N_**, as **PC5** and **PC7** equipped with **Naph** pendants led to high reaction yields and full conversion (Table 3, entries 10–12). Note to mention, the results obtained using **PC5** and **PC7** resemble the activity observed with Ir(ppy)_3_ only when 20 equiv. of **Naph** was used, thus further denoting the activity of the here presented **Naph**‐based designed complexes. The same trend was observed when comparing **PC8** ‐ **PC11**, which feature the presence of the fluorinated **L3_C N_** ligand (Table 3, entries 13–16).

**Table 3:** Evaluation of the photosensitizers in the dearomative [4+2]‐cycloaddition reaction of **E**

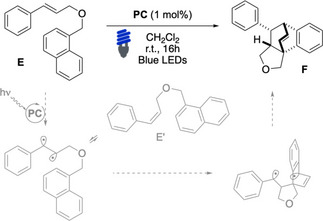				
Entry^[a]^	PC	E	E’	F
1	Ir(ppy)_3_	21	18	46
2^[b,c]^	Ir(ppy)_3_	0	0	93
3	[Ir(dF(CF_3_)ppy)_2_(dtbbpy)PF_6_]	14	9	64
4	[Ir(dF(CF_3_)ppy)_2_(bpy)PF_6_]	21	14	60
5	[Ir(ppy)_2_(bpy)PF_6_]	78	7	0
6	**PC1**	63	18	traces
7	**PC2**	31	26	26
8	**PC3**	52	31	traces
9^[c]^	**PC4**	30	32	13
10^[c]^	**PC5**	0	traces	89
11^[c]^	**PC6**	0	0	81
12^[c]^	**PC7**	0	0	85
13	**PC8**	29	23	45
14	**PC9**	0	0	68
15	**PC10**	0	0	68
16	**PC11**	26	19	55

^[a]^ Conditions: **E** (0.1 mmol), **PC** (1 mol%) in DCM (0.05 M), irradiated with blue LEDs (420–520 nm, λ_max_ emission 460 nm, copies of measured emission spectra in the SI) for 16 hours at 25 °C in 5 mm **NMR** tube under N_2_, **E**:**E’** ratio and d.r. were determined by ^**1**^ **H NMR** using 1,3,5‐trimethoxybenzene as internal standard, d.r. 82:18; ^[b]^ 20 equiv. of naphthalene were added; ^[c]^ isolated yield.

The results obtained from the three model catalytic reactions studied showed that several of the newly prepared Iridium complexes are competent photosensitizers for EnT processes. Poor results were observed using species that have relatively low E_T_ and short Tt (**PC1** ‐ **4**). Among complexes that have a sufficiently high E_T_ and long Tt (**PC5** – **11**), the best results were invariably achieved employing those that present pendant **Naph** fragments, such as **PC5**, rather than using those without them (**PC6**, **PC8**). Although the differences in the rate of substrate conversion and on the yield of the product might appear narrow (up to 14 %, Table 2), it is worth noting that these results were achieved by slashing the concentration of the additive by up to three orders of magnitude compared to our previous studies, from 20 equiv. (2000 mol%) to 2 mol% of catalytic additive.^[60]^

To further evaluate the potential of our new set of photosensitizers, we challenged the **PC**s that had demonstrated the highest efficiency in prior transformations in the activation of allene **G** (Table 4). Initially, we tested Ir(ppy)₃ both with and without naphthalene (20 equiv.) as an additive (Table 4, entries 1–2). The desired product was obtained in good yields only when naphthalene was included. Subsequently, we examined the newly developed catalysts, **PC5**, **PC10**, and **PC11** (Table 4, entries 3–5). All of these catalysts successfully delivered the desired product in moderate to good yields. These results highlighted the versatility and efficiency of the new photosensitizers in enabling EnT activation, offering a viable alternative to conventional catalysts under optimized conditions.

**Table 4:** Evaluation of the photosensitizers in catalytic [1,5]‐HAT/cyclization on allenamide **G**

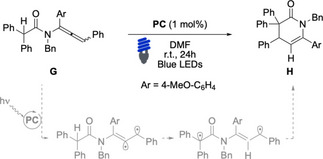		
Entry^[a]^	PC	H^[b]^
1	Ir(ppy)_3_	26
2^[c]^	Ir(ppy)_3_	86
3	**PC5**	43
4^[d]^	**PC10**	54
5	**PC11**	59

^[a]^ Conditions: **G** (0.1 mmol), **PC** (1 mol%) in DMF (0.1 M), irradiated with blue LEDs (420–520 nm, λ_max_ emission 460 nm, copies of measured emission spectra in the SI) for 24 hours at 25 °C in 5 mm **NMR** tube under N_2_, ^[b]^ determined by ^**1**^ **H NMR** using 1,3,5‐trimethoxybenzene as internal standard; ^[c]^ 20 equiv. of naphthalene were added; ^[d]^ isolated yield.

**Scheme 3:**
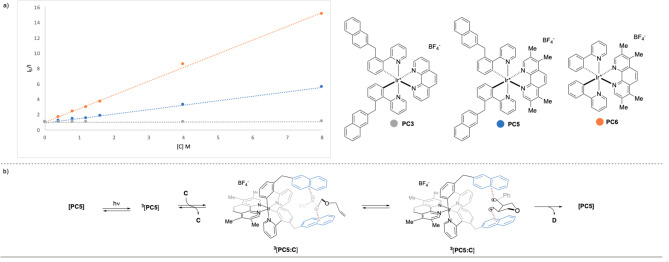
(a) Stern‐Volmer phosphorescence quenching studies of complex **PC3**, **PC5** and **PC6** in presence of **C**; (b) Proposed mechanism for enhanced catalytic activity of **PC**.

In order to confirm the role of the pendant **Naph** units for the transformation of **C** in **D** *via* [2+2] cycloaddition, we performed quenching experiments of three model Iridium complexes with substrate **C**, deriving the corresponding Stern‐Volmer plots (Scheme 3a). The organic molecule was not a competent quencher for **PC3** (gray line). This complex gave poor results in catalytic experiments, likely as a combination of its reduced EnT efficiency and relatively short Tt.

Conversely, **C** is a good quencher for the photoexcited **PC5** (K_SV_=564 M^−1^), which was indeed the best photosensitizer for the corresponding [2+2] photocycloaddition (Table 1, entry 10). Interestingly, a significantly higher quenching ability was measured employing **PC6** (K_SV_=1776 M^−1^), which shares the same structure of **PC5** with the exception of the two pendant **Naph** fragments. This difference can be due to the relatively higher E_T_ and Tt of the former compared to the latter (Δ=0.3 kcal mol^−1^ and 42 ns, respectively). The data derived from photophysical analyses suggest that **PC6** might be superior to **PC5** (Scheme 3), however, opposite catalytic performance was observed for the presented reactions (Tables 1, 2, 3, 4).^[61]^ These results could confirm that the beneficial role of the pendant **Naph** unit is due to the stabilization *via* dispersion interactions between the **Naph**‐moieties and the triplet state intermediates of EnT reaction, which could not take place using non‐decorated complexes such as **PC6** (Scheme 3b).

## Conclusions

In conclusion, we reported the synthesis of a family of heteroleptic Iridium(III) complex, which could act as Energy Transfer (EnT) photocatalysts upon irradiation with visible‐light photons. Our study delves into the ligand design, examining its impact on various photophysical properties of the complexes. This encompasses their light absorption, energy transfer dynamics, and triplet state longevity. Several of the complexes described herein gave better results than popular Iridium‐based complexes, making them promising candidates for EnT processes. The comparison of photocatalysts bearing pendant **Naph** units with their corresponding non‐decorated peers showed that the use of the former provided better catalytic results, thus indicating the generation of stabilizing radical‐ π dispersion interactions between **Naph** fragments and the triplet intermediates involved in EnT reactions. Noteworthily, the beneficial effect that was previously observed in related processes using a large molar excess of **Naph** (5–20 equiv.) can be still noticed at low concentrations of the aryl pendant (2 mol%) using present photosensitizers.

## Supporting Information

Detailed experimental procedures, analytical data, NMR spectra are provided in the Supporting Information. The authors have cited additional references within the Supporting Information. Deposition Number 2350302 (for **PC1**) contains the supplementary crystallographic data for this paper. These data are provided free of charge by the joint Cambridge Crystallographic Data Centre and Fachinformationszentrum Karlsruhe.

## Conflict of Interests

The authors declare no conflict of interest.

## Supporting information

As a service to our authors and readers, this journal provides supporting information supplied by the authors. Such materials are peer reviewed and may be re‐organized for online delivery, but are not copy‐edited or typeset. Technical support issues arising from supporting information (other than missing files) should be addressed to the authors.

Supporting Information
