# Supplementary material for: Tuning the Efficiency of Iridium(III) Complexes for Energy Transfer (EnT) Catalysis through Ligand Design
Source: Chemistry. 2025 Feb 25;31(18):e202403309. doi: 10.1002/chem.202403309 (PMC11937879; doi:10.1002/chem.202403309)
Supplement: Supplementary file 1 — Supporting Information [file CHEM-31-e202403309-s001.pdf]

Chemistry–A European Journal

Supporting Information

Tuning the Efficiency of Iridium(III) Complexes for Energy Transfer (EnT) Catalysis through Ligand Design

Davide Ruggeri, Matteo Hoch, Davide Spataro, Luciano Marchiò, Stefano Protti, Daniele Cauzzi, Matteo Tegoni, Matteo Lanzi,* and Giovanni Maestri*

Electronic Supplementary Information

for

Tuning the Efficiency of Iridium(III) Complexes for Energy Transfer (EnT) Catalysis through Ligand Design

Davide Ruggeri [a], Matteo Hoch [a], Davide Spataro [a], Luciano Marchiò [a], Stefano Protti [b], Daniele Cauzzi [a], Matteo Tegoni [a], Matteo Lanzi [a] and Giovanni Maestri [a]

a. Department of Chemistry, Life Sciences and Environmental Sustainability Università di Parma, Parco Area delle Scienze 17/A, 43124 Parma, Italy
b. Photo Green Lab, Department of Chemistry Università di Pavia, Via Taramelli 10, 27100, Pavia, Italy

matteo.lanzi@unipr.it
giovanni.maestri@unipr.it

Table of Contents

General Remarks 3

Setup adopted for the catalytic reactions 4

Procedures for synthesis of the ligands 7

- **Procedure for the preparation of ligand L1$_{C^N}$** 7
- **Procedure for the preparation of ligand L2$_{C^N}$** 8
- **Procedure for the preparation of ligand L3$_{C^N}$** 9
- **Procedure for the preparation of ligand L6$_{N^N}$** 10
- **Procedure for the preparation of ligand L7$_{N^N}$** 13
- **Procedure for the preparation of ligand L8$_{N^N}$** 15
- **Procedures for the preparation of Iridium Dimers 2a-2d** 17

General procedure for the preparation of Heteroleptic Iridium Complexes PC 1-7 (GP-1) 19

General procedure for the preparation of Heteroleptic Iridium Complexes PC 8 (GP-2) 24

General procedure for the preparation of Heteroleptic Iridium Complexes PC 9-10 (GP-3) 25

Procedures for the preparation of substrates 29

- **Preparation of G** 29

Screening of the catalytic activities of the Ir-complexes 30

- **Catalytic isomerization reaction** 31
- **Catalytic [2+2] reaction** 33
- **Catalytic [4+2] reaction** 35
- **Catalytic [1,5]-HAT/cyclization on allenamide G** 37

Photophysical Characterizations of the complexes 39

Absorption Spectra 40

Emission Spectra 48

Photoluminescence Lifetime Measurements 55

Quantum yield measurements in ACN 63

Triplet energy measurements in ACN 64

Quenching Experiments 65

Cyclic Voltammetry Experiments 69

Copies of NMR spectra 74

- **Characterization of the Ligands** 75
- **Charatherization of complexes** 93
- **Photoisomerization of cinnamyl alcohol 3a, copies of NMR spectra** 119

References 135

General Remarks

All chemicals those syntheses are not reported hereafter were purchased from commercial sources and used as received. Solvents were dried passing through alumina columns using an Inert® system and were stored under nitrogen. Chromatographic purifications were performed under gradient using a Combiflash® system and prepacked disposable silica cartridges or through isocratic flash chromatography using commercial 60 Å silica gel. When necessary, compounds were additionally purified by Preparative Thin-Layer Chromatography. All reactions that required heating were performed with the use of high-vacuum grade silicon oil. Reactions promoted by visible light were performed into standard 5 mm NMR tubes, surrounded by a commercial strip of 300 RGB household LEDs (12V, 14W). These were put at ca. 10 cm and irradiated blue light (10.7 $W{\cdot}m^{-2}$ in the whole visible range; 0.35 $W{\cdot}m^{-2}{\cdot}nm^{-1}$ at their λ_{max}: 459nm). The tubes were inside an oil bath fitted with a thermometer to monitor the temperature, oil bath was kept at 25 °C through a spire linked to an external chiller pumping a cooled water/ethylene glycol solution to maintain the desired temperature. ^{1}H and ^{13}C NMR spectra were recorded at 300 K on a Bruker 400 MHz spectrometer using residual non-deuterated solvents as internal standards (7.26 ppm for ^{1}H NMR and 77.00 ppm for ^{13}C-NMR for $CDCl_3$, 2.05 ppm for ^{1}H NMR and 29.84 ppm for ^{13}C NMR for acetone-d_6). The terms m, s, d, t, q and quint represent multiplet, singlet, doublet, triplet, quadruplet and quintuplet respectively, and the term brs is referred to broad signal. Mass analyses were recorded on an Infusion Water Acquity Ultra Performance LC HO6UPS-823M instrument equipped with a SQ detector (Electrospray source); high-resolution mass analyses were recorded on a LTQ ORBITRAP XL Thermo Mass Spectrometer (Electrospray source).

Single crystal Data were collected with a Bruker D8 diffractometer equipped with PhotonII area detector, using a CuKα or a MoKα microfocus 4 radiation source. The data collection strategy covered the sphere of reciprocal space. Absorption corrections were applied using the program SADABS. The structure was solved with the SHELXT code. Fourier analysis and refinement were performed by the fullmatrix least-squares methods based on F2 using SHELXL-2014 as implemented in Olex2. All the nonH atoms were refined with anisotropic displacement parameters. CCDC no 2350302 contains the X-Ray data for complex **PC1**.

Setup adopted for the catalytic reactions.

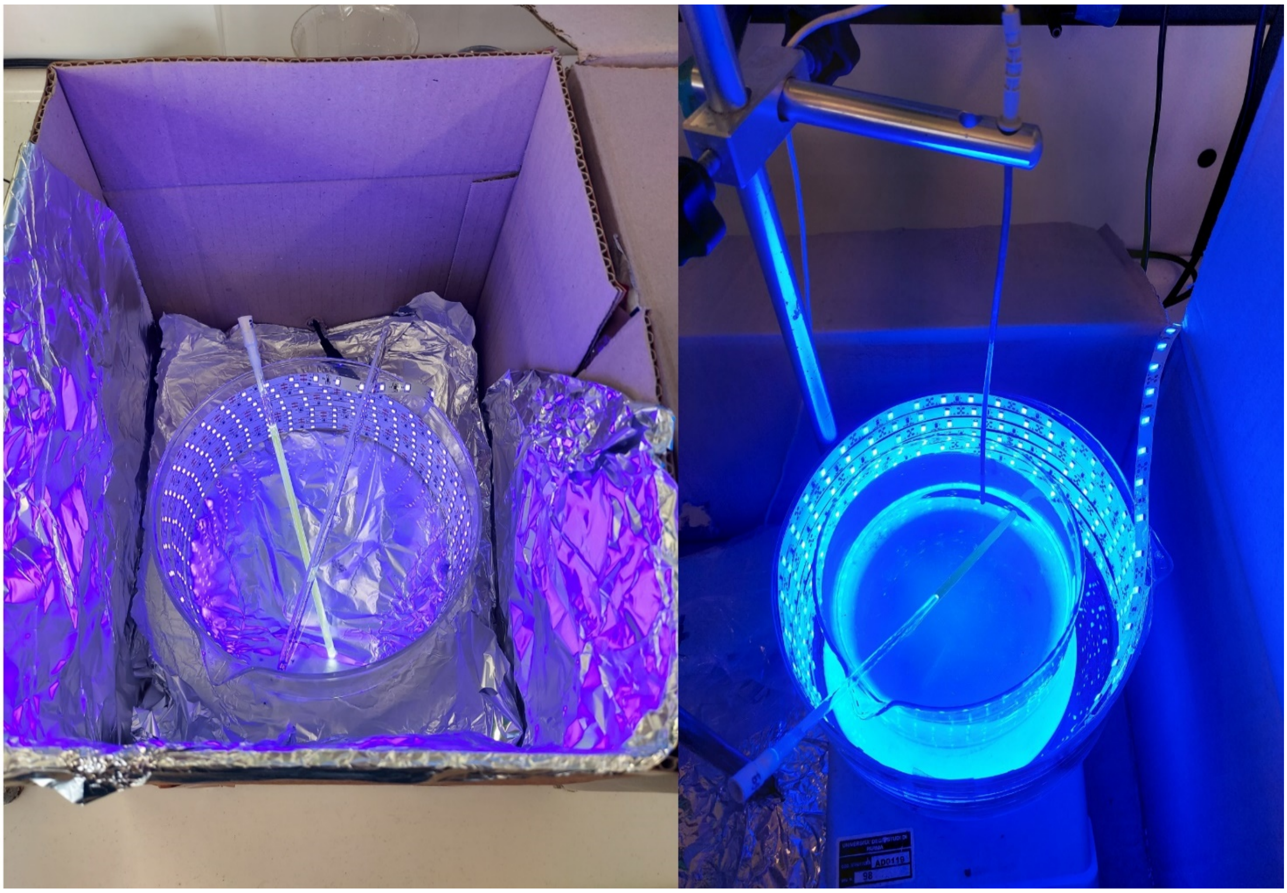

Left: setup using Violet LEDs; Right: setup using Blue LEDs

Emission spectra of the LEDs used in this work

Emission spectra of light sources were measured with a Jeti Spectroradiometer specbos 1211UV

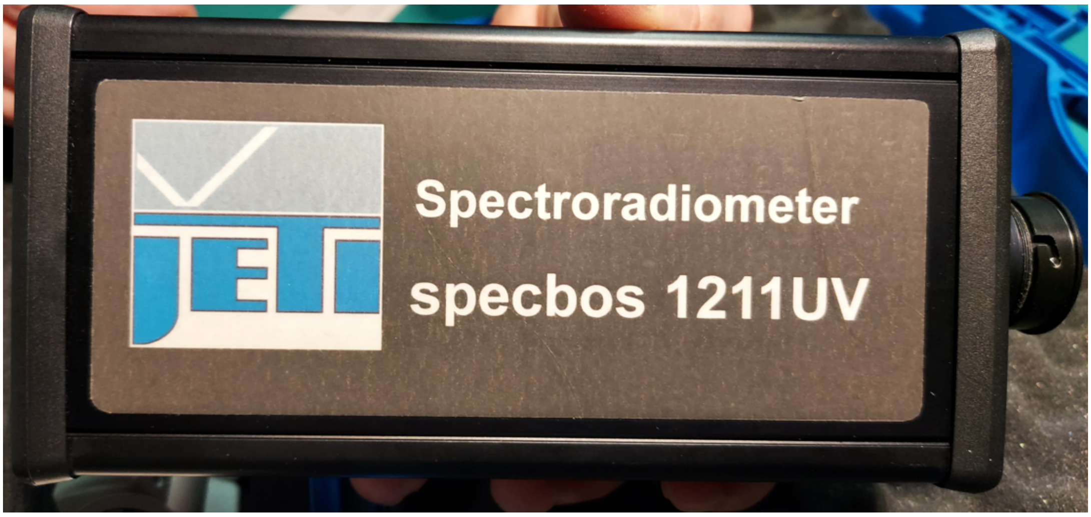

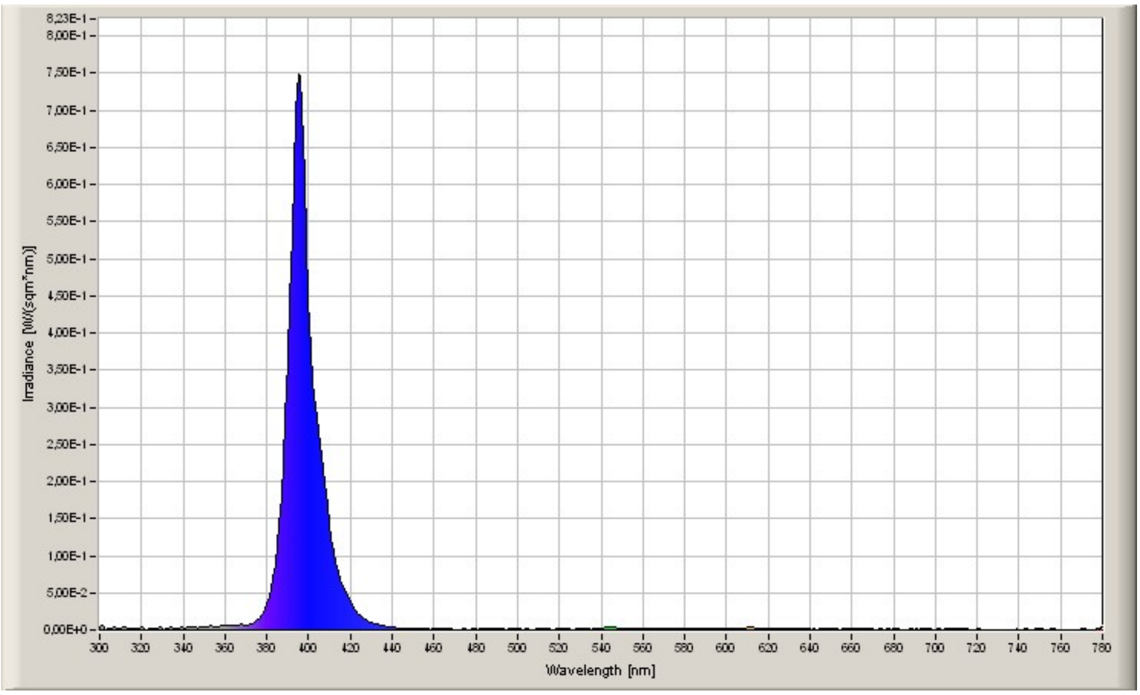

Emission spectra of the Violet LEDs light

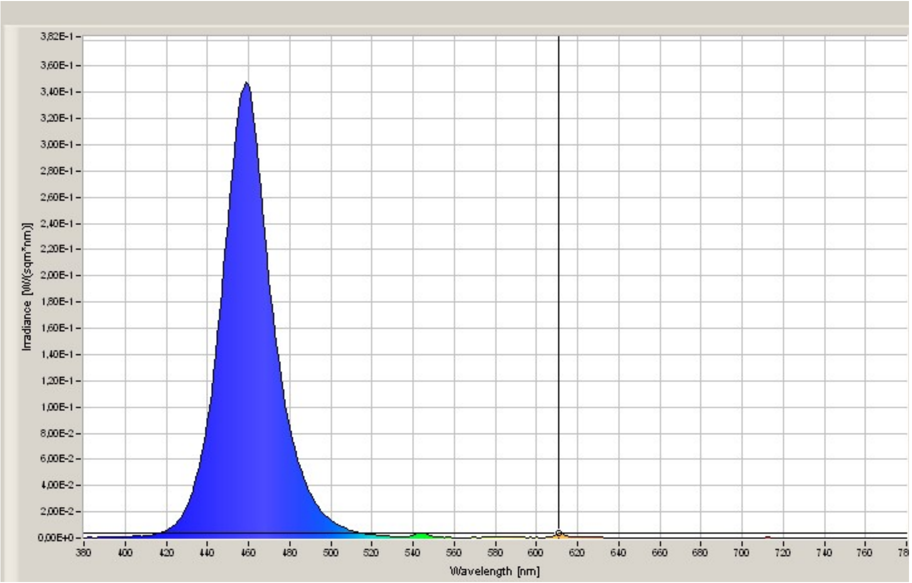

Emission spectra of the Blue LEDs light

Procedures for synthesis of the ligands

Procedure for the preparation of ligand L1$_{C^N}$

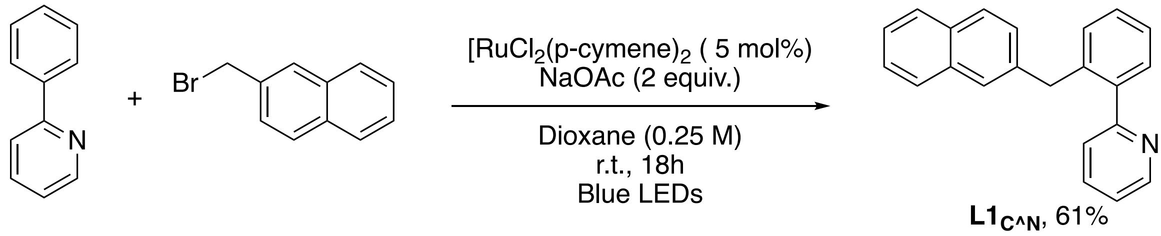

Dichloro(p-cymene)ruthenium(II) dimer (18 mg, 0.03 mmol, 5 mol%), sodium acetate (82 mg, 1 mmol, 2 equiv.) and 2-(Bromomethyl)naphthalene (110 mg, 0.5 mmol, 1 equiv.) were placed in a sealed vial, equipped with a stirring bar, under Nitrogen atmosphere. 1,4-Dioxane (2 mL, 0.25 M) and 2-Phenylpyridine **1a** (216 µL, 1.5 mmol, 1.5 equiv.) were then added and the resulting mixture was stirred at r.t. for 18h under blue LEDs irradiation (420-500 nm). The mixture was filtered through Celite and then the crude product was purified by chromatography on silica gel (Hexane:AcOEt 9:1) affording pure **L1$_{C^N}$** (89.6 mg, 61% yield).

^{1}H NMR (400 MHz, $CDCl_3$) δ 8.71 (ddd, J = 4.9, 1.8, 1.0 Hz, 1H), 7.78 – 7.73 (m, 1H), 7.69 – 7.60 (m, 3H), 7.44 – 7.38 (m, 3H), 7.38 – 7.31 (m, 3H), 7.29 – 7.25 (m, 2H), 7.23 (ddd, J = 7.6, 4.9, 1.2 Hz, 1H), 7.16 (dd, J = 8.5, 1.8 Hz, 1H), 4.29 (s, 2H). **^{13}C NMR** (101 MHz, $CDCl_3$) δ 160.1, 149.3, 140.8, 138.9, 138.8, 136.4, 133.6, 132.0, 130.9, 130.1, 128.6, 127.9, 127.8, 127.7, 127.7, 127.2, 126.6, 125.9, 125.3, 124.4, 121.9, 39.1. **ESI-MS** calcd for $C_{22}H_{17}N$ $[M+H]^+$ 296.14, found 296.25.

Procedure for the preparation of ligand L2$_{C^N}$

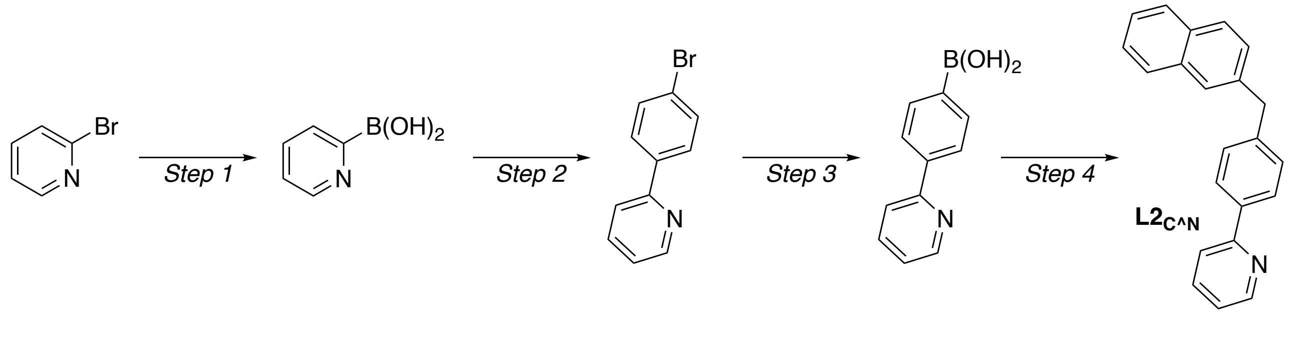

Step 1-2: 2-Bromopyridine (1.58 g, 10 mmol, 1 equiv.) was dissolved in dry Et_2O (25 mL, 0.4 M). The solution was cooled down to -78°C, then *n*BuLi 2.5 M was added (4 mL, 10 mmol, 1 equiv.) and the mixture was stirred for 30 minutes. $B(OMe)_3$ (2.08 g, 20 mmol, 2 equiv.) was added over 30 minutes at -78 °C, then the mixture was left stirring overnight, gradually reaching room temperature. The solvents were removed under reduced pressure, and MeOH was added and again removed, under reduced pressure. The crude boronic ester was directly treated with 1-Bromo-4-Iodobenzene (2.55 g, 9 mmol, 0.9 equiv.), $CsCO_3$ (5.29 g, 15 mmol, 1.5 equiv.) and $[Pd(PPh_3)_4]$ (346 mg, 0.3 mmol, 30 mol%) in Toluene (, 0.35 M) at 120°C for 18h. Then, the mixture was filtered over a pad of celite and purified by chromatography on silica gel (Hexane:AcOEt 9:1) affording 2-(4-bromophenyl)pyridine (489 mg, 2.1 mmol, 21% yield over 2 steps).

^{1}H NMR (400 MHz, $CDCl_3$) δ 8.69 (ddd, J = 4.9, 1.8, 1.0 Hz, 1H), 7.90 – 7.85 (m, 2H), 7.76 (ddd, J = 7.9, 7.3, 1.8 Hz, 1H), 7.70 (dt, J = 8.0, 1.2 Hz, 1H), 7.62 – 7.58 (m, 2H), 7.29 – 7.21 (m, 1H). Spectroscopic data are consistent with literature[4].

Step 3: 2-(4-bromophenyl)pyridine (489 mg, 2.1 mmol, 1 equiv.) was dissolved in THF (3 mL, 0.65 M). The solution was cooled down to -78°C, then *n*BuLi 2.5 M was added (1.08 mL, 2.7 mmol, 1.3 equiv.), the mixture was stirred at the same temperature for 1 hour. $B(OMe)_3$ (654 mg, 6.3 mmol, 3 equiv.) was added over 30 minutes at -78°C, the mixture was left stirring overnight, and allowed to return at room temperature gradually overnight. The reaction was quenched with water and the product was extracted with CH_2Cl_2. Solvents were partially removed under reduced pressure and petroleum ether was added forcing the precipitation of the boronic acid (210 mg, 1.05 mmol, 50% yield), which was filtered off.

Step 4: The boronic acid previously obtained was treated with 2-(Bromomethyl)naphthalene (232 mg, 1.05 mmol, 1 equiv.), $CsCO_3$ (565 mg, 1.6 mmol, 1.5 equiv.) and $[Pd(PPh_3)_4]$ (60 mg, 0.052 mmol, 5 mol%) in Toluene (3 mL, 0.35 M) at 100 °C for 18 hours. The mixture was filtered over a pad of celite and purified by chromatography on silica gel (Hexane:AcOEt 9:1) affording **L2$_{C^N}$** (69 mg, 0.233 mmol, 22% yield).

^{1}H NMR (400 MHz, $CDCl_3$) δ 8.71 – 8.67 (m, 1H), 7.96 – 7.92 (m, 2H), 7.83 – 7.76 (m, 3H), 7.75 – 7.69 (m, 2H), 7.66 (bs, 1H), 7.51 – 7.41 (m, 2H), 7.38 – 7.32 (m, 3H), 7.21 (ddd, J = 6.7, 4.9, 1.7 Hz, 1H), 4.21 (s, 2H). **^{13}C NMR** (101 MHz, $CDCl_3$) δ 157.4, 149.7, 142.1, 138.5, 137.4, 137.0, 133.7, 132.2, 129.7, 128.3, 127.8, 127.7, 127.7, 127.3, 127.2, 126.1, 125.5, 122.1, 120.6, 42.0. **ESI-MS** calcd for $C_{22}H_{17}N$ $[M+H]^+$ 296.14, found 296.20.

Procedure for the preparation of ligand L3$_{C^N}$

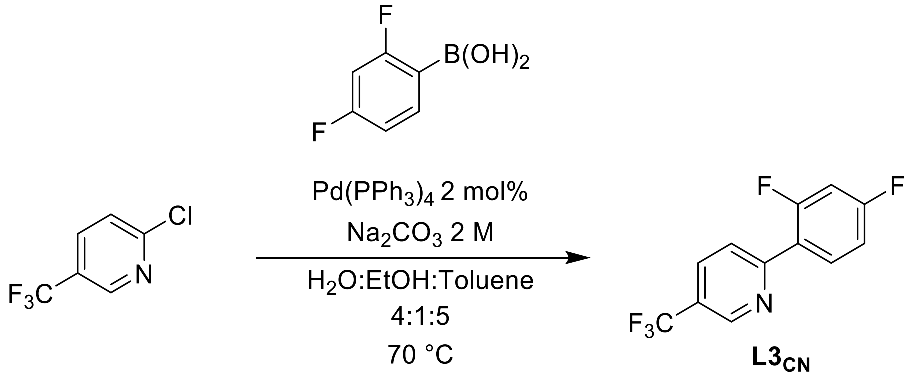

Fluorinated ligand 2-phenylpyridine **L3$_{CN}$** was synthetized adapting a literature procedure.[62]

To a flame dried 100 mL Schlenk, commercially available 2-chloro-5-(trifluoromethyl)pyridine (726 mg, 4 mmol, 1 equiv.) and (2,4-difluorophenyl)boronic acid (947 mg, 6 mmol, 1.5 equiv.) were added under nitrogen. Then toluene, ethanol (11 mL + 2 mL) and a solution of 2 M Na_2CO_3 (10 mL) were added. The mixture was stirred for 10 minutes, then [$Pd(PPh_3)_4$] (92.4 mg, 0.08 mmol, 2 mol%) was added. The resulting mixture was stirred at 70 °C until complete conversion (typically 48 hours). The mixture was then allowed to return to room temperature and the solvent removed under reduce pressure. The solid residue was suspended in water (60 mL) and extracted with DCM (3x 20 mL). The organic phases were collected, dried over Na_2SO_4 and filtered. The crude concentrated and purified by flash chromatography on silica gel (100% Hexane to Hexane:AcOEt 55:45) affording **L3$_{CN}$** as a white solid. (900 mg, 3.5 mmol, 88% yield).

^{1}H NMR (400 MHz, $CDCl_3$) δ 8.96 (s, 1H), 8.10 (td, J = 8.8, 6.5 Hz, 1H), 7.99 (dd, J = 8.4, 2.4 Hz, 1H), 7.93 – 7.88 (m, 1H), 7.04 (tdd, J = 8.7, 2.5, 1.0 Hz, 1H), 6.95 (ddd, J = 11.3, 8.7, 2.5 Hz, 1H). **^{13}C NMR** (101 MHz, $CDCl_3$) δ 165.2 (d, J = 12.4 Hz), 162.6 (d, J = 12.4 Hz), 162.2 (d, J = 12.0 Hz), 159.7 (d, J = 12.3 Hz), 155.8, 146.6 (d, J = 4.3 Hz), 133.8 (d, J = 3.6 Hz), 132.5 (dd, J = 9.8, 4.1 Hz), 125.9 – 124.57 (m), 123.7 (d, J = 10.7 Hz), 122.4 (d, J = 27.5 Hz), 112.28 (dd, J = 21.3, 3.7 Hz), 104.6 (t, J = 26.2 Hz). **^{19}F NMR** (565 MHz, $CDCl_3$) δ -62.3, -107.1 (p, J = 8.4 Hz), -111.9 (q, J = 9.9 Hz). **ESI-MS** calcd for $C_{22}H_{17}N$ $[M+H]^+$ 259.042, found 259.430.

Procedure for the preparation of ligand L6$_{N^{\wedge}N}$

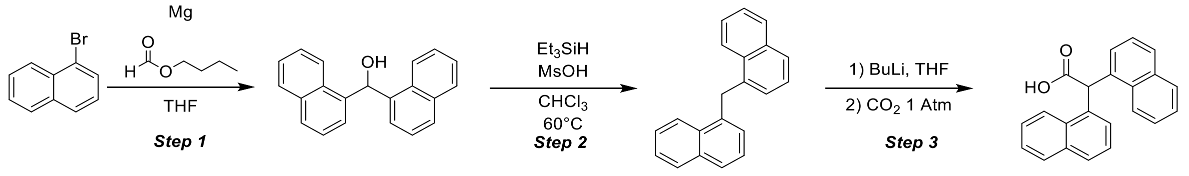

2,2-di(naphthalen-1-yl)acetic acid was synthetized following a modified literature procedure.[63,64]

Step 1: To a dried oven flask was introduced magnesium turnings (362 mg, 15 mmol, 1.05 equiv.) followed by THF (20 mL). Few drops of di-bromo-ethane were added in concomitance with a dropwise addition of a solution of 1-bromo-methyl-naphthalene (2.9 g, 14.4 mmol, 1 equiv.) in THF (10 mL, 1.44 M) maintaining the solution hot. Once the addition is ended, the resulting mixture was stirred at room temperature for two hours. Then, a solution of butyl formiate in THF (661 mg, 0.74 mL, 6.48 mmol, 0.45 eq) was slowly added. The resulting solution was stirred overnight at room temperature. The reaction was quenched by the slowly addition of a saturated solution of NH_4Cl (20 mL). The two phases were separated, and the water phase was extracted with diethyl ether (30 mL x 3). The organic phase was dried over Na_2SO_4 and the solvent was removed under reduced pressure. The residue was purified by chromatography on silica gel (exane:EtOAc 15:1) affording di(naphthalen-1-yl)methanol as a white solid (1.5 g, 5.3 mmol, 81% yield).
^{1}H NMR (400 MHz, $CDCl_3$) δ 8.07 (dd, J = 8.4, 1.3 Hz, 2H), 7.97 – 7.88 (m, 2H), 7.88 – 7.77 (m, 2H), 7.58 – 7.38 (m, 8H), 7.33 (s, 1H), 2.41 (bs, 1H). *Data consistent with literature.*[63]

Step 2: Di(naphthalen-1-yl)methanol (1.0 g, 3.5 mmol, 1 equiv.) was dissolved in chloroform (22 mL, 0.16 M), then MsOH (0.25 mL, 3.85 mmol, 1.1 equiv.) was added at 0 °C followed by Et_3SiH (0.9 mL, 5.6 mmol, 1.6 equiv.) was added dropwise under nitrogen. The resulting solution was stirred at 60 °C for 1 hour, then was quenched by the addition of a saturated aqueous solution of $NaHCO_3$ and extracted with DCM (10 mL x3). The organic phase was dried over Na_2SO_4, the solvent was removed under reduced pressure. The residue was purified by chromatography on silica gel (exane:EtOAc 30:1) affording di(naphthalen-1-yl)methane as a white solid (360 mg, 1.3 mmol, 38% yield).
^{1}H NMR (400 MHz, $CDCl_3$) δ 8.06 (d, J = 7.5 Hz, 2H), 7.92 (dd, J = 7.5, 1.6 Hz, 2H), 7.78 (d, J = 8.1 Hz, 2H), 7.58 – 7.45 (m, 4H), 7.36 (tt, J = 7.1, 1.3 Hz, 2H), 7.13 – 7.06 (m, 2H), 4.90 (s, 2H). *Data consistent with literature.*[64]

Step 3: Di(naphthalen-1-yl)methane (360 mg, 1.3 mmol, 1 equiv.) was dissolved in THF (10 mL, 0.1 M) under nitrogen. Then a solution of *n*BuLi 2.5 M in hexane (0.98 mL, 1.7 mmol, 1.3 equiv.) was slowly added at room temperature. Then the vessel equipped with a balloon charged with CO_2 and purged 3 times. (The solution was frozen in liquid nitrogen, the vessel connected to a vacuum line and the atmosphere replaced with CO_2). The solution was stirred for 12 h at room temperature. At the completion of the reaction, 2 N NaOH was added to the solution and stirred for 0.5 h. The mixture was extracted with diethyl ether (2x 20 mL) and the aqueous phase acidified with HCl. The resulting suspension was extracted with EtOAC (3 x 30 mL), washed with water and dried with Na_2SO_4, then the solvent was removed under reduced pressure. The resulting solid was purified by crystallization in DCM/Hexane affording 2,2-di(naphthalen-1-yl)acetic acid as a white solid (160 mg, 0.4 mmol, 30% yield).

^{1}H NMR (400 MHz, $CDCl_3$) δ 12.64 (brs, 1H) 8.01 – 7.93 (m, 2H), 7.91 (dd, J = 7.4, 2.0 Hz, 2H), 7.83 (d, J = 8.1 Hz, 2H), 7.56 – 7.44 (m, 4H), 7.40 (t, J = 7.7 Hz, 2H), 7.33 (dd, J = 7.2, 1.2 Hz, 2H), 6.55 (s, 1H). *Data consistent with literature.*[64]

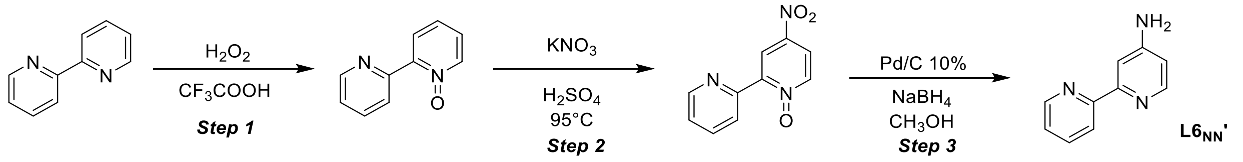

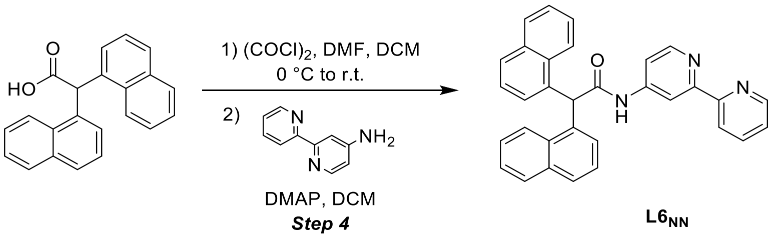

Ligand **L6$_{NN}$** was synthetized adapting a literature procedure.[65]

Step 1: Commercially available 2,2'-bipyridine (5.0 g, 32 mmol, 1 equiv.) was dissolved in trifluoroacetic acid (25 mL, 0.2 M) and an aqueous solution of 30 % hydrogen peroxide (5 mL, 166 mmol, 5.1 equiv.) was slowly added. The resulting solution was stirred at room temperature until complete consumption of the 2,2'-bipyridine (monitored by GC-MS). At the completion of the reaction, the pH of the solution was brought to 9 by the addition of a 25 % solution of NaOH. The resulting mixture was extracted with chloroform (30 mL x5) and dried with with Na_2SO_4. The solvent was removed under reduced pressure affording [2,2'-bipyridine] 1-oxide as a slightly pink solid in quantitative yield which was used without further purification or characterization.

Step 2: [2,2'-bipyridine] 1-oxide (3.0 g, 17 mmol, 1 equiv.) and KNO_3 (9.4 g, 93 mmol, 5.4 equiv.) were dissolved in 98% sulphuric acid (23 mL, 0.75 M) and heated to 95 °C for 48 hours. Then the reaction mixture was allowed to return to room temperature and poured in a 250 mL beaker with 100g of ice. The pH of the solution is then brought to a value of 9 by the dropwise addition of a 25 % solution of NaOH. The resulting suspension is extracted with DCM (50 mL x5), the organic phase was dried over Na_2SO_4 and then the solvent was removed under reduced pressure. The resulting solid was purified with by flash chromatography on silica gel ($CHCl_3$) affording 4-nitro-[2,2'-bipyridine] 1-oxide as a yellow solid (1.4 g, 6.5 mmol, 38 % yield).

^{1}H NMR (400 MHz, $CDCl_3$) δ 9.14 (d, *J* = 3.3 Hz, 1H), 8.86 (d, *J* = 8.1 Hz, 1H), 8.82 – 8.73 (m, 1H), 8.35 (d, *J* = 7.1 Hz, 1H), 8.06 (dd, *J* = 7.2, 3.3 Hz, 1H), 7.88 (t, *J* = 7.8 Hz, 1H), 7.49 – 7.37 (m, 1H). *Data consistent with literature.*[65]

Step 3: A two neck 250 mL round bottom flask was charged with 4-nitro-[2,2'-bipyridine] 1-oxide (1.1 g, 5 mmol, 1 equiv.) under nitrogen. Methanol (100 mL, 0.05 M) was added and solid was dissolved under vigorous stirring. The solution was then cooled to 0 °C and 10% Pd/C (230 mg, 5 mol %) was added. $NaBH_4$ (2.5 g, 60 mmol, 12 equiv.) was added portion wise with careful control of the temperature during the addition. The resulting mixture is stirred for 6 hours at 0°C. At the completion of the reaction, the resulting mixture was filtered to remove the catalyst and the methanol removed under reduced pressure. The resulting solid was suspended in 30 mL of water and extracted with Et_2O (50 mL x5), the organic phase was dried with Na_2SO_4 and then the solvent was removed under reduced pressure to afford [2,2'-bipyridin]-4-amine as a white solid (663 mg, 3.9 mmol 77 % yield) which was used without further purification.

^{1}H NMR (400 MHz, $CDCl_3$) δ 8.64 (ddd, *J* = 4.8, 1.8, 0.9 Hz, 1H), 8.35 (dt, *J* = 8.0, 1.1 Hz, 1H), 8.30 (d, *J* = 5.5 Hz, 1H), 7.78 (td, *J* = 7.7, 1.8 Hz, 1H), 7.67 (d, *J* = 2.4 Hz, 1H), 7.38 – 7.25 (m, 1H), 6.54 (dd, *J* = 5.5, 2.4 Hz, 1H), 4.26 (brs, 2H). *Data consistent with literature.*[65]

Step 4: A 25 mL two neck round bottom flask equipped with a magnetic stirred bar, under nitrogen was charged with 2,2-di(naphthalen-1-yl)acetic acid (50 mg, 0.16 mmol, 1 equiv.) and dissolved in dichloromethane (1.6 mL, 0.1 M). The solution was cooled to 0 °C and oxalyl chloride (20 µL, 0.2 mmol, 1.2 equiv.) was added followed by 2 drops of DMF. The resulting solution was stirred at room temperature until completed conversion of the stating material (monitored by TLC), then the solvent was removed under vacuum. The resulting oil was dissolved in 2 mL of DCM and a catalytic amount of DMAP was added. Then to this solution was added dropwise at 0 °C a second solution prepared dissolving [2,2'-bipyridin]-4-amine (27.3 mg, 0.16 mmol, 1 equiv.) and triethylamine (48.4 mg, 0.07 mL, 3 equiv.) in DCM (2 mL, 0.08 M). After the addition, the resulting solution was stirred at room temperature overnight. At the competition of the reaction the solvent was removed under reduced pressure and the crude product purified by chromatography on silica gel (DCM: MeOH:NH_4OH 9:1:0.01) affording N-([2,2'-bipyridin]-4-yl)-2,2-di(naphthalen-1-yl)acetamide **L6**$_{\mathbf{N^\wedge N}}$ as a white solid (33 mg, 0.07 mmol, 44% yield).

^{1}H NMR (400 MHz, $CDCl_3$) δ 8.62 – 8.51 (m, 2H), 8.35 (dt, *J* = 8.1, 1.1 Hz, 1H), 8.09 (dd, *J* = 5.6, 2.2 Hz, 1H), 7.97 – 7.82 (m, 7H), 7.78 (td, *J* = 7.8, 1.8 Hz, 1H), 7.68 (s, 1H), 7.56 – 7.38 (m, 6H), 7.28 (d, *J* = 7.5 Hz, 3H), 6.56 (s, 1H). **^{13}C NMR** (101 MHz, $CDCl_3$) 171.5, 157.0, 155.6, 150.7, 149.0 (2C), 145.5, 137.1, 134.2 (2C), 134.2 (2C), 131.7, 129.2 (2C), 129.0 (2C), 127.2 (2C), 126.7 (2C), 126.2 (2C), 125.7 (2C), 124.0, 123.1 (2C), 121.2, 113.7, 110.4, 54.1. **ESI-MS** calcd for $C_{32}H_{23}N_3O$ [M^+], 465.18 found 465.45.

Procedure for the preparation of ligand L7$_{N^N}$

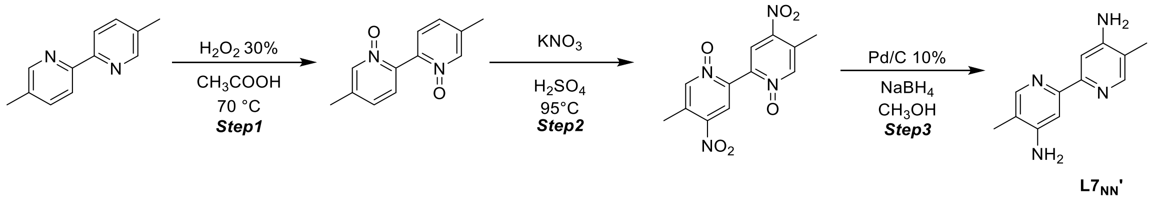

Step 1-To a round bottom flask equipped with a reflux condenser, 5,5'-dimethyl-2,2'-bipyridine (921 mg, 5 mmol, 1 equiv.) was added followed by glacial acetic acid (7 mL, 0.7 M). Then a solution of 30% hydrogen peroxide (1.4 mL, 13.7 mmol, 2.7 equiv.) was added dropwise at 0 °C. The resulting solution was heated to 70-80 °C for 30 min after which an additional 1 mL of solution of 30% hydrogen peroxide was added. The resulting mixture was refluxed overnight. The solution was poured in acetone (20 mL) and the solvent removed under reduced pressure until a white precipitate appear. The solid was recovered by vacuum filtration, washed with cold acetone and diethyl ether affording 5,5'-dimethyl-[2,2'-bipyridine] 1,1'-dioxide as a white solid (960 mg, 4.4 mmol, 89% yield).

^{1}H NMR (400 MHz, $CDCl_3$) δ 8.20 – 8.18 (m, 2H), 7.58 (d, *J* = 8.1 Hz, 2H), 7.14 (ddd, *J* = 8.2, 1.7, 0.8 Hz, 2H), 2.35 (s, 6H). **^{13}C NMR** (101 MHz, $CDCl_3$) δ 140.3 (2C), 139.6 (2C), 137.6 (2C), 128.2 (2C), 126.5 (2C), 18.7 (2C). **ESI-MS** calcd for $C_{12}H_{12}N_2O_2$ [M^+], 216.09, found 216.17. *Data consistent with literature.*[66]

Step 2-In a 100 mL Schlenk, 5,5'-dimethyl-[2,2'-bipyridine] 1,1'-dioxide (900 mg, 4.1 mmol, 1 equiv.) and KNO_3 (4.5 g, 45 mmol, 10.8 equiv.) were dissolved in 98% sulphuric acid (20 mL, 0.2 M). The resulting suspension was stirred at 95 °C for 30 hours. Then, the solution was allowed to return to room temperature and poured on 75g of ice in a 250 mL beaker. As the ice melted, a yellow precipitate formed, which was collected upon vacuum filtration and washed with cold water and diethyl ether and dried under vacuum affording 5,5'-dimethyl-4,4'-dinitro-[2,2'-bipyridine] 1,1'-dioxide as a yellow solid (370 mg, 1.20 mmol 28% yield).

^{1}H NMR (400 MHz, DMSO) δ 8.69 (s, 2H), 8.57 (s, 2H), 2.62 (s, 6H). *Data consistent with literature.*[66]

Step 3-A two neck 250 mL round bottom flask under nitrogen, was charged with 5,5'-dimethyl-4,4'-dinitro-[2,2'-bipyridine] 1,1'-dioxide (370 mg, 1.2 mmol, 1 equiv.). Methanol (50 mL, 0.02 M) was added, and the resulting suspension cooled to 0 °C. Then 10% Pd/C (12 mg, 10 mol%) was added. $NaBH_4$ (1.9 g, 28 mmol, 24 equiv.) was added portion wise with careful control of the temperature during the addition. The resulting mixture was stirred for 6 hours at 0 °C. At the completion of the reaction, the resulting mixture was filtered to remove the catalyst and the methanol removed under reduced pressure. The resulting solid was suspended in 30 mL of water and extracted with Et_2O (50 mL x5), the organic phase was dried with Na_2SO_4, then the solvent was removed under reduced pressure to afford 5,5'-dimethyl-[2,2'-bipyridine]-4,4'-diamine **L7$_{N^N}$'** as a white solid (98 mg, 0.46 mmol 39 % yield) which was directly used without further purification.

^{1}H NMR (600 MHz, DMSO-*d$_6$*) δ 7.96 (s, 2H), 7.58 (s, 2H), 5.81 (s, 4H), 2.07 (s, 6H). **^{13}C NMR** (101 MHz, DMSO-*d$_6$*)) δ 155.44 (2C), 153.96 (2C), 149.77 (2C), 116.72 (2C), 105.60 (2C), 14.97 (2C). **ESI-MS** calcd for $C_{12}H_{15}N_4$ [M+H]$^+$, 215.13 found 215.16.

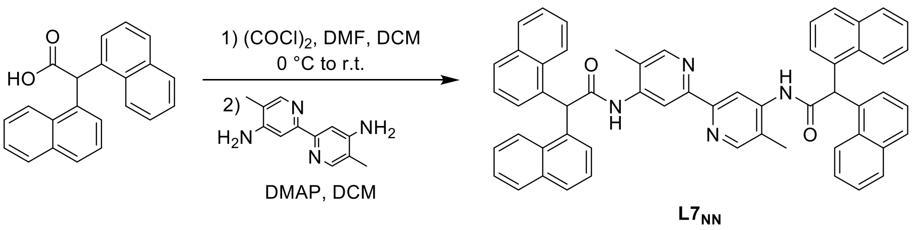

A 25 mL two neck round bottom flask equipped with a magnetic stirred bar, under nitrogen was charged with 2,2-di(naphthalen-1-yl)acetic acid (106.18 mg, 0.34 mmol, 1 equiv.) and dissolved in dichloromethane (3 mL, 0.1 M). The solution was cooled to 0 °C and oxalyl chloride (0.036 mL, 0.42 mmol, 1.2 equiv.) was added followed by 2 drops of DMF. The resulting solution was stirred at room temperature until completed conversion of the stating material (monitored by TLC), then the solvent was removed under vacuum. The resulting oil was dissolved in 3 mL of DCM and a catalytic amount of DMAP was added. Then to this solution was added dropwise at 0 °C a second solution prepared dissolving 5,5'-dimethyl-[2,2'-bipyridine]-4,4'-diamine (35 mg, 0.16 mmol, 0.5 equiv.) and triethylamine (120 mg, 0.170 mL, 3.5 equiv.) in DCM (2 mL, 0.08 M). After the addition, the resulting solution was stirred at room temperature overnight. At the competition of the reaction, monitored by TLC, the solvent was removed under reduced pressure affording N,N'-(5,5'-dimethyl-[2,2'-bipyridine]-4,4'-diyl)bis(2,2-di(naphthalen-1-yl)acetamide) **L7**$_{\mathbf{N^\wedge N}}$ as an orange solid (33 mg, 0.07 mmol, 44% yield) which was used without further purification. *The presence of N,N'-(5,5'-dimethyl-[2,2'-bipyridine]-4,4'-diyl)bis(2,2-di(naphthalen-1-yl)acetamide) in the reaction crude was confirmed by LC-MS analysis*: **ESI-MS** calc for $C_{56}H_{43}N_4O_2^+$ $[M+H]^+$ 803.339, found 803.284

Procedure for the preparation of ligand L8$_{N^\wedge N}$

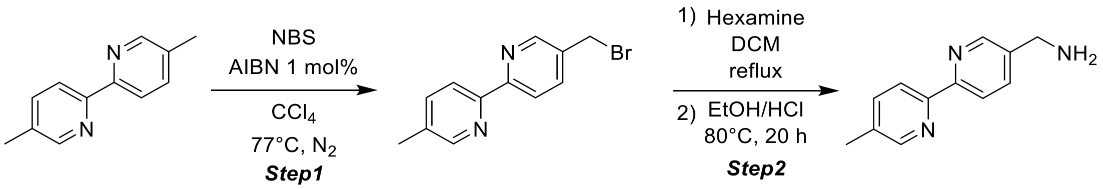

Step1- In a 100 mL Schlenk flask equipped with a magnetic stirring bar 5,5'-dimethyl-2,2'-bipyridine (500 mg, 2.7 mmol, 1 eq) was dissolved in CCl_4 (20 mL, 0.1M) under nitrogen. Then AIBN (5 mg, 0.027 mmol) was added. The resulting solution was refluxed under nitrogen. After 17 h, the mixture was filtered while hot and the filtrate was left at 4°C for 48 hours to allow the dibromo product to slowly crystallize from the solution. The solution was then filtered and the crystals washed with cold CCl_4, then the solvent was removed under reduced pressure to afford 5-(bromomethyl)-5'-methyl-2,2'-bipyridine (500 mg, 1.9 mmol, 70% yield) as a white solid which was directly used without further purifications.

^{1}H NMR (400 MHz, $CDCl_3$) δ 8.65 (d, J = 2.3 Hz, 1H), 8.49 (d, J = 2.3 Hz, 1H), 8.38 – 8.32 (m, 1H), 8.27 (d, J = 8.0 Hz, 1H), 7.82 (dd, J = 8.2, 2.4 Hz, 1H), 7.60 (ddt, J = 8.0, 5.7, 1.4 Hz, 1H), 4.52 (s, 2H), 2.38 (s, 3H).

Step2- In a 25 mL round bottom flask equipped with a magnetic stirrer bar, hexamine (308 mg, 2.2 mmol) was dissolved in dry DCM (5 mL). The solution was refluxed for 1h, then a solution of 5-(bromomethyl)-5'-methyl-2,2'-bipyridine (500 mg, 1.9 mmol) in DCM (10 mL, 0.2 M) was slowly added. Upon the addition, a formation of a white precipitate is noted, and the resulting suspension is refluxed for 20 h. At the end, the suspension is filtered and washed with cold DCM. The precipitate is then suspended in ethanol and a concentrate solution of HCl 37% (2 mL) is added dropwise. The suspension is then heated at 80°C overnight. Then the solution is cooled to room temperature and the solvent removed under reduced pressure. The residue was suspended in water (9 mL) and $CHCl_3$ (10 mL). Then a solution of NaOH 10 M is added upon pH 13 and extracted with DCM (3x 20 mL). The organic phase was dried with Na_2SO_4, filtered and the solvent removed under reduced pressure to afford (5'-methyl-[2,2'-bipyridin]-5-yl)methanamine (210 mg, 1.05 mmol, 56% yield) as a white solid which was used without any further purification.

^{1}H NMR (400 MHz, $CDCl_3$) δ 8.60 (d, J = 2.3 Hz, 1H), 8.51 – 8.47 (m, 1H), 8.32 (d, J = 8.1 Hz, 1H), 8.26 (d, J = 8.1 Hz, 1H), 7.78 (dd, J = 8.2, 2.3 Hz, 1H), 7.61 (dd, J = 8.1, 2.3 Hz, 1H), 3.95 (s, 2H), 2.38 (s, 3H), 1.46 (s, 2H). **^{13}C NMR** (101 MHz, $CDCl_3$) δ 155.3, 153.7, 149.8, 148.4, 138.2, 137.6, 135.9, 133.4, 120.7, 120.6, 44.0, 18.5. **ESI-MS** calcd for $C_{12}H_{13}N_3$ [M^+], 129,11 found 129.23.

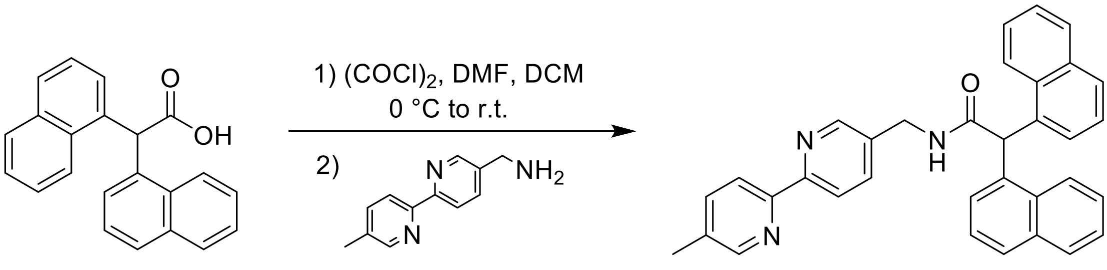

L8$_{NN}$

Step3-A 25 mL two neck round bottom flask equipped with a magnetic stirred bar, under nitrogen was charged with 2,2-di(naphthalen-1-yl)acetic acid (78 mg, 0.25 mmol, 1 eq) and dissolved in dichloromethane (2.5 mL, 0.1 M). The solution was cooled to 0 °C and oxalyl chloride (53.4 mg, 0.036 mL, 0.42 mmol, 1.2 eq) was added followed by 2 drops of DMF. The resulting solution was stirred at room temperature until completed conversion of the stating material (monitored by TLC), then the solvent was removed under vacuum. The resulting oil was dissolved in 3 mL of DCM and a catalytic amount of DMAP was added. Then to this solution was added dropwise at 0 °C a second solution prepared dissolving (5'-methyl-[2,2'-bipyridin]-5-yl)methanamine (47 mg, 0.24 mmol, 0.95 eq) and triethylamine (0.104 mL, 3.5 eq) in DCM (2 mL, 0.08 M). After the addition, the resulting solution was stirred at room temperature overnight. At the competition of the reaction, monitored by TLC, the solvent was removed under reduced pressure and the residue purified by flash column chromatography on silica gel (DCM:MeOH gradient) affording *N*-((5'-methyl-[2,2'-bipyridin]-5-yl)methyl)-2,2-di(naphthalen-1-yl)acetamide **L8$_{NN}$** as a white solid (30 mg, 0.06 mmol, 26% yield).

^{1}H NMR (400 MHz, $CDCl_3$) δ 8.50 (d, *J* = 2.2 Hz, 1H), 8.42 (d, *J* = 2.2 Hz, 1H), 8.27 (d, *J* = 8.1 Hz, 1H), 8.22 (d, *J* = 8.2 Hz, 1H), 7.97 – 7.83 (m, 4H), 7.79 (d, *J* = 8.2 Hz, 2H), 7.66 (dd, *J* = 8.2, 2.2 Hz, 1H), 7.52 – 7.39 (m, 5H), 7.35 (dd, *J* = 8.2, 7.2 Hz, 2H), 7.21 (d, *J* = 7.1 Hz, 2H), 6.50 (s, 1H), 6.35 (*brt*, *J* = 6.0 Hz, 1H), 4.51 (d, *J* = 6.0 Hz, 2H), 2.40 (s, 3H). **^{13}C NMR** (101 MHz, $CDCl_3$) δ 172.8, 154.2 (2C), 152.3 (2C), 148.9, 148.2, 138.6, 136.8, 135.0 (2C), 134.2 (2C), 131.9 (2C), 129.1 (2C), 128.6 (2C), 127.0 (2C), 126.8 (2C), 126.2 (2C), 125.7 (2C), 123.4 (2C), 121.3, 121.1, 52.8, 41.2, 18.5. **ESI-MS** calcd for $C_{34}H_{28}N_3O$ $[M+H]^+$, 494.22 found 494.30.

Procedures for the preparation of Iridium Dimers 2a-2d

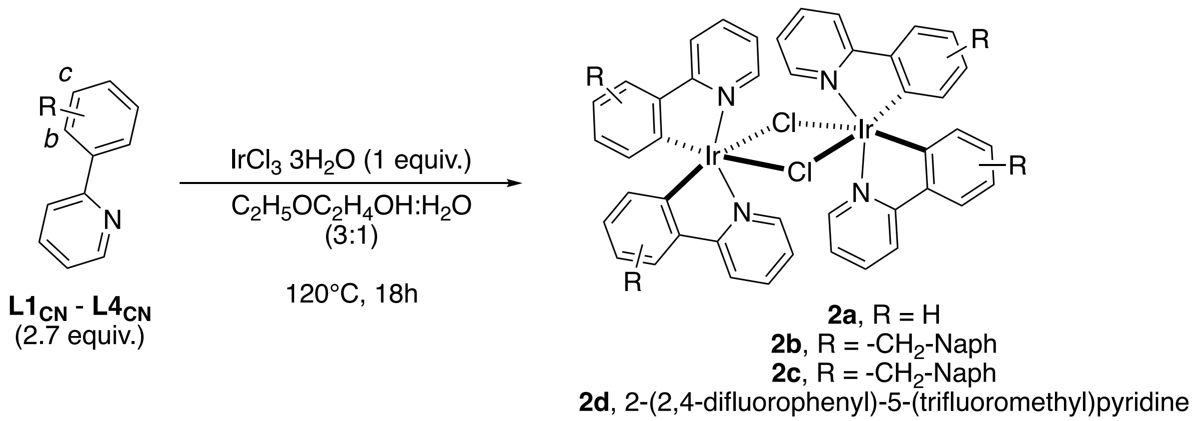

Dimers **2a-c** were synthetized adapting a literature procedure by Sun.[67]

2a: To a vial equipped with a magnetic stirrer under nitrogen $IrCl_3$ $3H_2O$ (178 mg, 0.5 mmol, 1 equiv.) was charged. A degassed solution of 2-Ethoxyethanol and water (12 mL, 3/1 v/v) was added followed by 2-phenylpyridine **L4**$_{C^\wedge N}$ (332 µL, 1.35 mmol, 2.7 equiv.). The mixture was stirred at 120 °C for 18 hours, then allowed to return to room temperature. Water was added and the resulting yellow precipitate was filtered off, washed with additional water and ethanol, and finally dried under reduced pressure affording pure **2a** $[Ir(ppy)_2Cl]_2$ (212 mg, 79% yield).

^{1}H NMR (400 MHz, $CDCl_3$) δ 9.24 (ddd, J = 5.8, 1.7, 0.8 Hz, 4H), 7.90 – 7.84 (m, 4H), 7.77 – 7.70 (m, 4H), 7.48 (dd, J = 7.8, 1.4 Hz, 4H), 6.80 – 6.71 (m, 8H), 6.56 (ddd, J = 7.8, 7.1, 1.4 Hz, 4H), 5.93 (dd, J = 7.8, 1.2 Hz, 4H). Spectroscopic data are consistent with literature.[68]

2b: To a vial equipped with a magnetic stirrer under nitrogen $IrCl_3$ $3H_2O$ (44.8 mg, 0.13 mmol, 1 equiv.) was charged. Degassed solution of 2-Ethoxyethanol and Water (2 mL, 3/1 v/v) was added followed by substituted 2-phenylpyridine **L1**$_{C^\wedge N}$ (108.8 mg, 0.37 mmol, 2.8 equiv.). The mixture was stirred at 120 °C for 18 hours, then allowed to return to room temperature. The yellow product is extracted from the mixture using CH_2Cl_2, then washed with water and finally dried over sodium sulfate. The crude product **2b** (450 mg, 1.64 mmol) is directly used for the synthesis of **PC1 - 5** since it is easier to purify by chromatography the Heteroleptic Iridium Complexes than the Iridium Dimers.

^{1}H NMR (400 MHz, $CDCl_3$) δ 9.09 (dd, J = 5.8, 1.7 Hz, 4H), 7.82 – 7.73 (m, 8H), 7.61 (d, J = 8.1 Hz, 4H), 7.51 (s, 4H), 7.45 – 7.23 (m, 20H), 6.72 – 6.58 (m, 8H), 5.96 (ddd, J = 7.3, 5.8, 1.3 Hz, 4H), 5.80 (dd, J = 7.7, 1.3 Hz, 4H), 4.68 (d, J = 17.4 Hz, 4H), 4.57 (d, J = 17.4 Hz, 4H). **^{13}C NMR** (101 MHz, $CDCl_3$) δ 167.9, 152.1, 149.2, 143.7, 137.8, 136.6, 135.7, 133.8, 132.1, 129.7, 128.4, 128.14, 128.10, 127.6, 127.4, 126.9, 126.8, 126.1, 125.4, 122.5, 121.3, 42.1.

2c: To a vial equipped with a magnetic stirrer under nitrogen $IrCl_3$ $3H_2O$ (17.6 mg, 0.05 mmol, 1 equiv.) was charged. Degassed solution of 2-Ethoxyethanol and Water (0.8 mL, 3/1 v/v) was added followed by substituted 2-phenylpyridine **L2**$_{C^N}$ (31.0 mg, 0.105 mmol, 2.1 equiv.). The mixture was stirred at 120 °C for 18 hours, then allowed to return to room temperature. The yellow product is extracted from the mixture using CH_2Cl_2, then washed with water and finally dried over sodium sulfate. The crude product **2c** (48 mg) is directly used for the following synthetic step, since it is easier to purify by chromatography the Heteroleptic Iridium Complexes than the Iridium Dimers.

Iridium Dimer **2d** was synthetized adapting a literature procedure.[62]

$IrCl_3$ $3H_2O$ (200 mg ,0.63 mmol,1 eq) and **L3**$_{C^N}$ (334 mg, 1.30 mmol, 2.05 eq) were placed in a sealed schlenk, equipped with a stirring bar, under Nitrogen atmosphere. A degassed 2/1 solution of 2-Ethoxyethanol and Water (12 mL) was added, and the mixture was stirred at 120°C for 48h, then cooled to r.t. Water was added (10 mL) and the resulting mixture stirred for 0.5 h. After that, the resulting yellow precipitate was filtered under vacuum washed with more water and hexane and finally dried under reduced pressure affording pure **2d** as a yellow solid (341mg, 0.23 mmol, 72% yield) which was used without further purification.

^{1}H NMR (600 MHz, $CDCl_3$) δ 9.51 (s, 4H), 8.46 (dd, J = 8.7, 2.9 Hz, 4H), 8.04 (dd, J = 8.7, 2.2 Hz, 4H), 6.43 (ddd, J = 12.4, 8.8, 2.3 Hz, 4H), 5.07 (dd, J = 8.8, 2.3 Hz, 4H). *Data consistent with literature.*[62]

General procedure for the preparation of Heteroleptic Iridium Complexes PC 1-7 (GP-1)

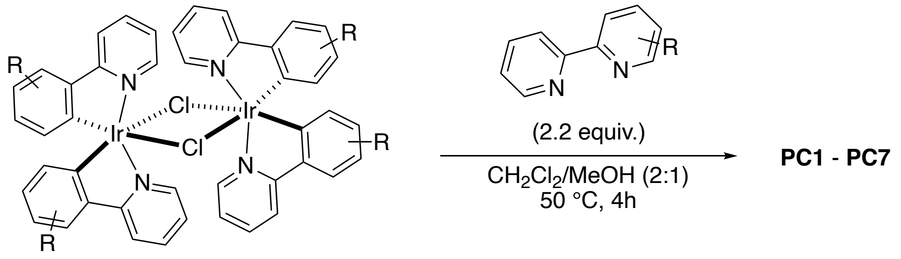

2a, R = H
2b, R = -CH_2-Naph
2c, R = -CH_2-Naph
2d, 2-(2,4-difluorophenyl)-5-(trifluoromethyl)pyridine

Heteroleptic iridium complexes **PC 1-7** and [Ir(ppy)$_2$(bpy)]BF_4 were synthetized adapting a literature procedure by Daniellou.[69]

A vial equipped with a stirring bar was charged under Nitrogen atmosphere with iridium dimer **2a-d** (1 equiv.) and the desired bidentate **L$_{N^N}$** ligand (2.2 equiv.). A degassed solution of CH_2Cl_2 and MeOH (0.005 M, 2/1 v/v) was then added, and the resulting solution was stirred at 50 °C for 4 hours. After cooling the solution to room temperature, NH_4BF_4 (20 equiv.) was added. The mixture was stirred for further 20 minutes. Solvents were removed under reduced pressure and the crude product was purified by chromatography on silica gel (CH_2Cl_2:MeOH 30:1). When necessary, additional purification was carried out through preparative TLC (CH_2Cl_2:MeOH 20:1) to afford pure **PC1-7**.

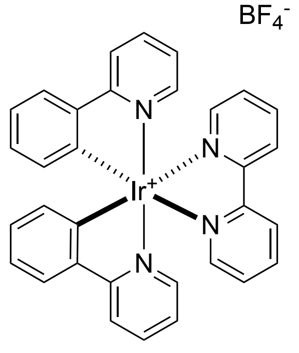

[Ir(ppy)$_2$(bpy)]BF$_4$

Complex [Ir(ppy)$_2$(bpy)]BF$_4$ was prepared according to procedure **GP1** from the corresponding iridium bridged dimer precursor **2a**. Orange crystalline solid (75 mg, 80% yield). **^{1}H NMR** (600 MHz, *Acetone-d$_6$*) δ 8.86 (dd, *J* = 8.2, 1.0 Hz, 2H), 8.29 (d, *J* = 17.5 Hz, 2H), 8.25 – 8.22 (m, 2H), 8.09 (ddd, *J* = 5.4, 1.6, 0.8 Hz, 2H), 7.95 (ddd, *J* = 8.2, 7.4, 1.5 Hz, 2H), 7.89 (dd, *J* = 7.8, 1.3 Hz, 2H), 7.83 (ddd, *J* = 5.8, 1.6, 0.8 Hz, 2H), 7.69 (ddd, *J* = 7.6, 5.5, 1.2 Hz, 2H), 7.16 (ddd, *J* = 7.4, 5.9, 1.4 Hz, 2H), 7.03 (d, *J* = 16.3 Hz, 2H), 6.91 (td, *J* = 7.4, 1.4 Hz, 2H), 6.47 – 6.07 (m, 2H). *Data consistent with literature.*[70]

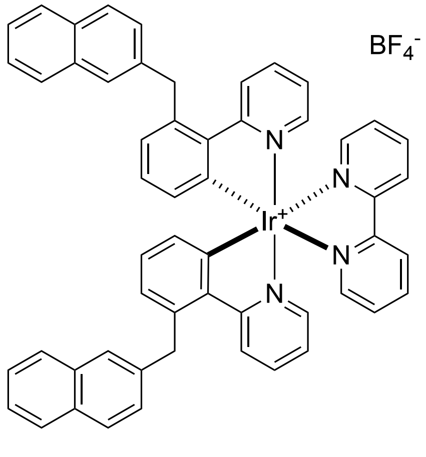

[Ir(2-(2-(naphthalen-2-ylmethyl)phenyl)pyridine)$_2$(bpy)]BF$_4$, PC1

Following **GP-1 PC1** was afforded in 40% yield over two steps. (20.1 mg, 0.019 mmol)
^{1}H NMR (400 MHz, *Acetone-d$_6$*) δ 8.81 (dt, J = 8.3, 1.1 Hz, 2H), 8.27 (td, J = 8.0, 1.7 Hz, 2H), 8.22 (d, J = 8.4 Hz, 2H), 8.14 (dd, J = 5.6, 1.4 Hz, 2H), 7.91 – 7.81 (m, 6H), 7.79 – 7.67 (m, 6H), 7.56 (bs, 2H), 7.48 – 7.42 (m, 6H), 7.03 – 6.98 (m, 4H), 6.92 (t, J = 7.5 Hz, 2H), 6.26 (dd, J = 7.6, 1.3 Hz, 2H), 4.82 (d, J = 17.2 Hz, 2H), 4.72 (d, J = 17.2 Hz, 2H). **^{13}C NMR** (101 MHz, *Acetone-d$_6$*) δ 167.8, 156.7, 154.6, 151.2, 150.5, 143.7, 140.6, 139.4, 138.9, 138.7, 134.6, 133.2, 131.4, 130.7, 129.4, 129.1, 128.9, 128.5, 128.4, 128.1, 127.4, 127.0, 126.4, 125.8, 125.1, 124.1, 42.4. **ESI-HRMS** calcd for $C_{54}H_{40}IrN_4^+$ [M-BF$_4$]$^+$ 937.2877, found 937.2886.

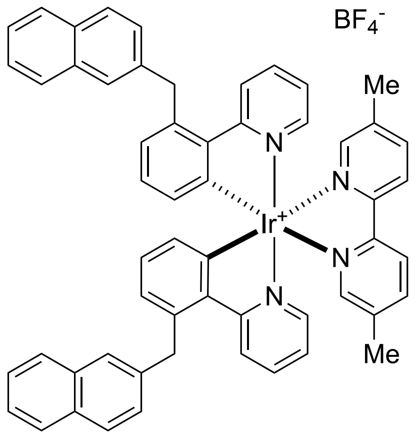

[Ir(2-(2-(naphthalen-2-ylmethyl)phenyl)pyridine)$_2$(5,5'-Dimethyl-bpy)]BF$_4$, PC2

Following **GP-1 PC2** was afforded in 29% yield over two steps. (16.0 mg, 0.015 mmol)
^{1}H NMR (400 MHz, *Acetone-d$_6$*) δ 8.64 (d, J = 8.3 Hz, 2H), 8.23 (d, J = 8.3 Hz, 2H), 8.06 (ddd, J = 8.3, 2.1, 0.8 Hz, 2H), 7.93 – 7.83 (m, 8H), 7.76 – 7.69 (m, 4H), 7.63 (bs, 2H), 7.49 – 7.41 (m, 6H), 7.03 – 6.98 (m, 4H), 6.92 (t, J = 7.5 Hz, 2H), 6.26 (dd, J = 7.5, 1.4 Hz, 2H), 4.82 (d, J = 17.1 Hz, 2H), 4.72 (d, J = 17.1 Hz, 2H), 2.31 (s, 6H). **^{13}C NMR** (101 MHz, *Acetone-d$_6$*) δ 167.8, 154.7, 154.4, 151.0, 150.5, 143.7, 140.9, 139.7, 139.3, 138.9, 138.7, 134.6, 133.2, 131.3, 130.6, 129.1, 128.8, 128.5, 128.4, 127.9, 127.5, 127.0, 126.4, 125.0, 124.9, 124.0, 42.3, 18.7. **ESI-HRMS** calcd for $C_{56}H_{44}IrN_4^+$ [M-BF$_4$]$^+$ 965.3190, found 965.3196.

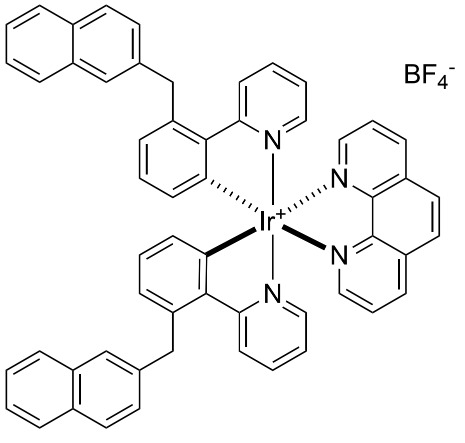

Ir(2-(2-(naphthalen-2-ylmethyl)phenyl)pyridine)$_2$(phen)]BF$_4$, PC3

Following **GP-1 PC3** was afforded in 34% yield over two steps. (17.8 mg, 0.017 mmol)
^{1}H NMR (400 MHz, *Acetone-d$_6$*) δ 8.88 (dd, J = 8.3, 1.5 Hz, 2H), 8.47 (dd, J = 5.0, 1.4 Hz, 2H), 8.36 (s, 2H), 8.21 (d, J = 8.4 Hz, 2H), 8.11 (dd, J = 8.3, 5.0 Hz, 2H), 7.89 (d, J = 8.5 Hz, 2H), 7.87 – 7.83 (m, 2H), 7.77 – 7.74 (m, 2H), 7.72 (dd, J = 5.8, 1.0 Hz, 2H), 7.65 (ddd, J = 8.6, 7.4, 1.7 Hz, 2H), 7.60 (bs, 2H), 7.50 – 7.41 (m, 6H), 7.04 (dd, J = 7.5, 1.4 Hz, 2H), 6.96 (t, J = 7.5 Hz, 2H), 6.82 (ddd, J = 7.3, 5.8, 1.3 Hz, 2H), 6.36 (dd, J = 7.5, 1.3 Hz, 2H), 4.84 (d, J = 17.2 Hz, 2H), 4.73 (d, J = 17.2 Hz, 2H). **^{13}C NMR** (101 MHz, *Acetone-d$_6$*) δ 167.8, 154.1, 151.8, 150.7, 147.6, 143.9, 139.7, 139.4, 138.8, 138.7, 134.6, 133.2, 132.6, 131.5, 130.6, 129.3, 129.1, 128.9, 128.5, 128.4, 128.1, 127.8, 127.4, 127.0, 126.4, 125.0, 123.9, 42.4. **ESI-HRMS** calcd for $C_{56}H_{40}IrN_4^+$ [M-BF$_4$]$^+$ 961.2877, found 961.2869.

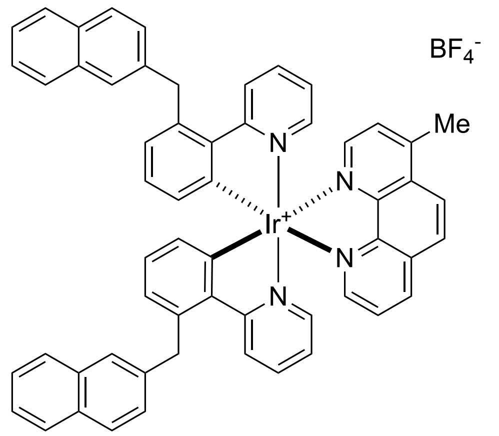

[Ir(2-(2-(naphthalen-2-ylmethyl)phenyl)pyridine)$_2$(4-Methyl-phen)]BF$_4$, PC4

Following **GP-1 PC4** was afforded in 25% yield over two steps. (17.2 mg, 0.016 mmol)
^{1}H NMR (400 MHz, *Acetone-d$_6$*) δ 8.87 (dd, J = 8.3, 1.5 Hz, 1H), 8.50 – 8.44 (m, 2H), 8.38 (d, J = 9.2 Hz, 1H), 8.29 (d, J = 5.2 Hz, 1H), 8.22 (dd, J = 8.6, 3.9 Hz, 2H), 8.09 (dd, J = 8.3, 5.1 Hz, 1H), 7.93 (dd, J = 5.2, 1.0 Hz, 1H), 7.90 (s, 1H), 7.86 (dd, J = 11.2, 3.7 Hz, 3H), 7.76 (dd, J = 7.1, 2.9 Hz, 2H), 7.71 (tt, J = 5.8, 1.0 Hz, 2H), 7.65 (dddd, J = 8.7, 7.4, 3.5, 1.7 Hz, 2H), 7.59 (bs, 2H), 7.52 – 7.40 (m, 6H), 7.03 (dt, J = 7.6, 1.6 Hz, 2H), 6.95 (t, J = 7.5 Hz, 2H), 6.82 (dddd, J = 7.2, 6.0, 5.0, 1.3 Hz, 2H), 6.36 (td, J = 7.5, 1.3 Hz, 2H), 4.85 (dd, J = 17.2, 2.9 Hz, 2H), 4.73 (dd, J = 17.1, 3.3 Hz, 2H), 2.98 (s, 3H). **^{13}C NMR** (101 MHz, *Acetone-d$_6$*) δ 167.9, 167.8, 154.4, 154.4, 151.9, 151.1, 150.6, 150.5, 150.1, 147.7, 147.2, 143.9, 143.9, 139.6, 139.4, 139.4, 138.8, 138.7, 138.7, 134.6, 134.6, 133.2, 132.4, 132.2, 131.6, 131.5, 130.6, 130.6, 129.1, 129.0, 128.9, 128.5, 128.4, 128.4, 128.4, 128.2, 128.2, 127.7, 127.4, 127.0, 126.4, 126.4, 125.9, 125.8, 125.0, 123.9, 123.9, 42.5, 19.1. **ESI-HRMS** calcd for $C_{57}H_{42}IrN_4^+$ [M-BF$_4$]$^+$ 975.3033, found 975.3057.

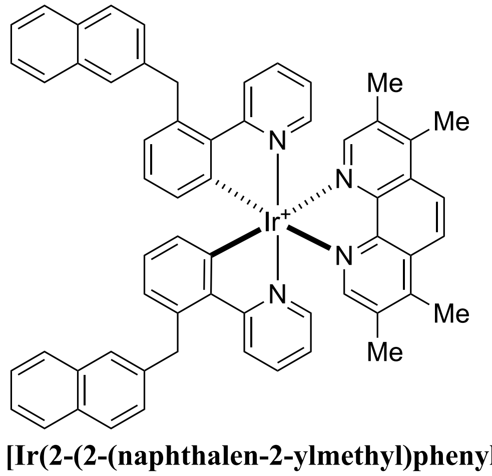

[Ir(2-(2-(naphthalen-2-ylmethyl)phenyl)pyridine)$_2$(3,4,7,8-Tetramethyl-phen)]BF$_4$, PC5

Following **GP-1 PC5** was afforded in 43% yield over two steps. (24.0 mg, 0.022 mmol)
^{1}H NMR (400 MHz, *Acetone-d$_6$*) δ 8.46 (s, 2H), 8.22 (d, J = 8.5 Hz, 2H), 8.13 (s, 2H), 7.90 – 7.84 (m, 4H), 7.76 – 7.70 (m, 4H), 7.68 – 7.63 (m, 4H), 7.48 – 7.40 (m, 6H), 7.02 (dd, J = 7.5, 1.4 Hz, 2H), 6.94 (t, J = 7.5 Hz, 2H), 6.82 (ddd, J = 7.3, 5.7, 1.3 Hz, 2H), 6.36 (dd, J = 7.5, 1.4 Hz, 2H), 4.84 (d, J = 17.0 Hz, 2H), 4.71 (d, J = 17.1 Hz, 2H), 2.84 (s, 6H), 2.45 (s, 6H). **^{13}C NMR** (101 MHz, *Acetone-d$_6$*) δ 167.9, 154.9, 151.9, 150.5, 147.4, 146.2, 143.8, 139.2, 138.7, 138.7, 136.1, 134.6, 133.2, 131.6, 130.9, 130.6, 129.1, 128.8, 128.5, 128.4, 127.9, 127.5, 127.0, 126.4, 125.3, 124.9, 123.9, 42.4, 18.2, 15.1. **ESI-HRMS** calcd for $C_{60}H_{48}IrN_4^+$ [M-BF$_4$]$^+$ 1017.3503, found 1017.3493.

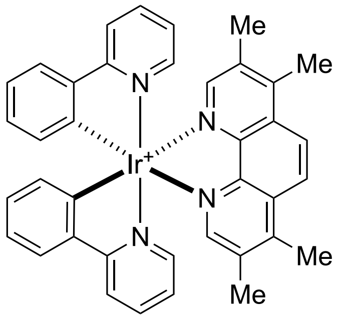

[Ir(dF(CF$_3$)ppy)$_2$[3,4,7,8-tetramethyl-1,10-phenanthroline]BF$_4$, PC6

Complex **PC6** was prepared according to the general procedure **GP1** from the corresponding iridium bridged dimer precursor **2b**. Yellow crystalline solid (51 mg, 75% yield). ^{1}**H NMR** (600 MHz, *Acetone-d$_6$*) δ 8.43 (s, 2H), 8.20 (dt, *J* = 8.2, 1.1 Hz, 2H), 8.12 (s, 2H), 7.90 (dd, *J* = 7.8, 1.3 Hz, 2H), 7.88 – 7.80 (m, 2H), 7.76 – 7.64 (m, 2H), 7.06 – 6.94 (m, 4H), 6.92 (td, *J* = 7.4, 1.3 Hz, 2H), 6.46 (dd, *J* = 7.6, 1.2 Hz, 2H), 2.81 (s, 6H), 2.35 (s, 6H). ^{13}**CNMR** (151 MHz, *Acetone-d$_6$*) δ 168.0 (2C), 151.3 (2C), 150.9 (2C), 149.3 (2C), 146.6 (2C), 145.6 (2C), 144.4 (2C), 138.5 (2C), 135.4 (2C), 131.9 (2C), 130.3 (2C), 130.1 (2C), 124.9 (2C), 124.6 (2C), 123.5 (2C), 122.4 (2C), 119.9 (2C), 17.22 (2C), 14.31 (2C). **ESI-HRMS** calcd for $C_{38}H_{32}IrN_4^+$ [M-BF$_4$]$^+$ 737.2251, found 737.2258.

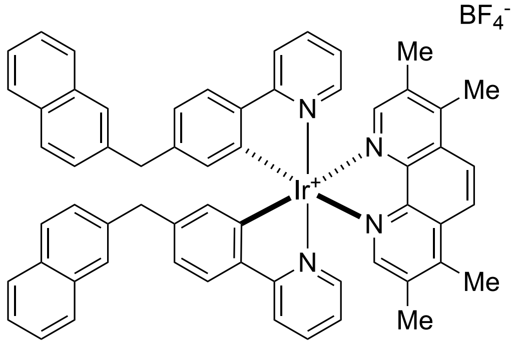

[Ir(2-(4-(naphthalen-2-ylmethyl)phenyl)pyridine)$_2$(3,4,7,8-Tetramethyl-phen)]BF$_4$, PC7

Following **GP-1 PC7** was afforded in 39% yield over two steps. (22.5 mg, 0.020 mmol)
^{1}**H NMR** (400 MHz, *Acetone-d$_6$*) δ 8.44 (s, 1H), 8.03 (s, 1H), 7.94 – 7.89 (m, 1H), 7.87 – 7.81 (m, 1H), 7.74 (d, J = 7.8 Hz, 3H), 7.53 – 7.44 (m, 4H), 7.40 – 7.37 (m, 1H), 7.20 (dd, J = 8.4, 1.8 Hz, 1H), 6.99 (dd, J = 8.0, 1.8 Hz, 1H), 6.56 (ddd, J = 7.4, 5.9, 1.4 Hz, 1H), 6.22 (d, J = 1.7 Hz, 1H), 4.00 (d, J = 15.1 Hz, 1H), 3.92 (d, J = 15.2 Hz, 1H), 2.82 (s, 3H), 2.31 (s, 3H). ^{13}**C NMR** (101 MHz, *Acetone-d$_6$*) δ 168.6, 152.1, 152.1, 149.5, 147.3, 146.4, 144.5, 143.1, 139.8, 138.8, 136.1, 134.6, 133.09, 133.06, 130.8, 128.8, 128.5, 128.45, 128.43, 127.8, 126.8, 126.2, 125.7, 125.3, 124.1, 123.5, 120.2, 42.5, 18.0, 15.1. **ESI-HRMS** calcd for $C_{60}H_{48}IrN_4^+$ [M-BF$_4$]$^+$ 1017.3503, found 1017.3514.

General procedure for the preparation of Heteroleptic Iridium Complexes PC 8 (GP-2)

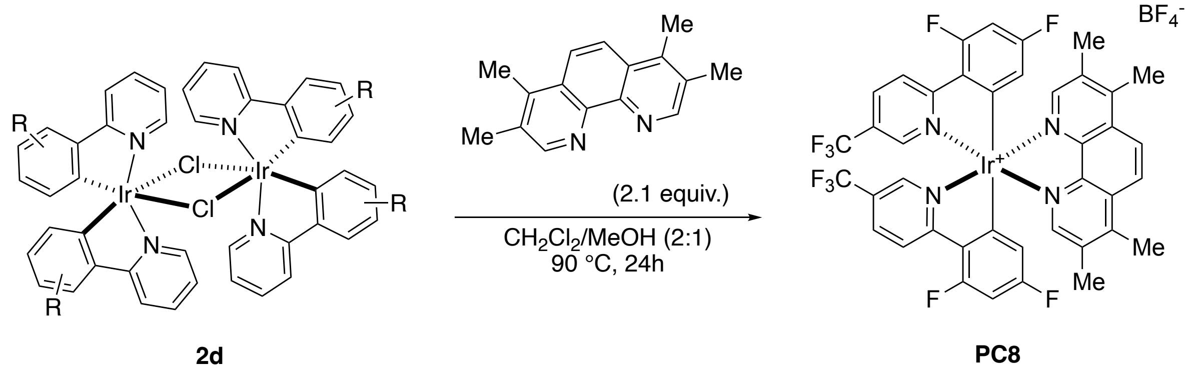

2d **PC8**

Heteroleptic iridium complex **PC8** was synthesized following a reported procedure.[71]

Iridium dimer **2d** (1 equiv.) and the bidentate N-N' ligand 3,4,7,8-tetramethyl-1,10-phenanthroline (2.1 equiv.) were placed in a sealed vial, equipped with a stirring bar, under Nitrogen atmosphere. A degassed solution of $CHCl_3$ and MeOH (2:1, 0.005M) was added and the solution was stirred at 90 °C for 24h. After cooling the solution to room temperarute, NH_4BF_4 was added, and the mixture was stirred for an addition hour. Solvents were removed under reduced pressure and the crude product was purified by chromatography on silica gel (CH_2Cl_2:MeOH 30:1 to 9:1) affording **PC8**. Yellow crystalline solid (70 mg, 86% yield).

^{1}H NMR (400 MHz, CD_2Cl_2) δ 8.56 (dd, J = 8.8, 3.1 Hz, 2H), 8.45 (s, 2H), 8.15 – 8.05 (m, 2H), 8.04 (s, 2H), 7.54 – 7.44 (m, 2H), 6.76 (ddd, J = 12.5, 9.1, 2.3 Hz, 2H), 5.86 (dd, J = 8.1, 2.3 Hz, 3H), 2.93 (s, 6H), 2.51 (s, 6H). **^{13}C NMR** (101 MHz, CD_2Cl_2) δ 168.1 (d, J = 7.3 Hz), 166.2 (d, J = 12.6 Hz), 164.0 (d, J = 12.9 Hz), 163.6 (d, J = 12.7 Hz), 161.4 (d, J = 13.2 Hz), 154.4 (d, J = 7.1 Hz), 151.3, 148.4, 145.0 (d, J = 4.8 Hz), 144.8, 136.4 (d, J = 37.6 Hz), 130.5, 126.6, 125.8, 125.5, 124.9, 123.9 (d, J = 21.0 Hz), 123.0, 120.3, 114.5 (dd, J = 17.8, 3.1 Hz), 99.9 (t, J = 26.8 Hz), 18.0, 15.2. **^{19}F NMR** (565 MHz, CD_2Cl_2) δ -63.2, -99.2 – -104.1 (m), -106.2 (td, J = 12.4, 3.3 Hz), -153.2. **ESI-HRMS** calcd for $C_{40}H_{26}F_{10}IrN_4^+$ [M-BF4]+ 945.1621, found 945.1630.

General procedure for the preparation of Heteroleptic Iridium Complexes PC 9-10 (GP-3)

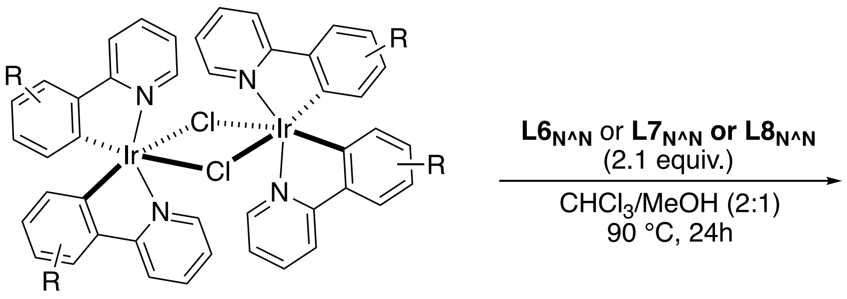

PC9 - PC11

2d

Iridium dimer **2d** (1 equiv.) and the desired bidentate N-N' ligand **L6**$_{N^N}$, **L7**$_{N^N}$ or **L8**$_{N^N}$ (2.1 equiv.) were placed in a sealed vial, equipped with a stirring bar, under Nitrogen atmosphere. A degassed solution of $CHCl_3$ and MeOH (2:1, 0.005M) was added and the solution was stirred at 90 °C for 24 hours. After cooling the solution to room temperature, $AgBF_4$ was added and the mixture was further stirred for one hour. Solvents were removed under reduced pressure and the crude product was purified by chromatography on silica gel (CH_2Cl_2:MeOH 30:1) and, when necessary, by an additional preparative TLC (CH_2Cl_2:MeOH 20:1), affording pure **PC9-PC11**.

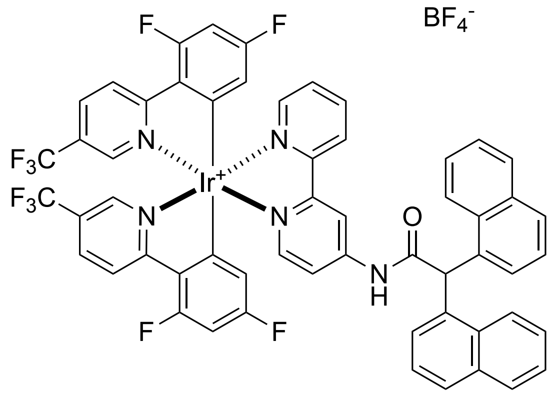

[Ir(dF(CF3)ppy)2[N-([2,2'-bipyridin]-4-yl)-2,2-di(naphthalen-1-yl)acetamide]BF4, PC9

Complex **PC9** was prepared according to the procedure **GP3** from the corresponding iridium bridged dimer precursor **2d**. Yellow crystalline solid (45 mg, 56% yield).

^{1}H NMR (400 MHz, $CDCl_3$) δ 10.42 (s, 1H), 8.85 (d, *J* = 8.2 Hz, 1H), 8.76 (d, *J* = 2.3 Hz, 1H), 8.54 (dd, *J* = 6.4, 2.2 Hz, 1H), 8.47 (dt, *J* = 8.7, 3.2 Hz, 2H), 8.39 – 8.13 (m, 3H), 8.04 (dt, *J* = 8.8, 2.4 Hz, 2H), 7.94 – 7.73 (m, 6H), 7.66 (d, *J* = 6.4 Hz, 1H), 7.58 – 7.43 (m, 6H), 7.44 – 7.31 (m, 4H), 6.87 (s, 1H), 6.65 (dddd, *J* = 12.4, 8.8, 3.8, 2.3 Hz, 2H), 5.62 (ddd, *J* = 15.7, 8.0, 2.3 Hz, 2H). **^{13}C NMR** (101 MHz, $CDCl_3$) δ 173.4, 168.0, 166.2 (d, *J* = 11.7 Hz), 163.8 (d, *J* = 17.7 Hz), 161.4, 156.1, 155.1, 155.0, 154.0, 150.7, 150.0 (d, *J* = 19.3 Hz), 145.1, 144.5, 141.0, 136.6, 134.0 (d, *J* = 4.1 Hz), 133.7 (d, *J* = 7.5 Hz), 131.8, 128.60 (d, *J* = 20.0 Hz), 127.0 (d, *J* = 38.7 Hz), 126.1, 126.0, 125.9, 125.2, 125.1, 123.9, 123.8, 123.6, 123.5, 122.8, 120.1, 117.8, 116.0, 114.1 (t, *J* = 19.0 Hz), 103.5 – 97.1 (m), 52.1. **^{19}F NMR** (565 MHz, $CDCl_3$) δ -62.4 -62.7, -101.0 (dt, *J* = 12.8, 8.5 Hz), -101.2 (dt, *J* = 12.8, 8.6 Hz), -105.5 (dt, *J* = 14.9, 7.7 Hz), -105.6 (td, *J* = 12.7, 3.5 Hz), -149.3 ($^{10}BF_4$), -149.3 ($^{11}BF_4$). **ESI-HRMS** calcd for $C_{56}H_{33}F_{10}IrN_5O^+$ $[M-BF_4]^+$ 1174.2149, found 1174.2154.

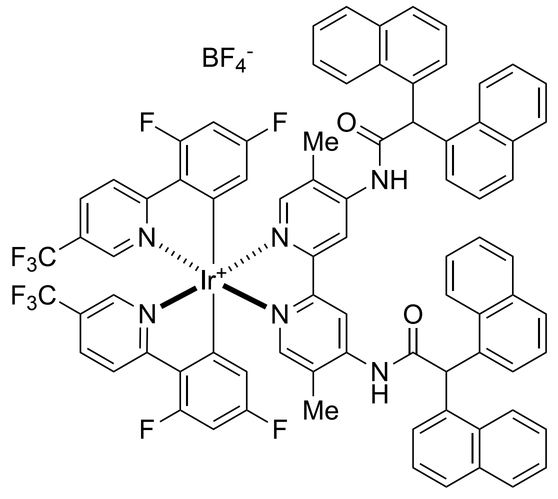

[Ir(dF(CF$_3$)ppy)$_2$[N,N'-(5,5'-dimethyl-[2,2'-bipyridine]-4,4'-diyl)bis(2,2-di(naphthalen-1-yl)acetamide)]BF$_4$, PC10

Complex **PC10** was prepared according to the procedure **GP3** from the corresponding iridium bridged dimer precursor **2d**. Yellow crystalline solid (54 mg, 68% yield).

^{1}H NMR (600 MHz, CDCl$_3$) δ 9.42 (s, 2H), 8.54 (s, 2H), 8.44 (dd, *J* = 8.9, 2.9 Hz, 2H), 8.15 – 8.08 (m, 2H), 8.03 (d, *J* = 8.3 Hz, 4H), 7.89 – 7.85 (m, 5H), 7.82 (t, *J* = 7.3 Hz, 5H), 7.58 (s, 2H), 7.53 – 7.33 (m, 14H), 7.28 (s, 1H), 6.85 (s, 2H), 6.59 (ddd, *J* = 11.4, 8.9, 2.3 Hz, 2H), 5.63 – 5.47 (m, 2H), 1.67 (s, 6H).**^{13}C NMR** (151 MHz, CDCl$_3$) 172., 168.22, 155.0 (d, *J* = 23.3 Hz), 150.6, 147.5, 145.3, 136.9, 134.4 (d, *J* = 8.5 Hz), 132.0, 129.3, 129.2, 128.3, 127.6 (d, *J* = 10.6 Hz), 127.2 (d, *J* = 20.8 Hz), 126.6, 125.9, 124.25, 123.8 (d, *J* = 15.0 Hz), 115.0, 114.3 (d, *J* = 17.3 Hz), 100.2, 53.5, 15.0.**^{19}F NMR** (565 MHz, CDCl$_3$) δ -62.4, -98.0 – -103.7 (m), -105.6 (t, *J* = 12.4 Hz), -151.4 (^{10}BF$_4$), -151.4 (^{11}BF$_4$). **ESI-HRMS** calcd for $C_{80}H_{52}F_{10}IrN_6O_2^+$ [M$^+$] 1511.3616, found 1511.3610.

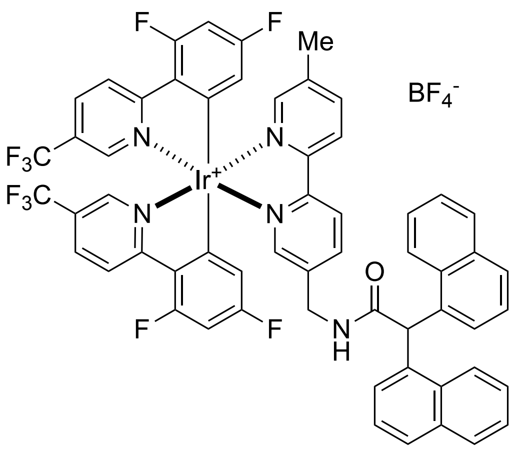

{Ir(dF(CF$_3$)ppy)$_2$[*N*-((5'-methyl-[2,2'-bipyridin]-5-yl)methyl)-2,2-di(naphthalen-1-yl)acetamide]}BF$_4$, PC11

Complex **PC11** prepared according to procedure **GP3** from the corresponding iridium bridged dimer **2d**. Yellow crystalline solid (75 mg, 71% yield).

^{1}H NMR (600 MHz, *Acetone-d6*) δ 8.75 (dd, *J* = 15.4, 8.4 Hz, 2H), 8.60 (dd, *J* = 8.8, 2.5 Hz, 1H), 8.45 (dd, *J* = 8.7, 2.6 Hz, 1H), 8.39 (dd, *J* = 8.9, 2.1 Hz, 1H), 8.30 – 8.23 (m, 3H), 8.21 (dd, *J* = 8.4, 2.0 Hz, 1H), 8.13 (dd, *J* = 8.3, 2.0 Hz, 1H), 8.10 (d, *J* = 2.0 Hz, 1H), 8.04 (d, *J* = 8.5 Hz, 1H), 8.01 – 7.91 (m, 5H), 7.85 (dd, *J* = 17.3, 8.2 Hz, 2H), 7.51 (dtd, *J* = 7.7, 3.8, 1.9 Hz, 2H), 7.47 (ddd, *J* = 8.3, 6.7, 1.4 Hz, 1H), 7.43 (ddd, *J* = 8.3, 6.8, 1.3 Hz, 1H), 7.39 (t, *J* = 7.7 Hz, 1H), 7.27 (t, *J* = 7.7 Hz, 1H), 7.20 (d, *J* = 7.2 Hz, 1H), 7.00 (d, *J* = 7.2 Hz, 1H), 6.83 (ddd, *J* = 12.1, 9.3, 2.3 Hz, 1H), 6.75 (ddd, *J* = 12.1, 9.3, 2.4 Hz, 1H), 6.51 (s, 1H), 5.92 (ddd, *J* = 17.0, 8.4, 2.3 Hz, 2H), 4.45 – 4.36 (m, 2H), 2.31 (s, 3H). **^{13}CNMR** (151 MHz, *Acetone-d$_6$*) δ 172.9, 155.5, 154.2, 152.2, 151.9, 147.0, 141.9, 141.8, 141.0, 140.2, 138.1, 136.4 (d, *J* = 1.7 Hz), 135.1, 132.8, 129.8, 128.8, 128.7, 127.6, 127.5, 127.4, 127.4, 126.7, 126.6, 126.2 (d, *J* = 2.1 Hz), 125.7, 125.5, 124.9, 124.8, 124.2, 115.5, 100.2 (d, *J* = 16.3 Hz), 51.7, 41.3, 18.5. **^{19}FNMR** (565 MHz, *Acetone-d$_6$*) δ -63.2, -63.3, -104.6 (dt, *J* = 12.1, 8.9 Hz), -104.7 (dt, *J* = 11.9, 8.9 Hz), -107.4 (td, *J* = 12.3, 3.0 Hz), -107.9 (td, *J* = 12.2, 2.9 Hz), -151.5 ($^{10}BF_4$), -151.6 ($^{11}BF_4$). **ESI-MS** calcd for $C_{58}H_{37}F_{10}IrN_5O^+$ $[M-BF_4]^+$ 1202.24, found 1202.21.

Procedures for the preparation of substrates

Preparation of C

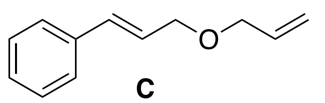

C

Following the procedure reported at the reference [72] of the manuscript, **C** was obtained in 87% yield. Spectroscopic data are consistent with literature. (421 mg, 2.42 mmol).

^{1}H NMR (400 MHz, $CDCl_3$) δ 7.42 – 7.37 (m, 2H), 7.35 – 7.29 (m, 2H), 7.25 – 7.21 (m, 1H), 6.62 (dt, J = 15.9, 1.6 Hz, 1H), 6.31 (dt, J = 15.9, 6.0 Hz, 1H), 5.96 (ddt, J = 17.2, 10.4, 5.6 Hz, 1H), 5.32 (dd, J = 17.2, 1.7 Hz, 1H), 5.22 (dd, J = 10.4, 1.5 Hz, 1H), 4.17 (dd, J = 6.1, 1.5 Hz, 2H), 4.05 (dt, J = 5.6, 1.4 Hz, 2H).

Preparation of E

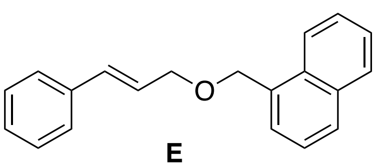

E

Following the procedure reported at the reference [41] of the manuscript, **E** was obtained in 76% yield. Spectroscopic data are consistent with literature. (450 mg, 1.64 mmol)

^{1}H NMR (400 MHz, $CDCl_3$) δ 8.18 (dd, *J* = 8.4, 1.2 Hz, 1H), 7.94 – 7.81 (m, 2H), 7.61 – 7.50 (m, 3H), 7.47 (dd, *J* = 8.2, 6.9 Hz, 1H), 7.44 – 7.40 (m, 2H), 7.34 (t, *J* = 7.4 Hz, 2H), 7.29 – 7.23 (m, 1H), 6.68 (d, *J* = 15.9 Hz, 1H), 6.39 (dt, *J* = 15.9, 6.0 Hz, 1H), 5.05 (s, 2H), 4.29 (dd, *J* = 6.1, 1.5 Hz, 2H). **^{13}C NMR** (101 MHz, $CDCl_3$) δ 137.0, 134.1, 134.0, 133.0, 132.1, 129.0, 128.9 (2C), 128.0, 126.8 (2C), 126.6, 126.4, 126.1, 125.6, 124.4, 71.2, 70.9. **ESI-MS** calcd for $C_{20}H_{18}NaO$ $[M+Na]^+$ 297.12, found 297.15.

Preparation of G

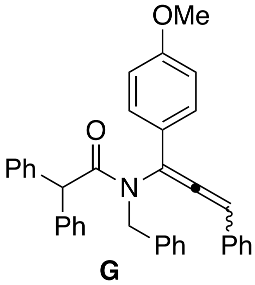

G

Following the procedure reported at the reference [41] of the manuscript, **G** was obtained in 91% yield as white solid. Spectroscopic data are consistent with literature. (490 mg, 0.9 mmol).

^{1}H NMR (400 MHz, $CDCl_3$) δ 7.51 – 7.04 (m, 20H), 6.97 – 6.85 (m, 2H), 6.70 (dt, *J* = 6.9, 1.5 Hz, 2H), 5.93 (s, 1H), 5.50 (s, 2H), 4.52 – 4.27 (m, 1H), 3.88 (s, 3H).

Screening of the catalytic activities of the Ir-complexes

Catalytic isomerization reaction

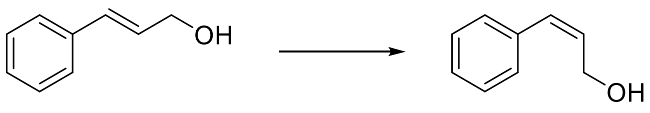

A B

To a vial charged with substrate **A** (0.1 mmol, 1 equiv.), an Iridium photosensitizer (1 mol%) and a dry and degassed mixture of MeCN/$CHCl_3$ (7:3, 0.1 M) were added through a syringe. Then, DIPEA (10 mol%) was added, and the solution was transferred into an NMR tube capped with a rubber septum and it was placed in an oil bath kept at 25 °C and irradiated with blue LEDs stripe (420-500 nm) or (380-420 nm) for 1 hour. The mixture was then concentrated in vacuo. The *Z/E* ratio were determined by **^{1}H NMR** spectra on the reaction crude. Spectroscopic data are consistent with literature.[41]

^{1}H NMR (400 MHz, $CDCl_3$) δ 7.36 – 7.17 (m, 5H), 6.58 (d, *J* = 11.8 Hz, 1H), 5.88 (dt, *J* = 11.7, 6.4 Hz, 1H), 4.45 (dd, *J* = 6.5, 1.7 Hz, 2H), 1.64 (s, 1H). **ESI-MS** calcd for $C_9H_{11}O$ $[M+H]^+$ 135.18, found 135.21.

Table S1: Evaluation of the photosensitizers in the isomerization reaction of A

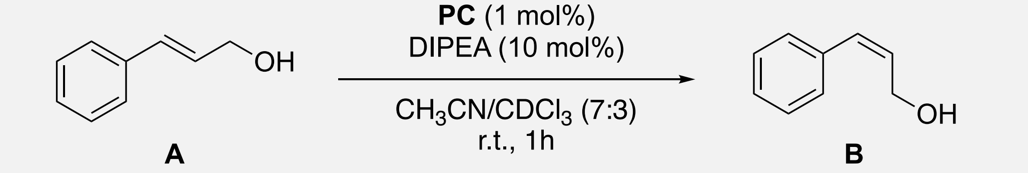

Entry[a]	PC	A	B
**1**	Ir(ppy)$_3$	64	36
**2**[b]	Ir(ppy)$_3$	24	76
**3**	[Ir(dF(CF$_3$)ppy)$_2$(dtbbpy)PF$_6$]	69	31
**4**[c]	[Ir(dF(CF$_3$)ppy)$_2$(bpy)PF$_6$]	77	23
**5**	[Ir(ppy)$_2$(bpy)PF$_6$]	94	6
**6**	**PC1**	93	7
**7**	**PC2**	89	11
**8**	**PC3**	97	3
**9**	**PC4**	91	9
**10**	**PC5**	29	71
**11**	**PC6**	31	69
**12**	**PC7**	40	60
**13**	**PC8**	80	20
**14**	**PC9**	54	46
**15**	**PC10**	56	44
**16**	**PC11**	80	20

[a]**Conditions**: **A** (0.1 mmol), DIPEA (10 mol%), **PC** (1 mol%) in MeCN/$CHCl_3$ (7:3, 0.1 M), irradiated with blue LEDs stripe (420-500 nm) for 1 hour at 25 °C in 5 mm NMR tube under N_2, A:B ratio was determined by ^{1}H NMR; [b]5 equiv. Naphtalene were added; [c]from ref. 41 of the main manuscript

Catalytic [2+2] reaction

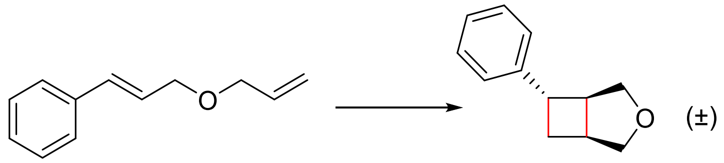

C D

To a vial charged with substrate **C** (0.1 mmol, 1 equiv.) and the desired Iridium photocatalyst (1 mol%), dry and degassed ACN (0.05 M) was added. The solution was transferred in a 5 mm-wide (external diameter) NMR tube and degassed via freeze-pump cycles (2x). The homogeneous solution was irradiated at room temperature with blue LEDs stripe (420-500 nm) or (380-420 nm) for 4 hours. The mixture was then concentrated and purified by chromatography on silica gel affording product **D** as a mixture of two diastereoisomers (*d:r*=86:14)

^{1}H NMR (400 MHz, $CDCl_3$) δ 7.37 – 7.31 (m, Dia1, 2H; Dia2, 2H), 7.30 – 7.26 (m, Dia1, 2H), 7.24 – 7.19 (m, Dia1, 1H; Dia2, 3H), 4.01 (dd, J = 9.3, 7.3 Hz, Dia1, 2H), 3.86 (d, J = 9.0 Hz, Dia2, 1H), 3.72 (dd, J = 9.8, 1.6 Hz, Dia2, 2H), 3.63 (dd, J = 9.3, 5.7 Hz, Dia1, 1H), 3.53 (dd, J = 9.3, 4.6 Hz, Dia1, 1H), 3.45 (dd, J = 9.0, 4.4 Hz, Dia2, 1H), 3.38 (dd, J = 10.0, 6.7 Hz, Dia2, 1H), 3.30 – 3.17 (m, Dia1, 1H; Dia2, 1H), 3.07 – 2.93 (m, Dia1, 2H; Dia2, 1H), 2.45 (dddd, J = 12.4, 10.4, 8.2, 2.4 Hz, Dia2, 1H), 2.32 (dt, J = 12.4, 8.1 Hz, Dia1, 1H), 2.23 – 2.15 (m, Dia1, 1H; Dia2, 1H). **^{13}C NMR** (101 MHz, $CDCl_3$, Dia1) δ 146.2, 128.5 (2C), 126.5 (2C), 126.0, 74.5, 74.1, 47.3, 42.0, 35.4, 31.9. **ESI-HRMS** calcd for $C_{12}H_{14}O$ $[M+H]^+$ 175.1045, found 175.1050

Table S2: Evaluation of the photosensitizers in the [2+2]-reaction of C

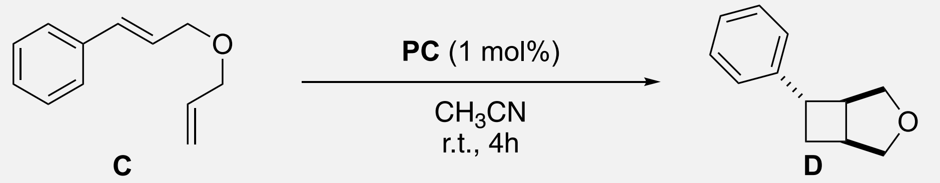

Entry[a]	PC	C	C'	D
**1**	Ir(ppy)$_3$	11	50	21
**2[b]**	Ir(ppy)$_3$	0	0	99
**2**	[Ir(dF(CF$_3$)ppy)$_2$(dtbbpy)PF$_6$]	2	4	65
**3**	[Ir(dF(CF$_3$)ppy)$_2$(bpy)PF$_6$]	16	35	49
**4**	[Ir(ppy)$_2$(bpy)PF$_6$]	91	0	0
**5**	**PC1**	92	traces	0
**6**	**PC2**	38	38	7
**7**	**PC3**	70	11	traces
**8**	**PC4**	28	42	5
**9[c,d]**	**PC5**	0	traces	86
**10**	**PC6**	0	0	72
**11**	**PC7**	2	4	72
**12**	**PC8**	10	21	49
**13**	**PC9**	7	25	52
**14**	**PC10**	0	0	63[b]
**16**	**PC11**	12	43	27

[a]**Conditions**: **C** (0.1 mmol), **PC** (1 mol%) in MeCN (0.05 M), irradiated with blue LEDs stripe (420-500 nm) for 4 hours at 25 °C in 5 mm NMR tube under N_2, C:C' ratio and d.r. were determined by ^{1}H NMR, d.r. 86:14; [b]20 equiv. of naphthalene were added; [c]Isolated yield; [d]from ref. 41 of the main manuscript

Catalytic [4+2] reaction

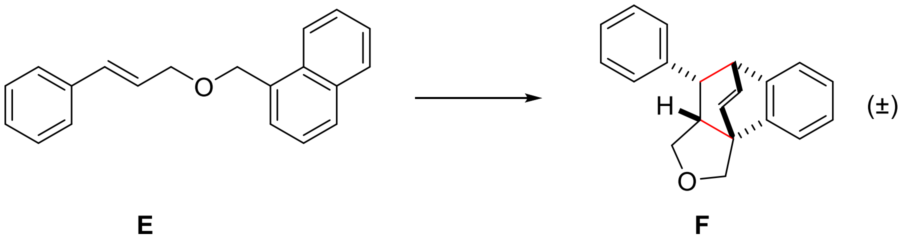

To a vial charged with substrate **E** (0.1 mmol, 1 equiv.) and the desired Iridium photocatalyst (1 mol%), dry and degassed CH_2Cl_2 (0.05 M) was added. The solution was transferred in a 5 mm-wide (external diameter) NMR tube and degassed via freeze-pump cycles (2x). The homogeneous solution was irradiated at room temperature with blue LEDs stripe (420-500 nm) for 18 hours. The mixture was then concentrated and purified by chromatography on silica gel affording product **F** as a mixture of two diastereoisomers (*d:r*=82:18)

^{1}H NMR (400 MHz, $CDCl_3$) δ 7.43 (m, Dia2, 1H), 7.33 (m, Dia2, 2H), 7.28 – 7.06 (m, Dia1, 7H; Dia2, 6H), 6.91 (dd, *J* = 7.6, 6.2 Hz, Dia1, 1H), 6.79 (dd, *J* = 7.7, 1.2 Hz, Dia2, 1H), 6.64 (m, Dia1, 2H; Dia2, 1H), 6.38 (d, *J* = 7.6 Hz, Dia1, 1H), 4.96 (d, *J* = 8.7 Hz, Dia2, 1H), 4.52 (s, Dia1, 2H), 4.16 – 4.03 (m, Dia1, 1H; Dia2, 2H), 3.93 (d, *J* = 6.1 Hz, Dia1, 1H), 3.36 (dd, *J* = 11.0, 7.4 Hz, Dia1, 1H), 2.88 – 2.81 (m, Dia1 1H; Dia2, 2H), 2.56 (dd, *J* = 6.9, 1.6 Hz, Dia2, 1H), 2.41 (dt, *J* = 11.3, 7.1 Hz, Dia2, 1H), 2.21 (dddd, *J* = 11.0, 7.5, 6.3, 1.1 Hz, Dia1, 1H). **^{13}C NMR** (101 MHz, $CDCl_3$, Dia1) δ 144.2, 143.4, 140.3, 138.7, 136.0, 128.2 (2C), 127.6 (2C), 126.5, 126.3, 125.6, 125.1, 118.7, 72.7, 70.1, 55.5, 54.7, 50.0, 47.2. **ESI-HRMS** calcd for $C_{20}H_{18}O$ $[M+H]^+$ 275.3650, found 275.3663.

Table S3: Evaluation of the photosensitizers in the dearomative [4+2]-cycloaddition reaction of E

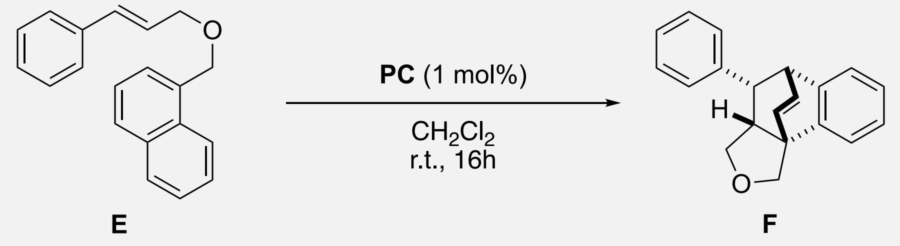

Entry[a]	PC	E	E’	F
**1**	$Ir(ppy)_3$	21	18	46
**2**[b,c]	$Ir(ppy)_3$	0	0	93
**3**	$[Ir(dF(CF_3)ppy)_2(dtbbpy)PF_6]$	14	9	64
**4**	$[Ir(dF(CF_3)ppy)_2(bpy)PF_6]$	21	14	60
**5**	$[Ir(ppy)_2(bpy)PF_6]$	78	7	0
**6**	**PC1**	63	18	Traces
**7**	**PC2**	31	26	26
**8**	**PC3**	52	31	Traces
**9**[c]	**PC4**	30	32	13
**10**[c]	**PC5**	0	traces	89
**11**[c]	**PC6**	0	0	81
**12**[c]	**PC7**	0	0	85
**13**	**PC8**	29	23	45
**14**	**PC9**	0	0	68
**15**	**PC10**	0	0	68
**16**	**PC11**	26	19	55

[a]**Conditions**: **E** (0.1 mmol), **PC** (1 mol%) in DCM (0.05 M), irradiated with blue LEDs stripe (420-500 nm) for 18 hours at 25 °C in 5 mm NMR tube under N_2, E:E’ ratio and d.r. were determined by ^1H NMR, d.r. 82:18; [b]20 equiv. of naphthalene were added; [c]Isolated yield

Catalytic [1,5]-HAT/cyclization on allenamide G

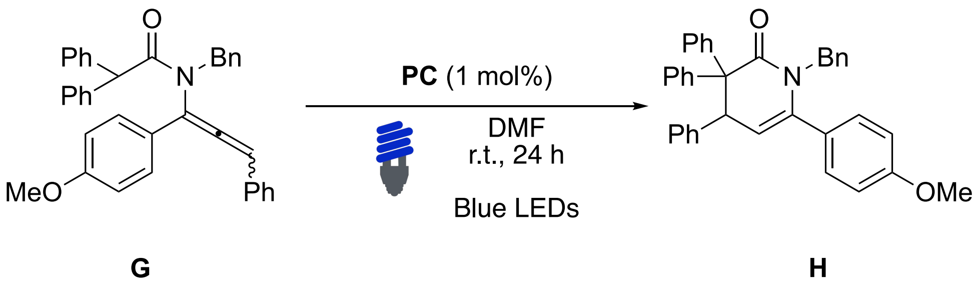

To a vial charged with substrate **G** (0.1 mmol, 1 equiv.) and the desired Iridium photocatalyst (1 mol%), dry degassed DMF (0.1 M) were added through a syringe. Then the solution was transferred into an NMR tube capped with a rubber septum and it was placed in an oil bath kept at 25 °C and irradiated with blue LED stripes (420-500 nm) for 24 hours. The mixture was then concentrated in vacuo and purified trough column chromatography (Hexane:EtOAc) to afford pure **H** as a white solid.

^{1}H NMR (600 MHz, $CDCl_3$) δ 7.61 – 7.54 (m, 2H), 7.33 (q, J = 6.6 Hz, 3H), 7.27 – 7.18 (m, 3H), 7.11 – 7.00 (m, 3H), 6.97 (t, J = 7.3 Hz, 2H), 6.91 (t, J = 7.6 Hz, 2H), 6.83 (t, J = 8.2 Hz, 3H), 6.76 (d, J = 8.3 Hz, 2H), 6.58 (d, J = 7.8 Hz, 2H), 6.45 (d, J = 7.5 Hz, 2H), 5.57 (d, J = 6.9 Hz, 1H), 5.18 (d, J = 14.5 Hz, 1H), 4.33 (d, J = 14.5 Hz, 1H), 4.10 (d, J = 7.0 Hz, 1H), 3.77 (s, 3H). *Data consistent with literature.*[41]

Table S4: Evaluation of the photosensitizers in Catalytic [1,5]-HAT/cyclization on allenamide G

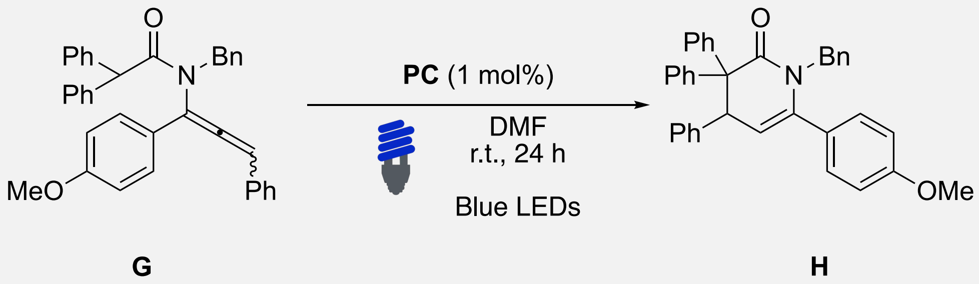

Entry[a]	PC	**G** (%yield)	**H** (%yield)
**1**	Ir(ppy)$_3$	0	26
**2**[b]	Ir(ppy)$_3$	0	86
**3**	**PC5**	0	43
**4**	**PC10**	0	54[c]
**5**	**PC11**	0	59

[a]**Conditions**: **G** (0.1 mmol), PC (1 mol%) in DMF (0.1 M), irradiated with blue LEDs stripe (420-500 nm) for 24 hours at 25 °C in 5 mm NMR tube under N_2, [b]20 equiv. of naphthalene were added; [c]Isolated yields.

Photophysical Characterizations of the complexes

PC	ε at 460 nm [M^{-1} cm^{-1}]	λ_{EM} [nm]	**Triplet energy** [Kcal/mol]	**Triplet lifetime** τ [μs]	**Quantum yield** in ACN	K_{sv} [M^{-1}]	**Kq** [$(M*s)^{-1}$]
$Ir(ppy)_3$	-	518	59.5	4.7	0.4	-	-
$[Ir(dFCF_3ppy)_2(bpy)PF_6]$	-	483	61.6	2.28	-	-	-
$[Ir(ppy)_2(bpy)PF_6]$	-	608	55.3	0.34	0.08	-	-
**PC1**	995.5	580	56.7	0.24	0.13	-	-
**PC2**	843.8	550	57.9	0.87	0.16	-	-
**PC3**	900.8	575	56.4	0.61	0.13	10	1.7E+7
**PC4**	626.8	565	57.2	0.82	0.20	-	-
**PC5**	919.5	525	61.3	1.83	0.14	564	3.1E+8
**PC6**	851.8	524	61.6	1.87	0.08	1776	9.5E+8
**PC7**	806.1	551	59.5	1.2	0.15	-	-
**PC8**	568.6	476	63.0	3.9	0.34	-	-
**PC9**	684.3	474	63.4	8.3	0.35	-	-
**PC10**	557.7	478	62.9	1.1<br>13.1	0.48	-	-
**PC11**	129.8	473	64.0	5.5<br>33.5	0.56	-	-

Absorption Spectra

UV−vis absorption spectra of were recorded on a V-550 Jasco Spectrophotometer with a resolution of 1 nm. Samples were prepared using degassed ACN, at a concentration of 10^{-5}M by dilution of a 100 μL of a 10^{-3} M solution of the complex in a 10 mL volumetric flask. The measures were performed with cells of optical path of 1 cm. UV−vis absorption spectra of **PC8-11** were recorded on a Spectrophotometer UV/Vis/NIR Lambda 750 with a resolution of 1 nm. Sample solutions were prepared using degassed CH_3CN, at a concentration of 10^{-5}M by dilution of a 100 μL of a 10^{-3}M solution of the complex in a 10 mL volumetric flask to reach a concentration of $1*10^{-5}$ M. The measures were performed with cells of optical path of 1 cm.

fac-Ir(ppy)$_3$ absorption spectrum

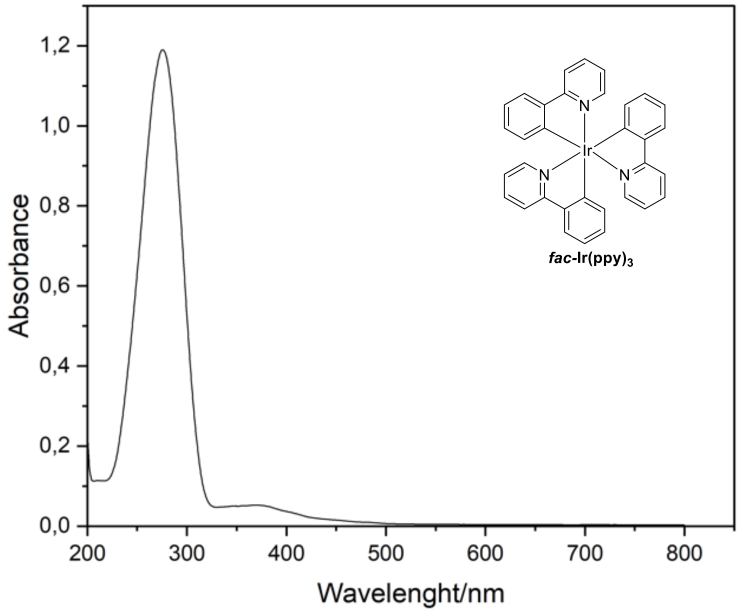

PC1 absorption spectrum

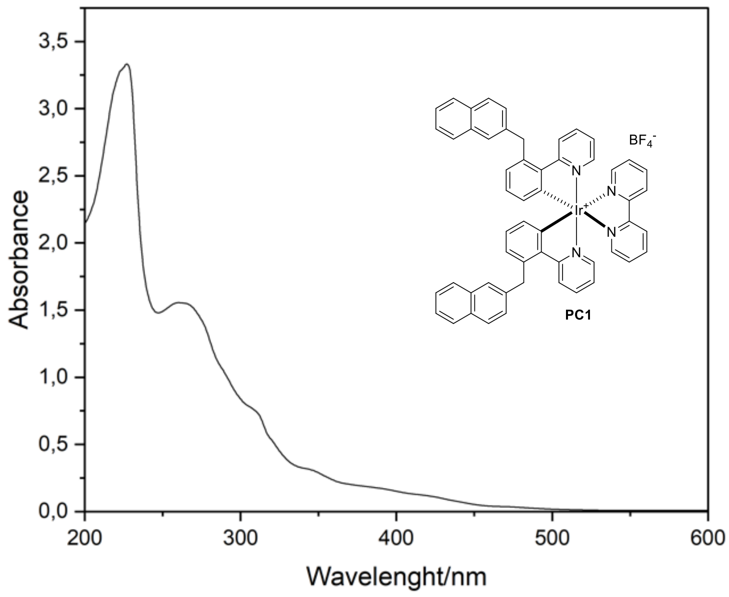

PC2 absorption spectrum

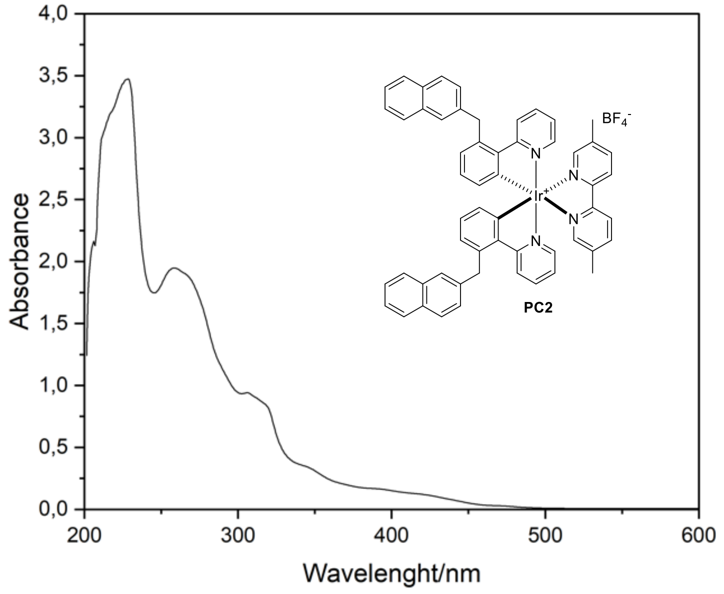

PC3 absorption spectrum

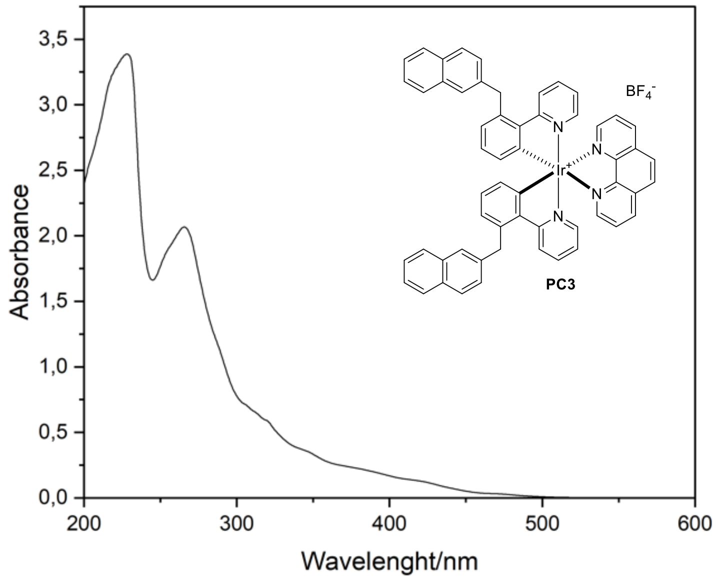

PC4 absorption spectrum

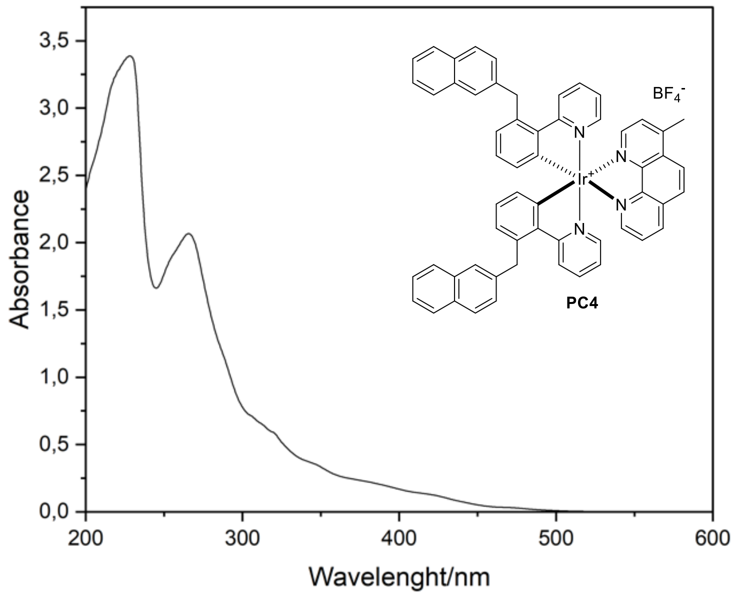

PC5 absorption spectrum

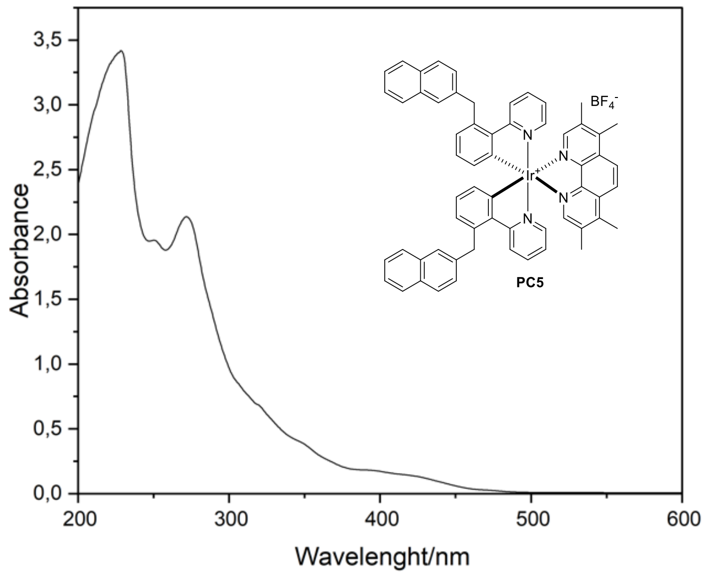

PC5 absorption spectrum 350-650 nm range

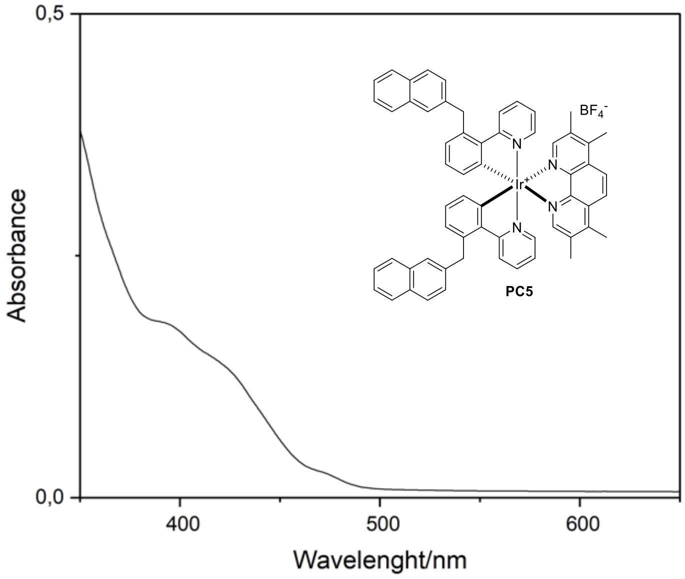

PC6 absorption spectrum

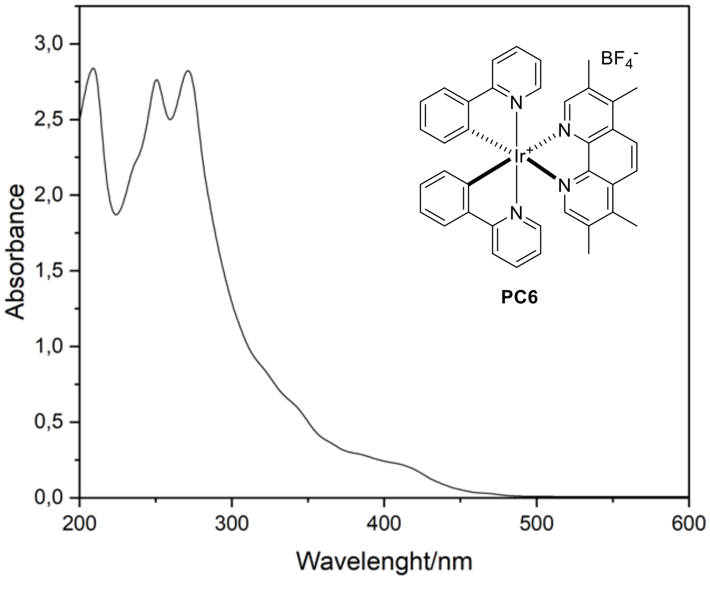

PC7 absorption spectrum

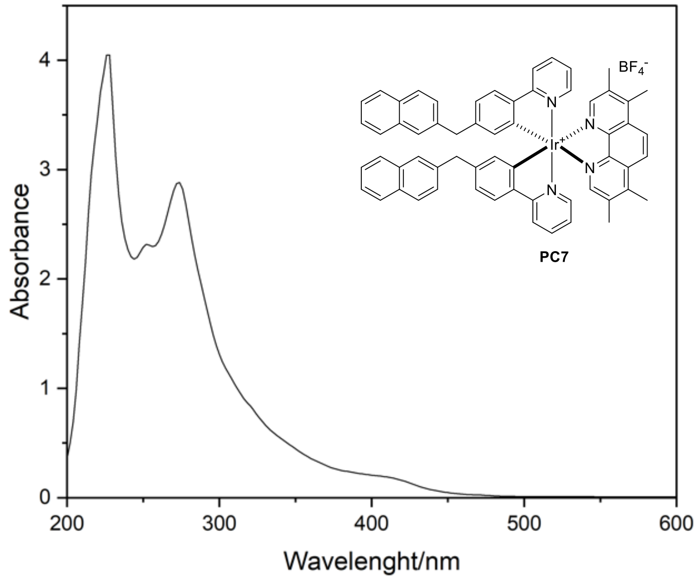

PC8 absorption spectrum

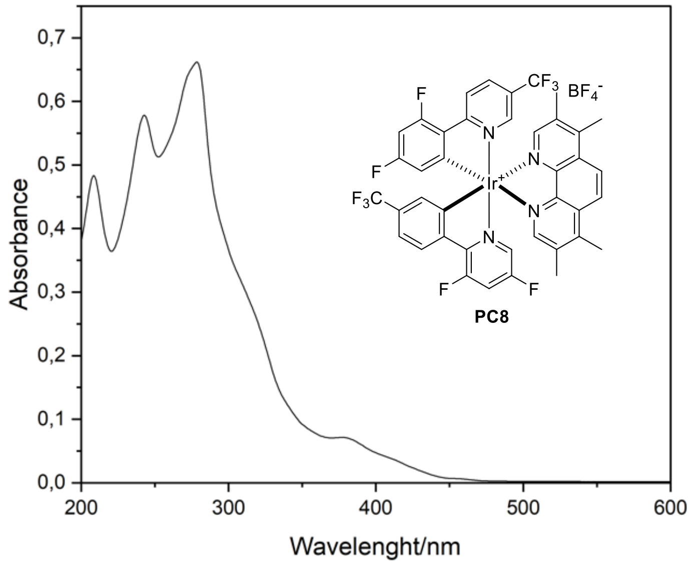

PC9 absorption spectrum 1x10^{-5}M

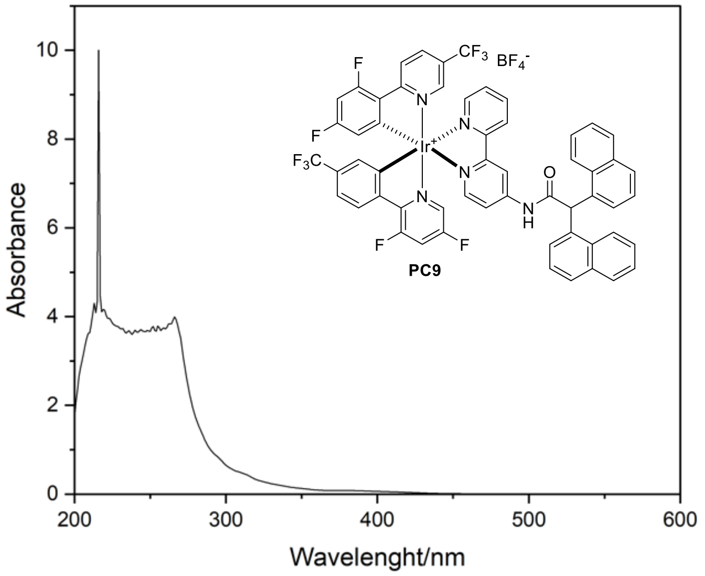

PC9 absorption spectrum 5x10^{-4}M (*reaction condition*)

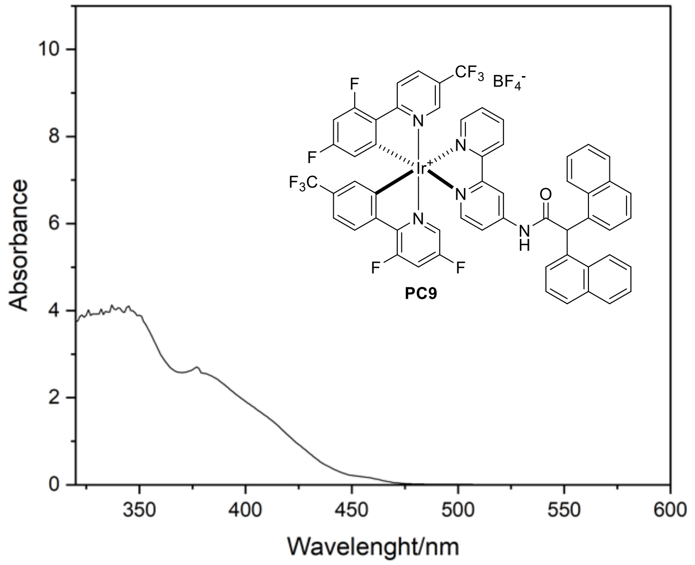

PC10 absorption spectrum 1x10^{-5}M

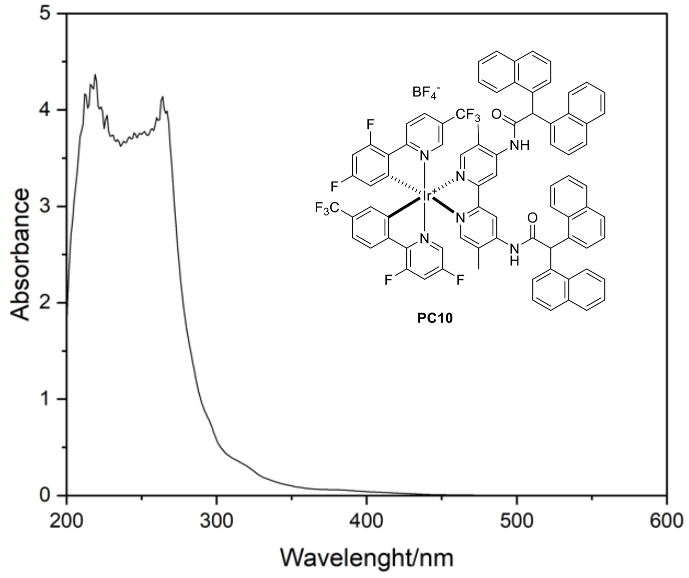

PC10 absorption spectrum 5x10^{-4}M (*reaction condition*).

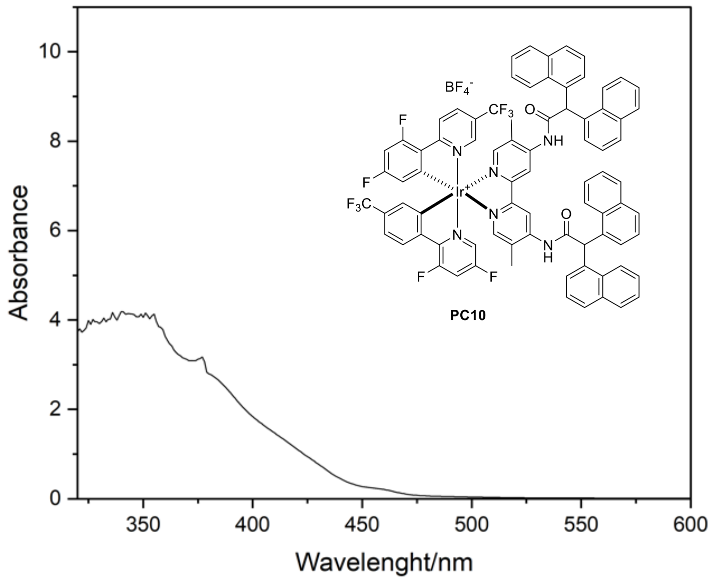

PC11 absorption spectrum 1x10^{-5}M

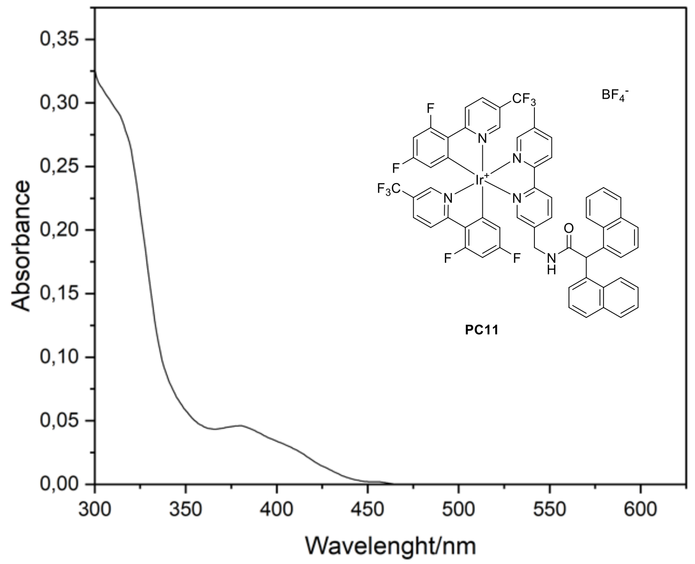

PC11 absorption spectrum 5x10^{-4}M (*reaction condition*)

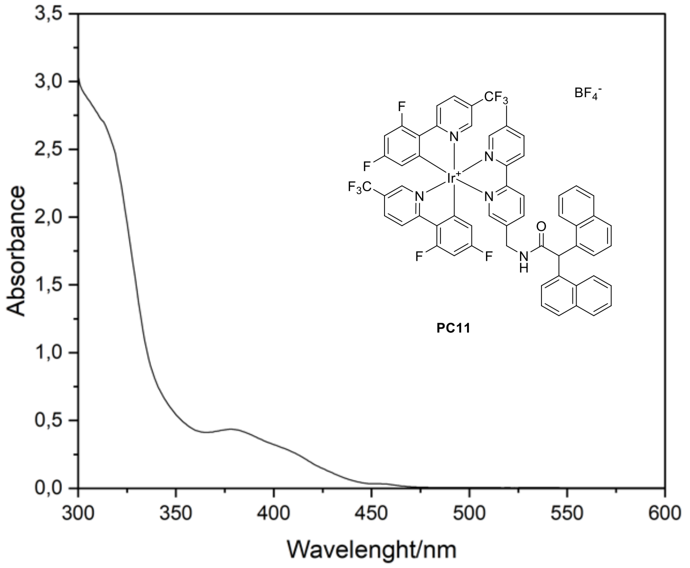

Emission Spectra

Phosphorescence Emission spectra were recorded on FLS 980 Edinburg (**PC1-7**) and on a FLS1000 Edinburgh Fluorometer (**PC8- PC11**), with a resolution of 1 nm.
Samples were prepared in dry degassed CH_3CN. The solutions were prepared at a concentration of 10^{-5} M by dilution of 100 µL of a 10^{-3}M solution of the complex in a 10 mL volumetric flask. A quartz cuvette (optical path = 1 cm) capped with a rubber septum was used for the acquisition. The samples solutions were deoxygenated by bubbling a stream of nitrogen for 10 minutes prior to the acquisition of the spectra.

fac-Ir(ppy)₃ emission spectrum

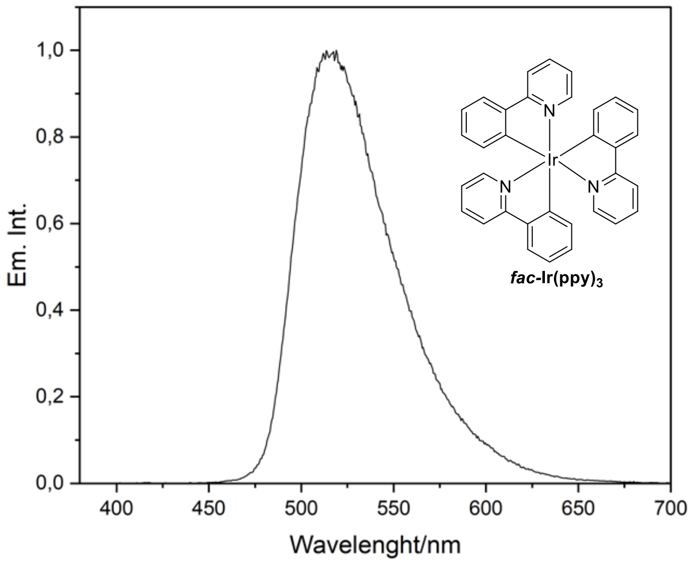

Ir(ppy)₂bpy(BF)₄ emission spectrum

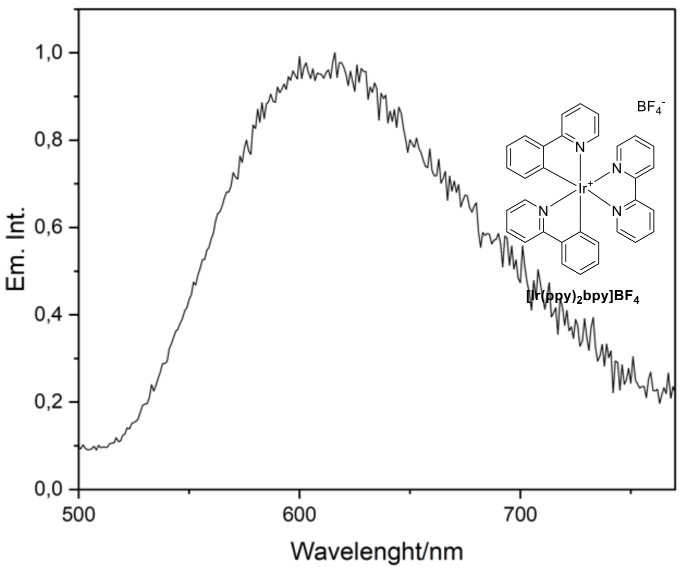

PC1 emission spectrum

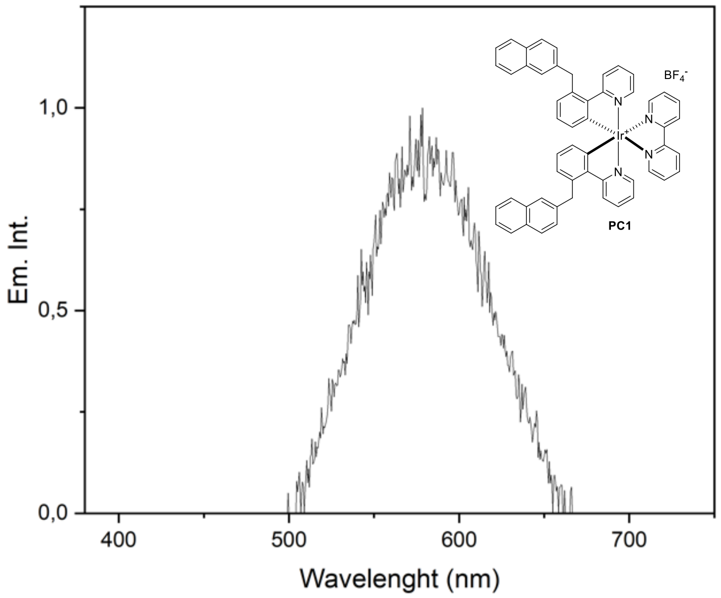

PC2 emission spectrum

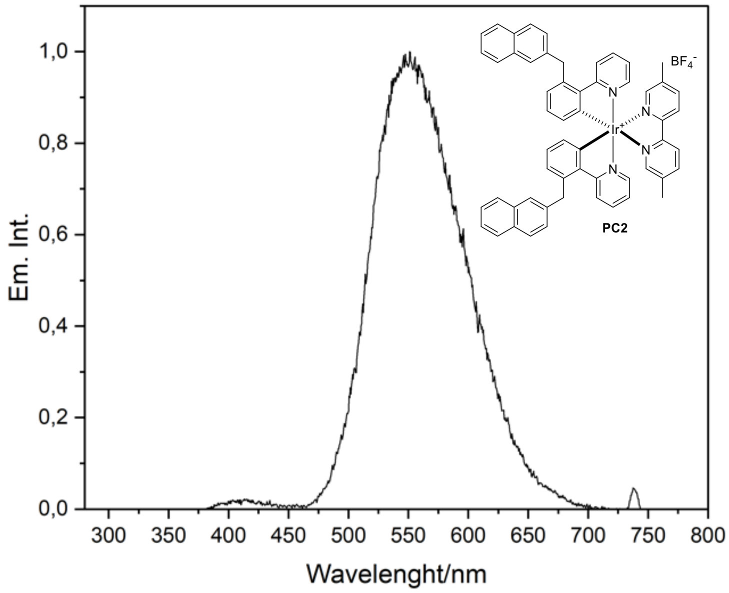

PC3 emission spectrum

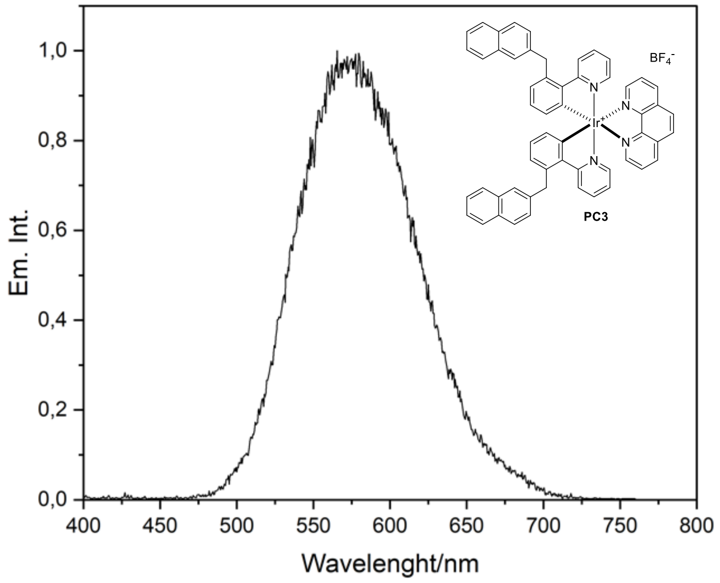

PC4 emission spectrum

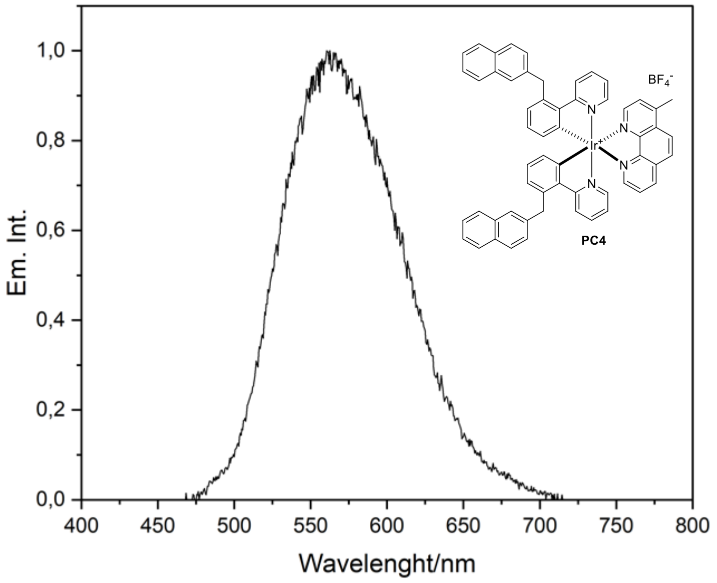

PC5 emission spectrum

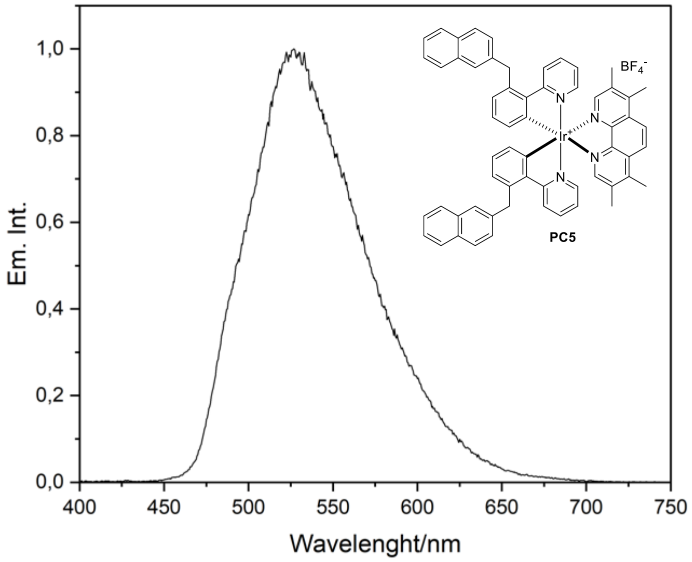

PC6 emission spectrum

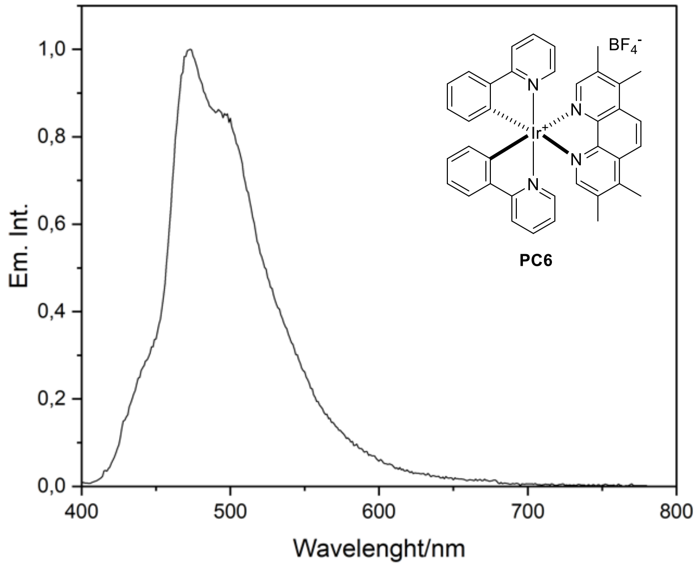

PC7 emission spectrum

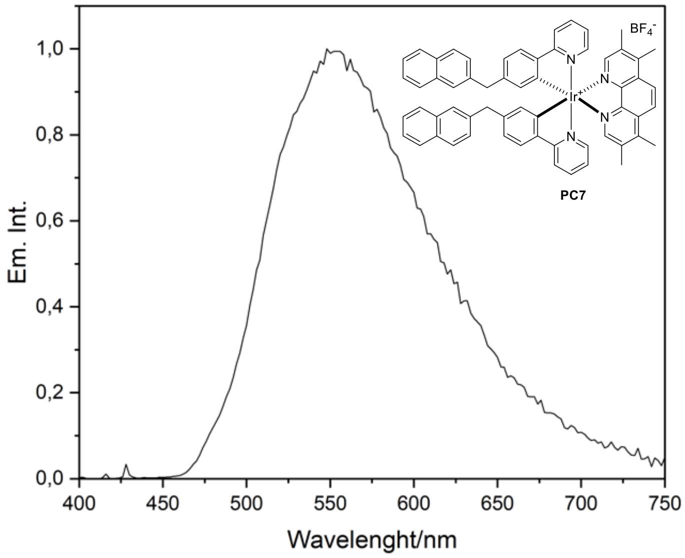

PC8 emission spectrum

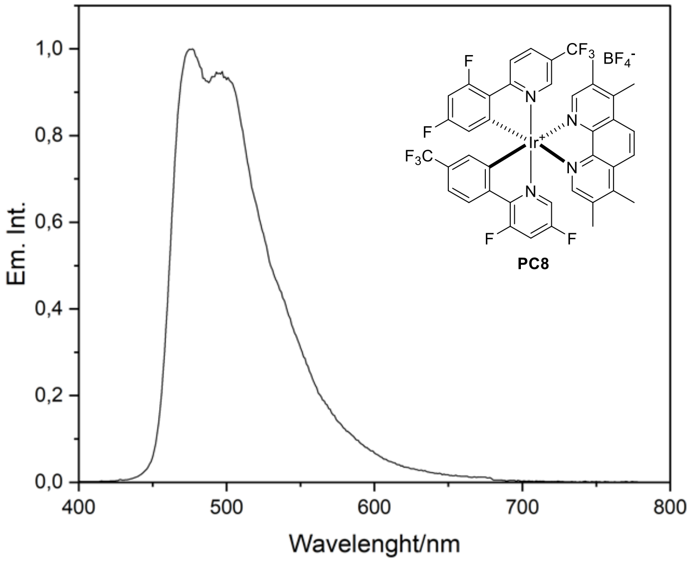

PC9 emission spectrum

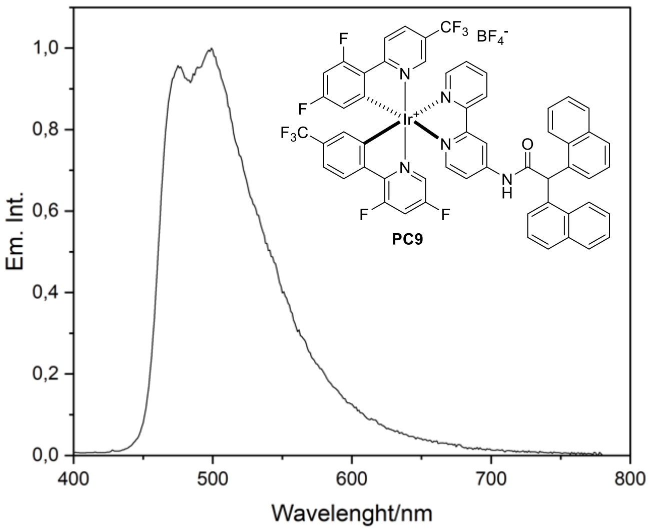

PC10 emission spectrum

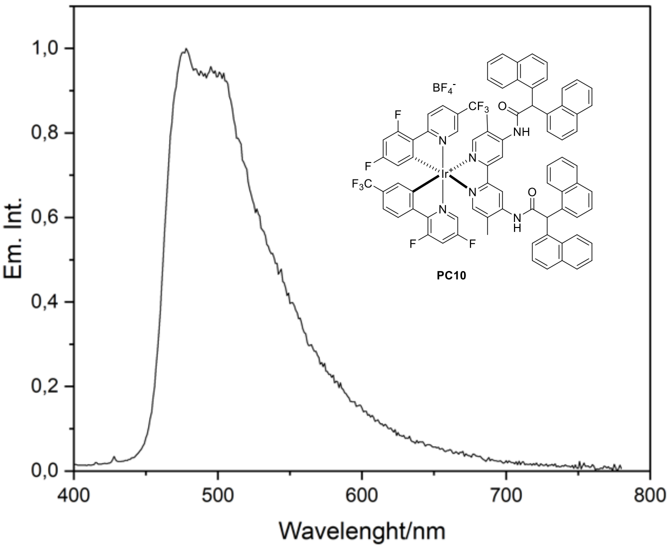

PC11 emission spectrum

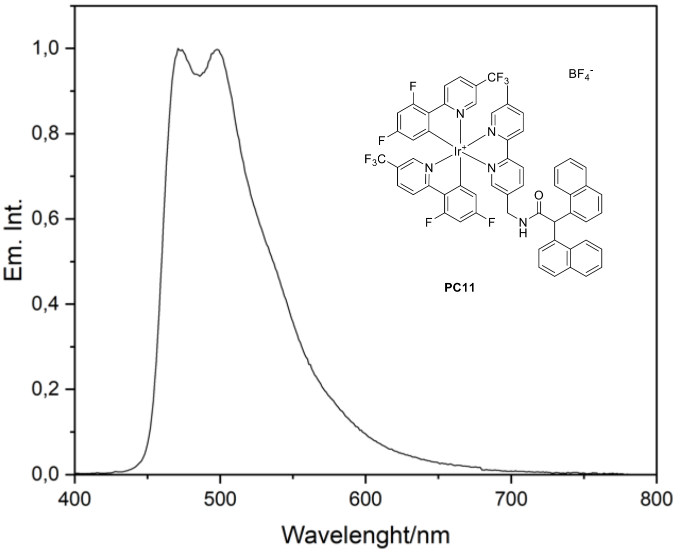

Photoluminescence Lifetime Measurements

Measurements of phosphorescence Decay lifetime were carried out with LS-55 Perkin Elmer spectrofluorimeter (**PC 1-6**) and with a FLS1000 Edinburgh Fluorometer (**PC 7-11**). Samples were prepared in CH_3CN. The concentration was adjusted to reach an absorption around 0.2 at the excitation wavelength. The exciting wavelength was set at 375 nm. The emission was collected at the corresponding maximum wavelength for each **PC**. A quartz cuvette (optical path = 1 cm) capped with a rubber septum was used. The solution was degassed bubbling nitrogen in it immediately prior to use.

Interpolation of the resulting Decay curve to recover the corresponding lifetime of excited species, was performed mathematically using the Floracle programme provided by Edinburgh Instruments. The interpolation algorithm was based on the following equation:

$$R(t) = B_1\, e^{(\frac{-t}{\tau_1})} + B_2\, e^{(\frac{-t}{\tau_2})}$$

The determination of a monoexponentially or biexponentially type fit of decay, was performed by analysis of result square sum of residues.

fac- Ir(ppy)₃ ‘s phosphorescence decay

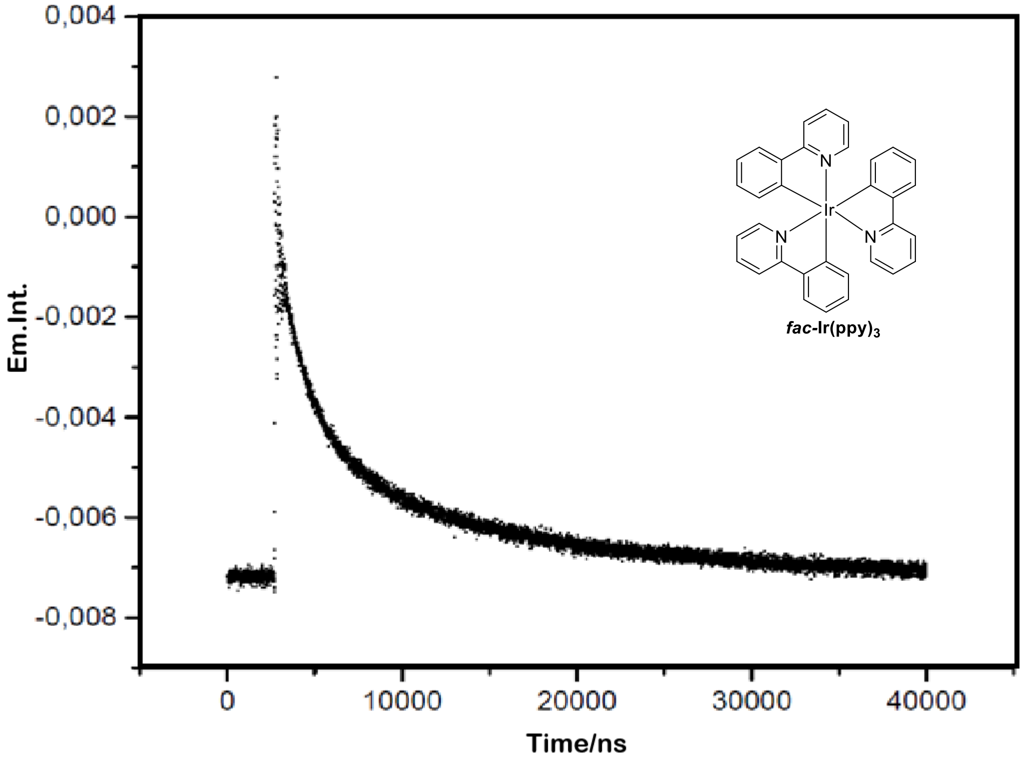

PC1 phosphorescence decay

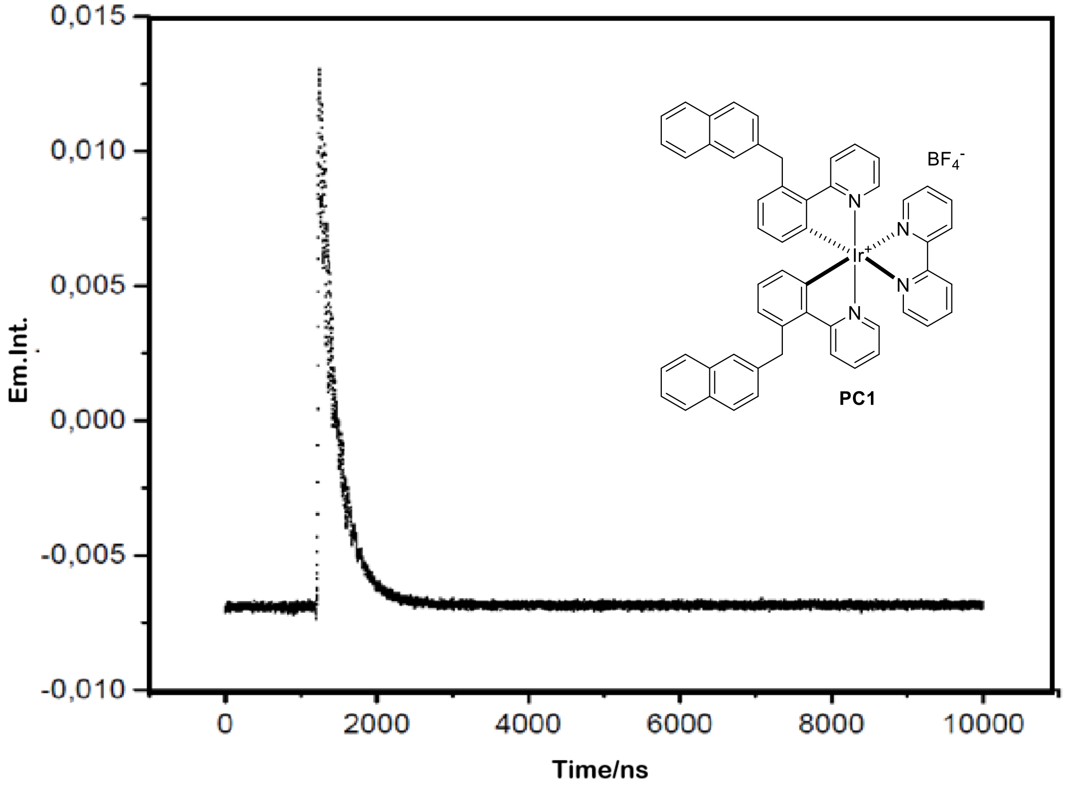

PC2 phosphorescence decay

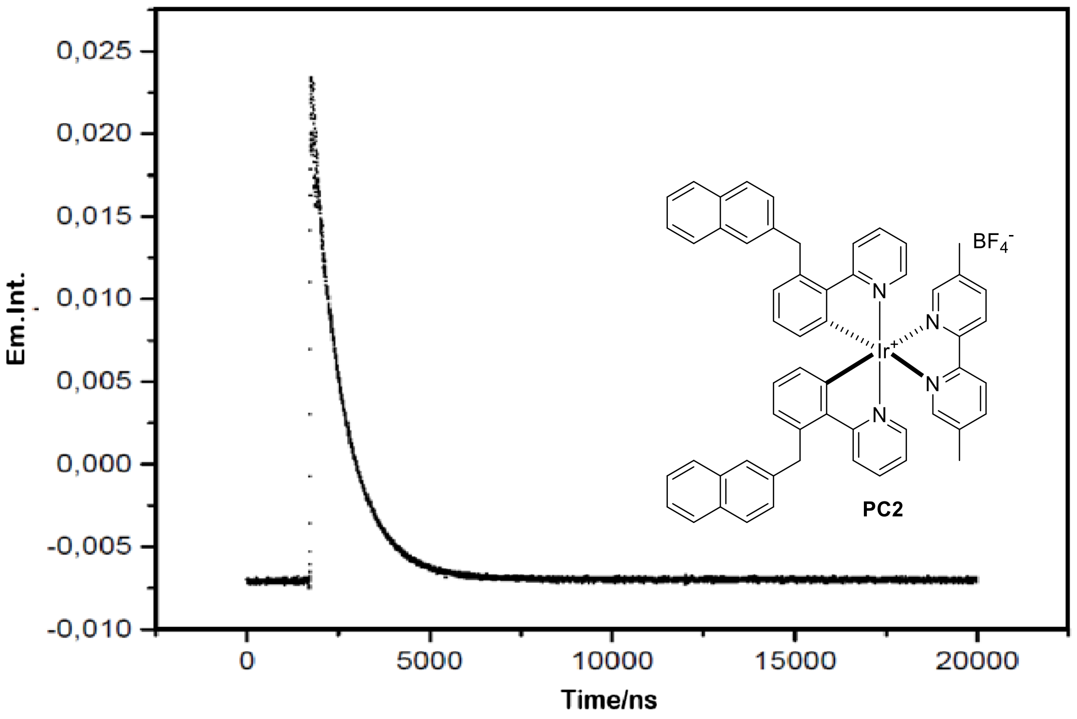

PC3 phosphorescence decay

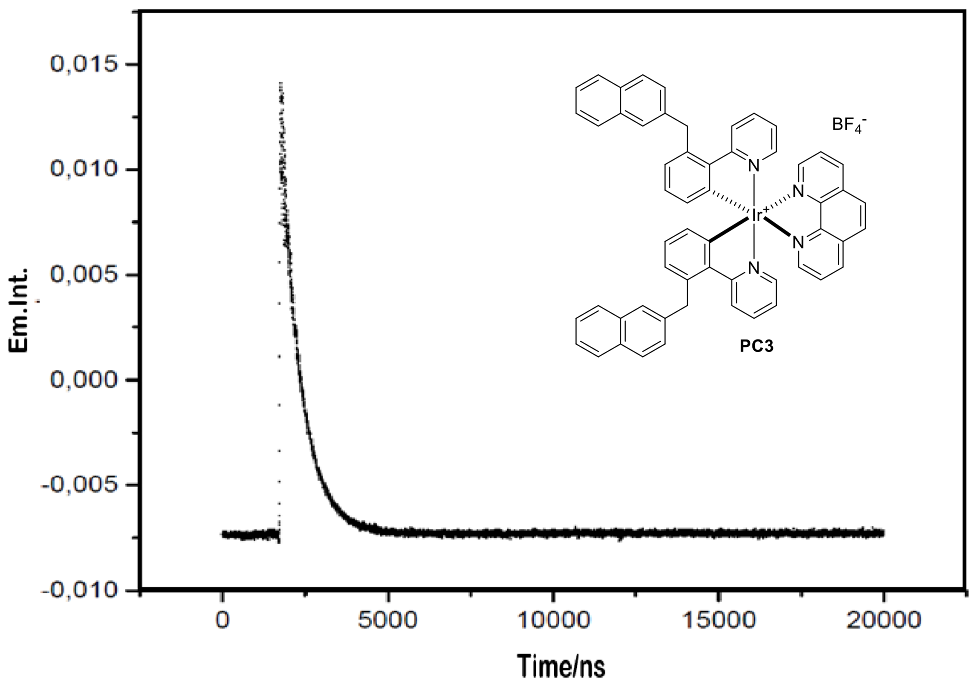

PC4 phosphorescence decay

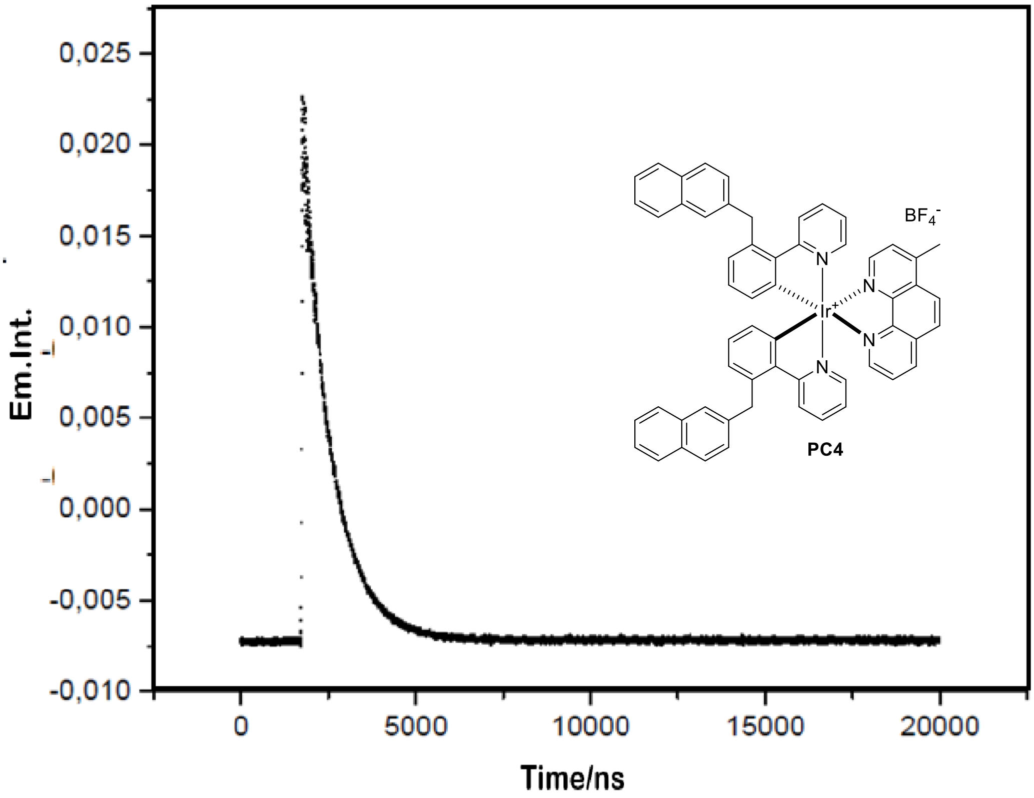

PC5 phosphorescence decay

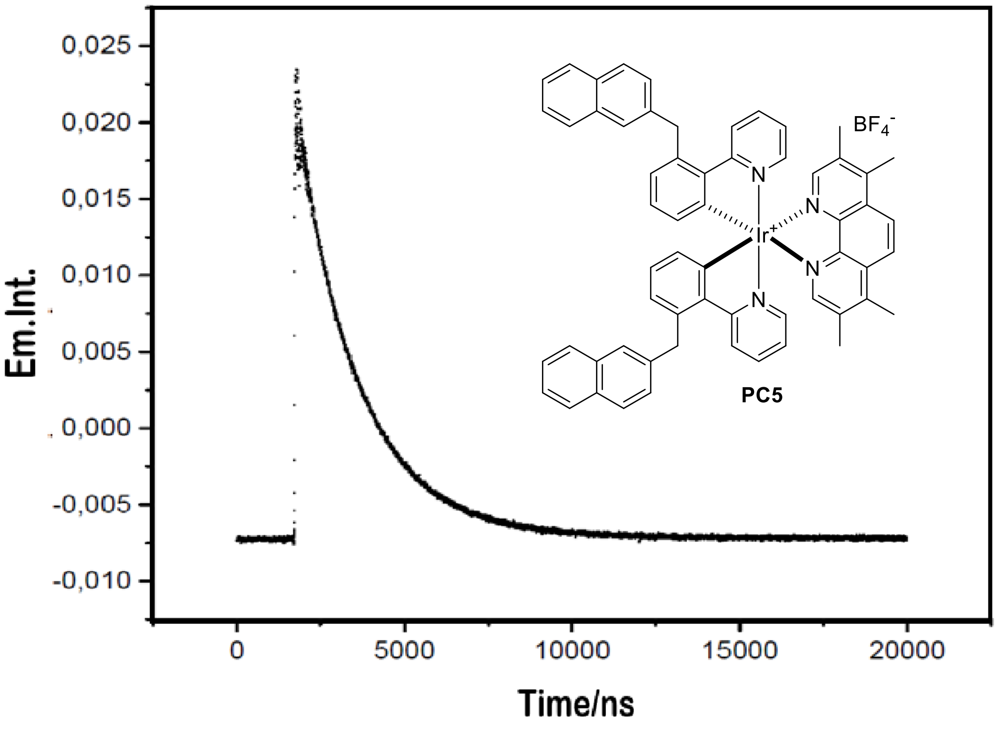

PC6 phosphorescence decay

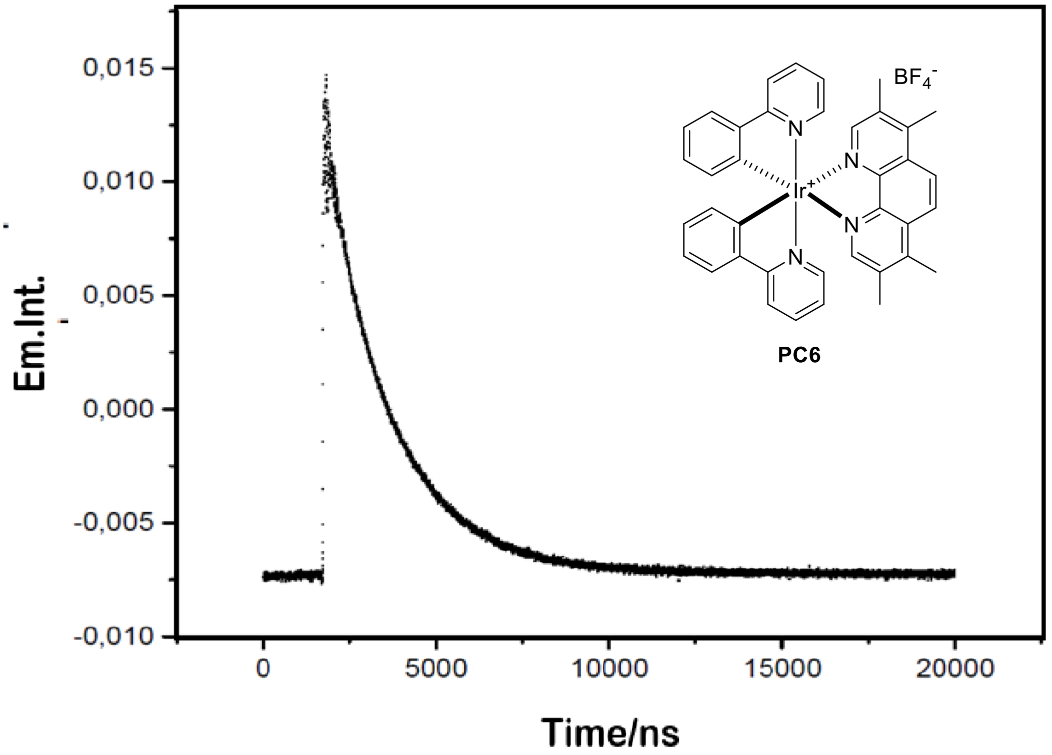

PC7 phosphorescence decay

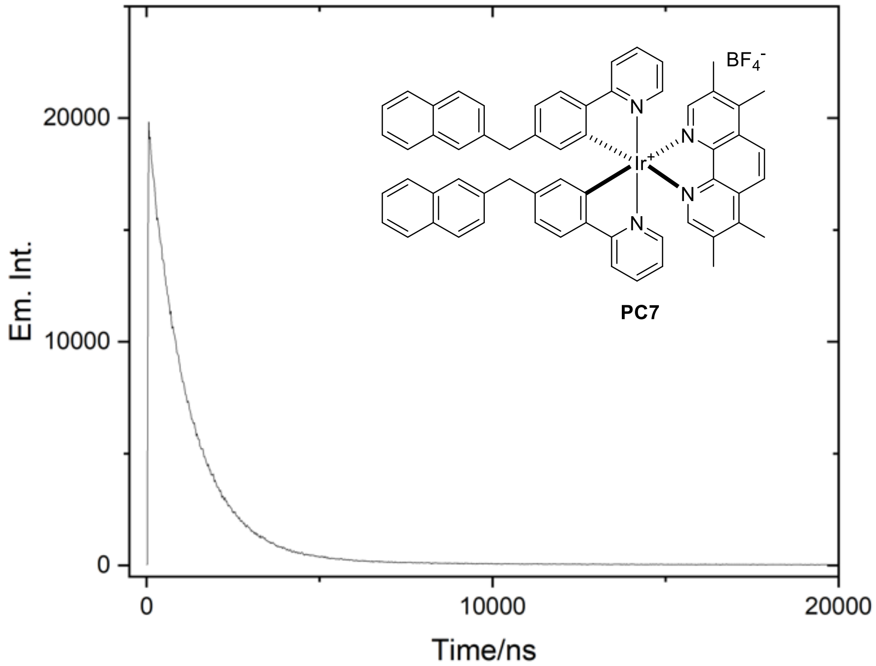

PC8 phosphorescence decay

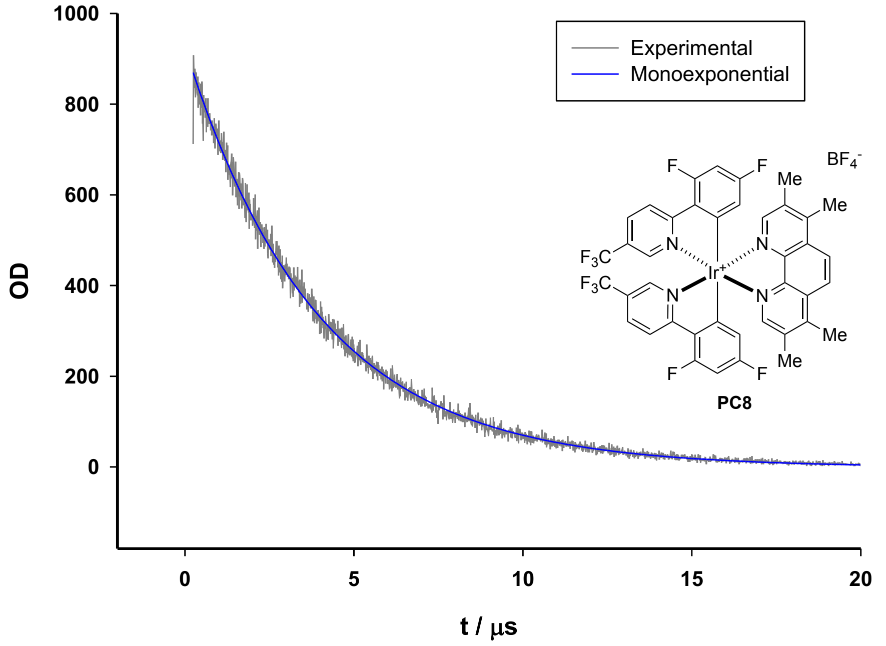

PC9 phosphorescence decay

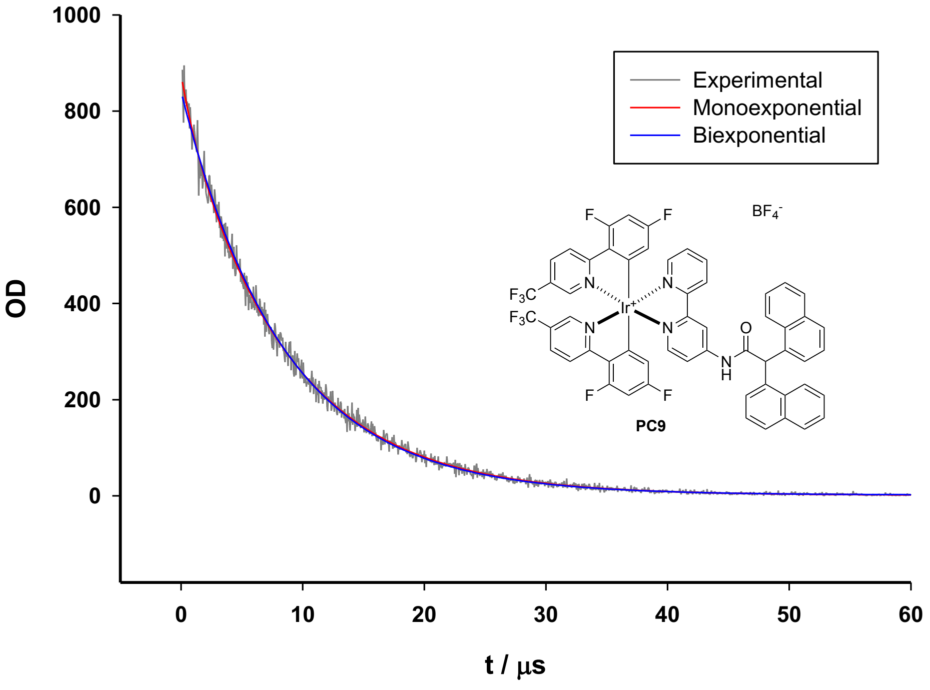

PC10 phosphorescence decay

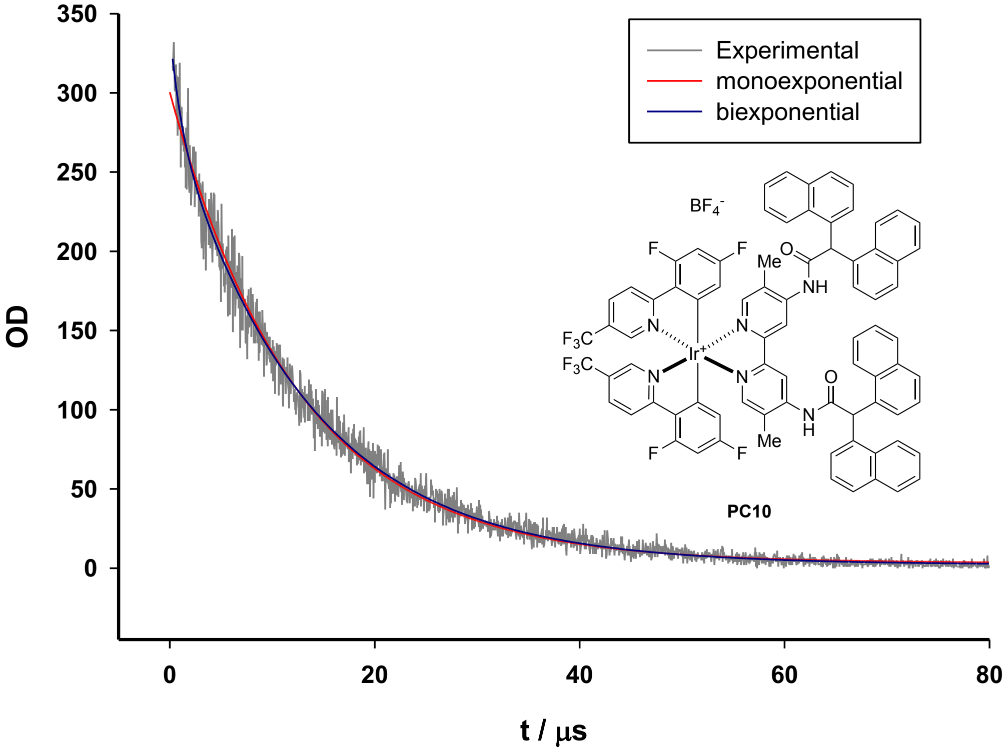

Mono exponential	Bi exponential
Y = y0 + a·e(-bx)<br>Y = y0 + a·e(-bx)<br>R2 = 0.9866<br>Y0 = 3.2±0.3<br>A = 297±1<br>B = 0.804±0.0004	Y = y0 + a·e(-bx)+c·e(-dx)<br>R2 = 0.9899<br>Y0 = 2.2<br>A = 53±1<br>C = 285±1<br>B = 0.93±0.1<br>D = 0.0762±0.0006
τ_1 = 12.44 µs	τ_1 = 1.08 µs<br>τ_2 = 13.1 µs

P11 phosphorescence decay

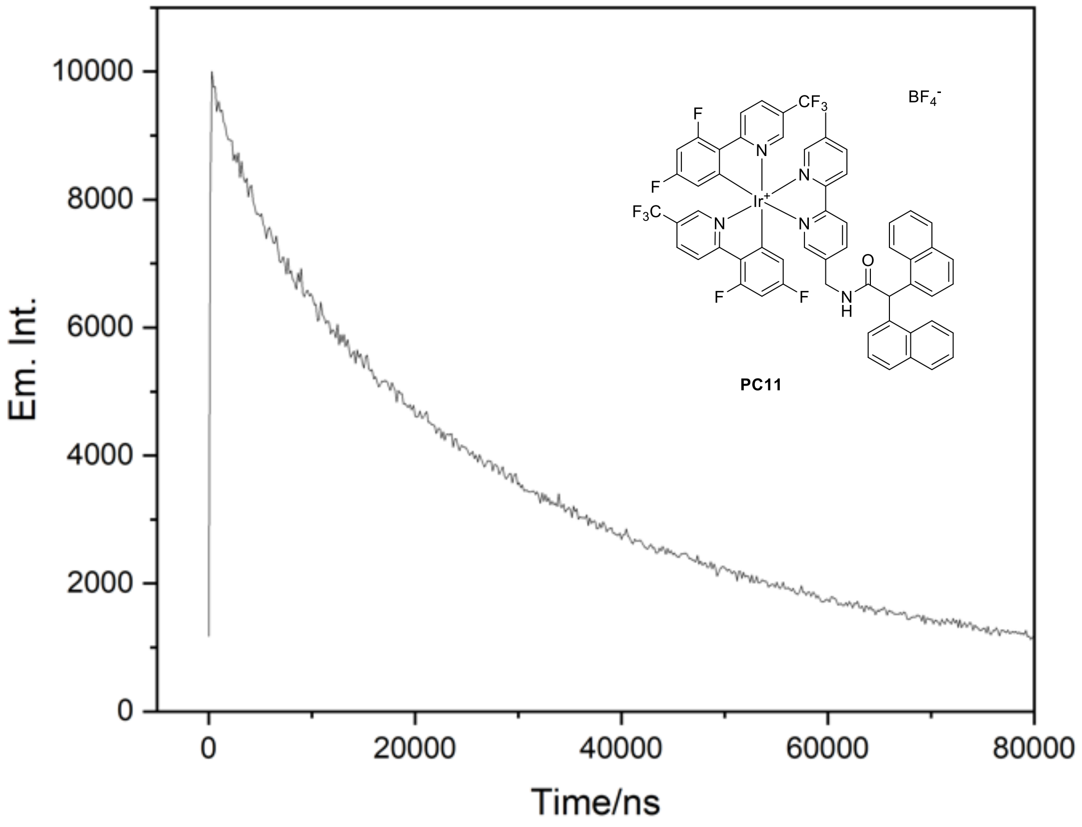

Mono exponential	Bi exponential
$Y = A1 \cdot e(-x/\tau_1) + y0$<br>R2 = 0.96629<br>Y0 = 980 ± 20<br>A = 8300 ± 20	$Y = y0 + A \cdot e(-x/\tau_1) + B \cdot e(-x/\tau_2)$<br>R2 = 0.9991<br>Y0 = 2.2<br>A = 1800±60<br>B = 7590±40
τ_1 = 25.1 μs	τ_1 = 4.84 μs<br>τ_2 = 32.5 μs

Quantum yield measurements in ACN

Quantum yield of complex **PC1-11** were determined fluorometrically relative to a fluorescent standard with a known quantum yield, using the method reported in literature.[73] To determine the quantum yield of **PC1-PC11** we used commercially available Ir(ppy)$_3$ as reference standard, according to the following equation:

$$Q = Q_r * \frac{I}{I_r} * \frac{A_r}{A} * \frac{n^2}{n_r^2}$$

Where Q_r is the quantum yield of reference compound, *I* is the integrated intensity of emission spectra reference compound, A_r is the absorbance at the excitation wavelength, *n* is the refractive index of the used solvent, and the subscript *r* refers to reference compounds. For acetonitrile solutions, we used *n=1.344* as refractive index value.

Quantum yield was determined using 1x10^{-5}M solution of complex **PC1-PC11**, and a standard solution of Ir(ppy)$_3$ 1x10^{-5}M freshly prepared from dilution of a 10^{-3}M starting solution prepared by dissolving a weighted amount of complex in dry CH_3CN. Measurements of Absorbance and Emission intensity were performed using the same instrumental conditions at $\lambda_{xcitation}$ of 380 nm for all samples.

PC	Abs at 380 nm	I	Q	Q%
**Ir(ppy)$_3$**	4.98E-02	637953.00	0.40	40.0
**Ir(ppy)$_2$(bpy)BF$_4$**	4.97E-02	1345776.00	0.08	8.5
**PC1**	1.87E-01	7790143.00	0.13	13.0
**PC2**	1.75E-01	9123674.00	0.16	16.2
**PC3**	2.23E-01	9285718.00	0.13	13.0
**PC4**	1.49E-01	9371295.00	0.20	20.0
**PC5**	1.91E-01	8402333.00	0.14	14.0
**PC6**	2.95E-01	7988775.00	0.08	8.0
**PC7**	2.50E-01	12159064.00	0.15	15.2
**PC8**	7.22E-02	7813481.00	0.33	33.8
**PC9**	7.94E-02	8919279.00	0.35	35.0
**PC10**	5.88E-02	8950281.00	0.48	47.5
**PC11**	4.77E-02	8524068.00	0.56	55.8

Triplet energy measurements in ACN

Triplet energy measurements were performed using the data from collected emission spectra of degassed $1x10^{-5}$ M solutions of **PC1-11** in dry ACN at 375 nm. $\lambda_{intersection}$ was determined mathematically from the intersection between the line tangential to the inflection point of the ascending portion of the curve and the x axis.

$$E_t = \frac{h * c_0 * N_a}{\lambda_{Intersection}} * 0.2388 * 10^6 [\left(\frac{Kcal}{mol}\right)]$$

h= Plank constant; *c_o= light speed in vacuum; N_a= Avogadro number.*

PC	Intersection (nm)	Et (Kcal/mol)
PC1	504.0	56.7
PC2	493.4	57.9
PC3	506.7	56.4
PC4	499.8	57.2
PC5	466.8	61.3
PC6	464.2	61.6
PC7	480.4	59.5
PC8	453.5	63.0
PC9	451.0	63.4
PC10	454.0	62.9
PC11	446.3	64.0

Quenching Experiments

Phosphorescence Quenching experiments were recorded on a FLS 980 Edinburg with a resolution of 1 nm. Samples were prepared in ACN. The concentration of the photocatalyst was adjusted to reach an absorption around 0.2 at the excitation wavelength. The exciting wavelength was set at 380 nm. A quartz cuvette (optical path = 1 cm) capped with a rubber septum was used. The solution was degassed bubbling nitrogen in it immediately prior to use and each time after the additions of the quencher.

Quenching of **PC3** with substrate **C**

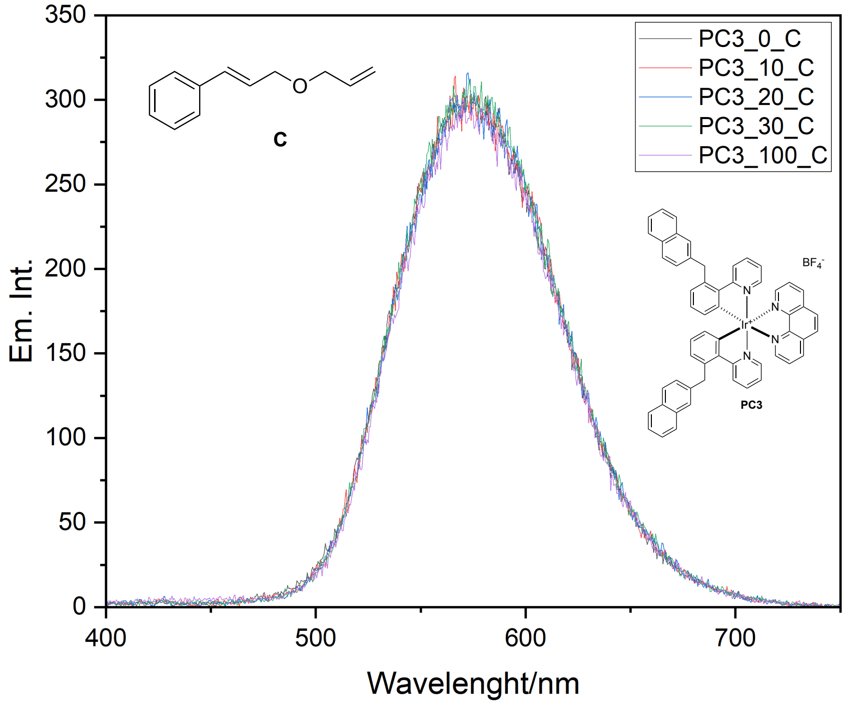

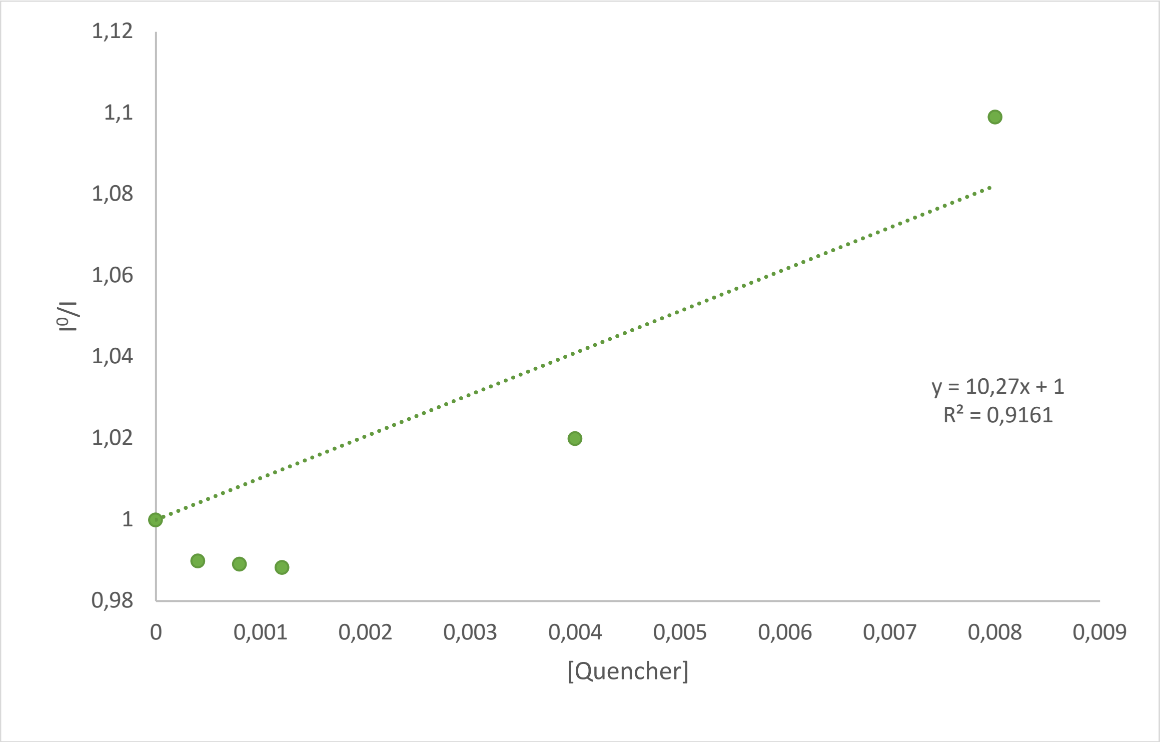

R^2	τ_0 [ns]	**K_{SV}** [M^{-1}]	**Kq** [$(M*ns)^{-1}$]
0.9161	609	10.3	0,016864

Quenching of **PC5** with substrate **C**

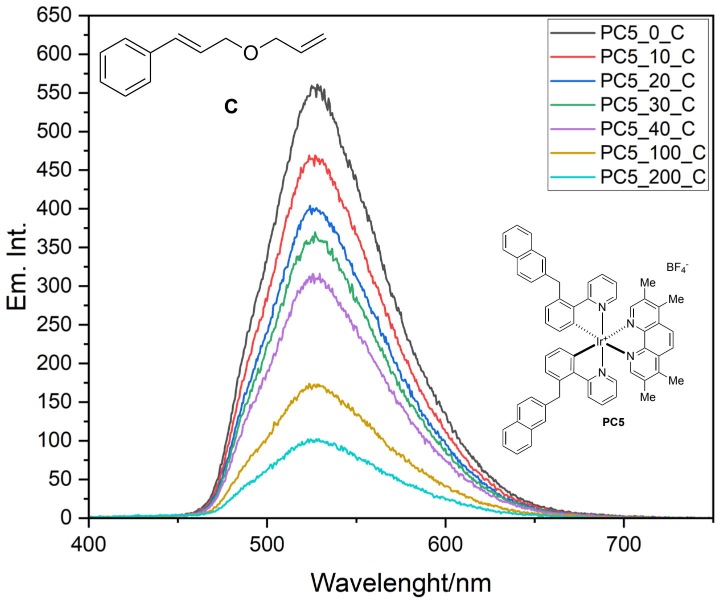

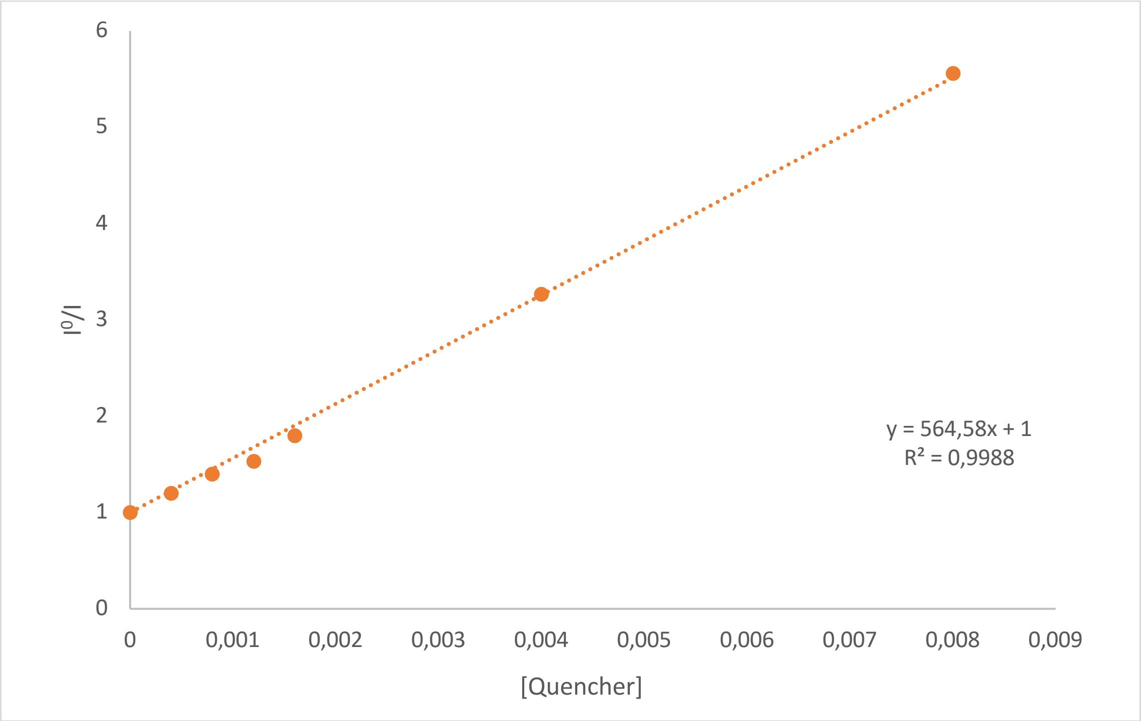

R^2	τ_0 [ns]	**K$_{SV}$** [M^{-1}]	**Kq** [(M*ns)$^{-1}$]
0.9988	1825	564	0,3090411

Quenching of **PC6** with substrate **C**

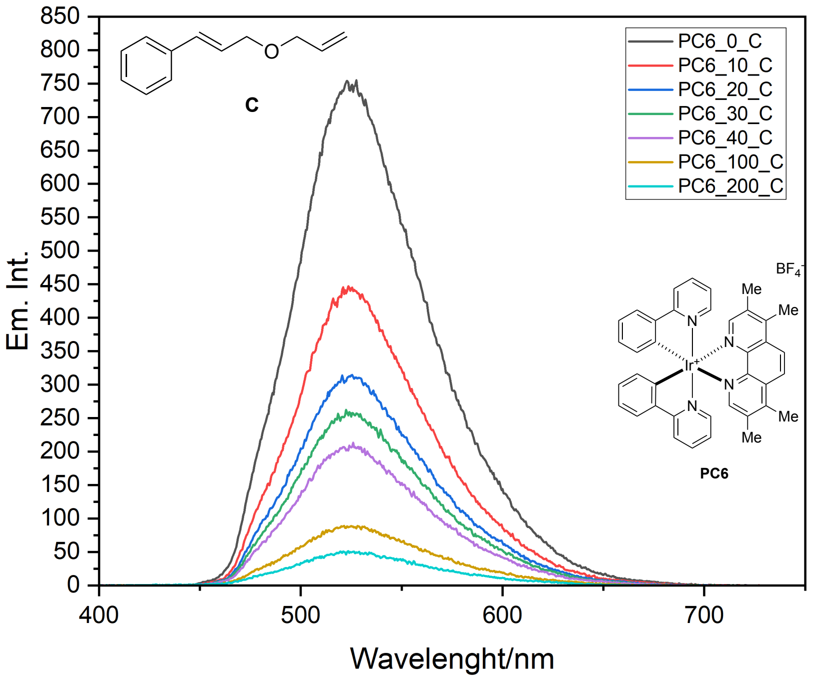

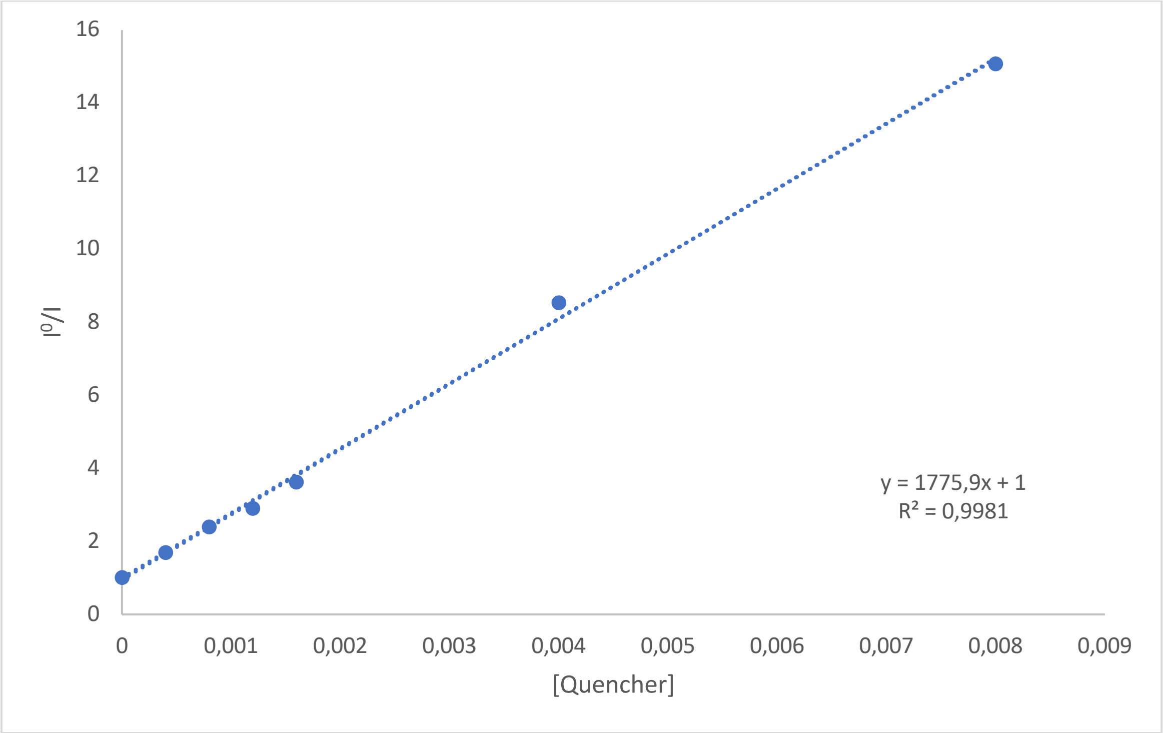

R^2	τ_0 [ns]	K_{SV} [M^{-1}]	Kq [$(M*ns)^{-1}$]
0,9981	1867	1776	0,951259

Cyclic Voltammetry Experiments

All the spectra were measure with IKA ElectraSyn 2.0 in dry and degassed CH_3CN using a glassy carbon disc working electrode, a platinum plate counter electrode and Ag/AgCl as reference electrode. The excited state redox potential were estimated from the measured ground state redox potential and the excited state Energy $E_{0,0}$ as described by Fox.[74-75]

$$E_{ox}^{*} = E_{ox} - E(0,0)/e$$
$$E_{red}^{*} = E_{red} + E(0,0)/e$$

Where E_{ox} and E_{red} are the oxidation and reduction potential calculated from CV measurements, E(0,0) is the Calculated energy of triplet states from emission spectra and e is the elemental charge.

Blank CV of $TBAPF_6$ 0.1M in CH_3CN.

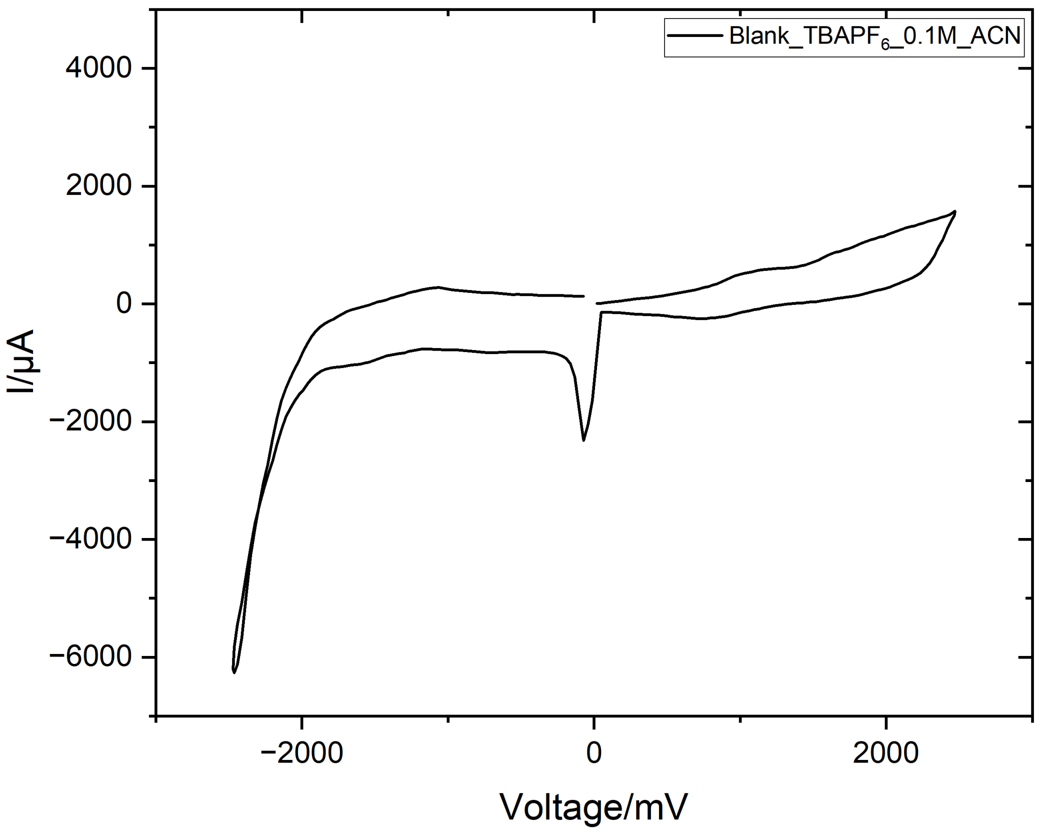

Conditions: 3 Segments, Initial Voltage: 0 mV, Direction: rising, Upper Voltage: 2.5V, Lower Voltage: -2.5V, Final Voltage: 0 mV, Sweep: 600 (mV/s)

CV of **PC5** (2mM) in CH_3CN with $TBAPF_6$ 0.1M as electrolyte.

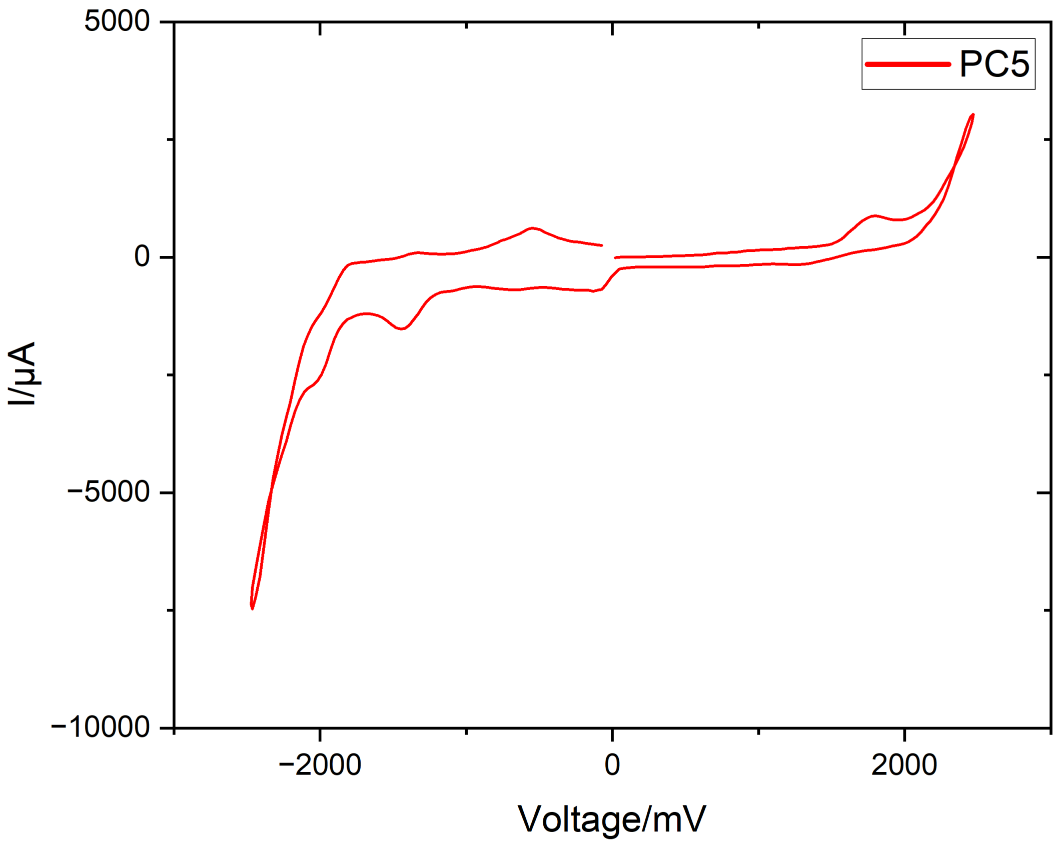

Conditions: 3 Segments, Initial Voltage: 0 mV, Direction: rising, Upper Voltage: 2.5V, Lower Voltage: -2.5V, Final Voltage: 0 mV, Sweep: 600 (mV/s)

PC5	V
**Ered (V vs Ag/AgCl)**	-1.46
***Ered (V vs Ag/AgCl)**	1.20
**Eox (V vs Ag/AgCl)**	1.78
***Eox (V vs Ag/AgCl)**	-0.88

CV of **PC6** (2mM) in CH_3CN with $TBAPF_6$ 0.1M as electrolyte.

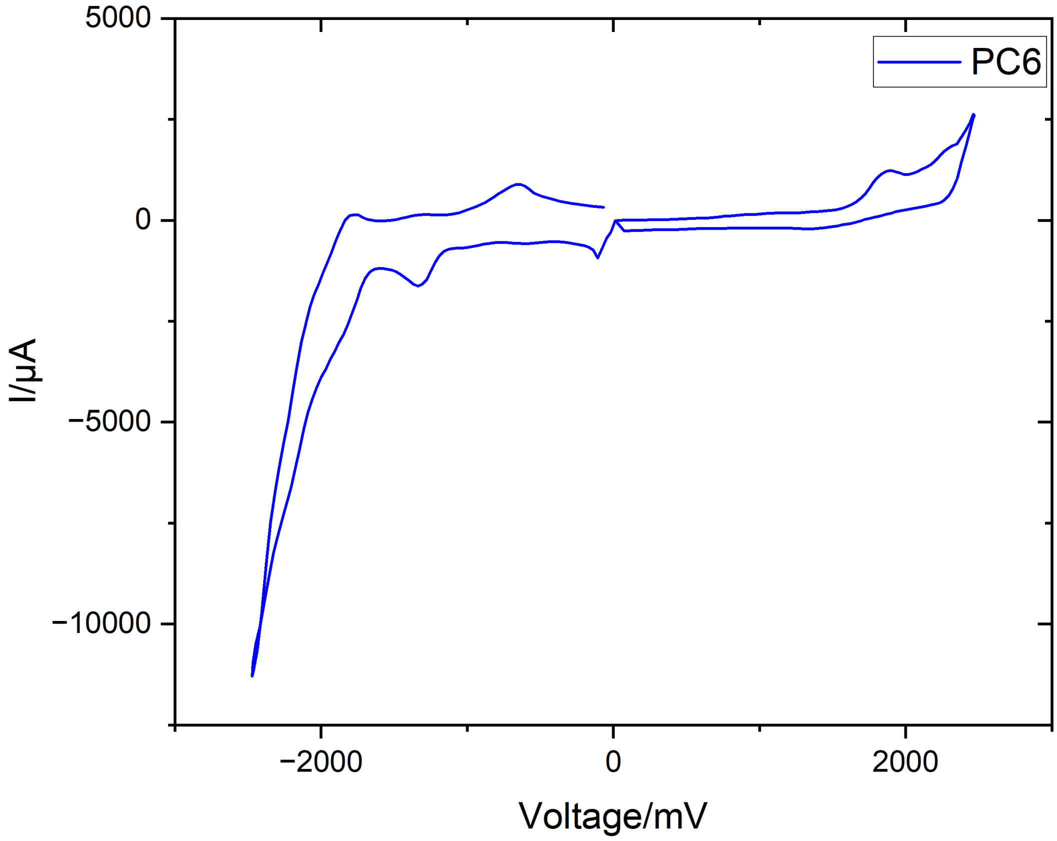

Conditions: 3 Segments, Initial Voltage: 0 mV, Direction: rising, Upper Voltage: 2.5V, Lower Voltage: -2.5V, Final Voltage: 0 mV, Sweep: 600 (mV/s)

PC6	V
**Ered (V vs Ag/AgCl)**	-1.35
***Ered (V vs Ag/AgCl)**	1.32
**Eox (V vs Ag/AgCl)**	1.89
***Eox (V vs Ag/AgCl)**	-0.78

CV of **PC11** (2mM) in CH_3CN with $TBAPF_6$ 0.1M as electrolyte.

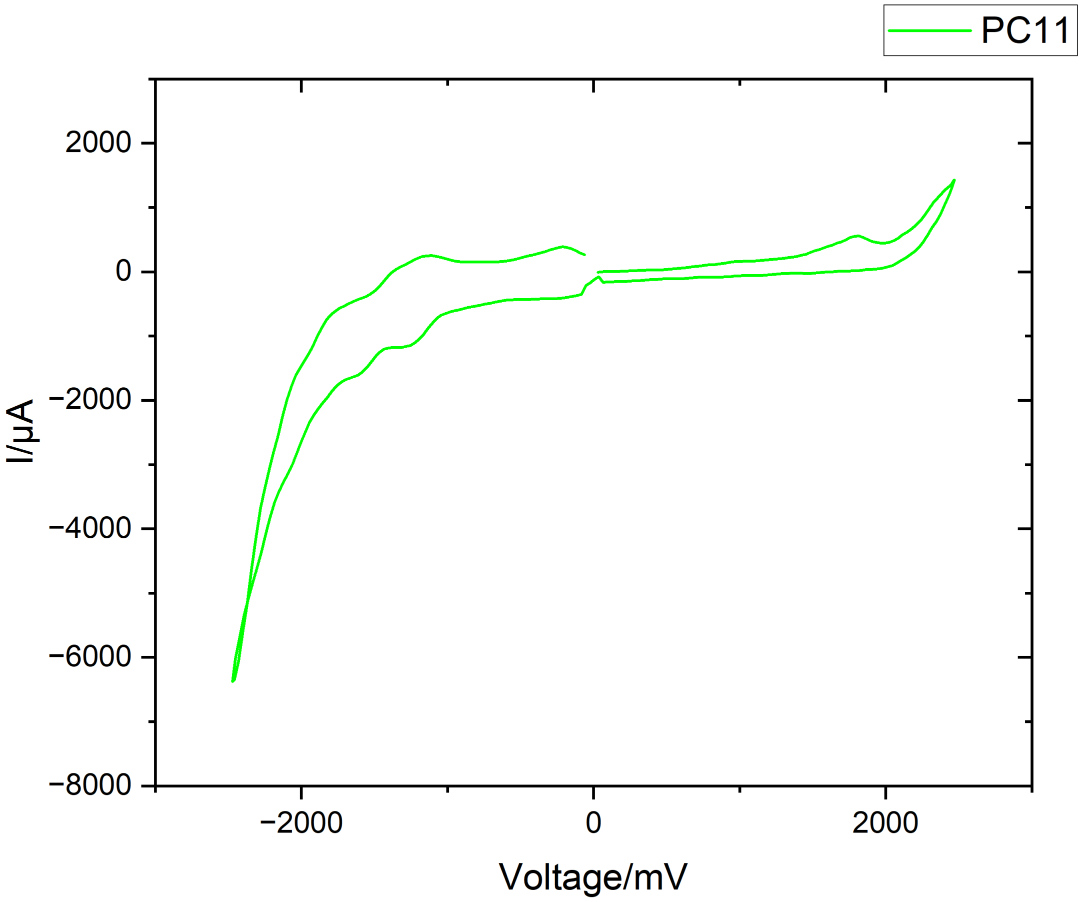

Conditions: 3 Segments, Initial Voltage: 0 mV, Direction: rising, Upper Voltage: 2.5V, Lower Voltage: -2.5V, Final Voltage: 0 mV, Sweep: 600 (mV/s)

**PC11**	V
**Ered (V vs Ag/AgCl)**	-1.61
***Ered (V vs Ag/AgCl)**	1.17
**Eox (V vs Ag/AgCl)**	1.79
***Eox (V vs Ag/AgCl)**	-0.99

Copies of NMR spectra

Characterization of the Ligands

L1$_{C^\wedge N}$, ^{1}H NMR (400 MHz, $CDCl_3$)

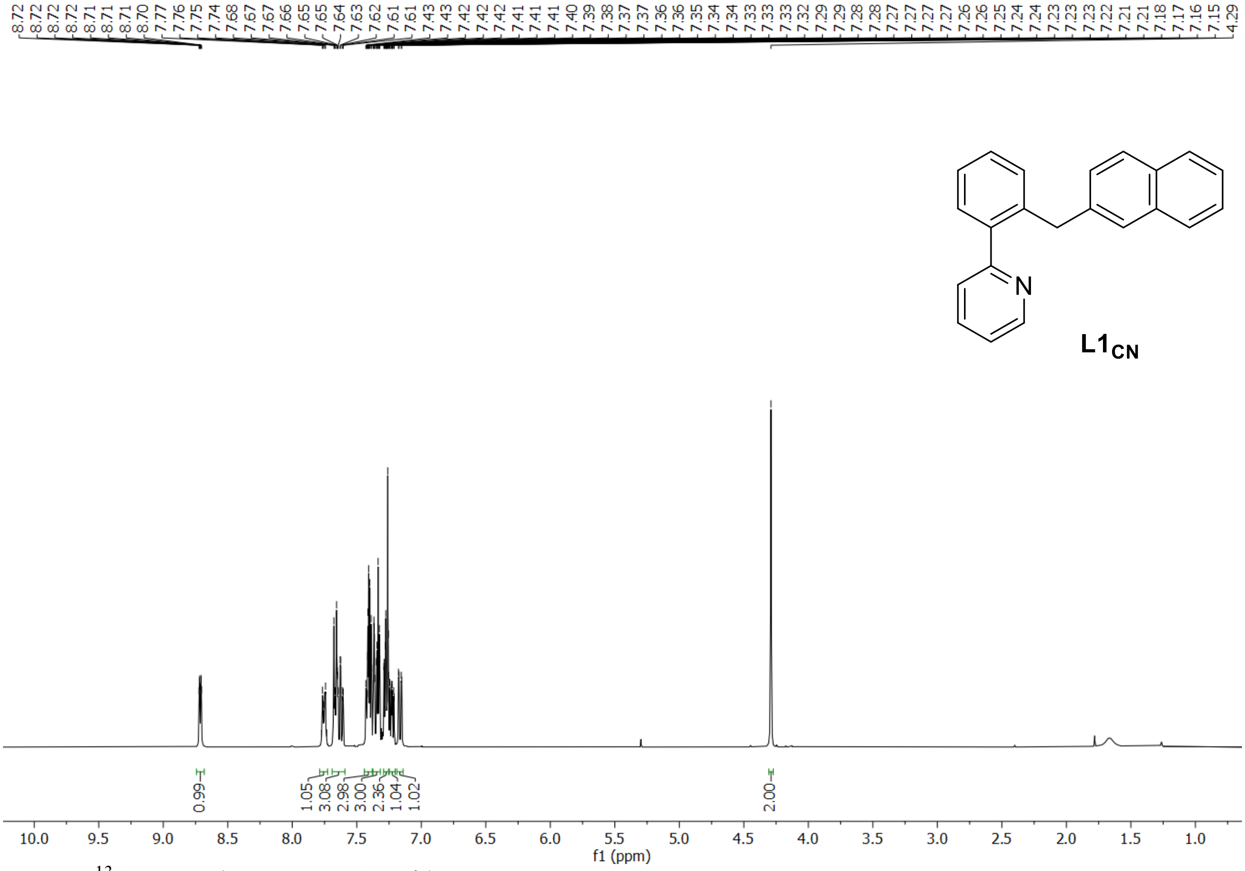

L1$_{CN}$

L1$_{C^\wedge N}$, ^{13}C NMR (101 MHz, $CDCl_3$)

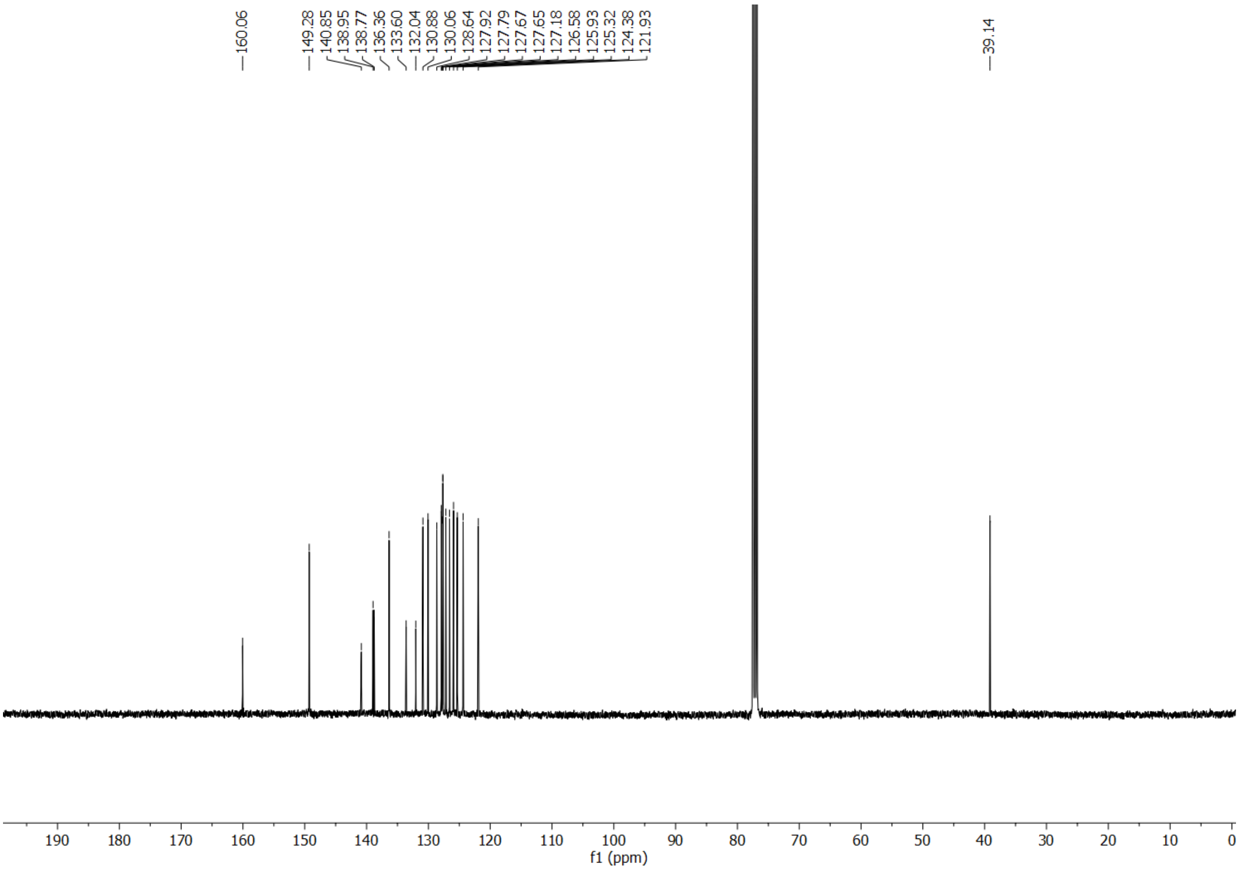

2-(4-bromophenyl)pyridine, ^{1}H NMR (400 MHz, $CDCl_3$)

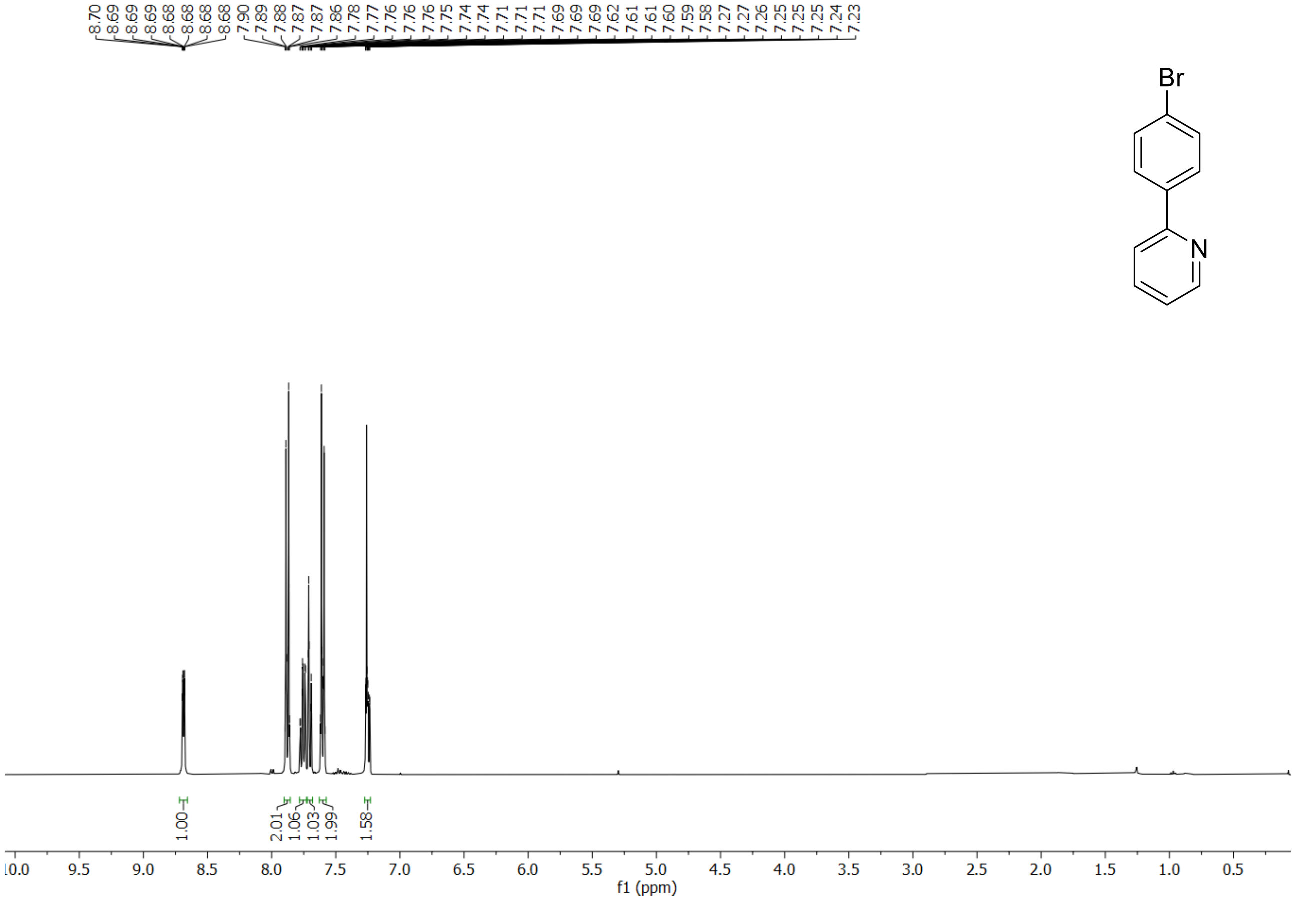

L2$_{C^N}$, ^{1}H NMR (400 MHz, $CDCl_3$)

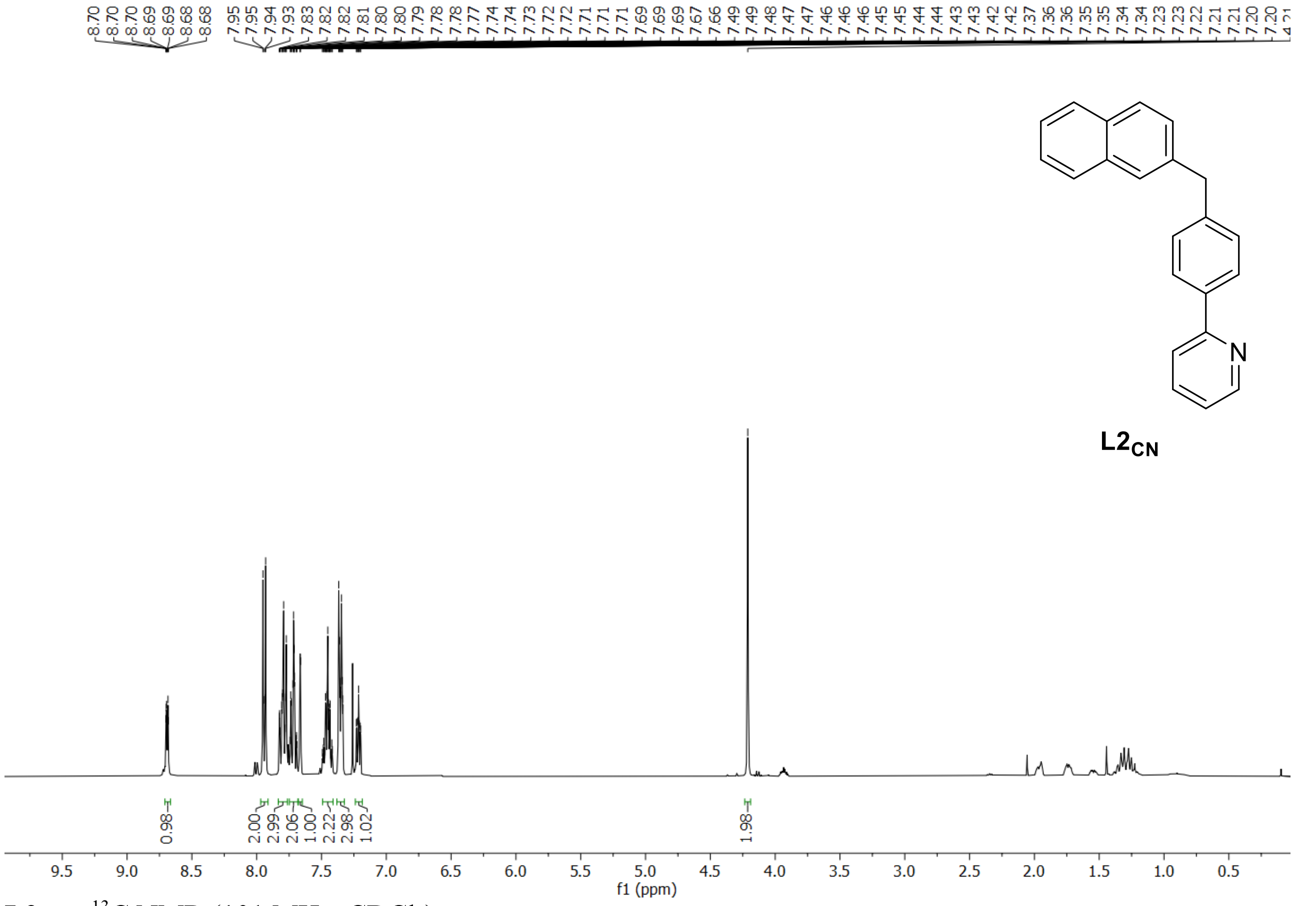

L2$_{C^N}$, ^{13}C NMR (101 MHz, $CDCl_3$)

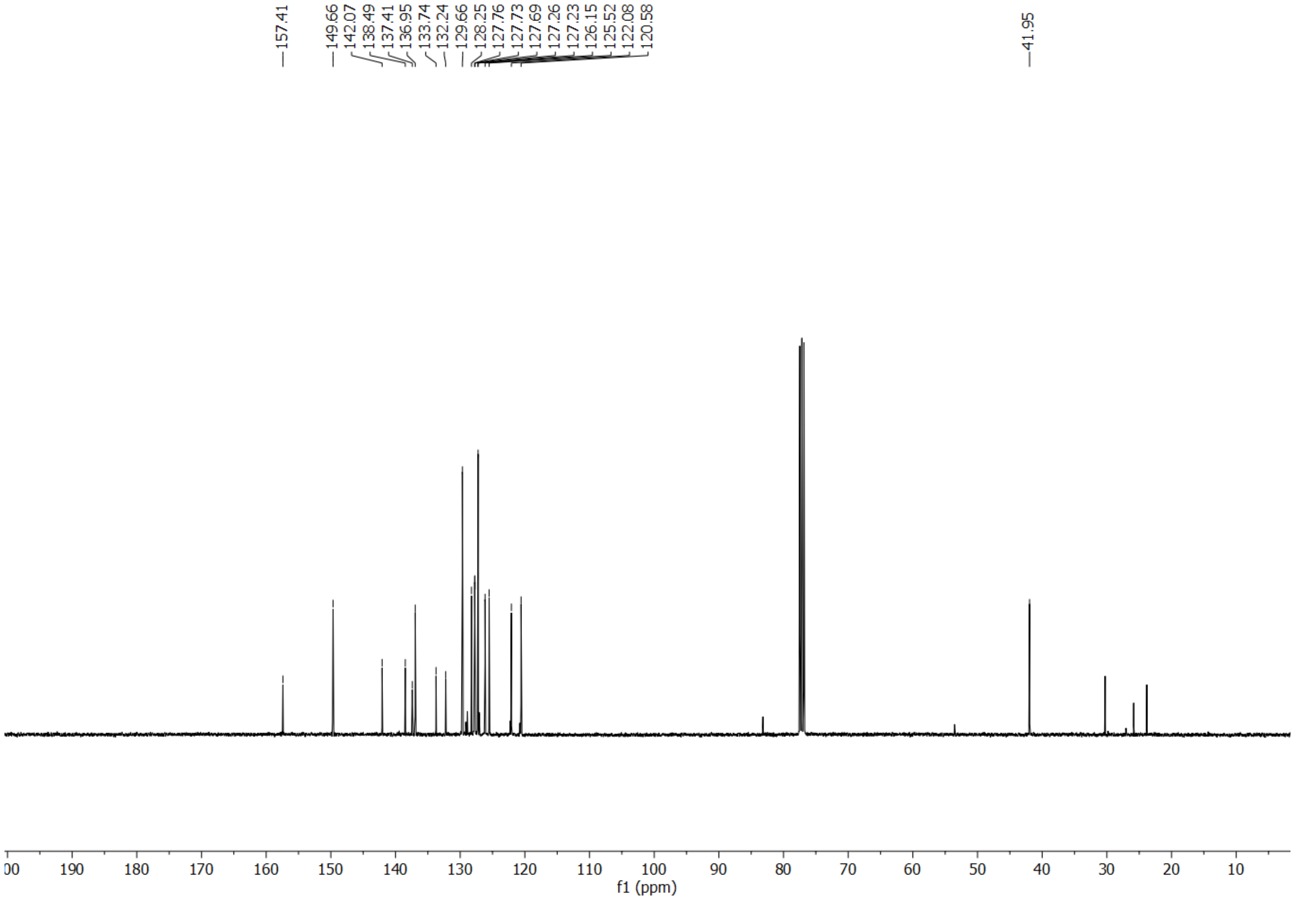

L3$_{C^N}$, ^{1}H NMR (400 MHz, $CDCl_3$)

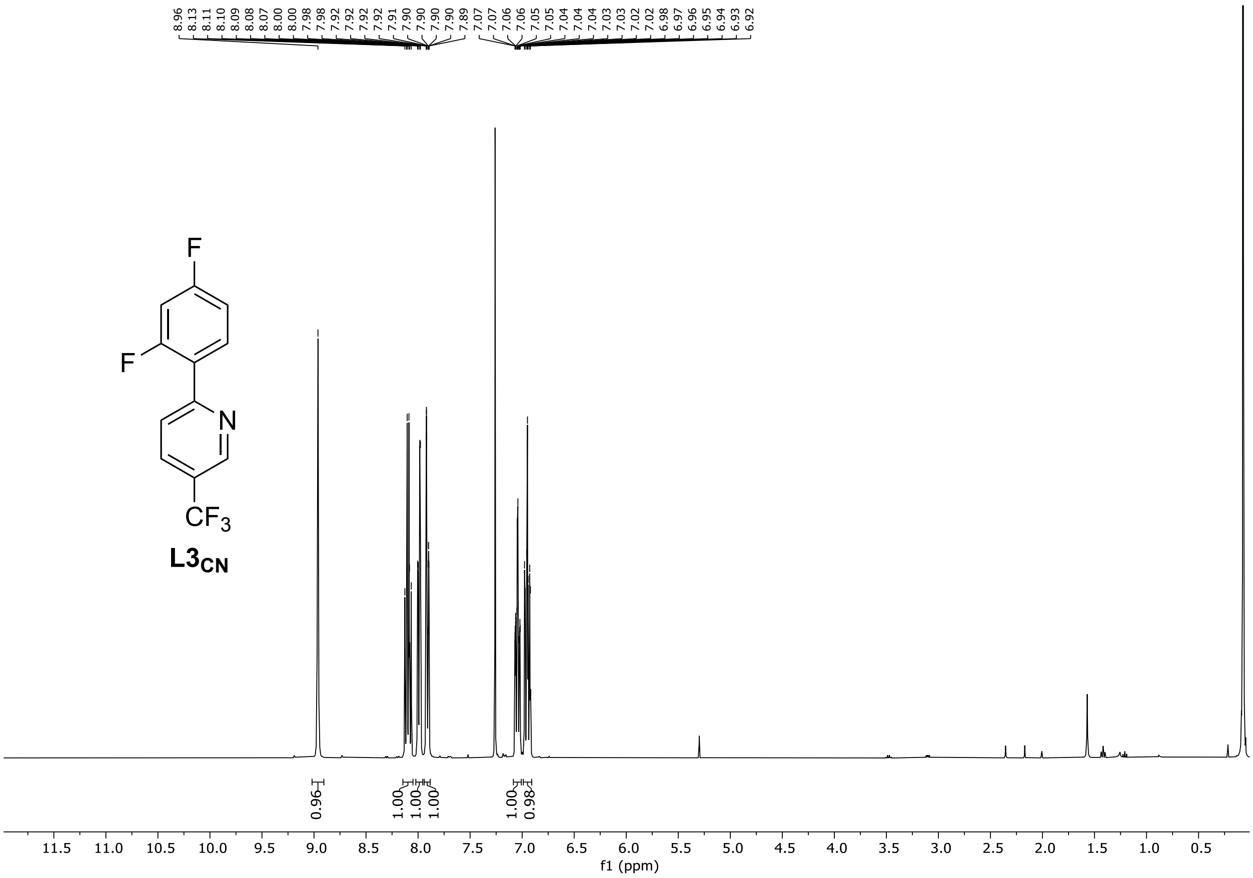

L3$_{C^N}$, ^{13}C NMR (101 MHz, $CDCl_3$)

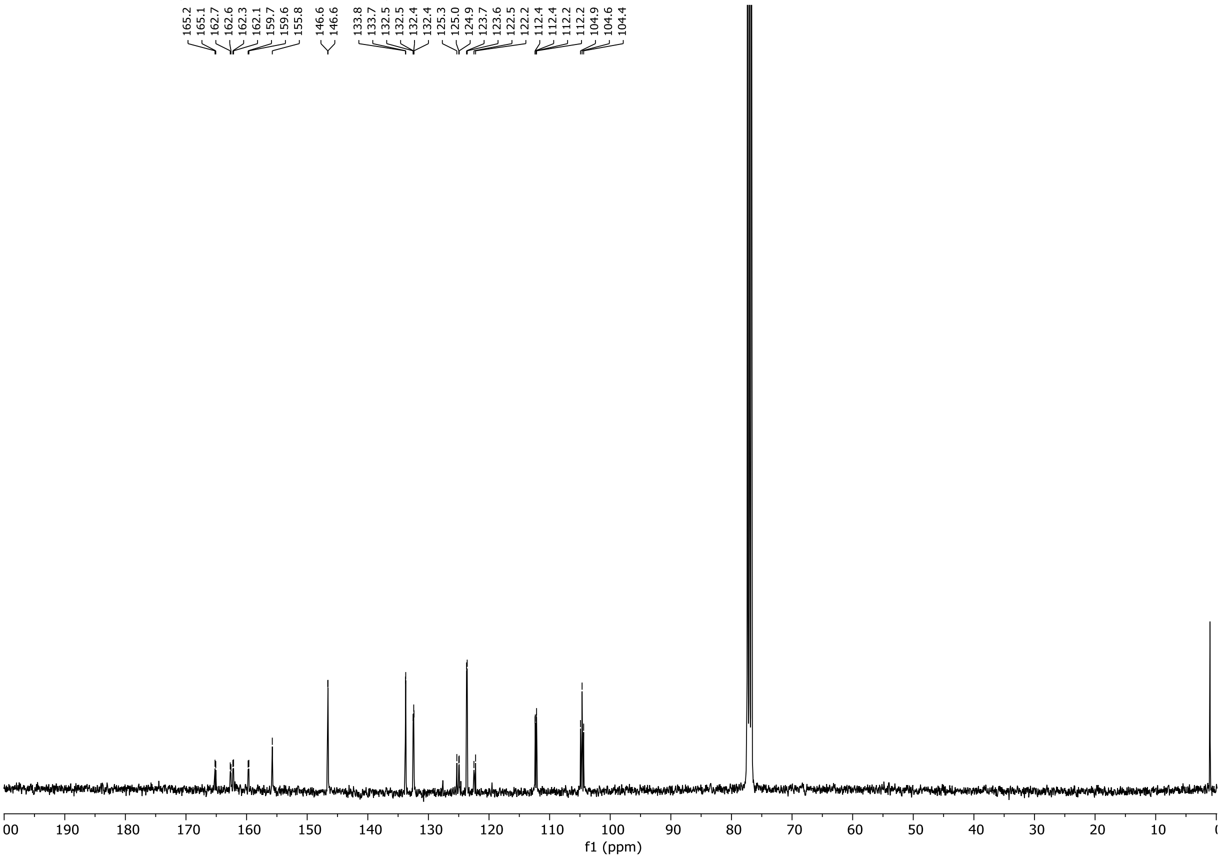

L3$_{C^N}$, ^{19}F NMR (565 MHz, $CDCl_3$)

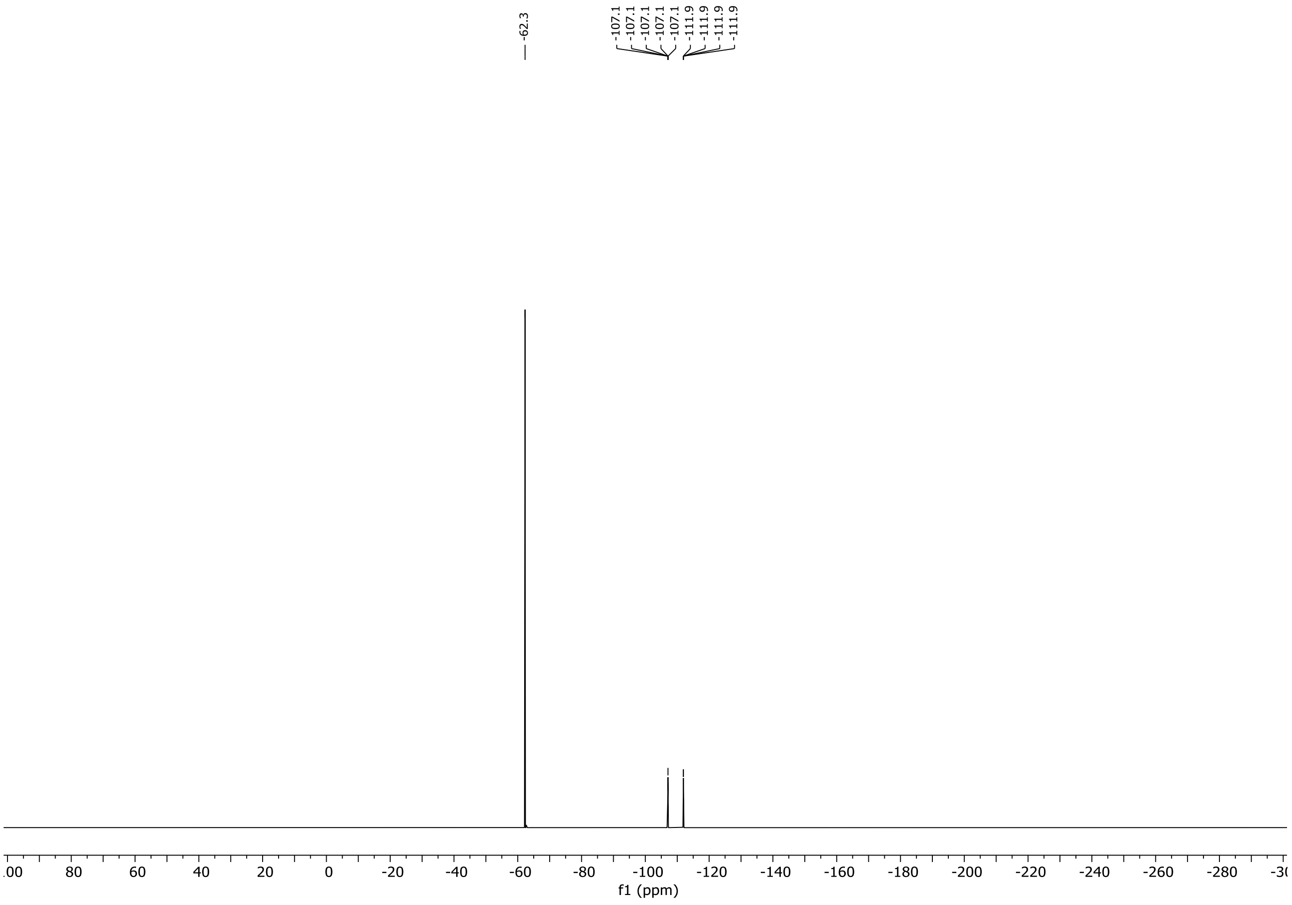

Di(naphthalen-1-yl)methanol, ^{1}H NMR (400 MHz, $CDCl_3$)

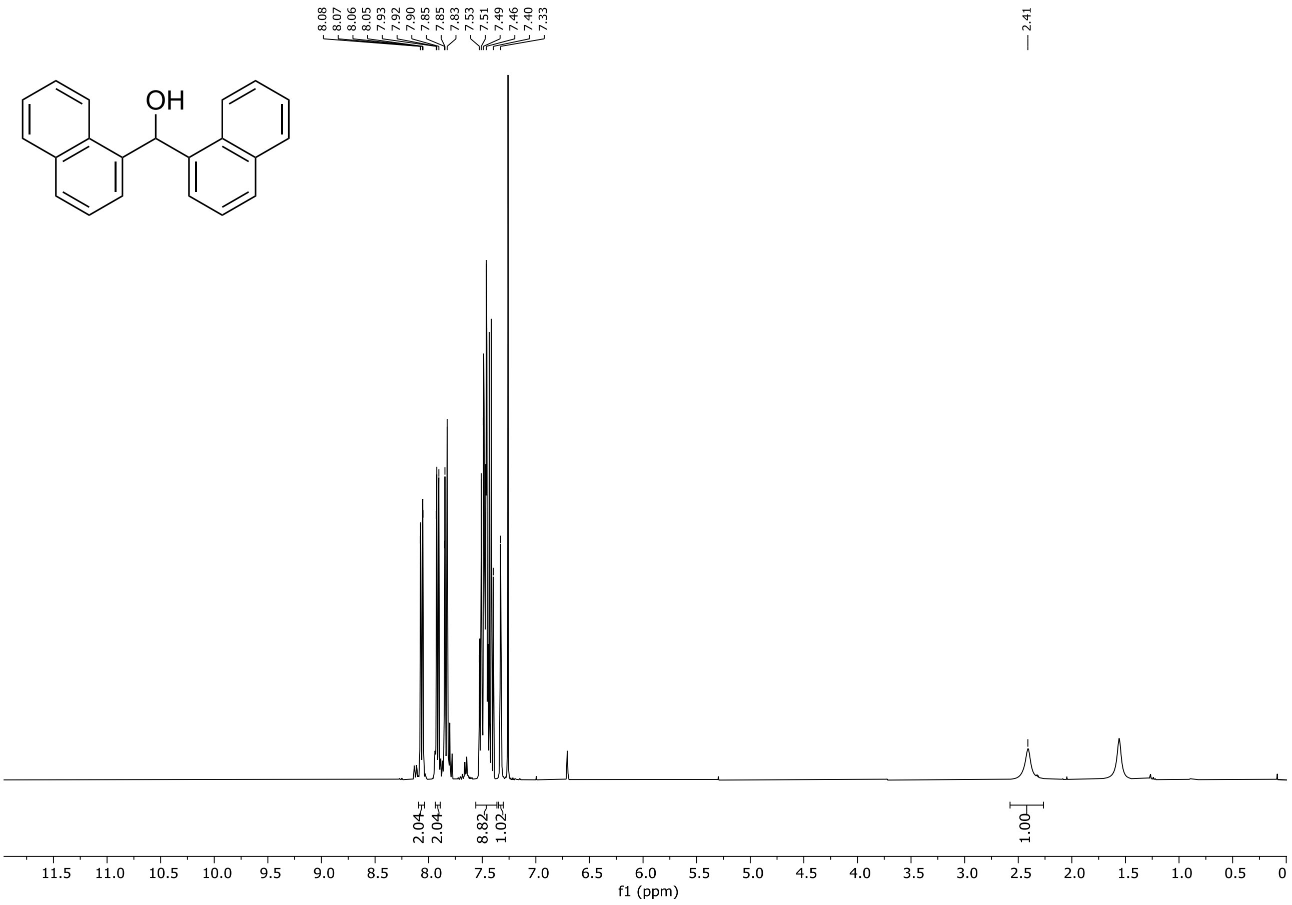

Di(naphthalen-1-yl)methane, ^{1}H NMR (400 MHz, $CDCl_3$)

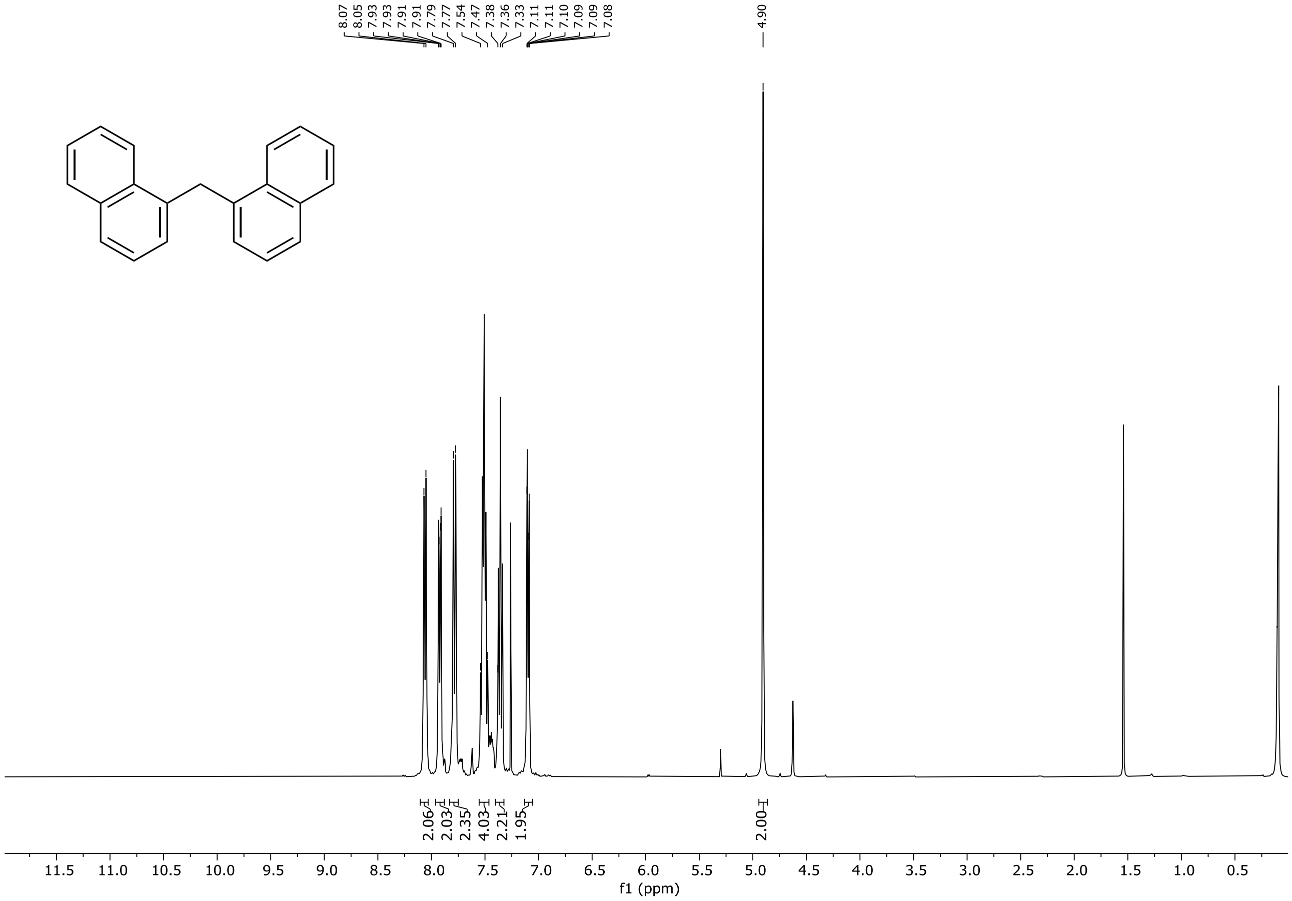

2,2-di(naphthalen-1-yl)acetic acid, ^{1}H NMR (400 MHz, $CDCl_3$)

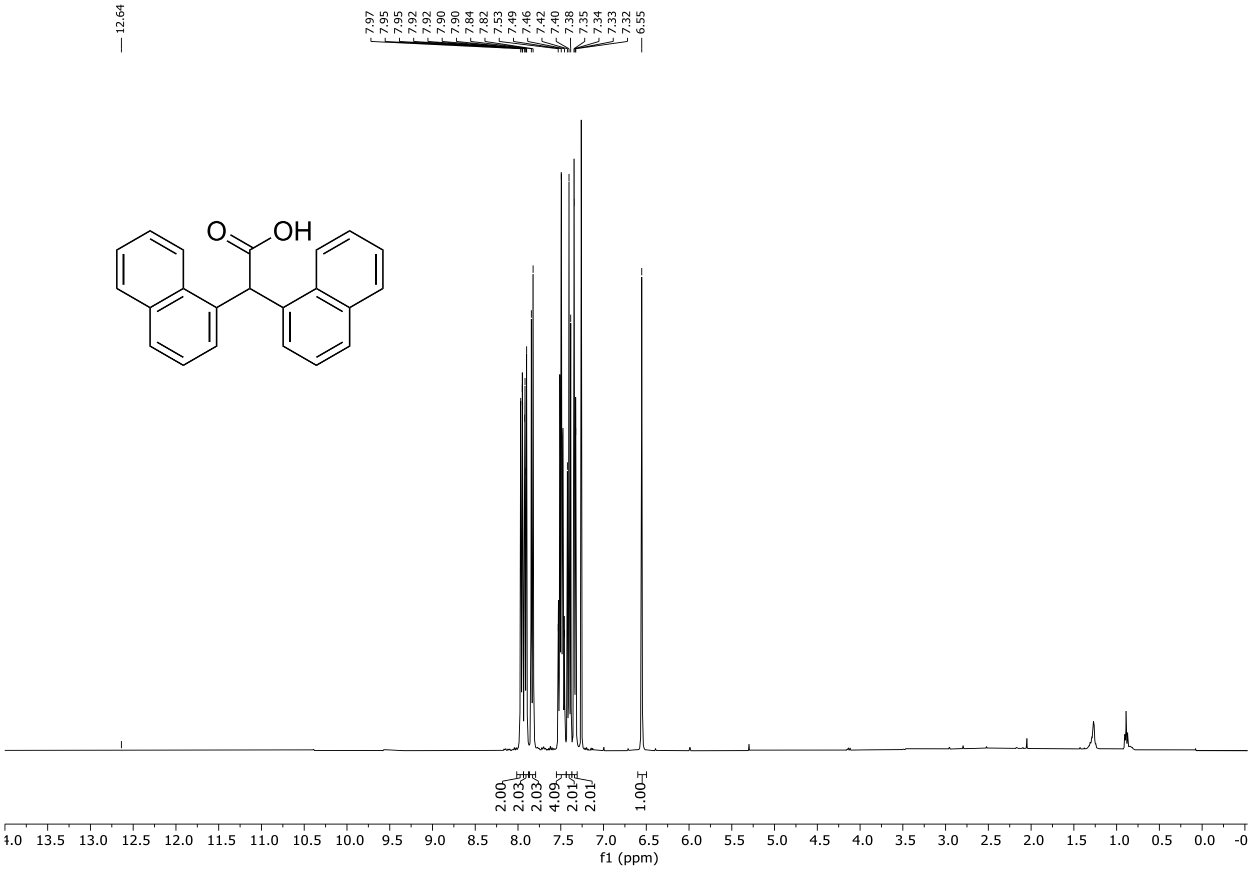

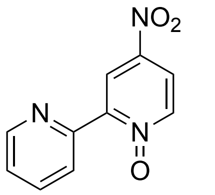

4-nitro-[2,2'-bipyridine] 1-oxide, ^{1}H NMR (400 MHz, $CDCl_3$)

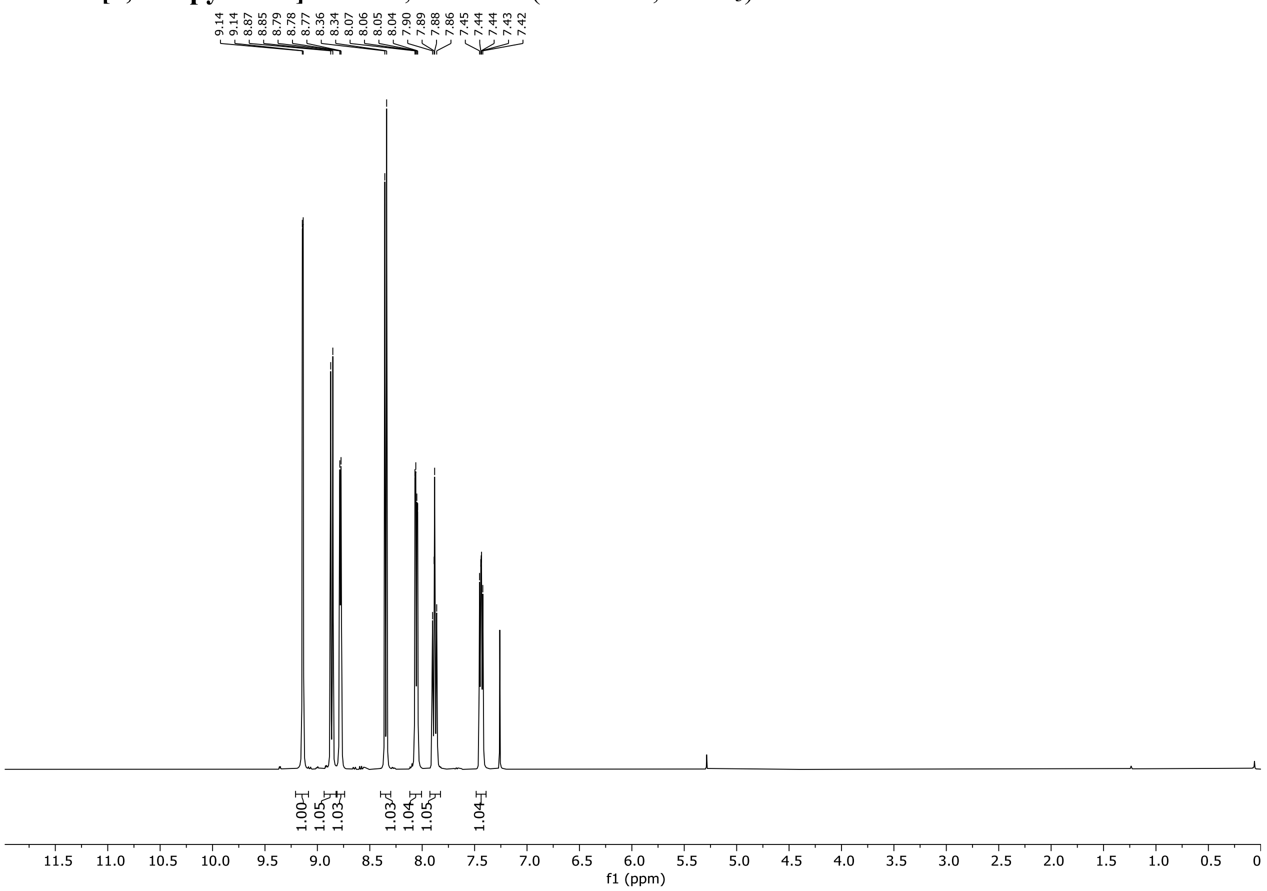

[2,2'-bipyridin]-4-amine L6$_{N^N}$', ^{1}H NMR (400 MHz, $CDCl_3$)

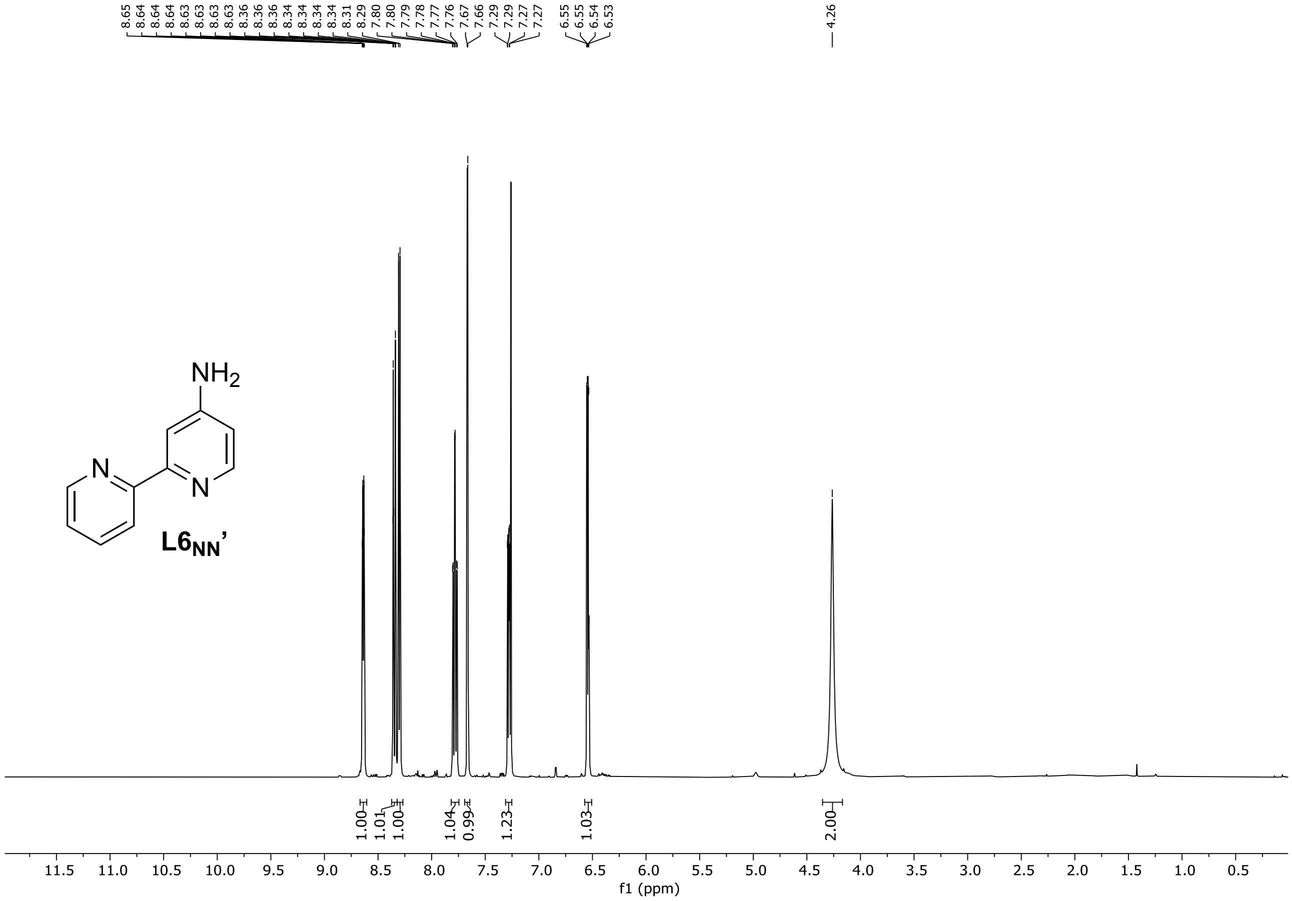

N-([2,2'-bipyridin]-4-yl)-2,2-di(naphthalen-1-yl)acetamide L6$_{N^N}$, ^{1}H NMR (400 MHz, $CDCl_3$)

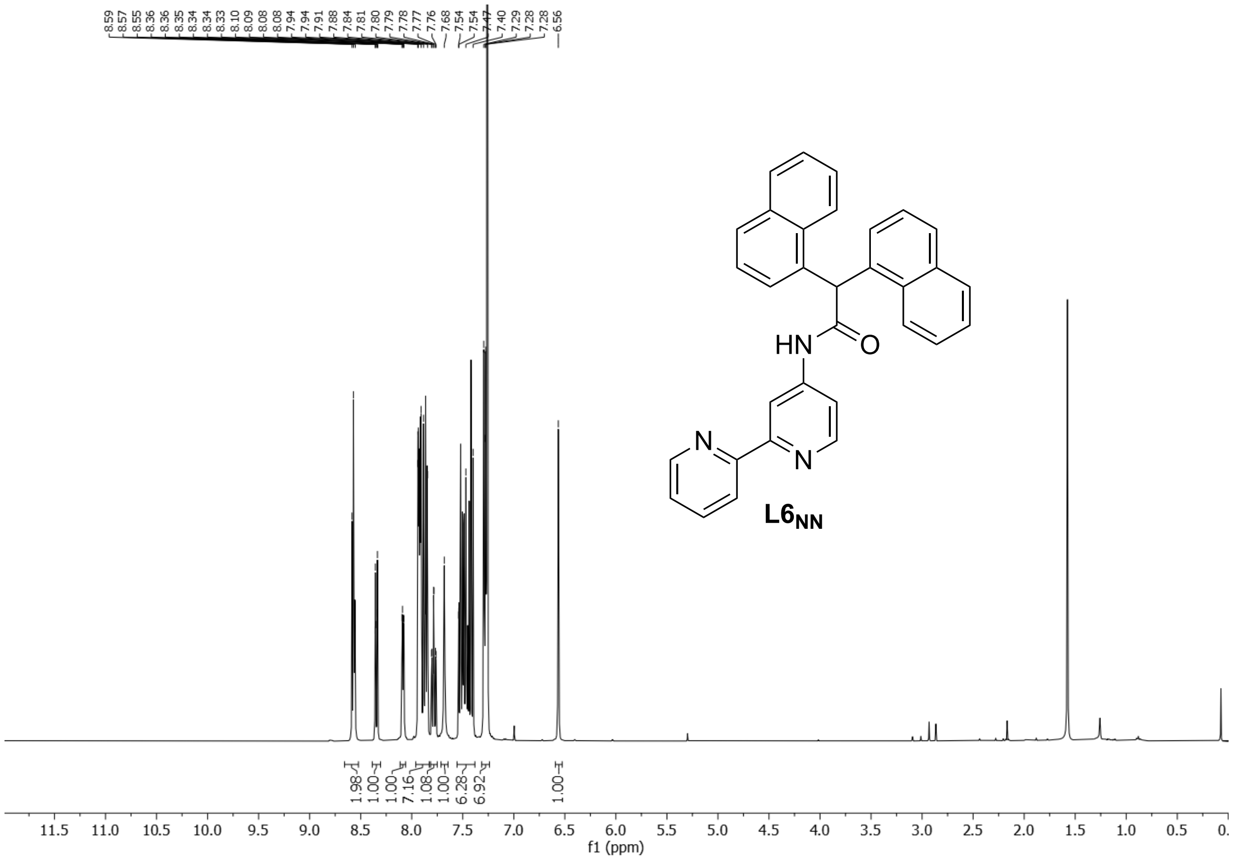

L6$_{N^N}$, ^{13}C NMR (101 MHz, $CDCl_3$)

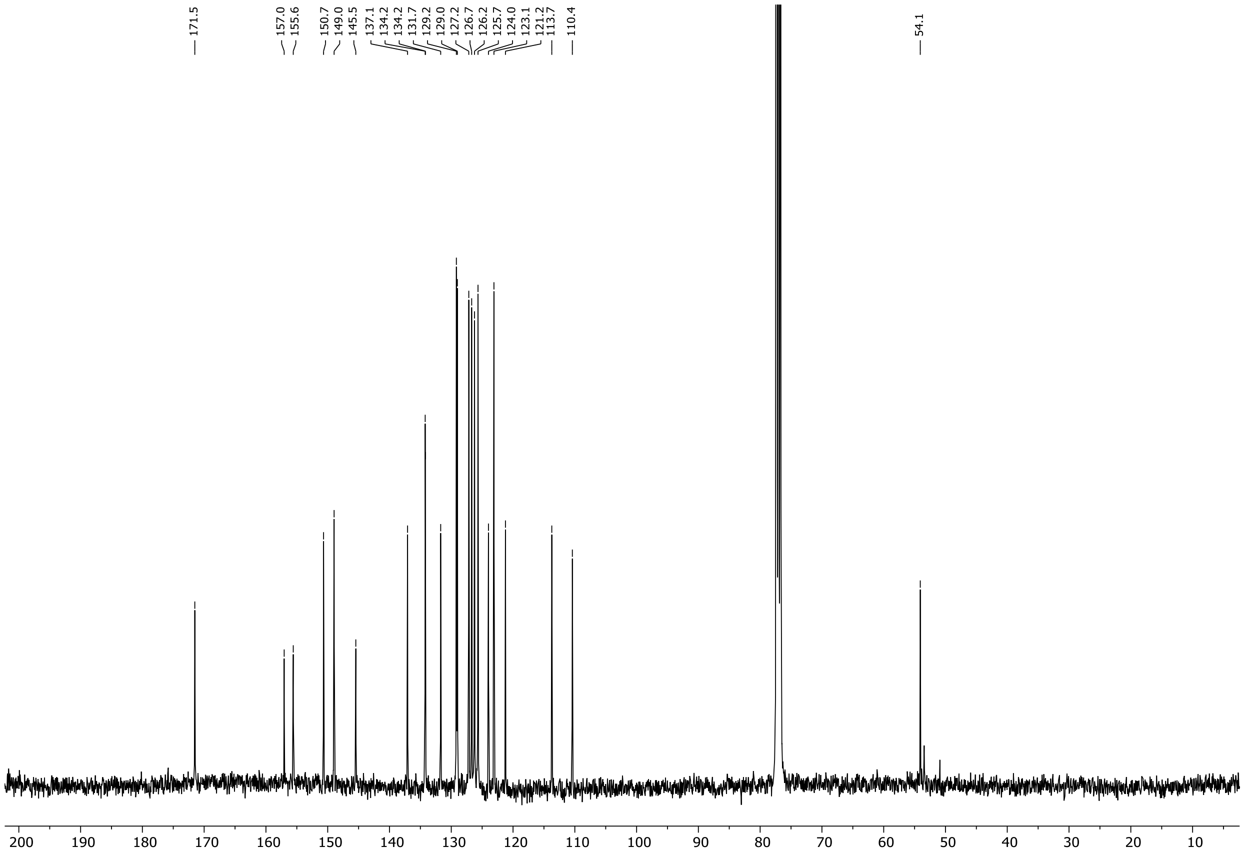

5,5'-dimethyl-[2,2'-bipyridine] 1,1'-dioxide, ^{1}H NMR (400 MHz, $CDCl_3$)

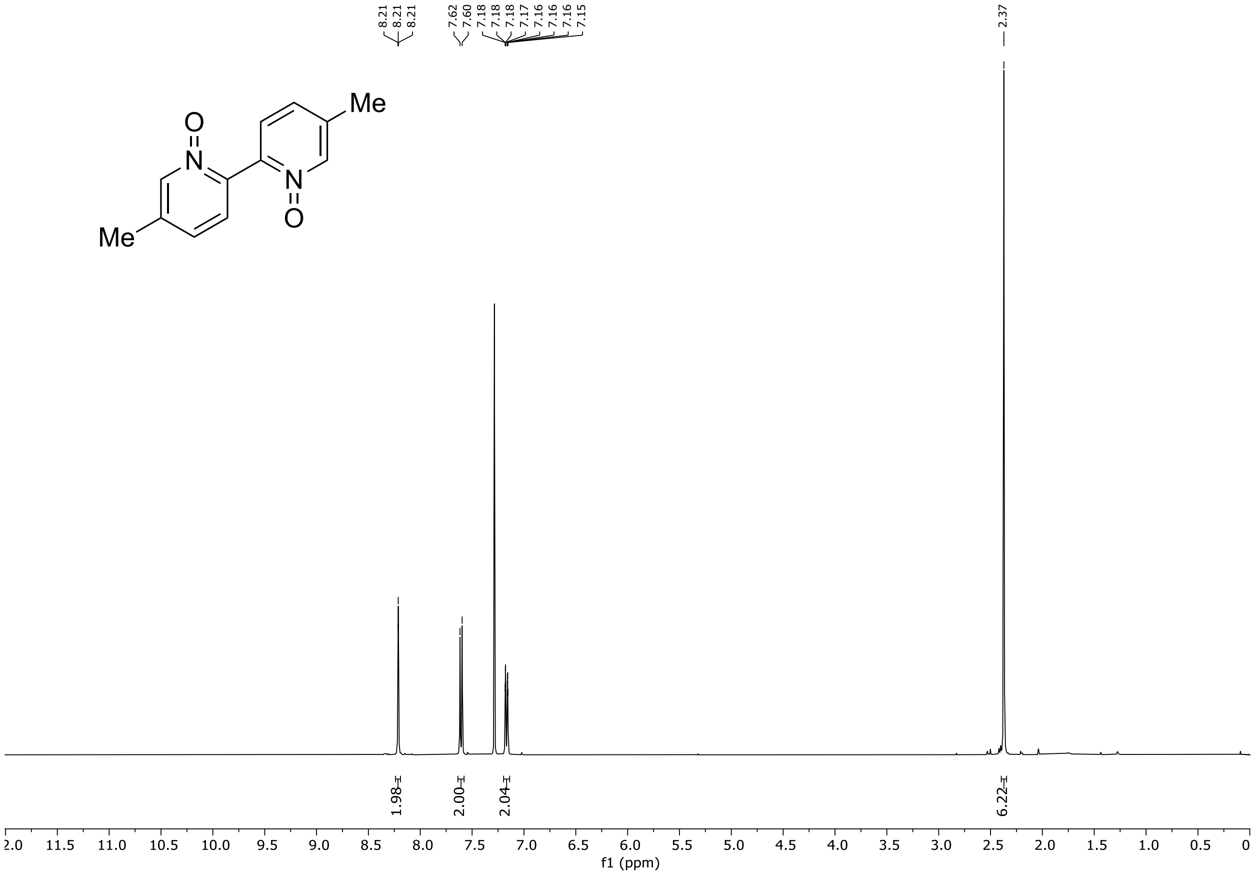

5,5'-dimethyl-[2,2'-bipyridine] 1,1'-dioxide, ^{13}C NMR (101 MHz, $CDCl_3$)

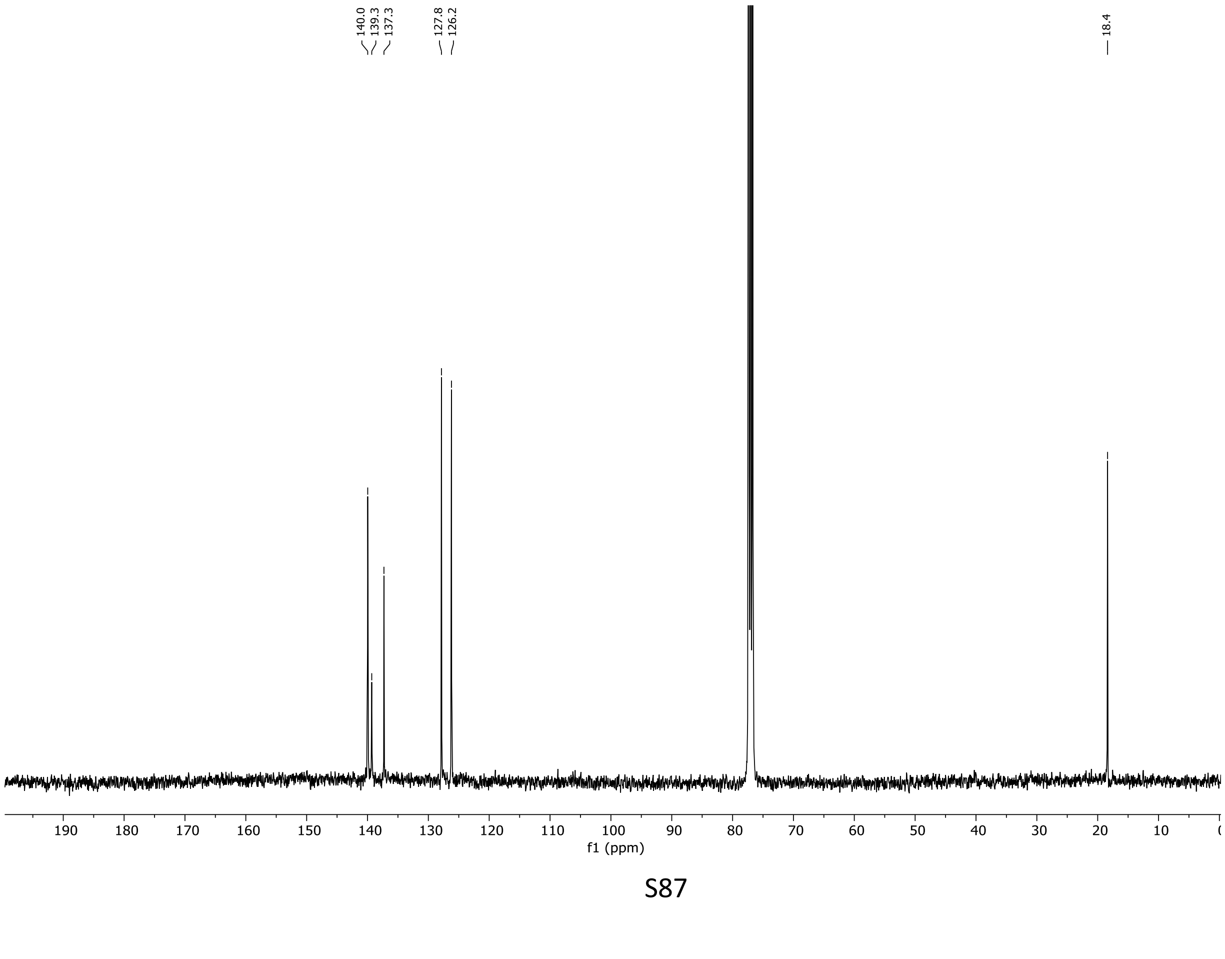

5,5'-dimethyl-4,4'-dinitro-[2,2'-bipyridine] 1,1'-dioxide, ^{1}H NMR (400 MHz, DMSO-d_6)

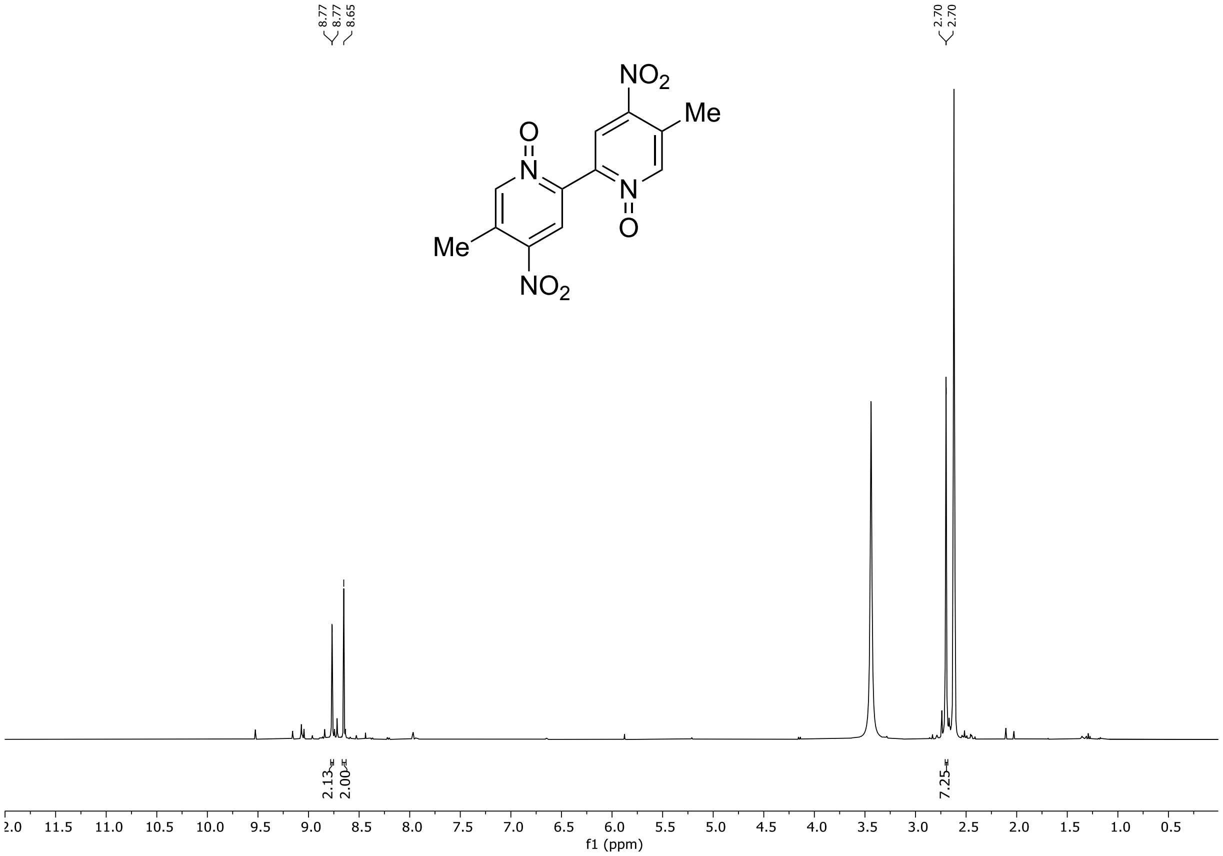

5,5'-dimethyl-[2,2'-bipyridine]-4,4'-diamine 1f’, ^{1}H NMR (400 MHz, DMSO-d_6)

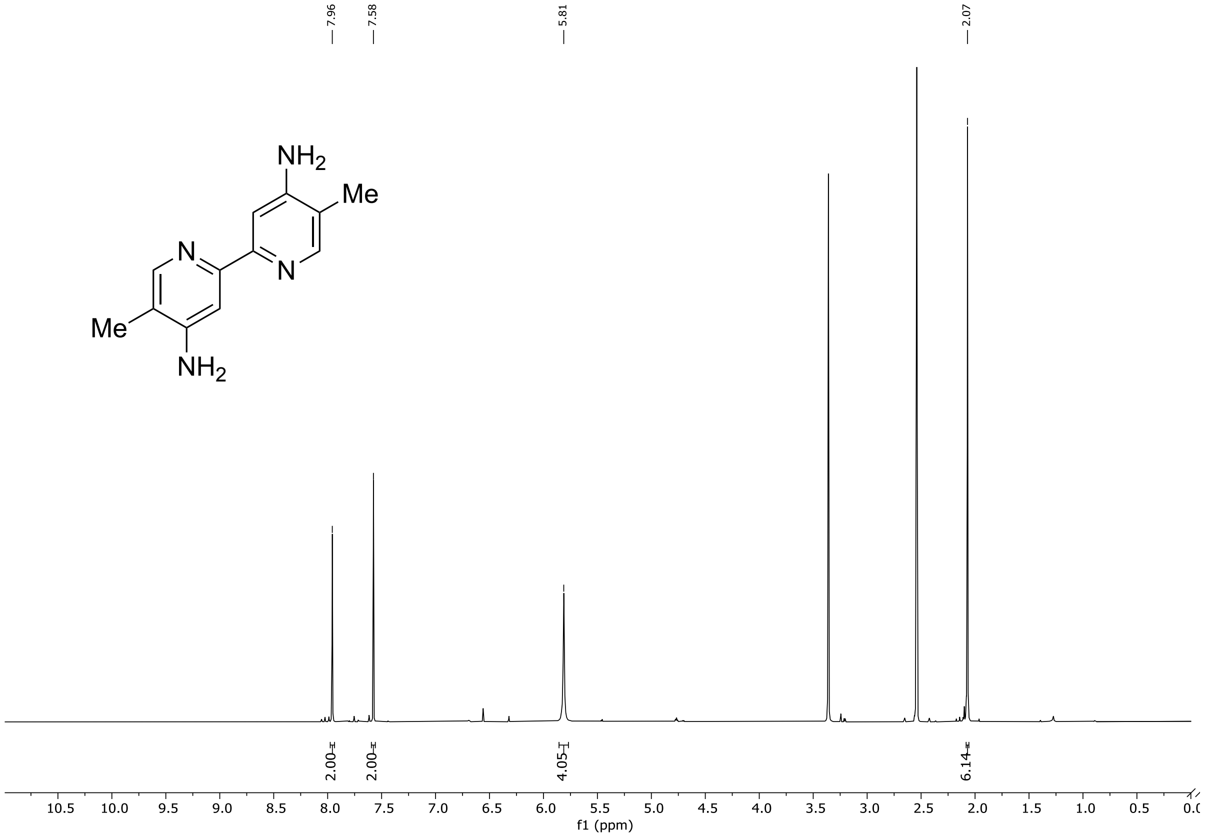

5,5'-dimethyl-[2,2'-bipyridine]-4,4'-diamine 1f’, ^{1}H NMR (400 MHz, DMSO-d_6)

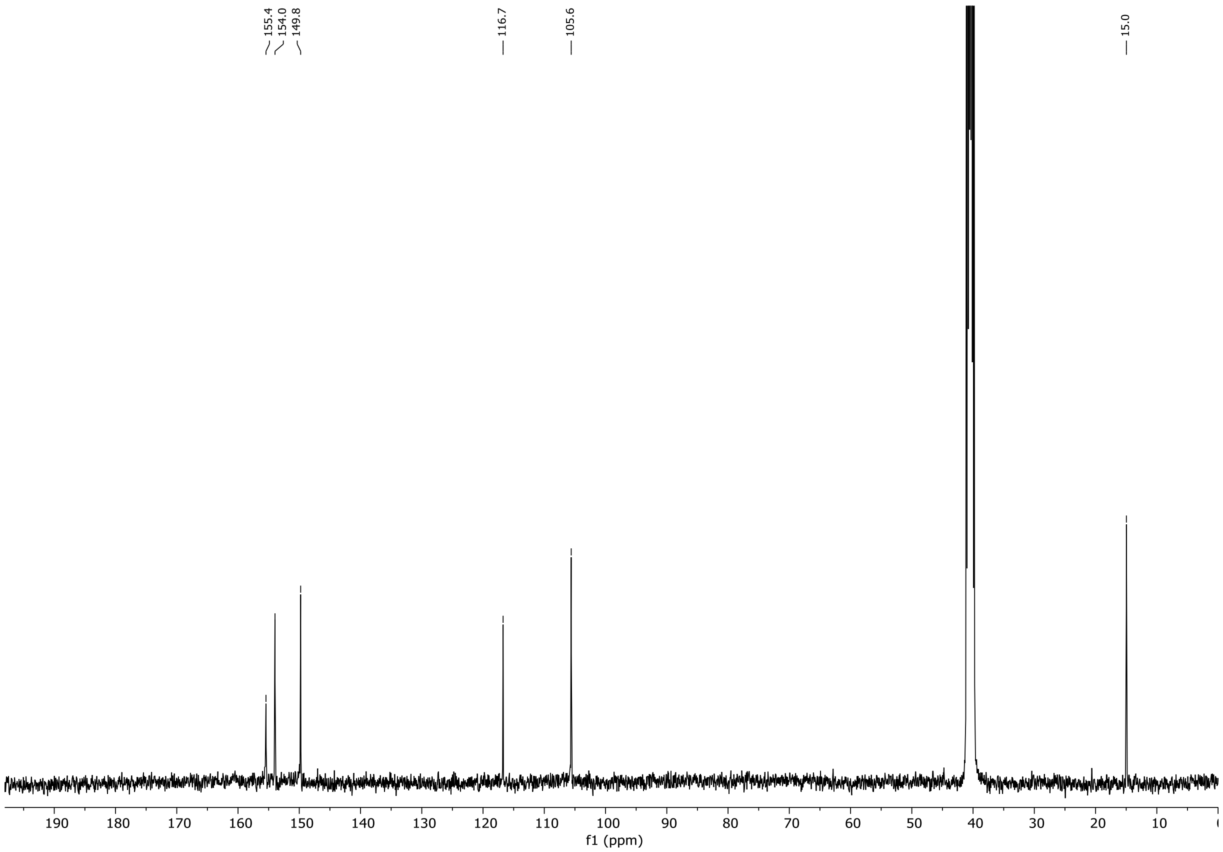

5-**(bromomethyl)-5'-methyl-2,2'-bipyridine (crude product),** ^{1}HNMR (400MHz, $CDCl_3$)

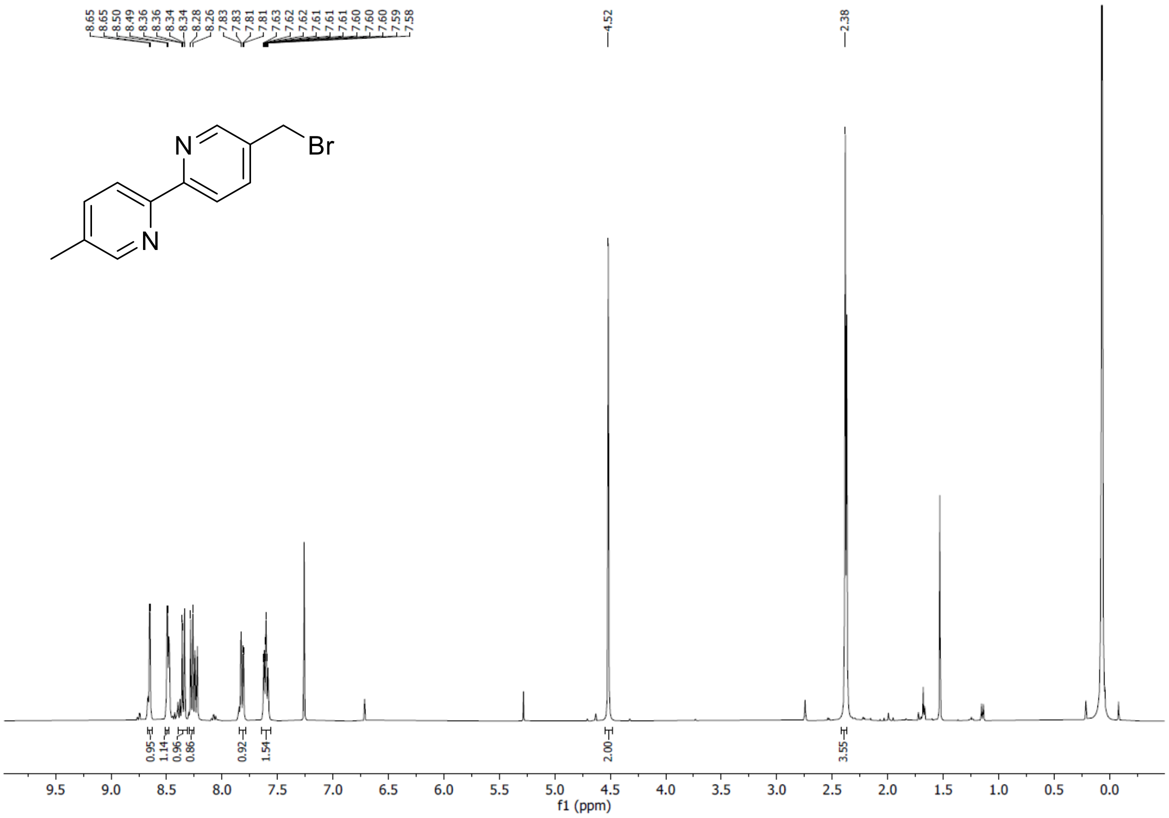

(5'-**methyl-[2,2'-bipyridin]-5-yl)methanamine**, ^{1}HNMR (400MHz, $CDCl_3$)

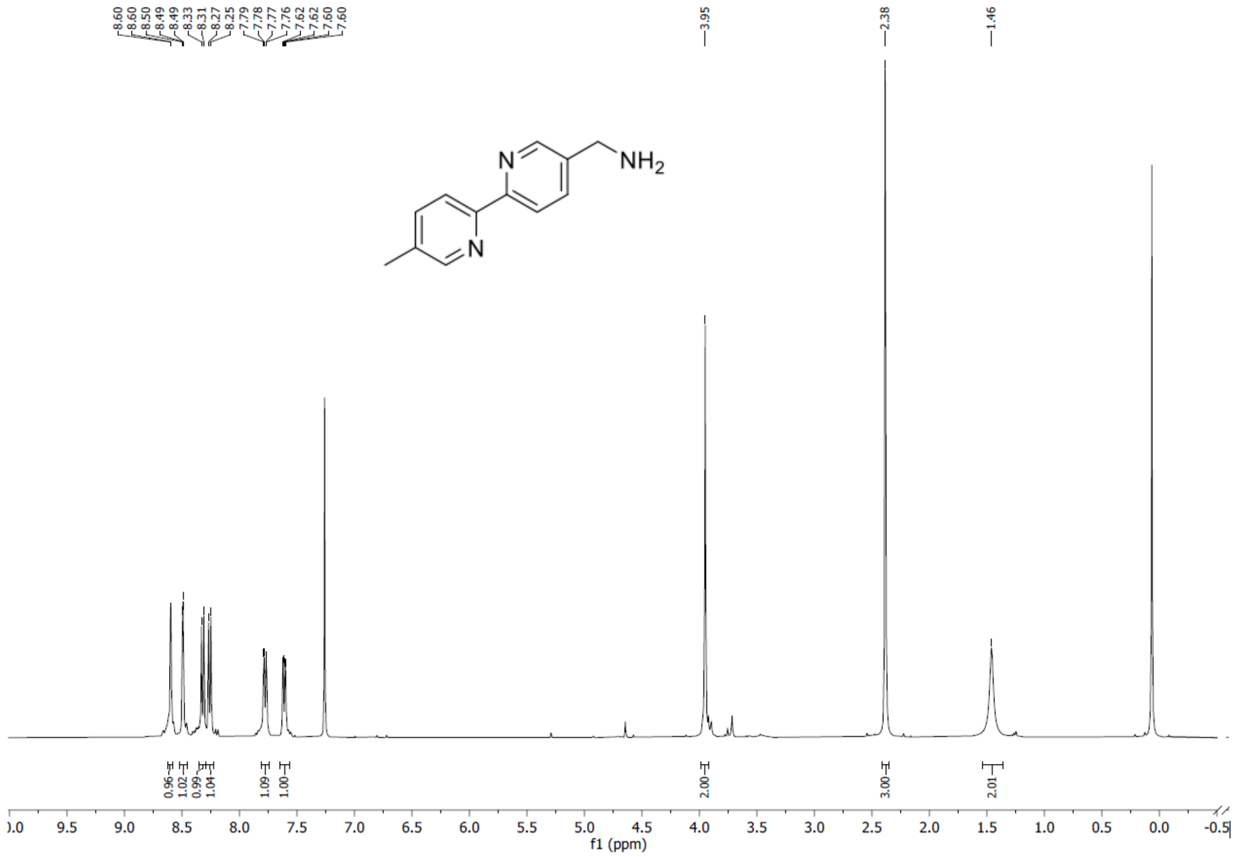

(5'-methyl-[2,2'-bipyridin]-5-yl)methanamine, ^{13}CNMR (101MHz, $CDCl_3$)

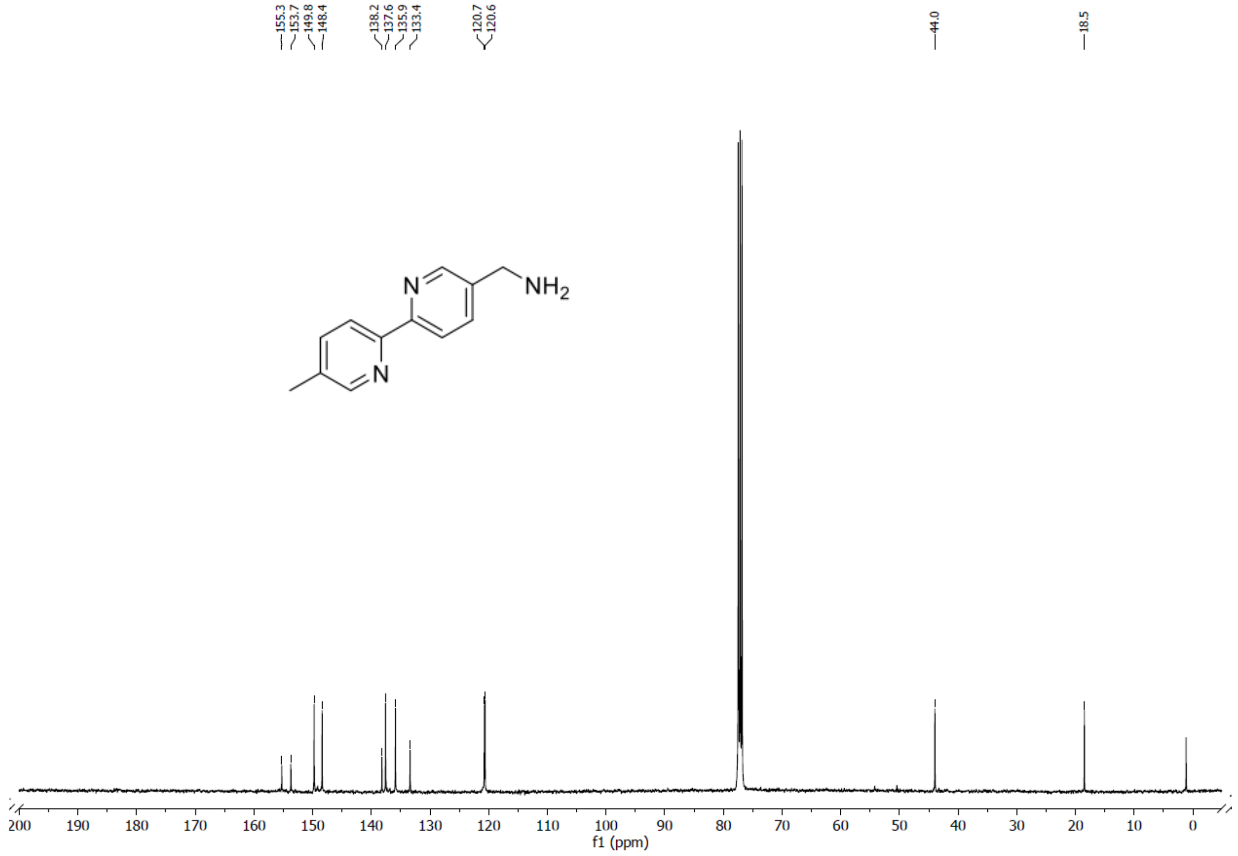

***N*-((5'-methyl-[2,2'-bipyridin]-5-yl)methyl)-2,2-di(naphthalen-1-yl)acetamide L8$_{N^\wedge N}$**, ^{1}HNMR (400Mhz, CDCl$_3$)

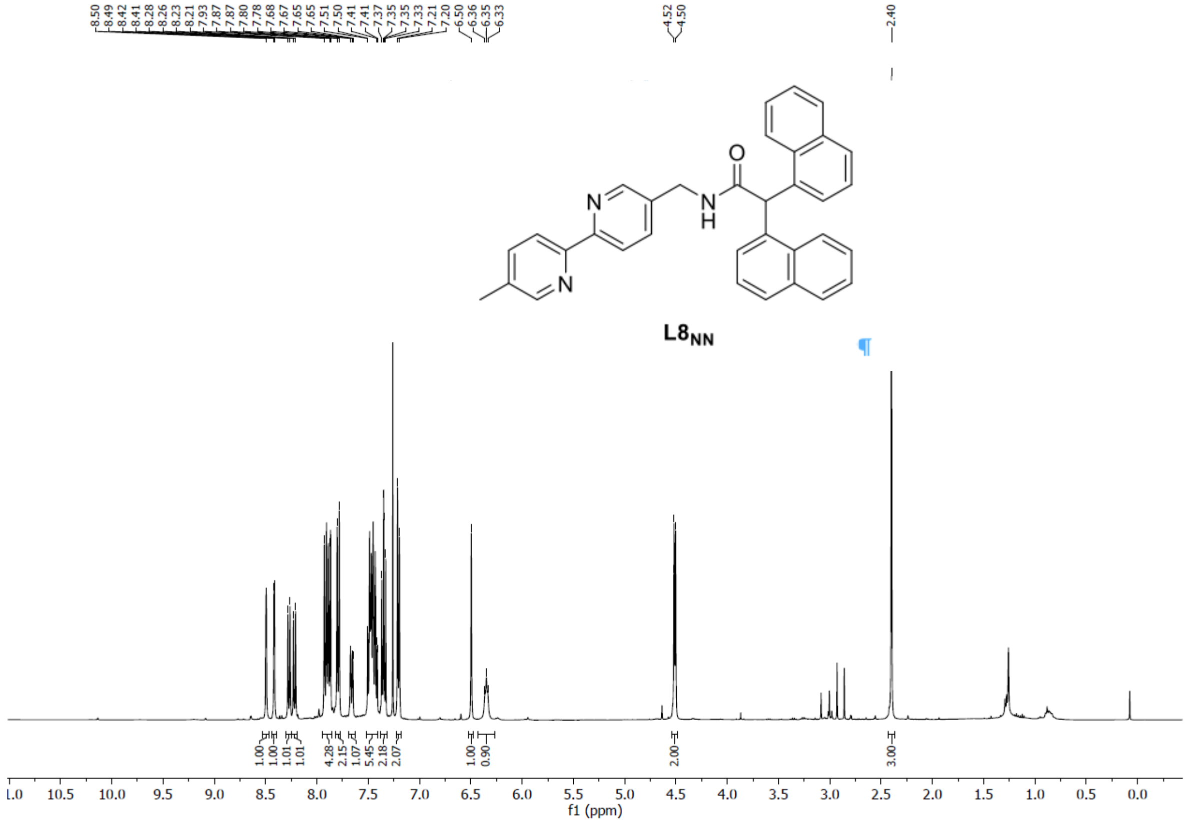

***N*-((5'-methyl-[2,2'-bipyridin]-5-yl)methyl)-2,2-di(naphthalen-1-yl)acetamide L8$_{N^\wedge N}$**, ^{13}CNMR (101MHz, CDCl$_3$)

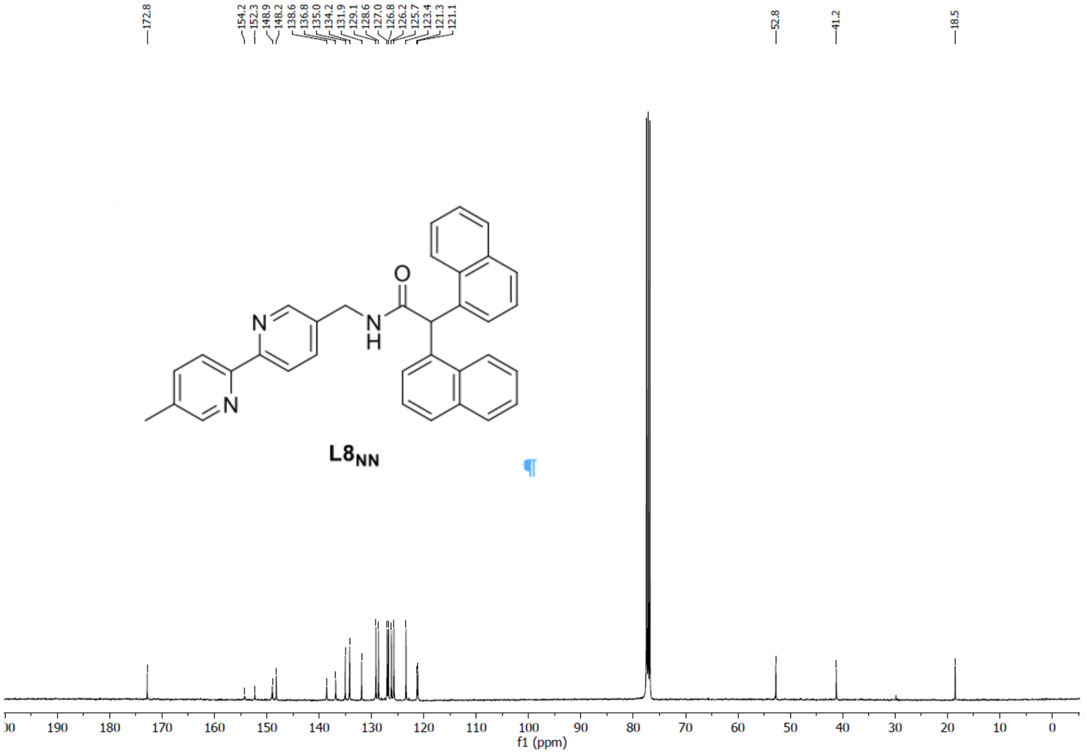

Charatherization of complexes

2a, ^{1}H NMR (400 MHz, $CDCl_3$)

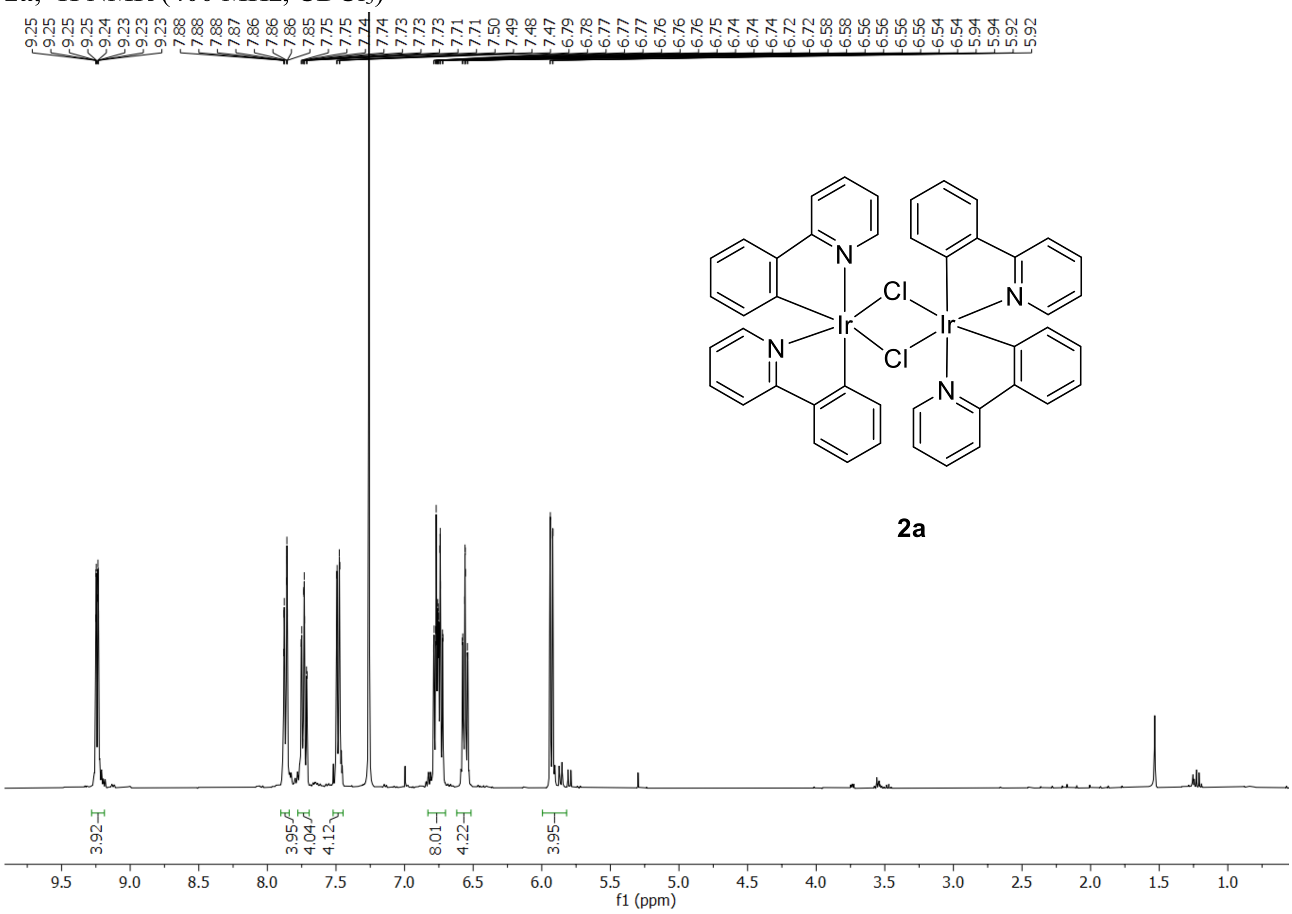

2b (crude), ^{1}H NMR (400 MHz, $CDCl_3$)

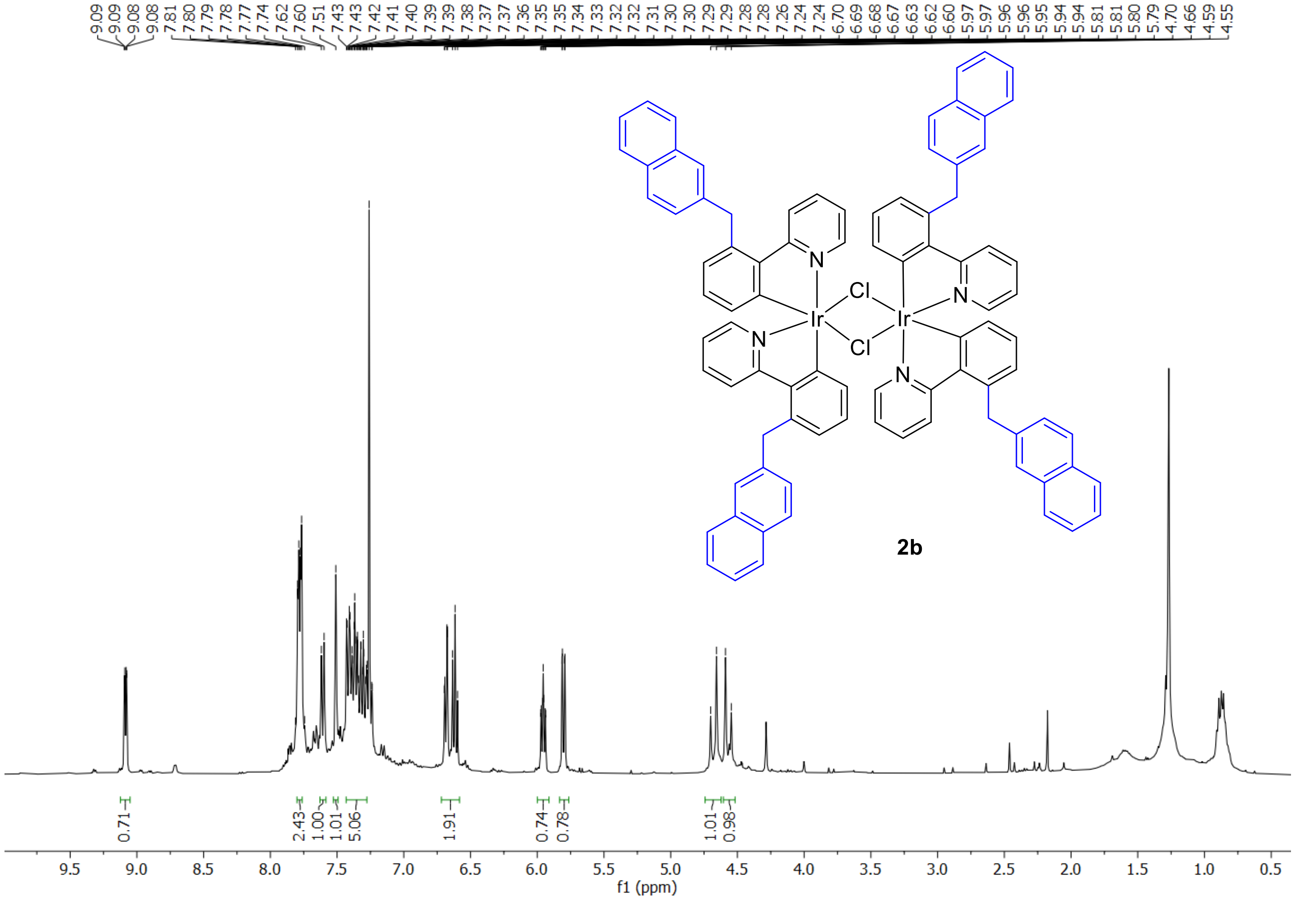

2b (crude), ^{13}C NMR (101 MHz, $CDCl_3$)

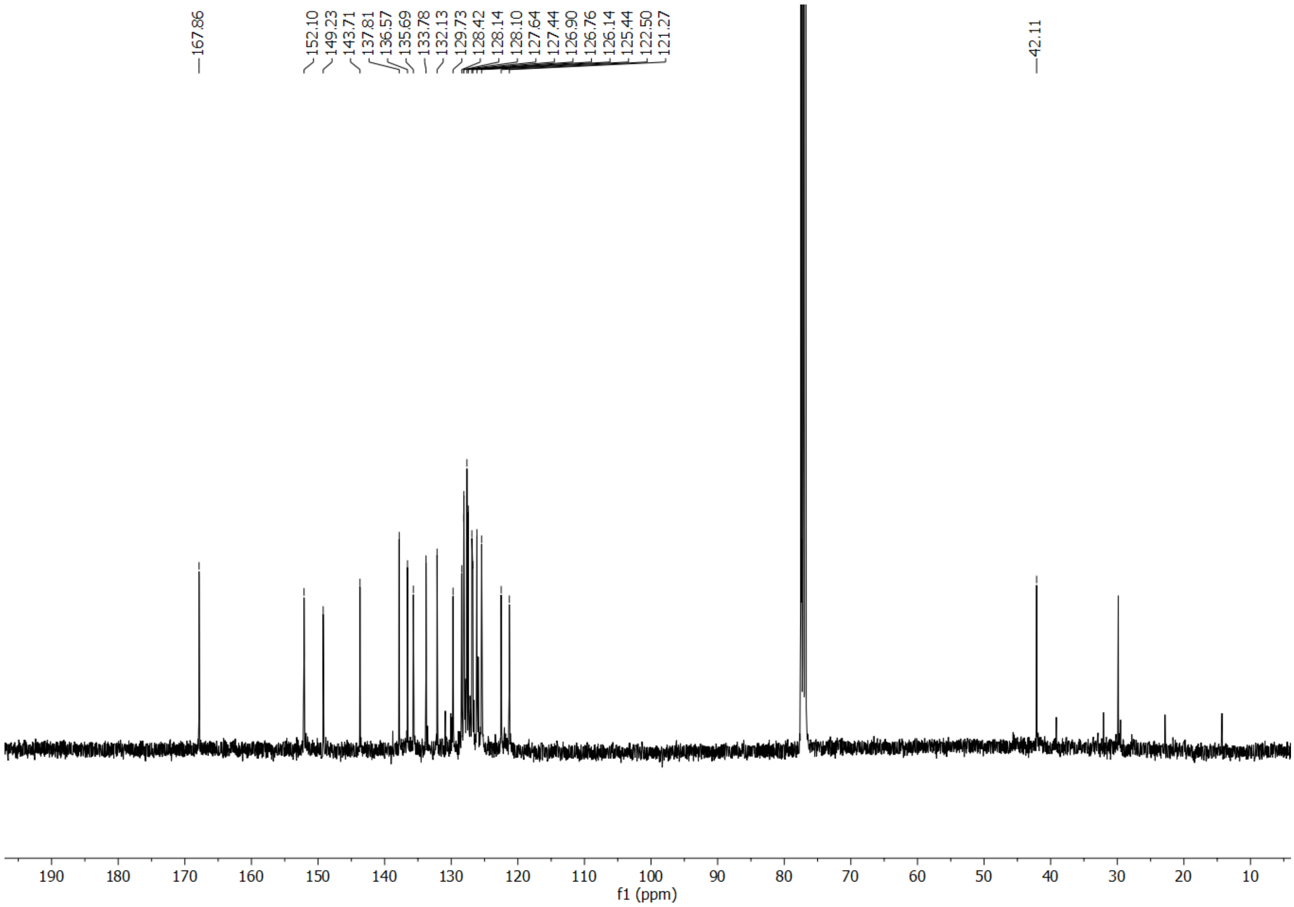

[Ir(µCl)(dF(CF$_3$)ppy)$_2$]$_2$, 2d, ^{1}H NMR (600 MHz, $CDCl_3$)

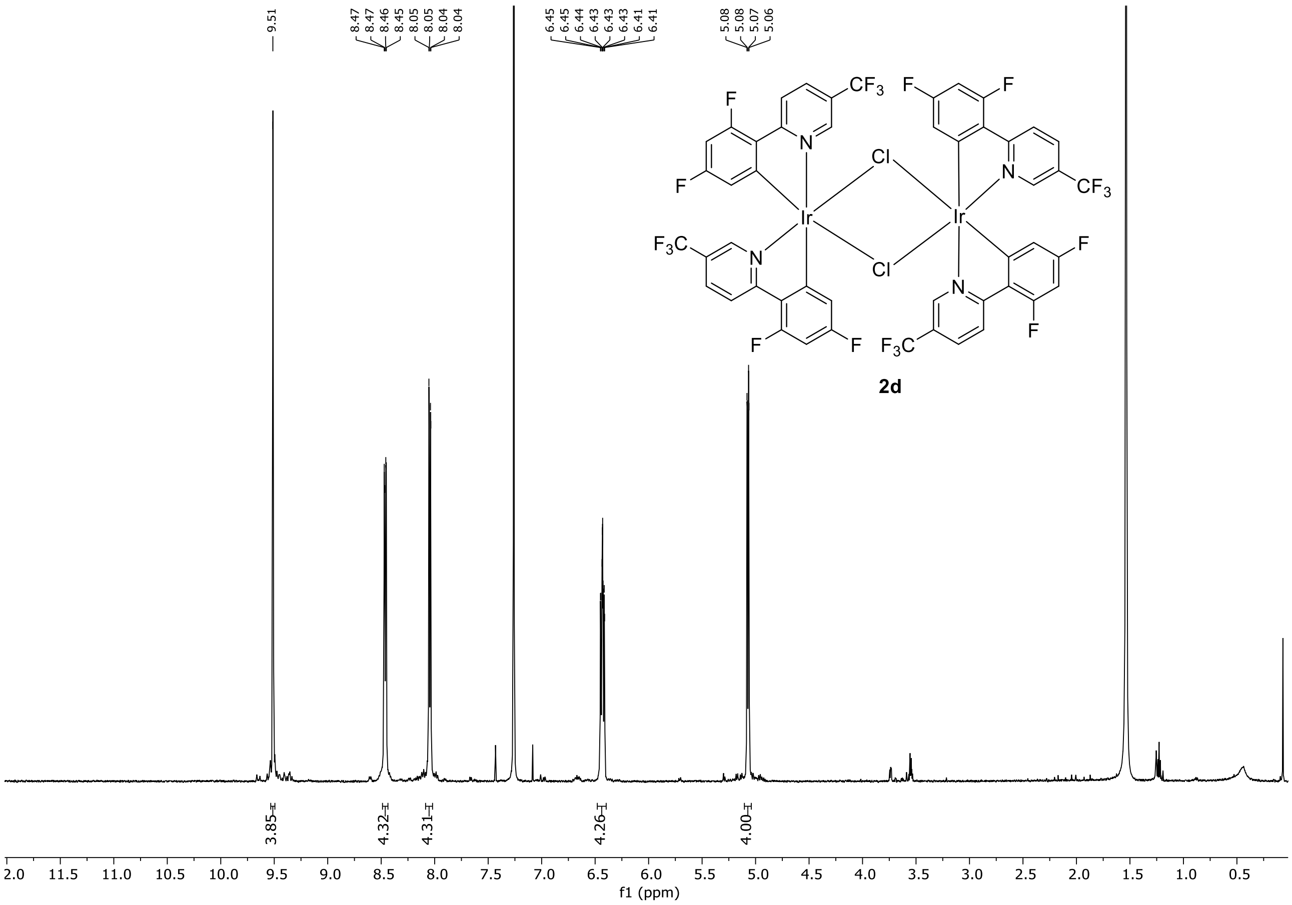

Ir(ppy)(bpy)BF$_4$, ^{1}H NMR (400 MHz, *Acetone-d$_6$*)

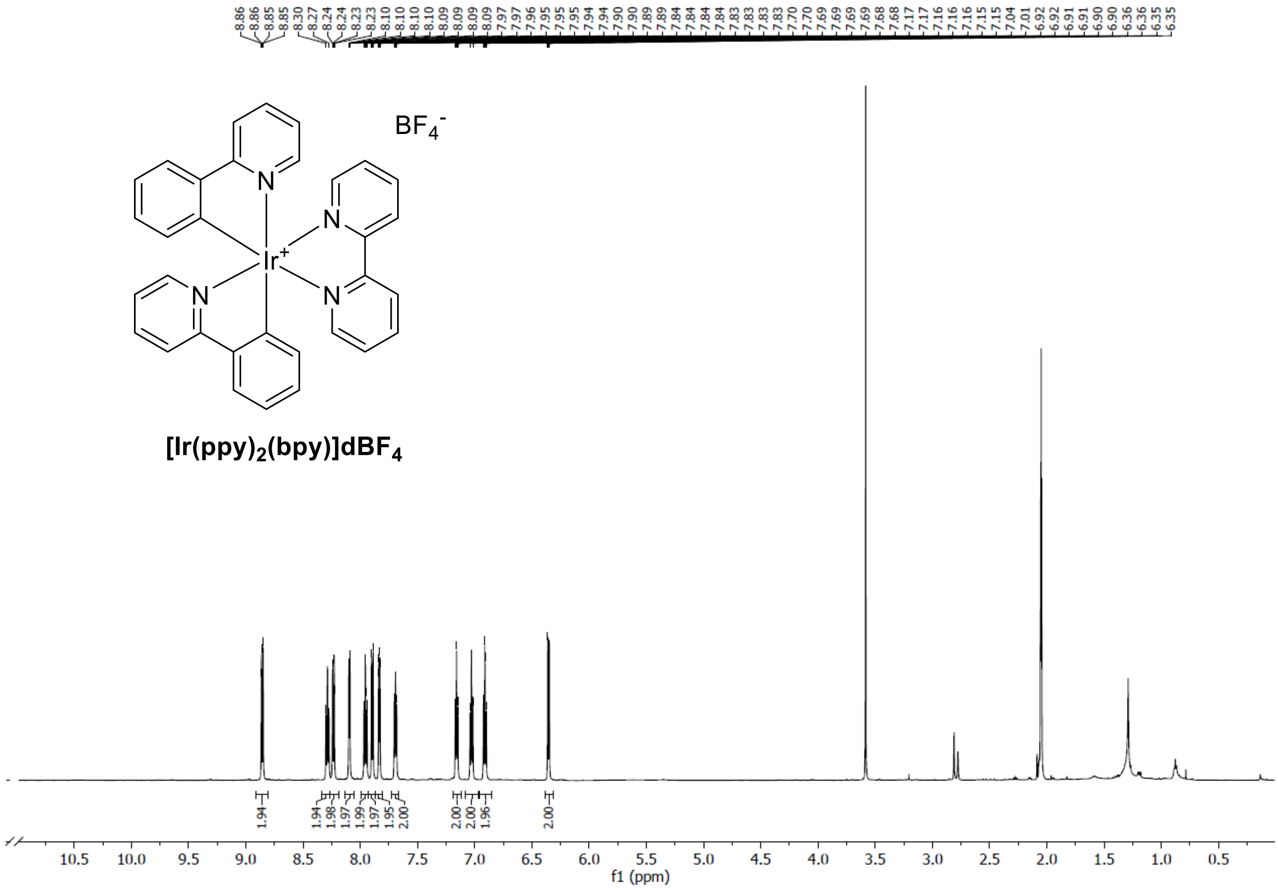

PC1, ^{1}H NMR (400 MHz, Acetone-d_6)

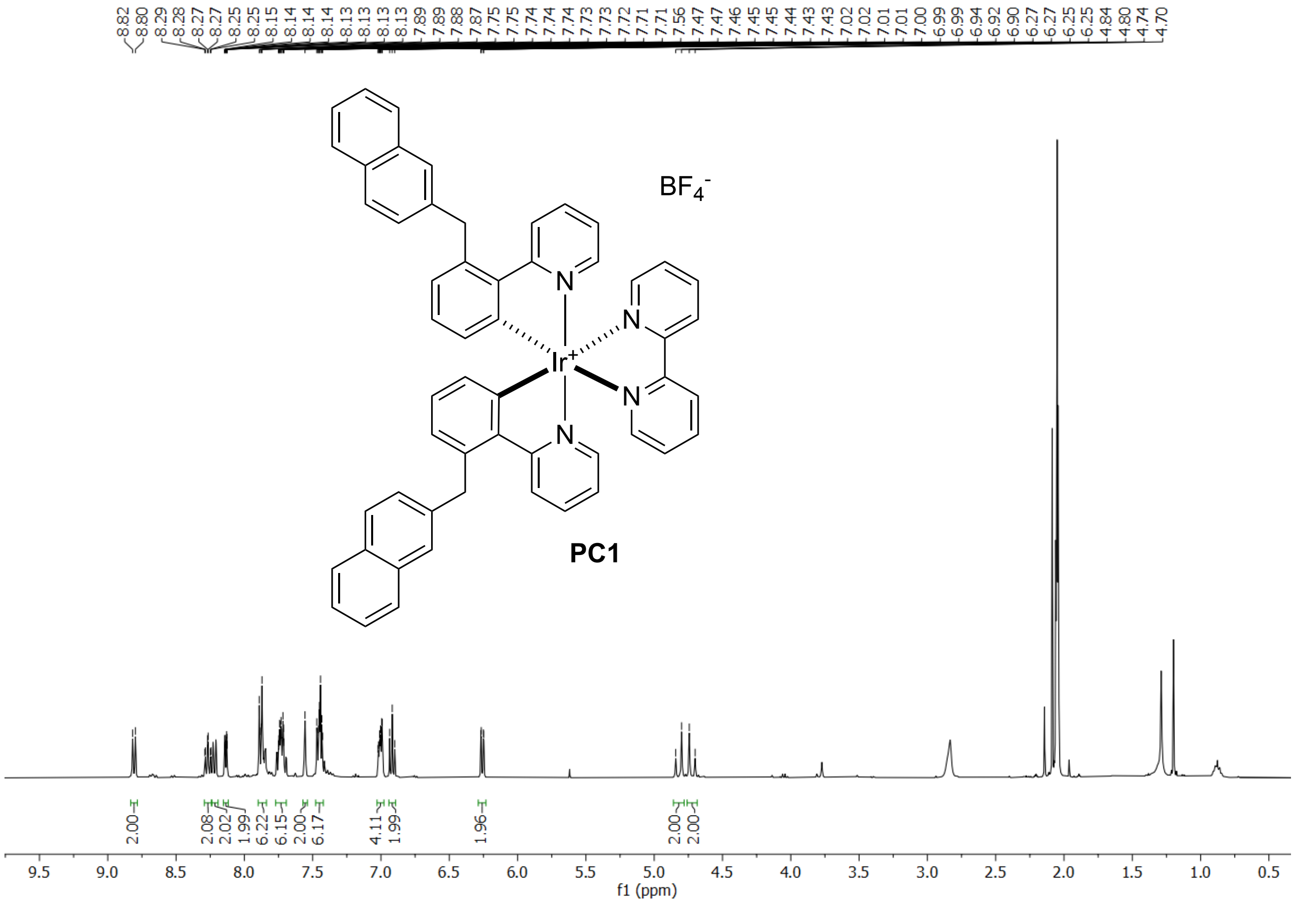

PC1, ^{13}C NMR (101 MHz, Acetone-d_6)

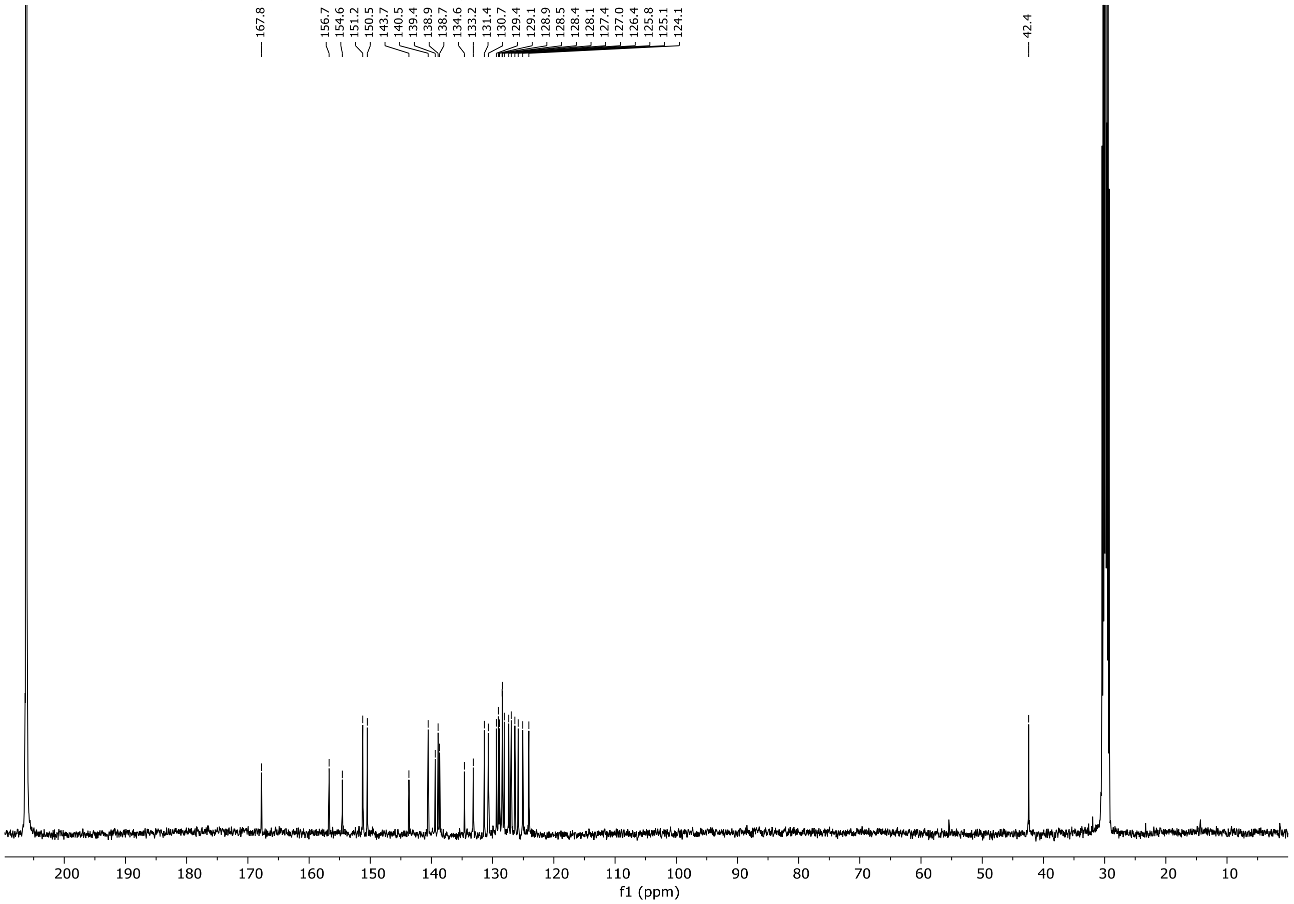

PC2, ^{1}H NMR (400 MHz, Acetone-d_6)

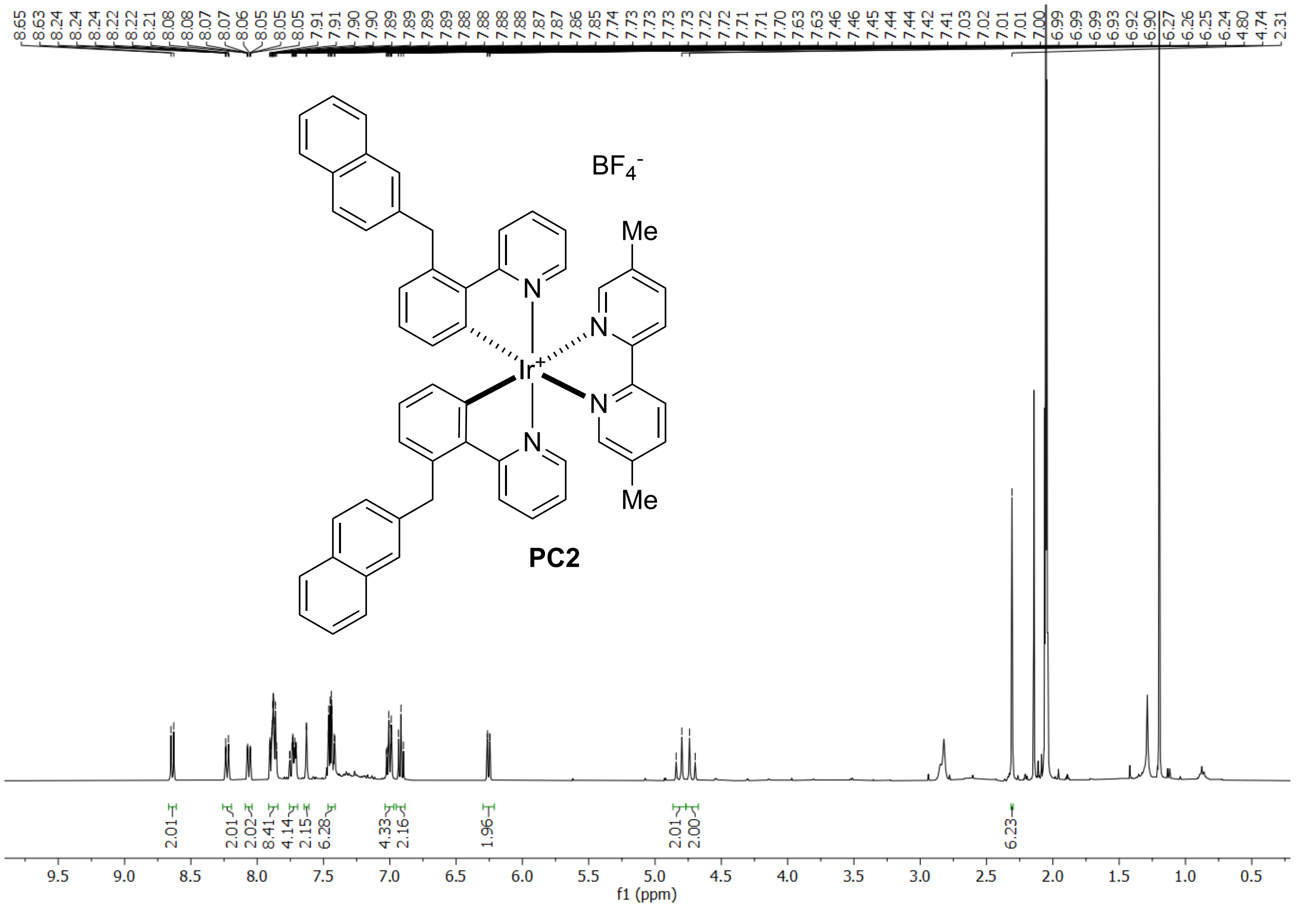

PC2, ^{13}C NMR (101 MHz, Acetone-d_6)

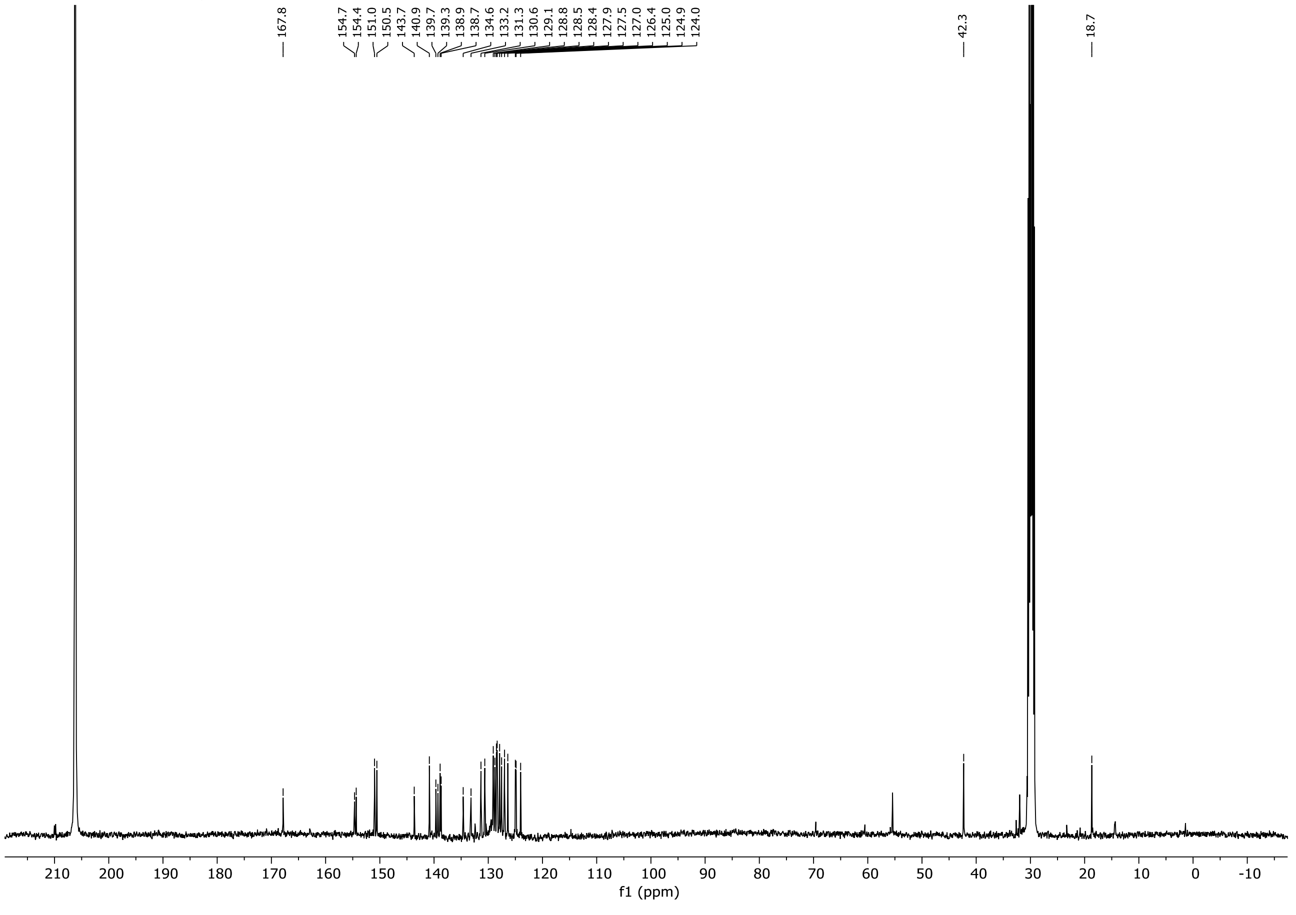

PC3, ^{1}H NMR (400 MHz, Acetone-d_6)

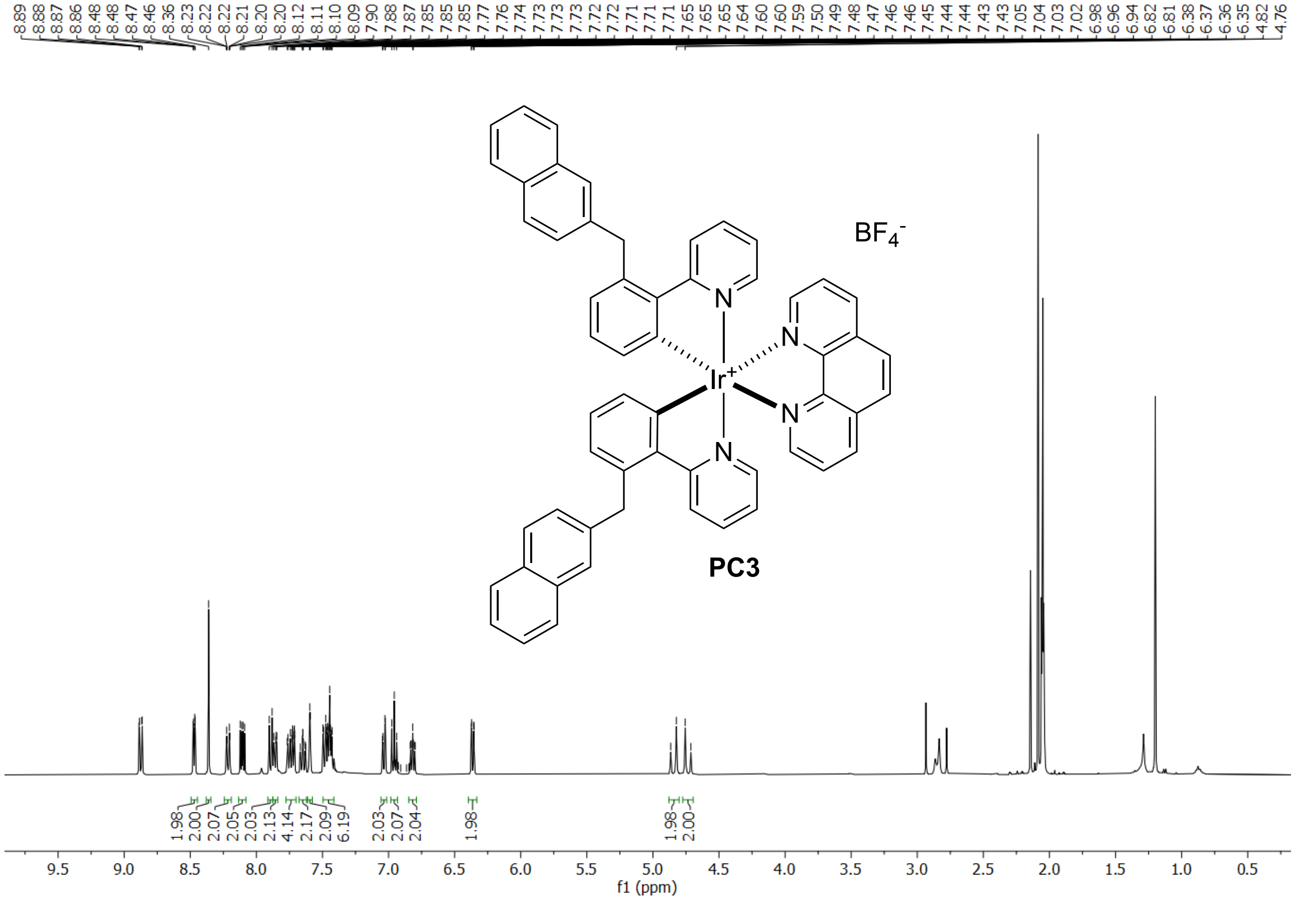

PC3, ^{13}C NMR (101 MHz, Acetone-d_6)

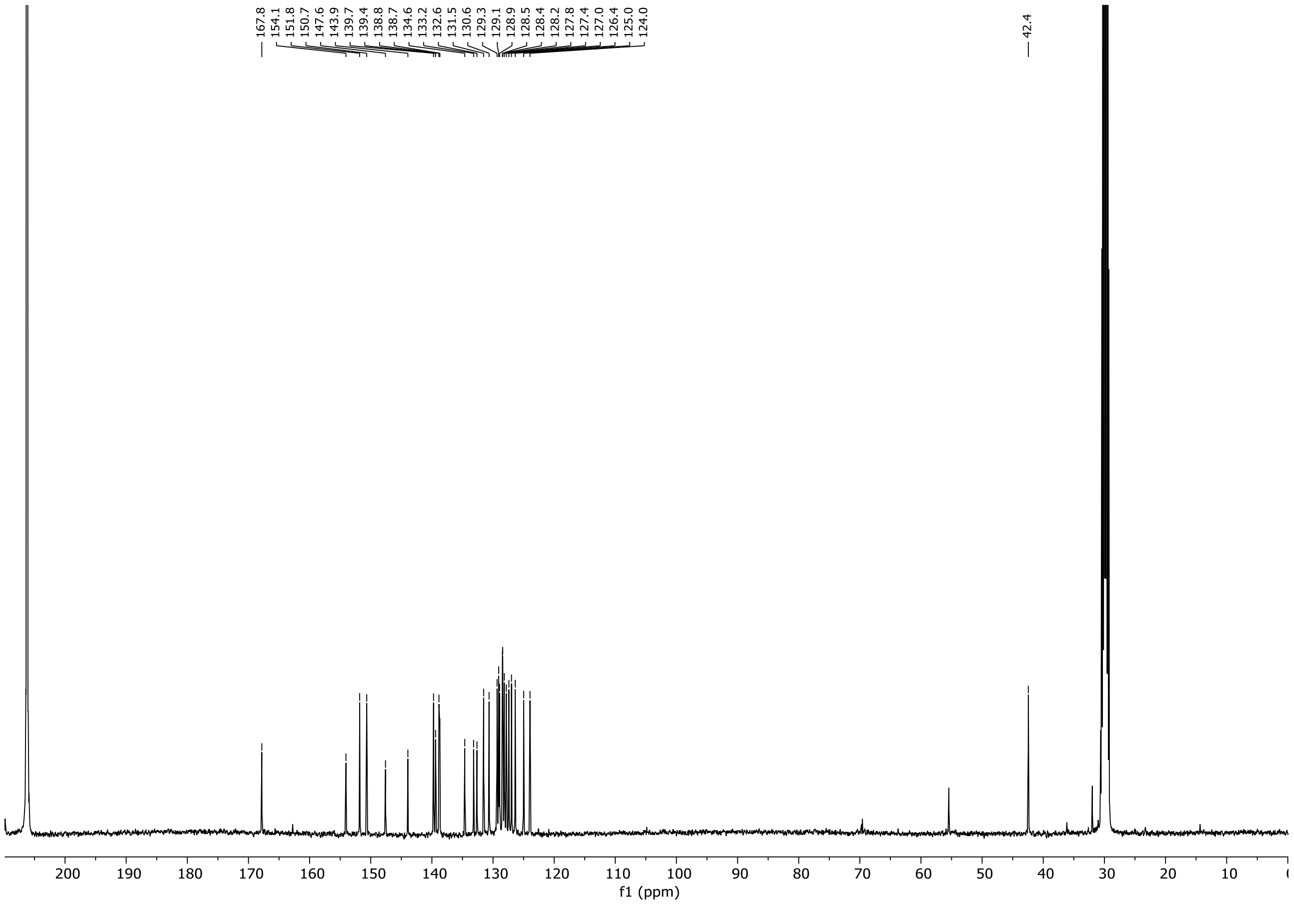

PC4, ^{1}H NMR (400 MHz, Acetone-d_6)

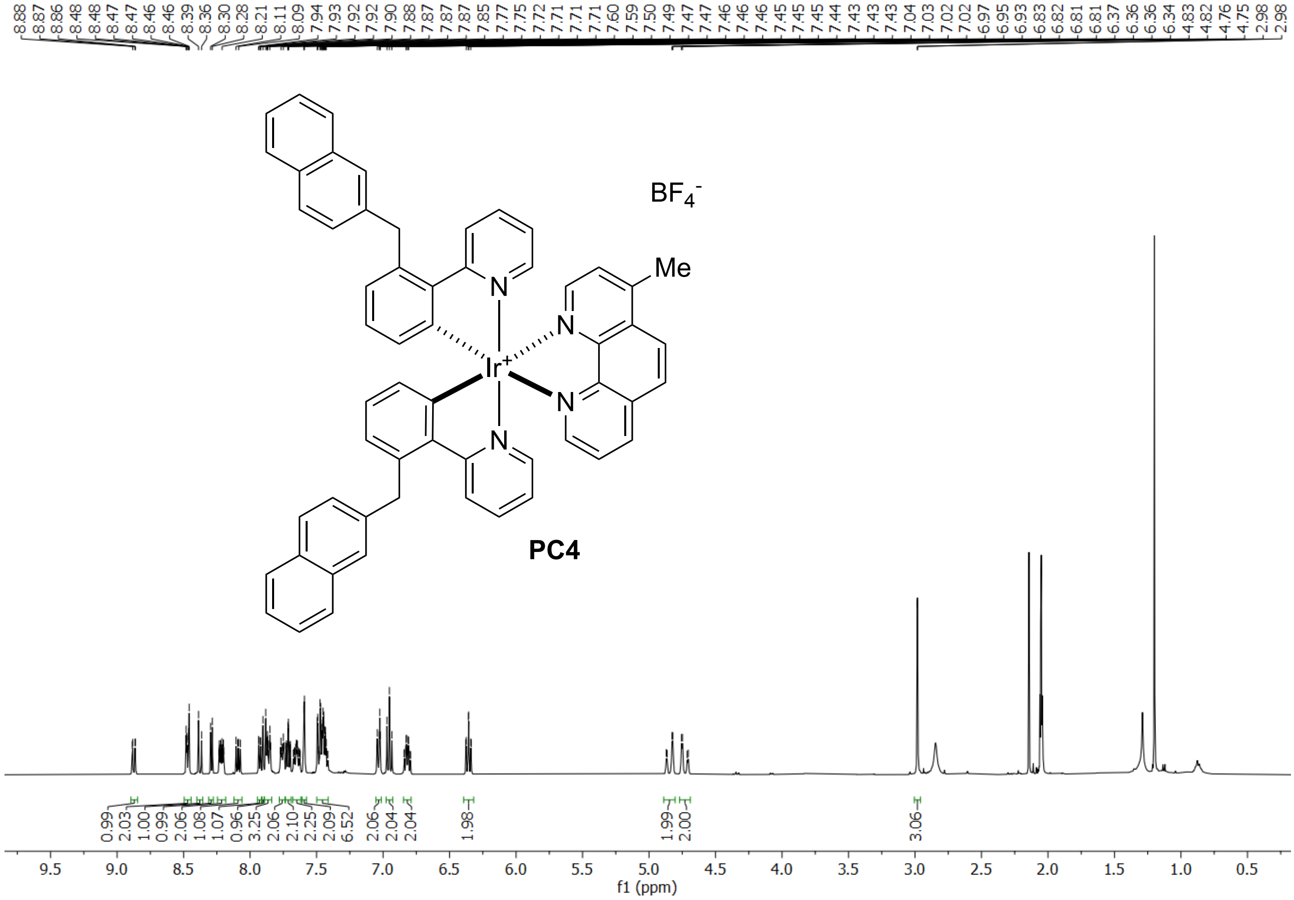

PC4, ^{13}C NMR (101 MHz, Acetone-d_6)

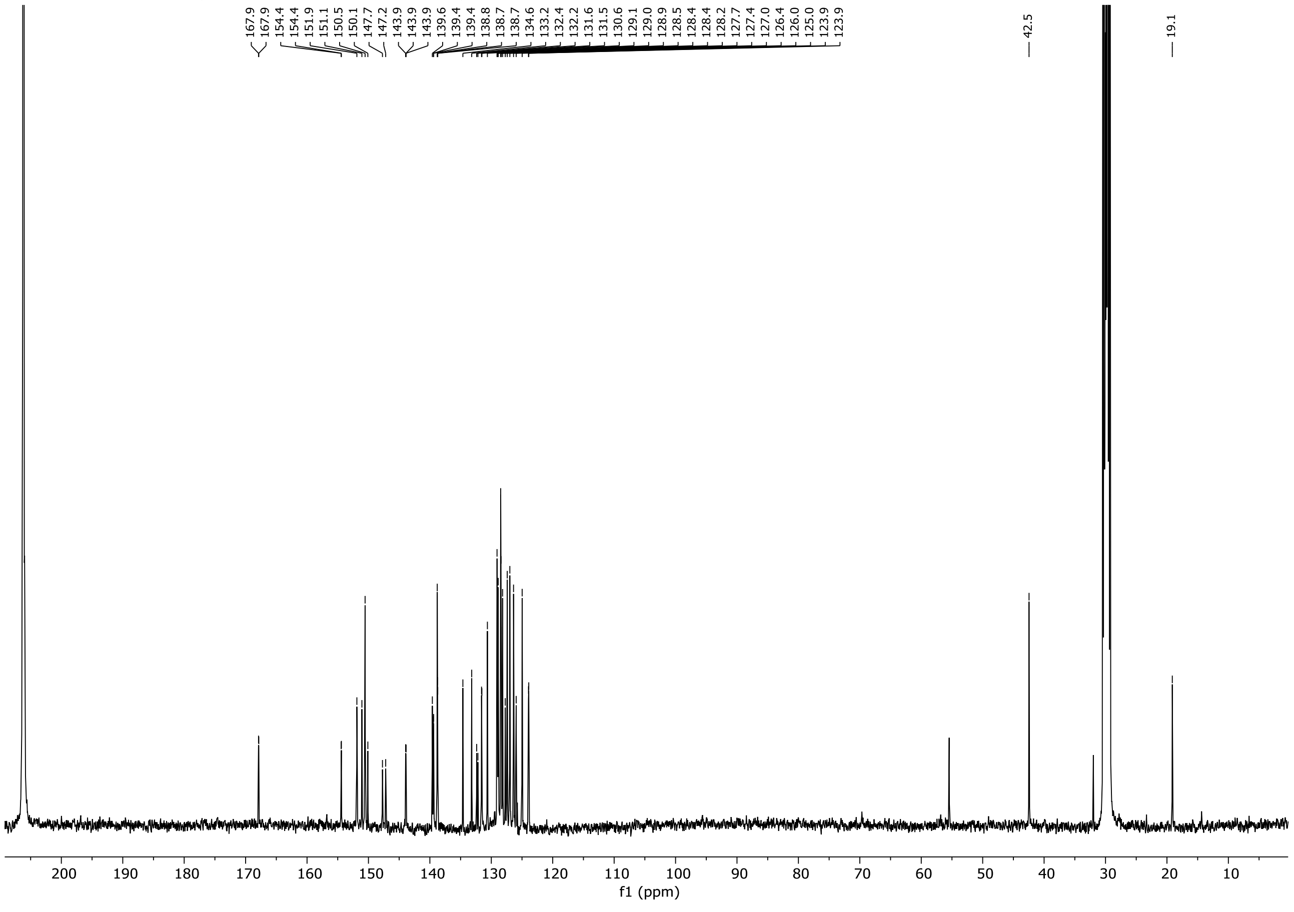

PC5, ^{1}H NMR (400 MHz, Acetone-d_6)

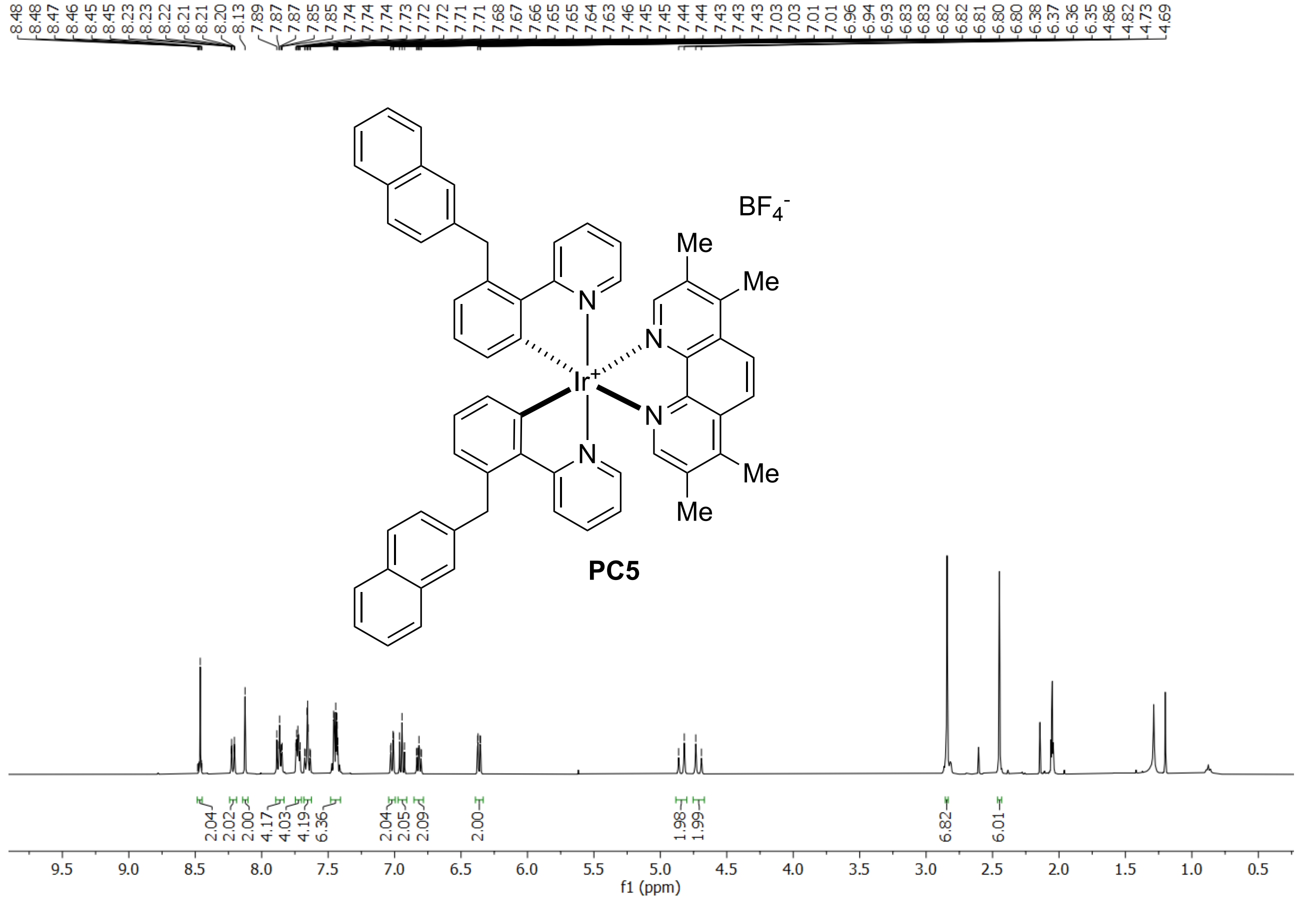

PC5, ^{13}C NMR (101 MHz, Acetone-d_6)

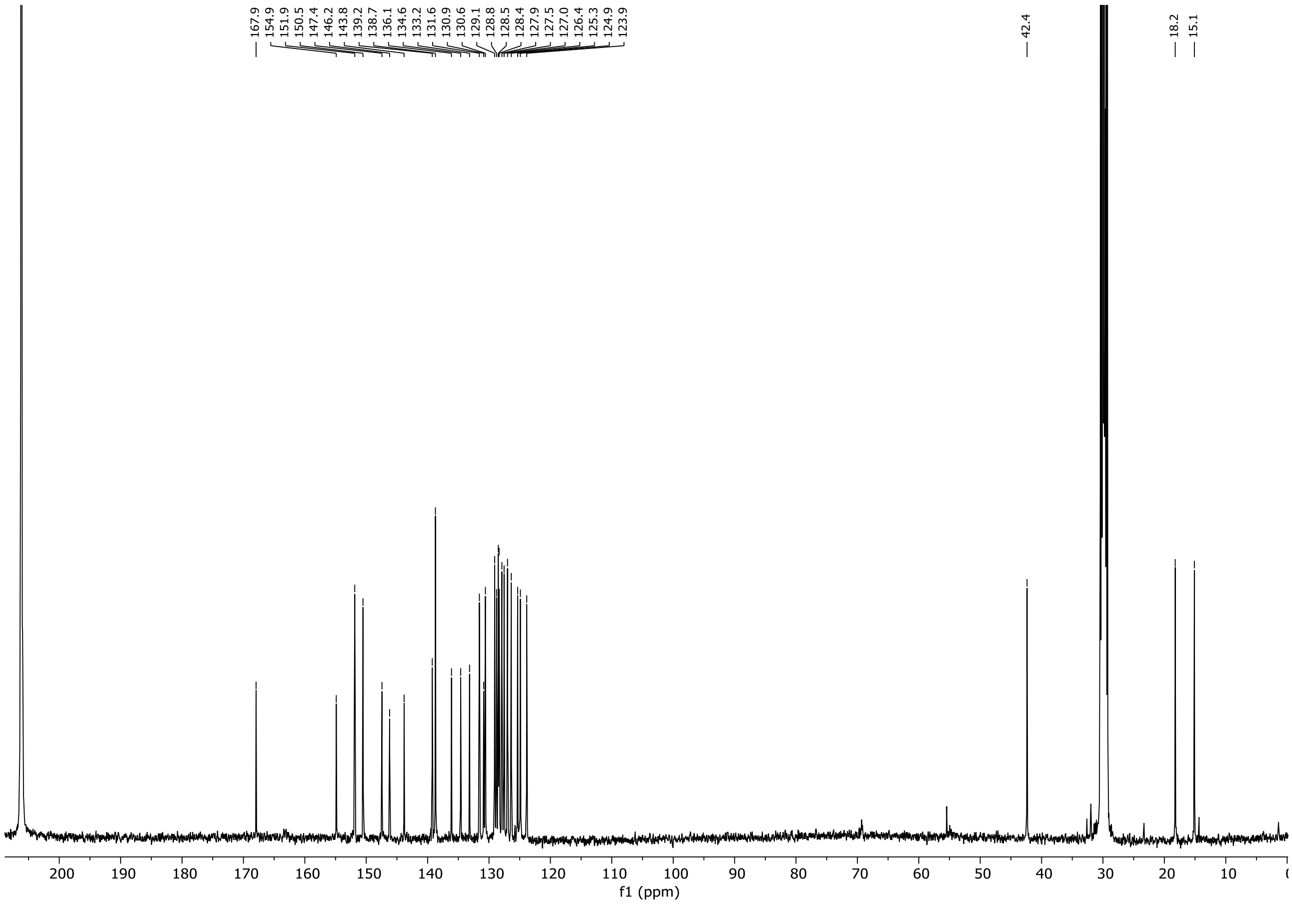

PC6, ^{1}H NMR (400 MHz, Acetone-d_6)

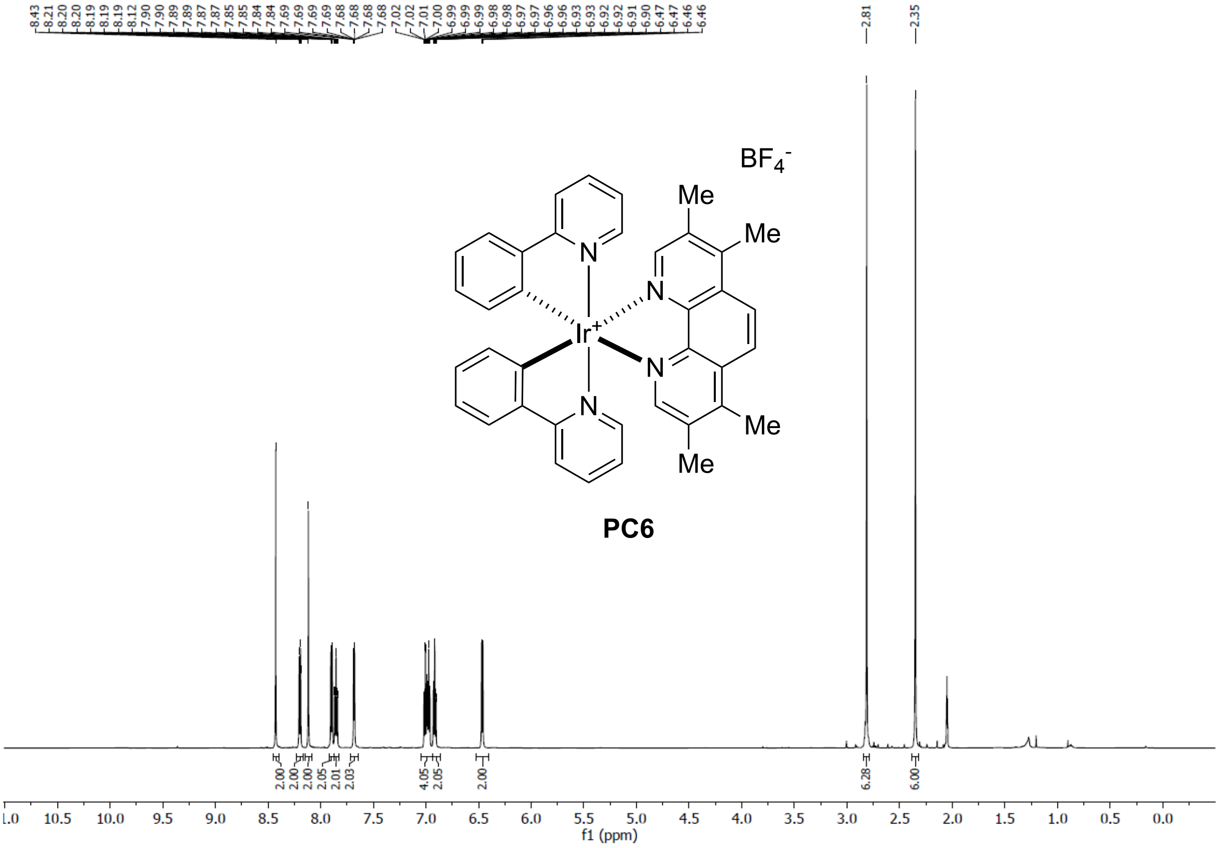

PC6, ^{13}C NMR (101 MHz, Acetone-d_6)

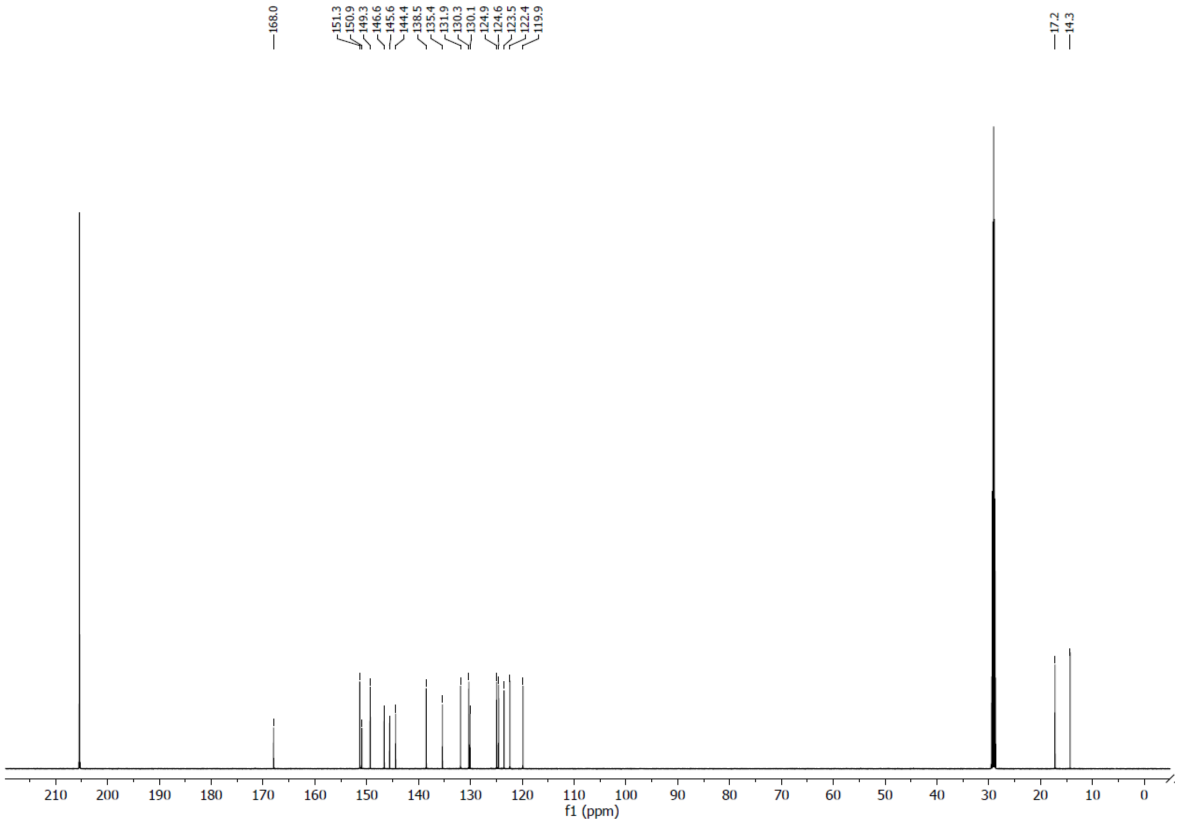

PC7, ^{1}H NMR (400 MHz, Acetone-d_6)

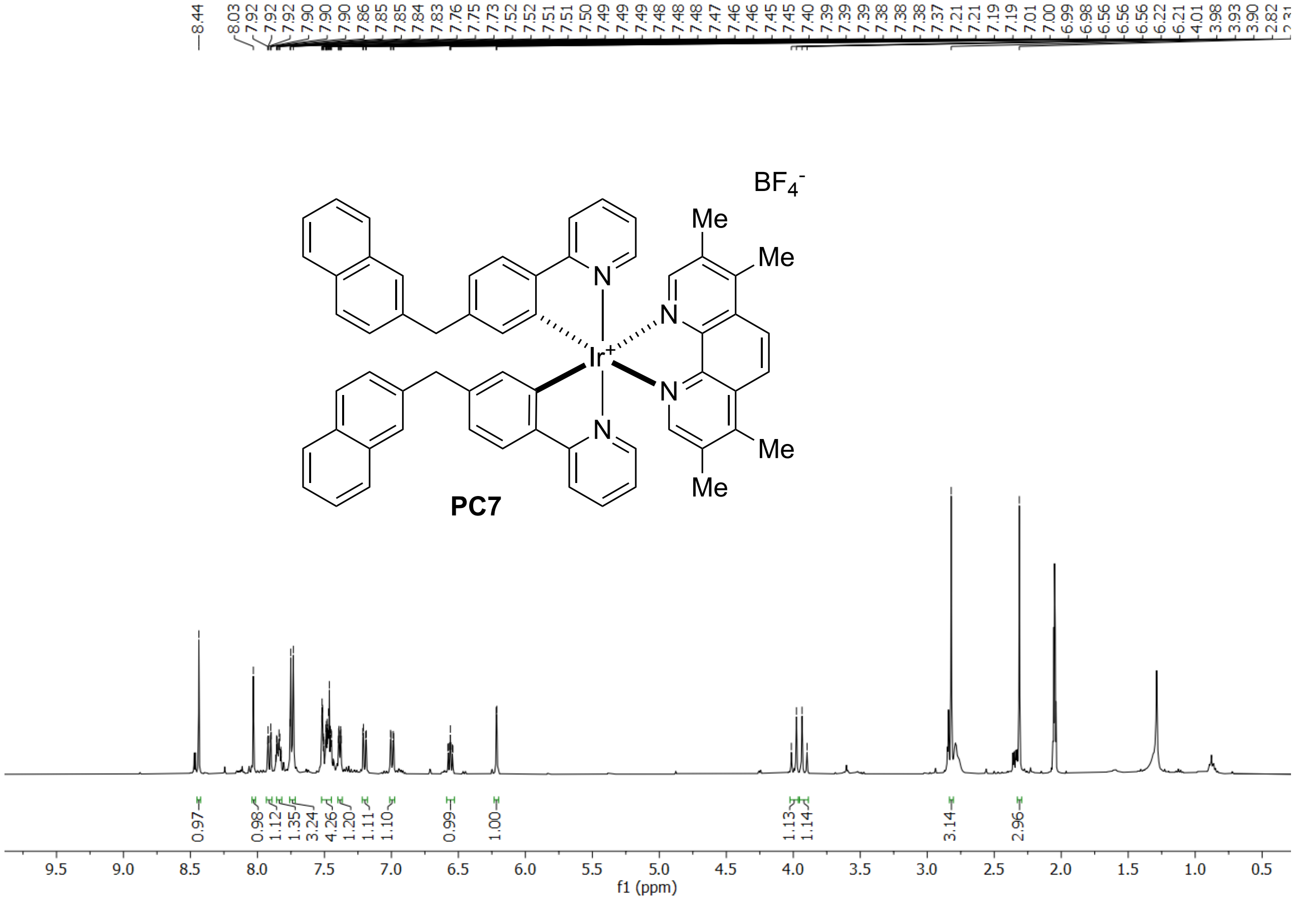

PC7, ^{13}C NMR (101 MHz, Acetone-d_6)

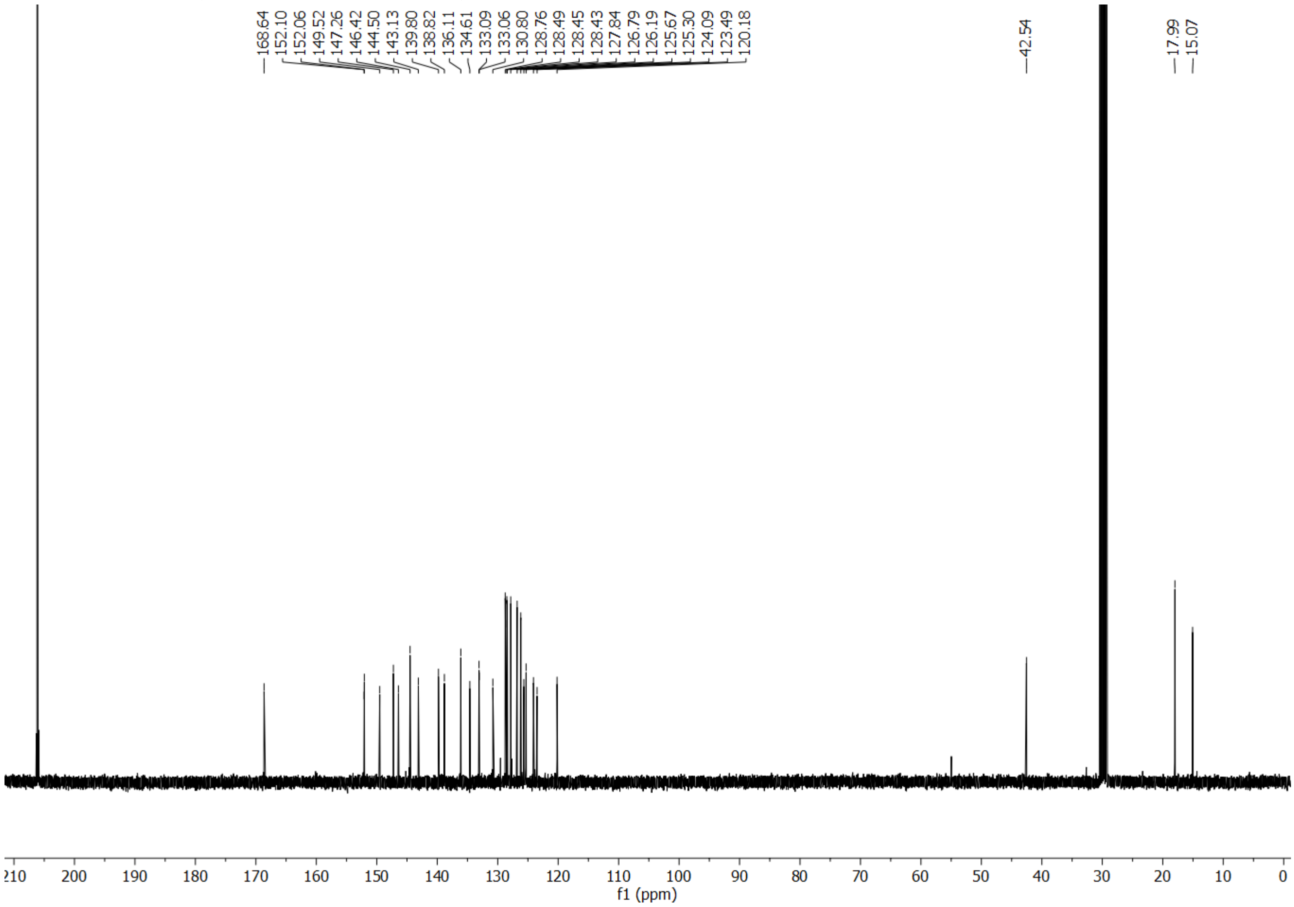

PC8, ^{1}H NMR (400 MHz, CD_2Cl_2)

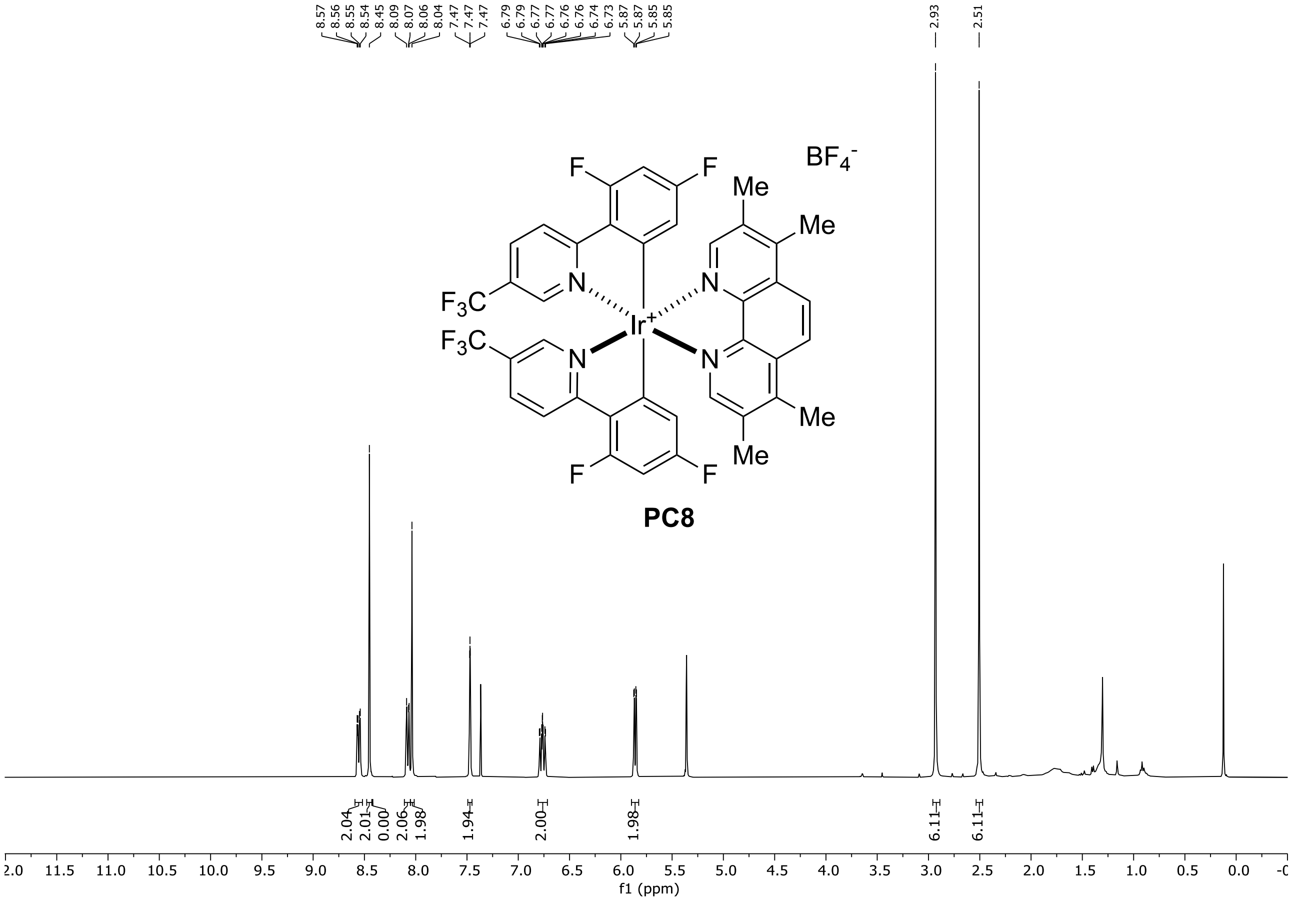

PC8, ^{13}C NMR (101 MHz, CD_2Cl_2)

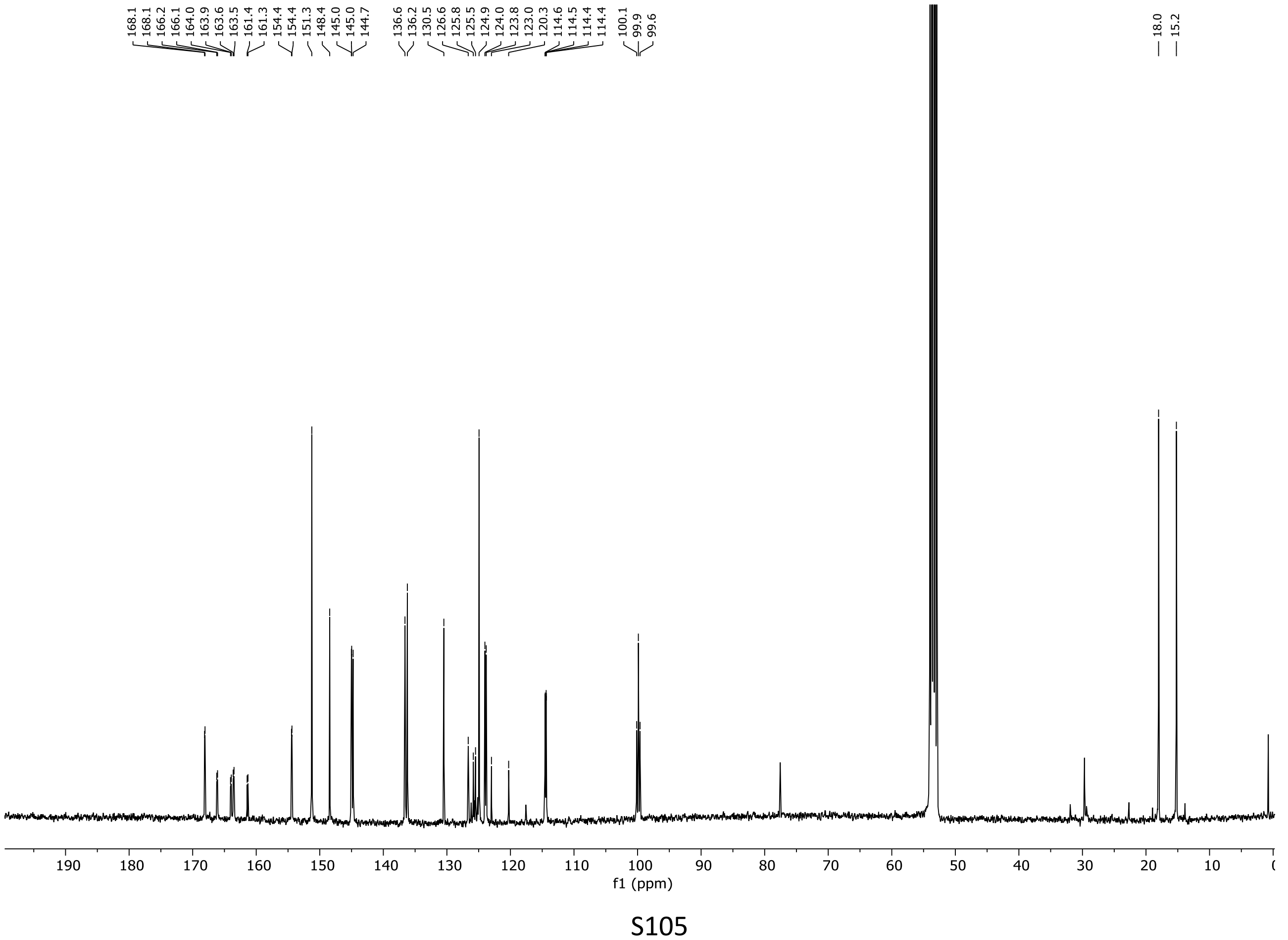

PC8, ^{19}F NMR (565 MHz, CD_2Cl_2)

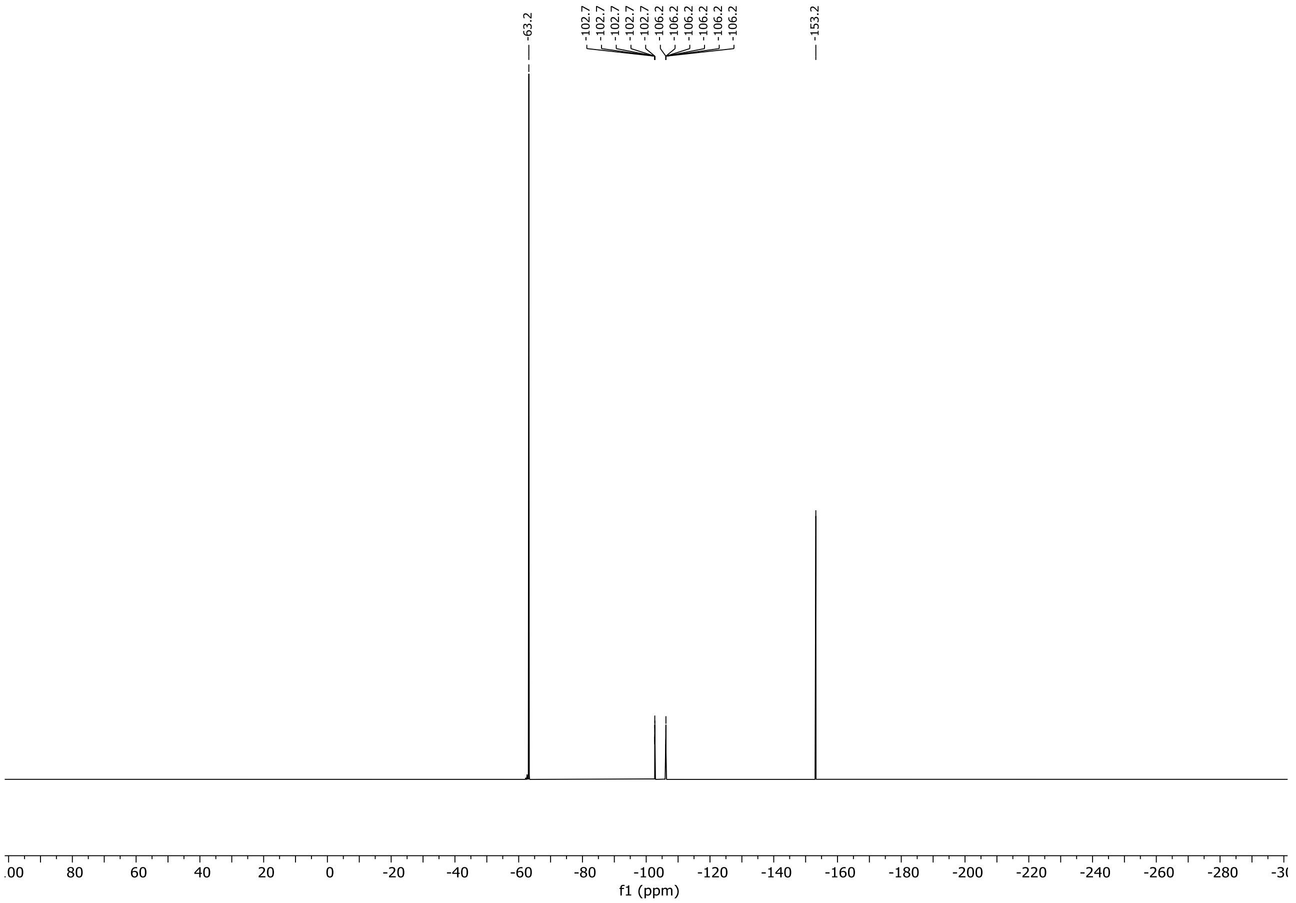

PC9, ^{1}H NMR (400 MHz, $CDCl_3$)

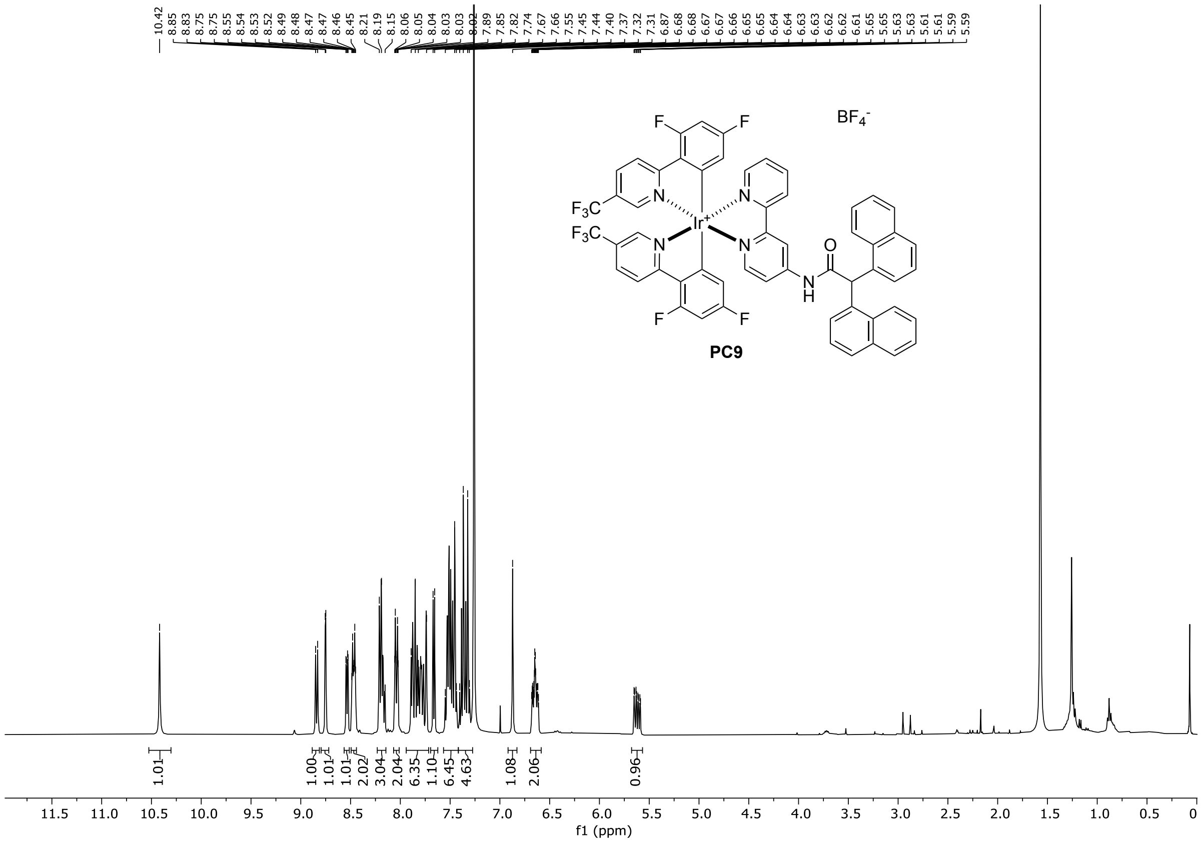

PC9, ^{13}C NMR (101 MHz, $CDCl_3$)

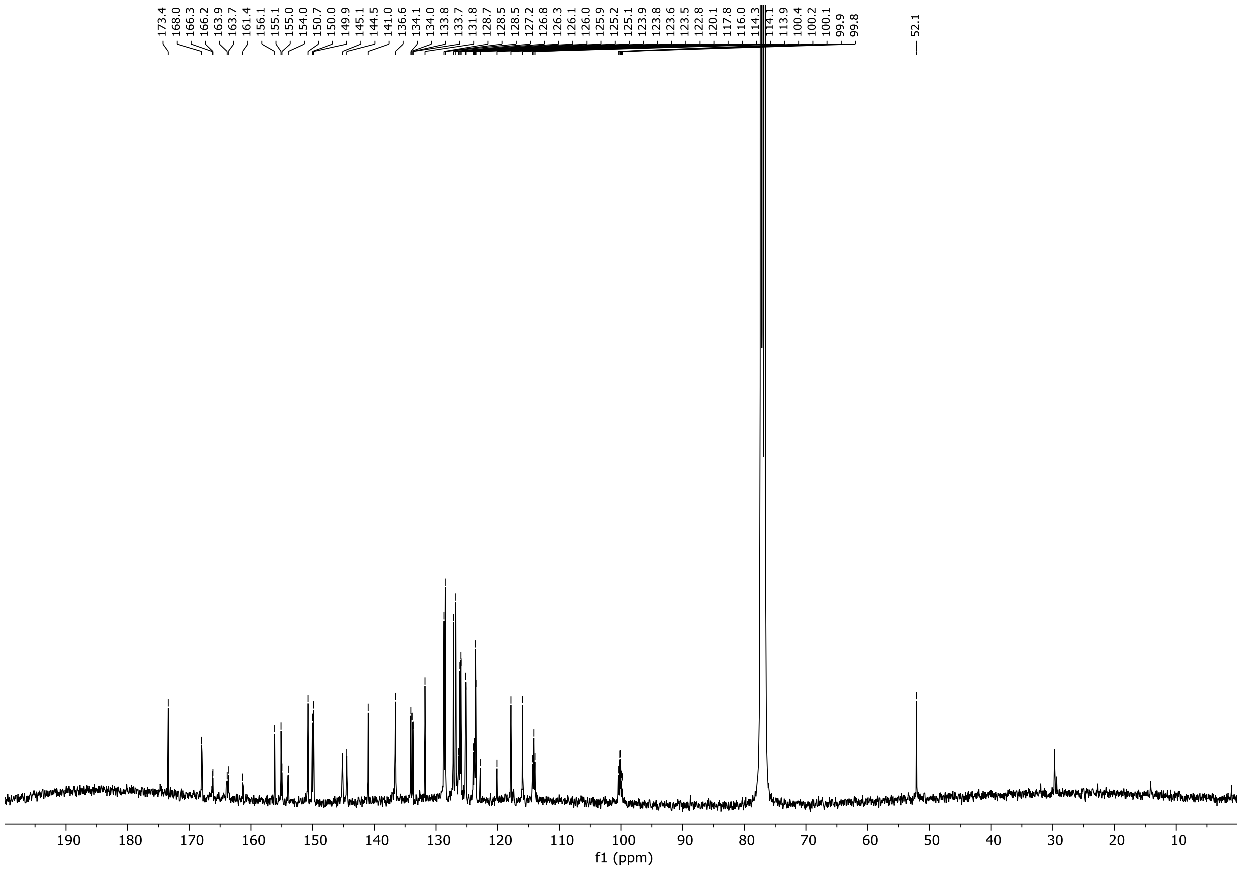

PC9, ^{19}F NMR (565 MHz, $CDCl_3$)

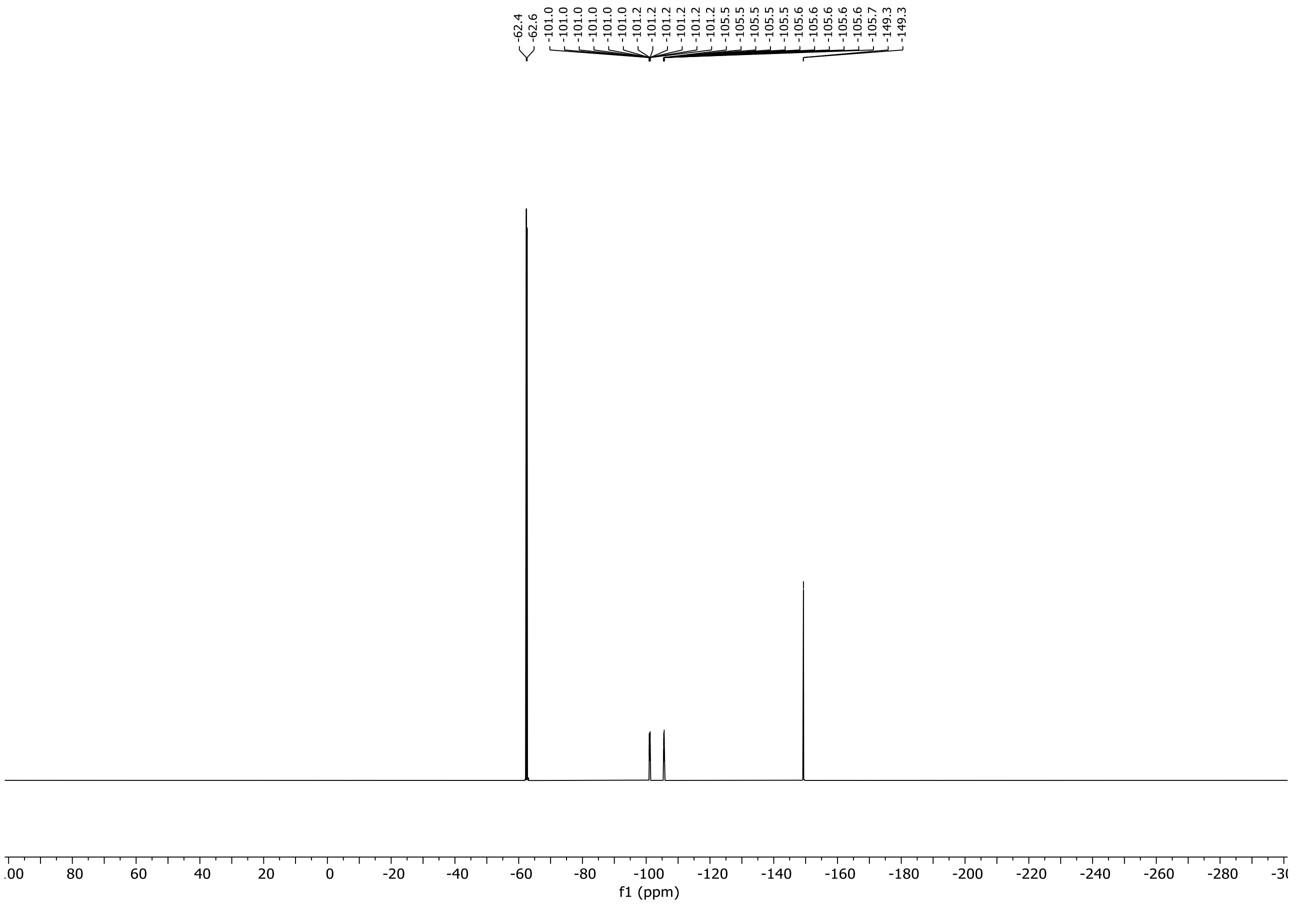

PC10, ^{1}H NMR (600 MHz, $CDCl_3$)

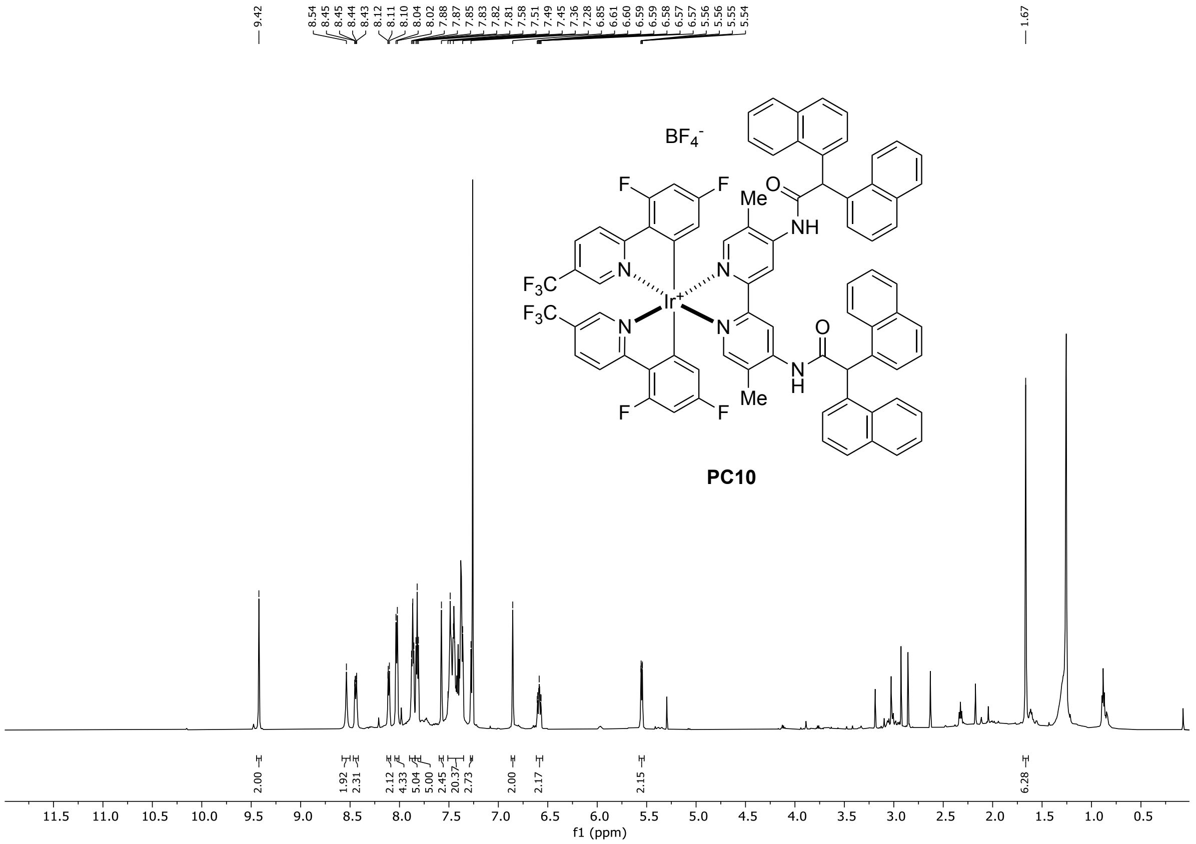

PC10, ^{13}C NMR (151 MHz, $CDCl_3$)

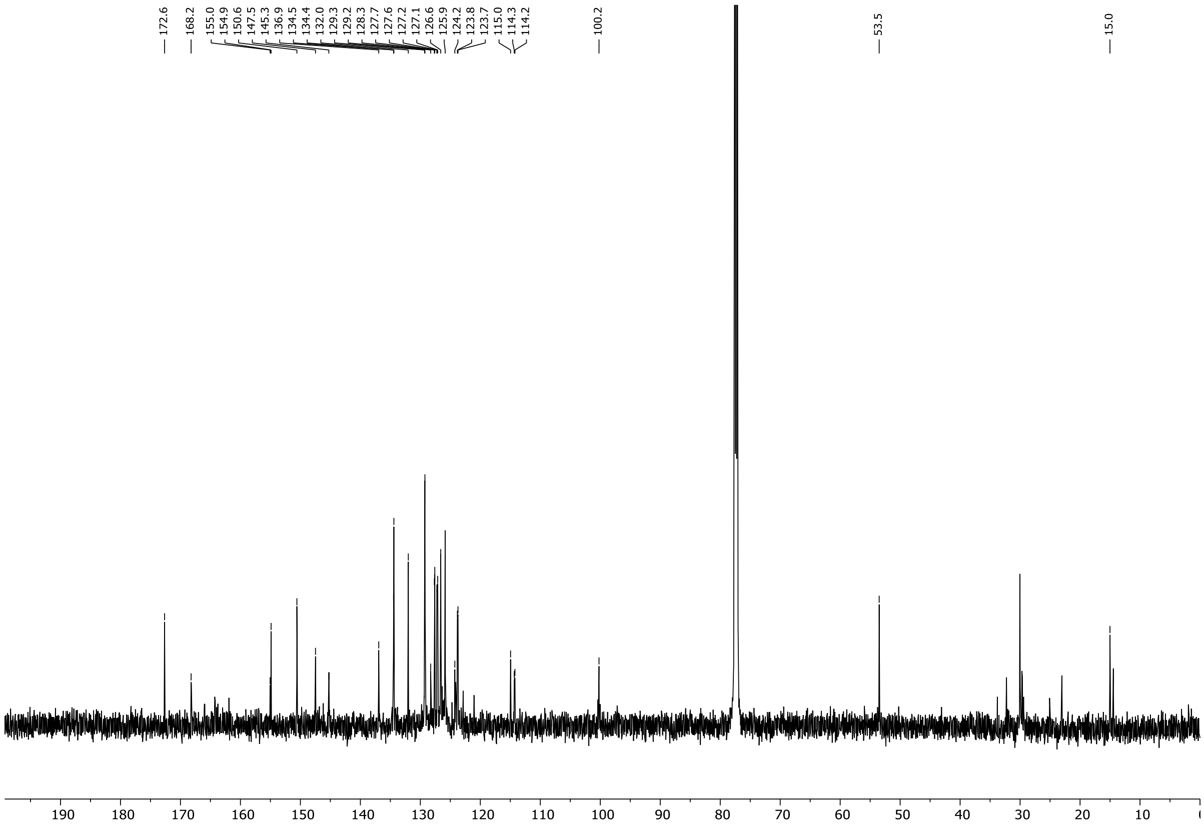

PC10,^{19}F NMR (565 MHz, $CDCl_3$)

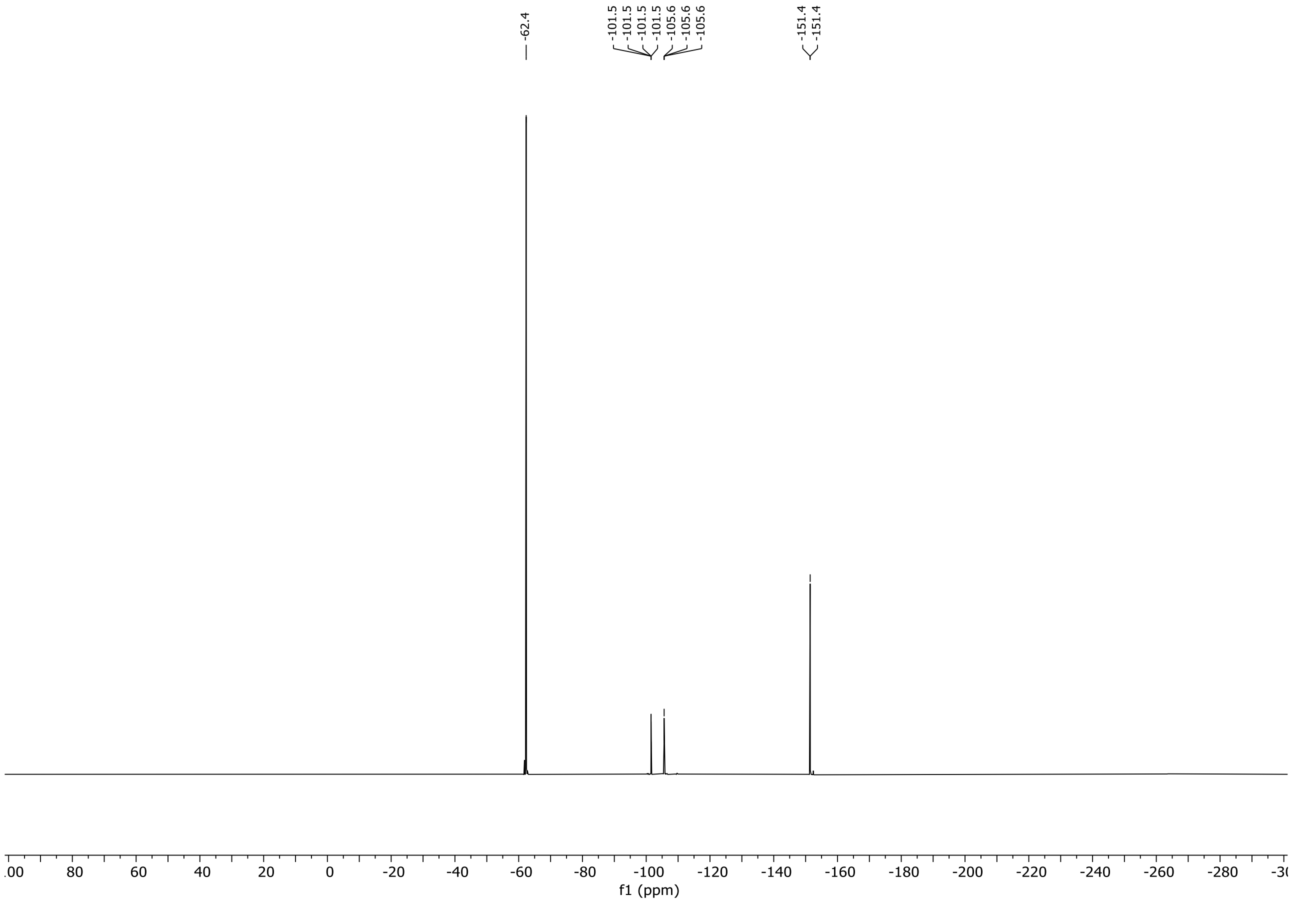

PC11, ^{1}HNMR (600MHz, *Acetone-d$_6$*)

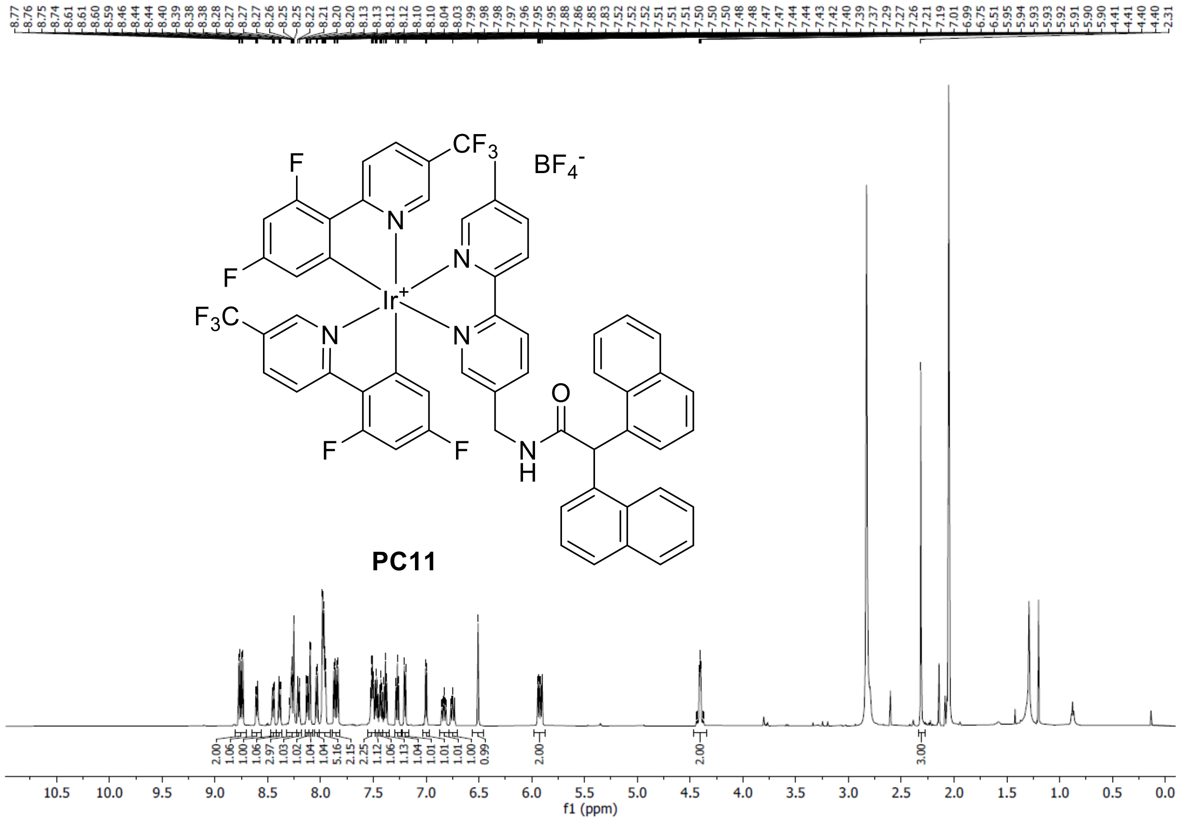

PC11, ^{13}CNMR (151 MHz, *Acetone-d$_6$*)

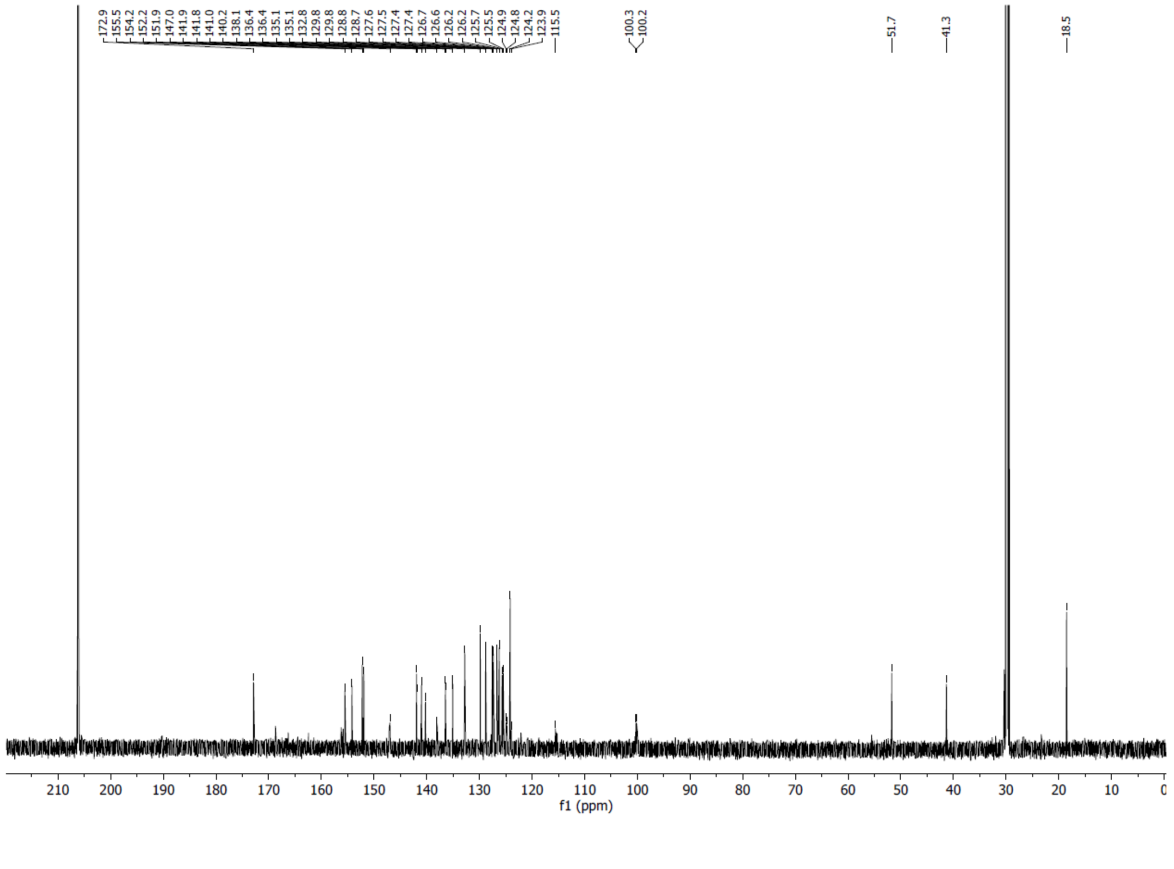

PC11, ^{19}FNMR (565 MHz, *Acetone-d$_6$*)

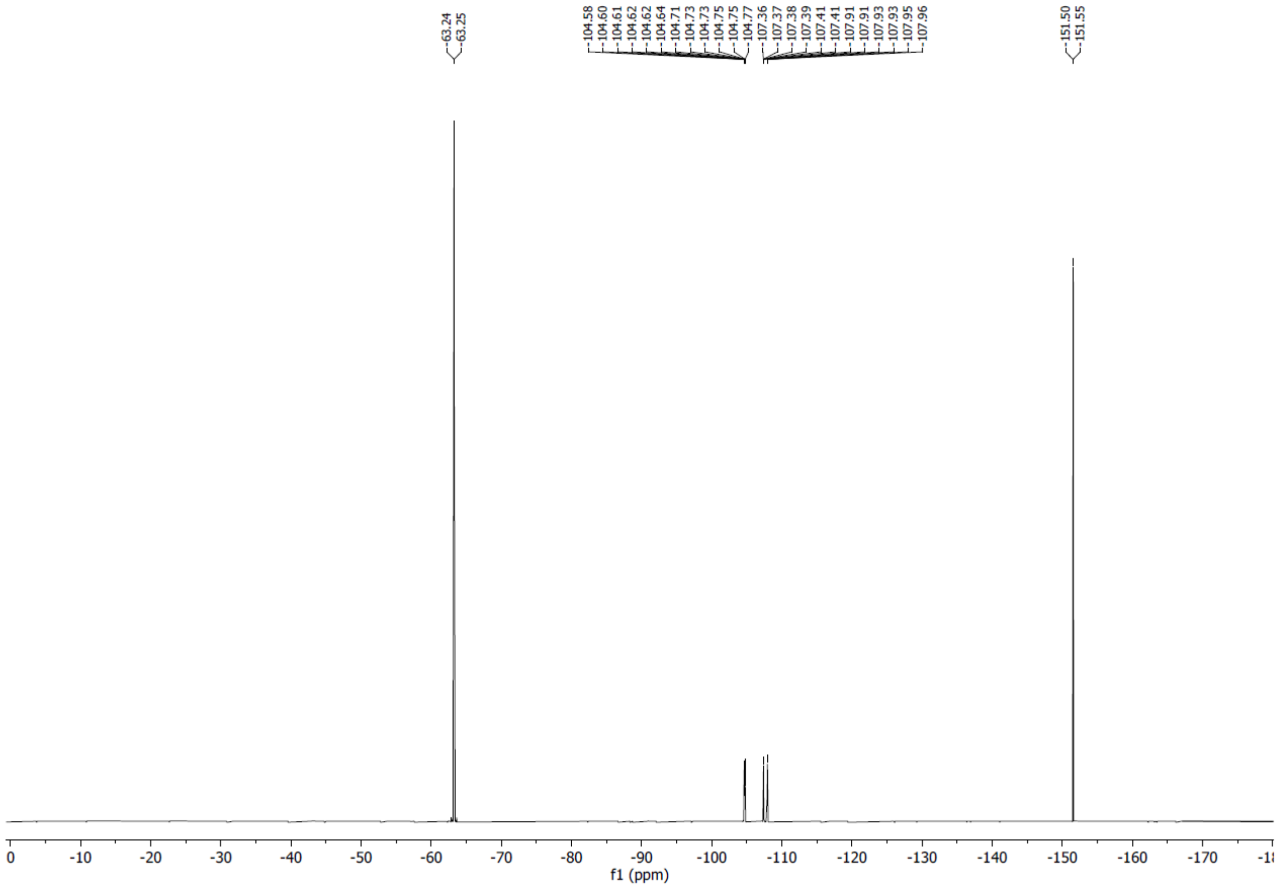

C, ^{1}H NMR (400 MHz, $CDCl_3$)

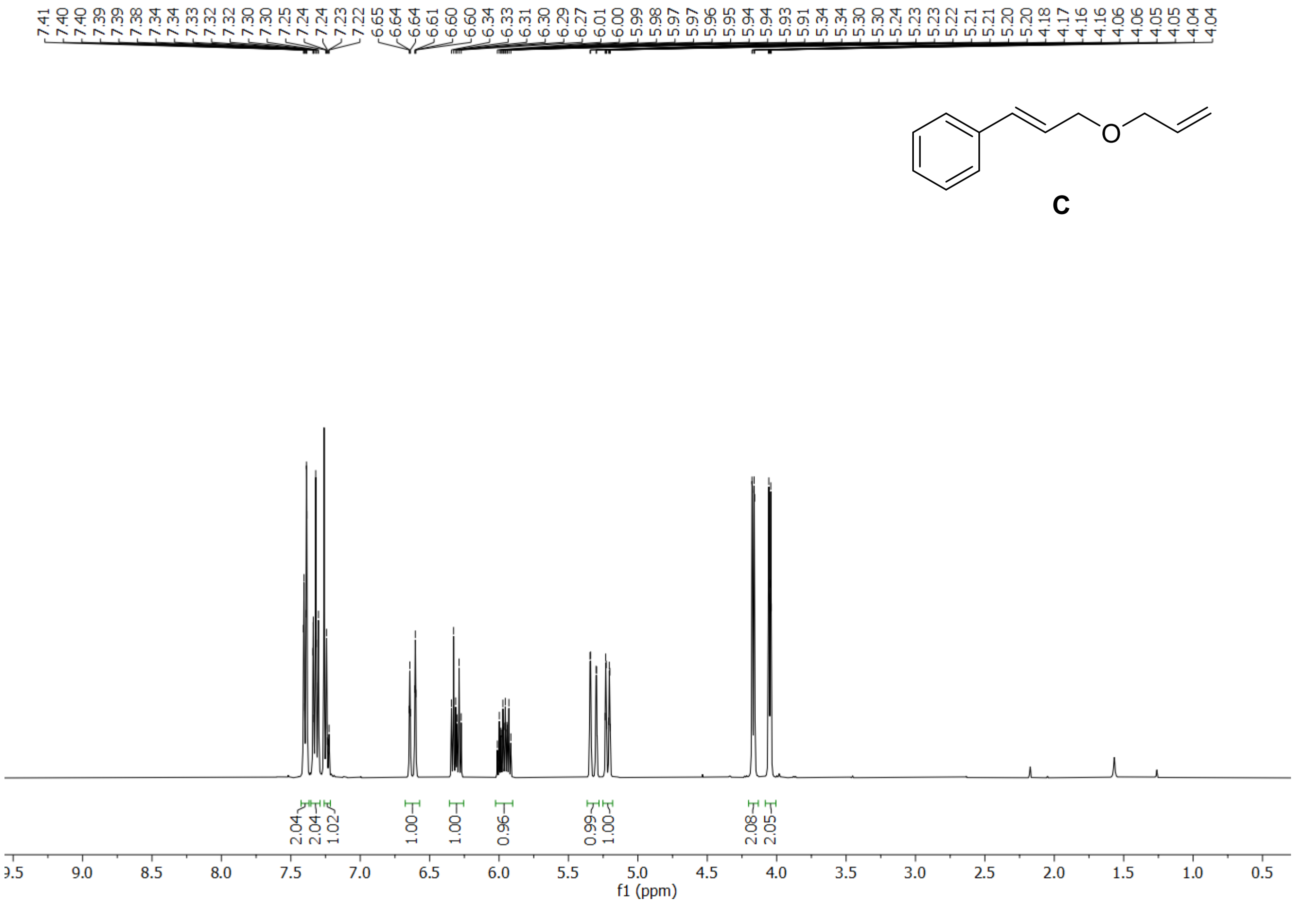

E, ^{1}H NMR (400 MHz, $CDCl_3$)

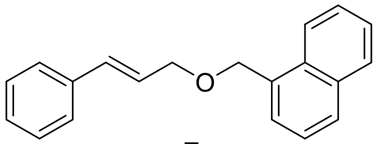

E

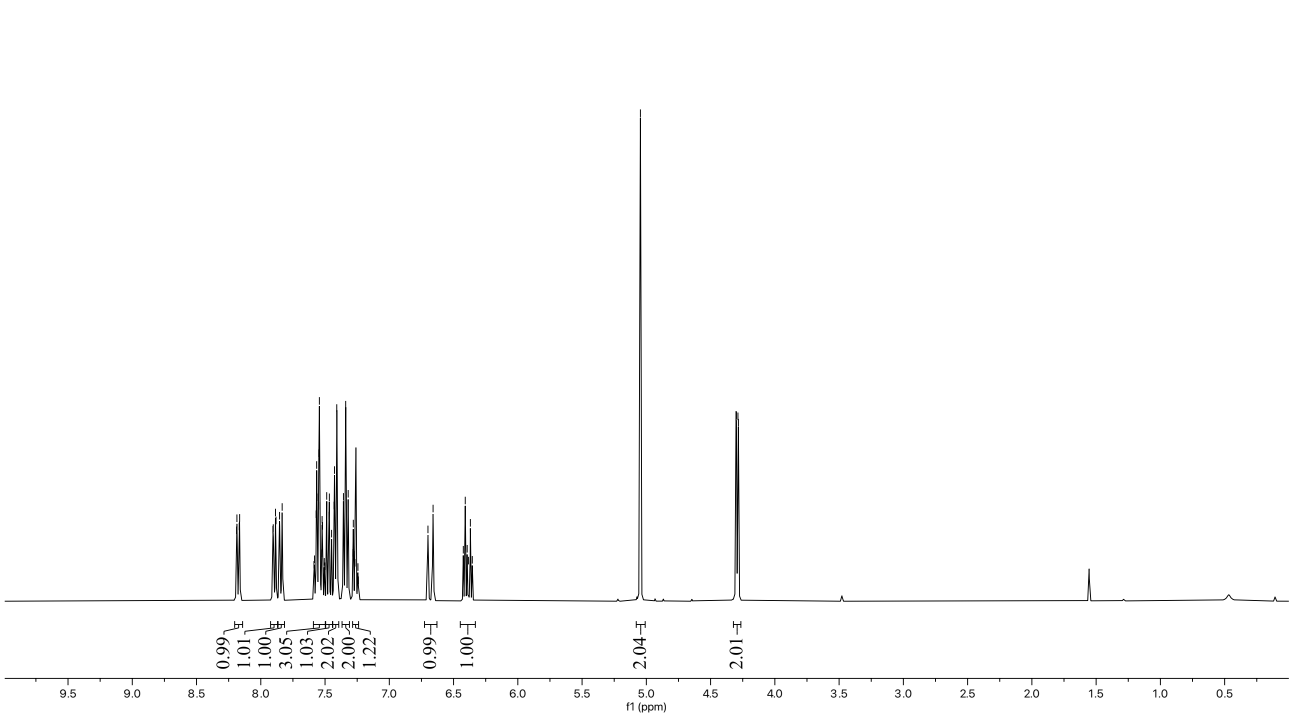

E, ^{13}C NMR (101 MHz, $CDCl_3$)

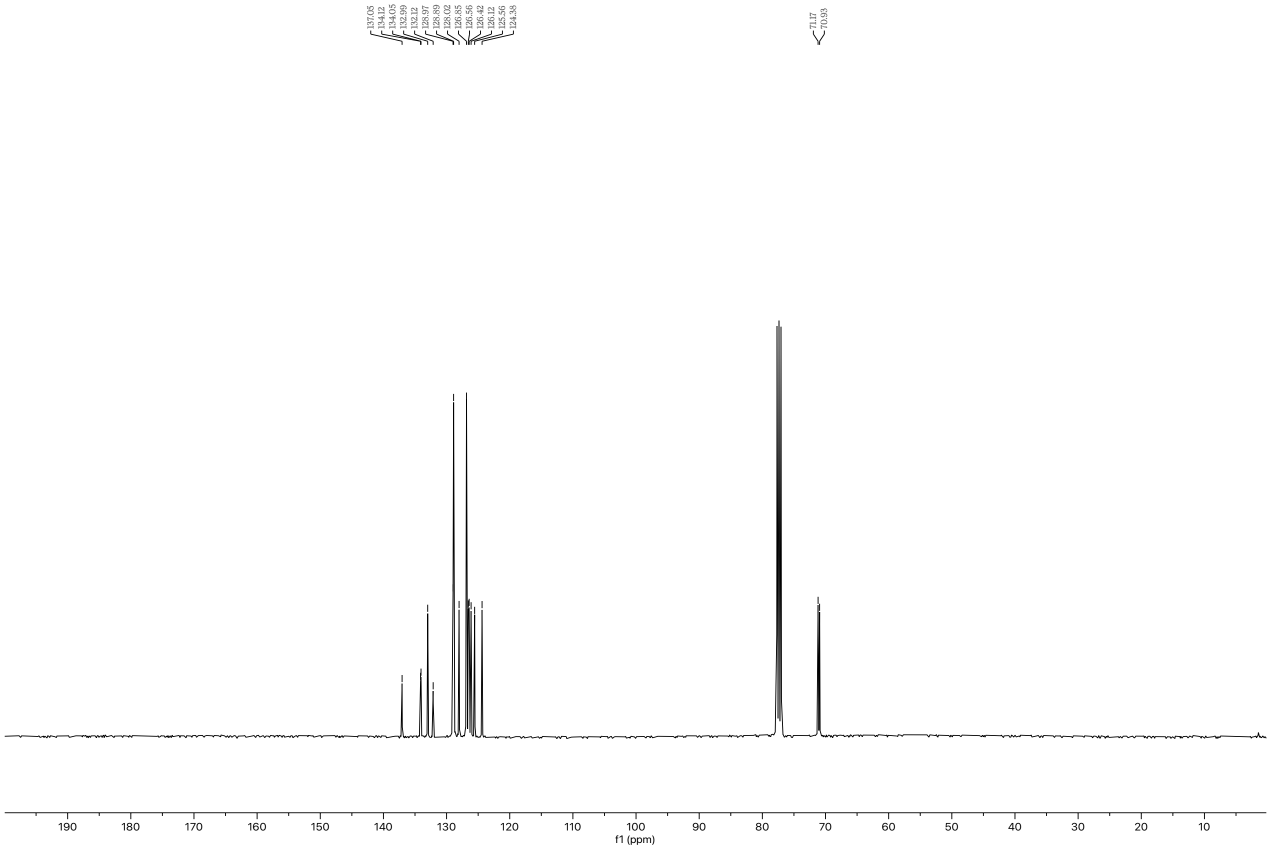

G, ^{1}HNMR (400MHz, $CDCl_3$)

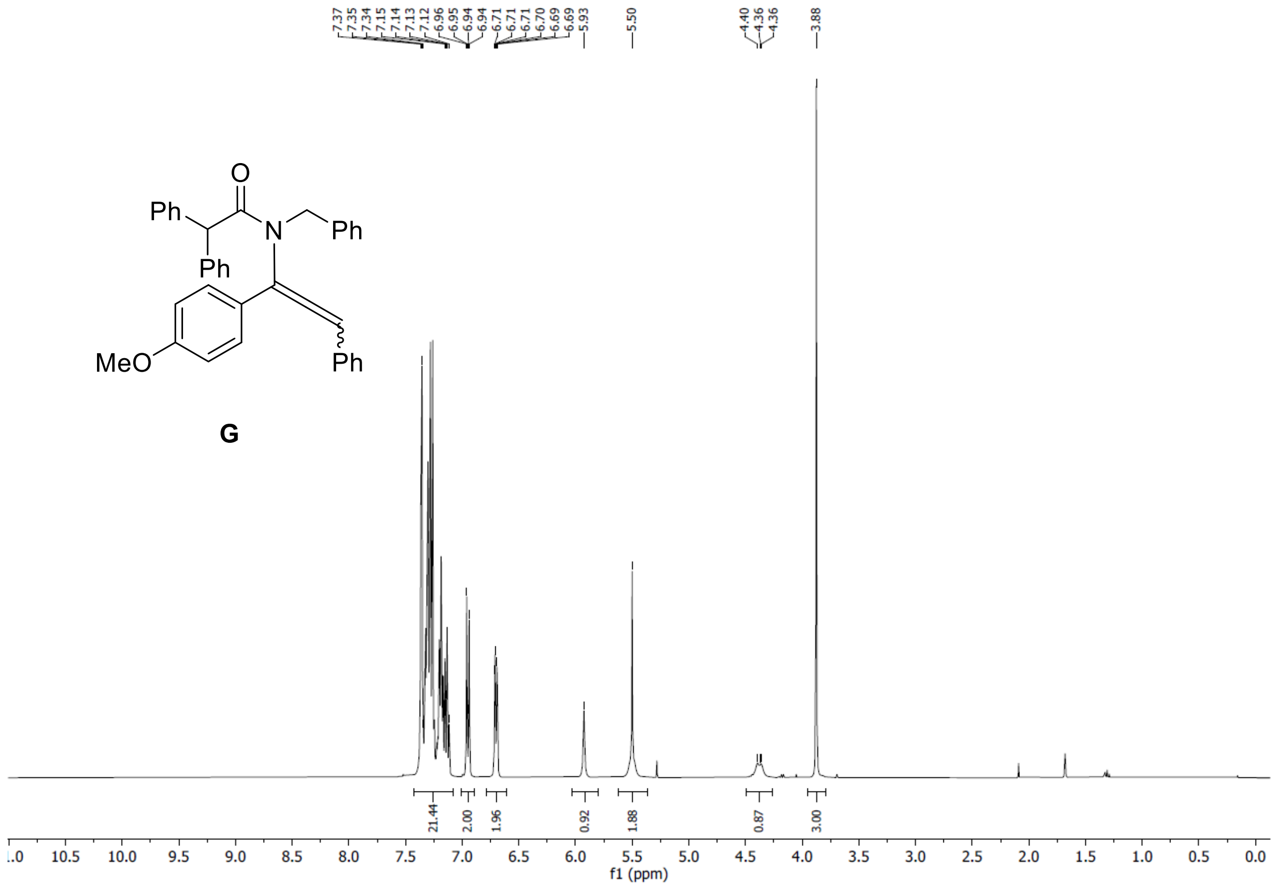

D, ^{1}H NMR (400 MHz, $CDCl_3$)

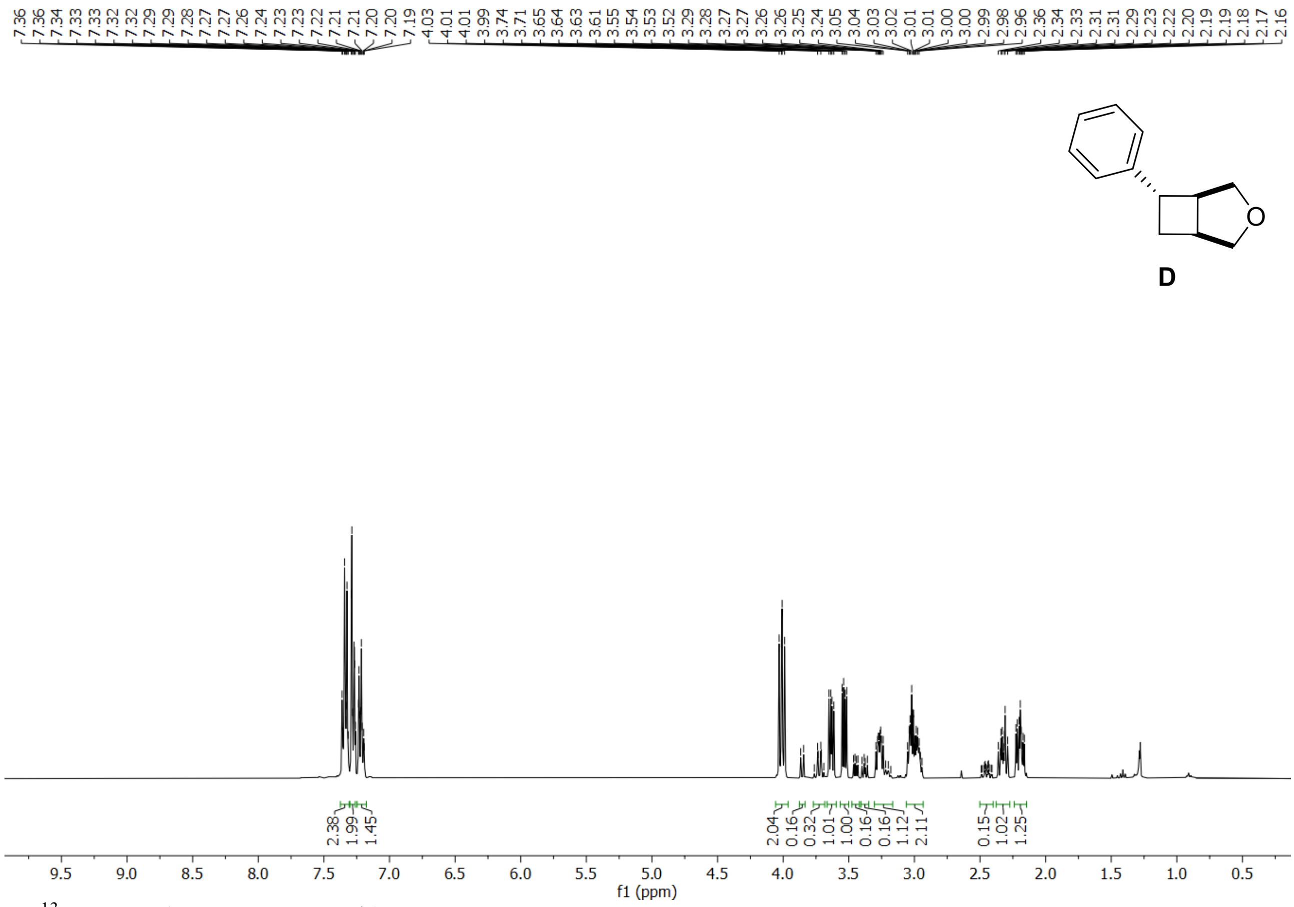

D, ^{13}C NMR (101 MHz, $CDCl_3$)

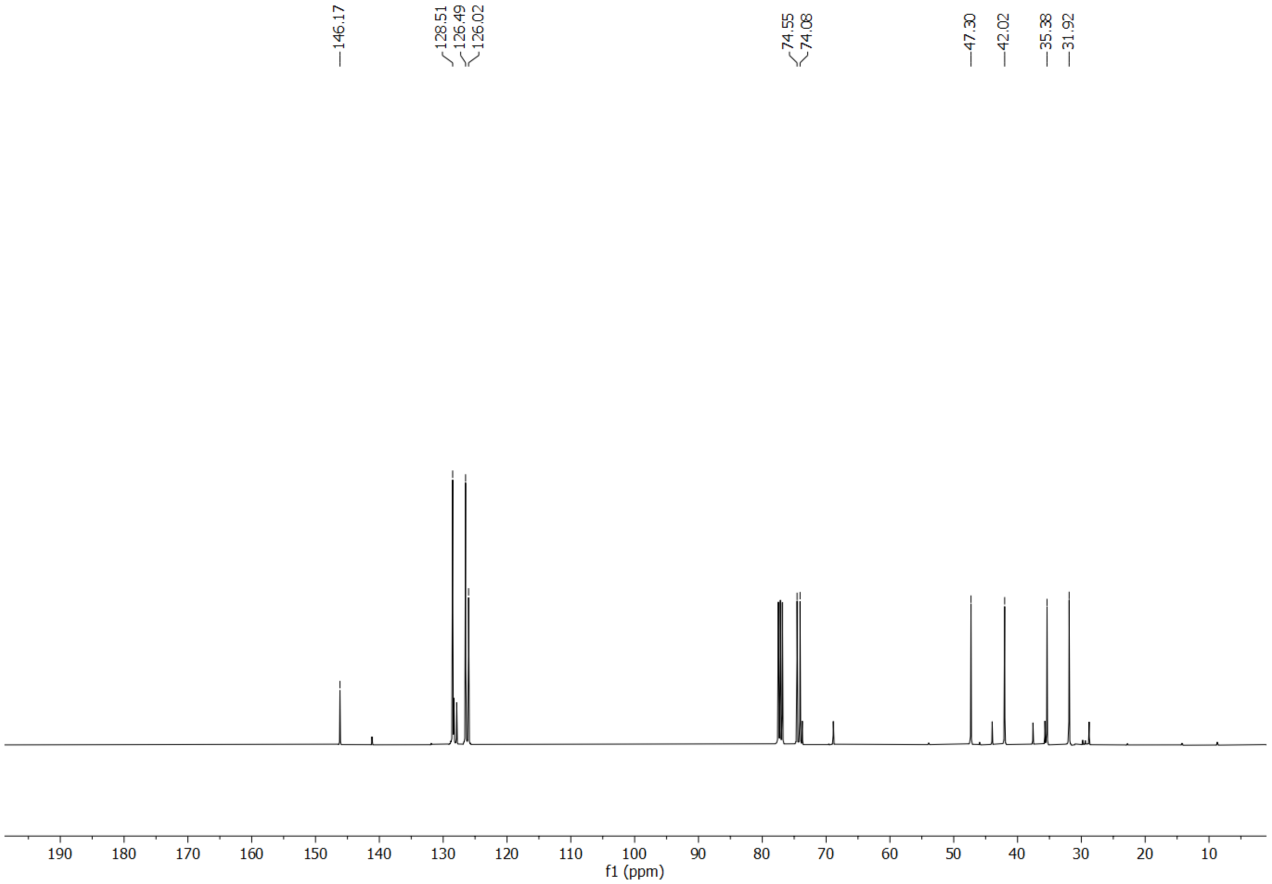

F, ^{1}H NMR (400 MHz, $CDCl_3$)

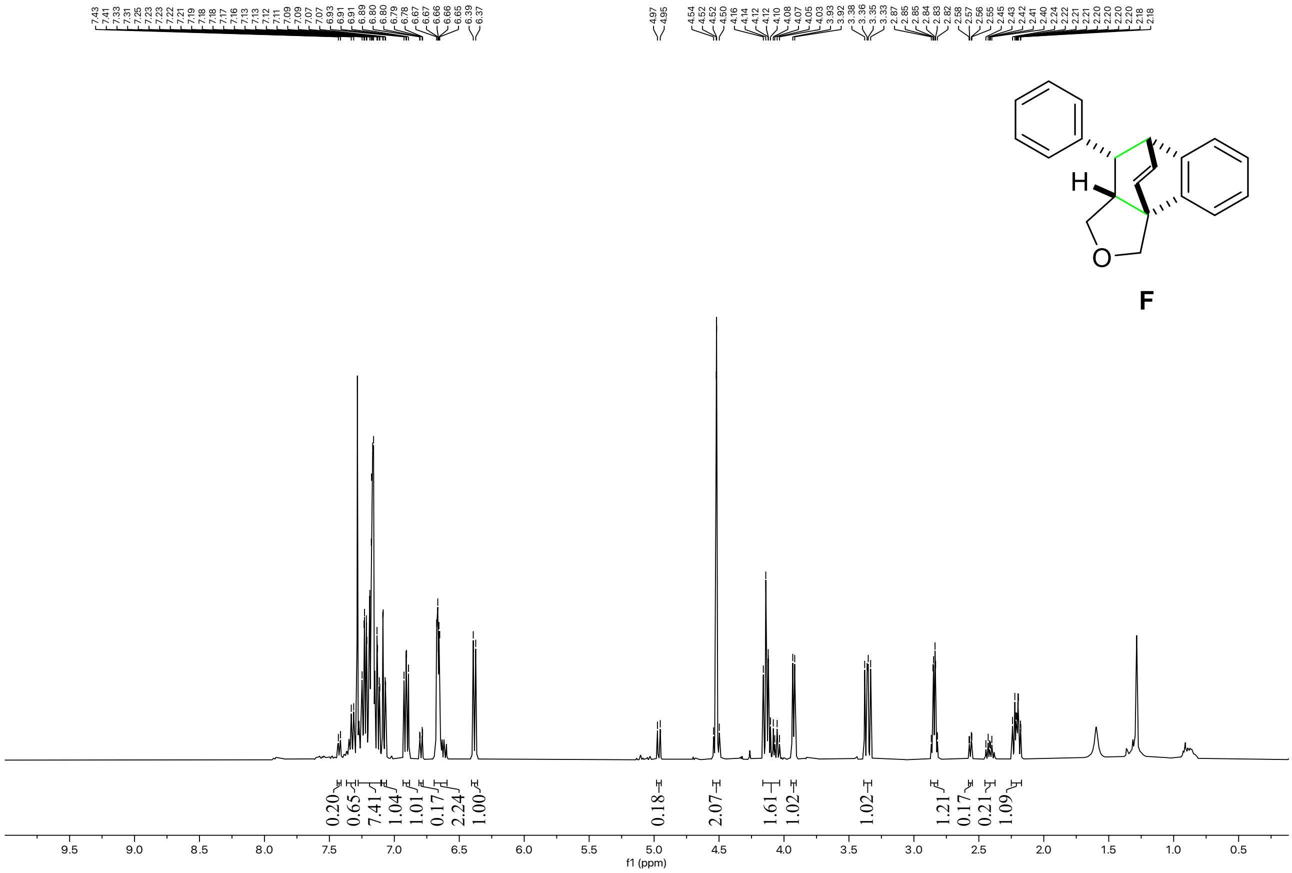

F, ^{13}C NMR (101 MHz, $CDCl_3$)

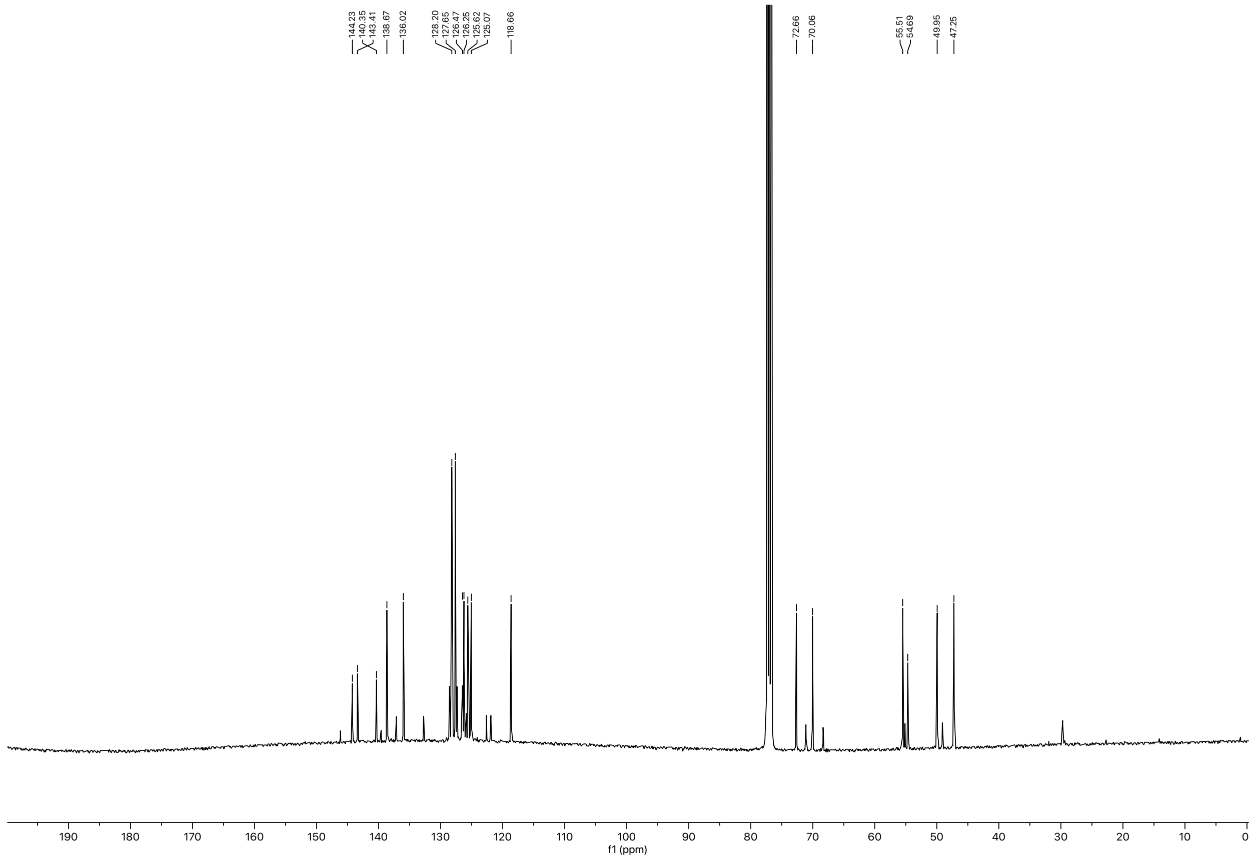

H, ^{1}HNMR (600MHz, $CDCl_3$)

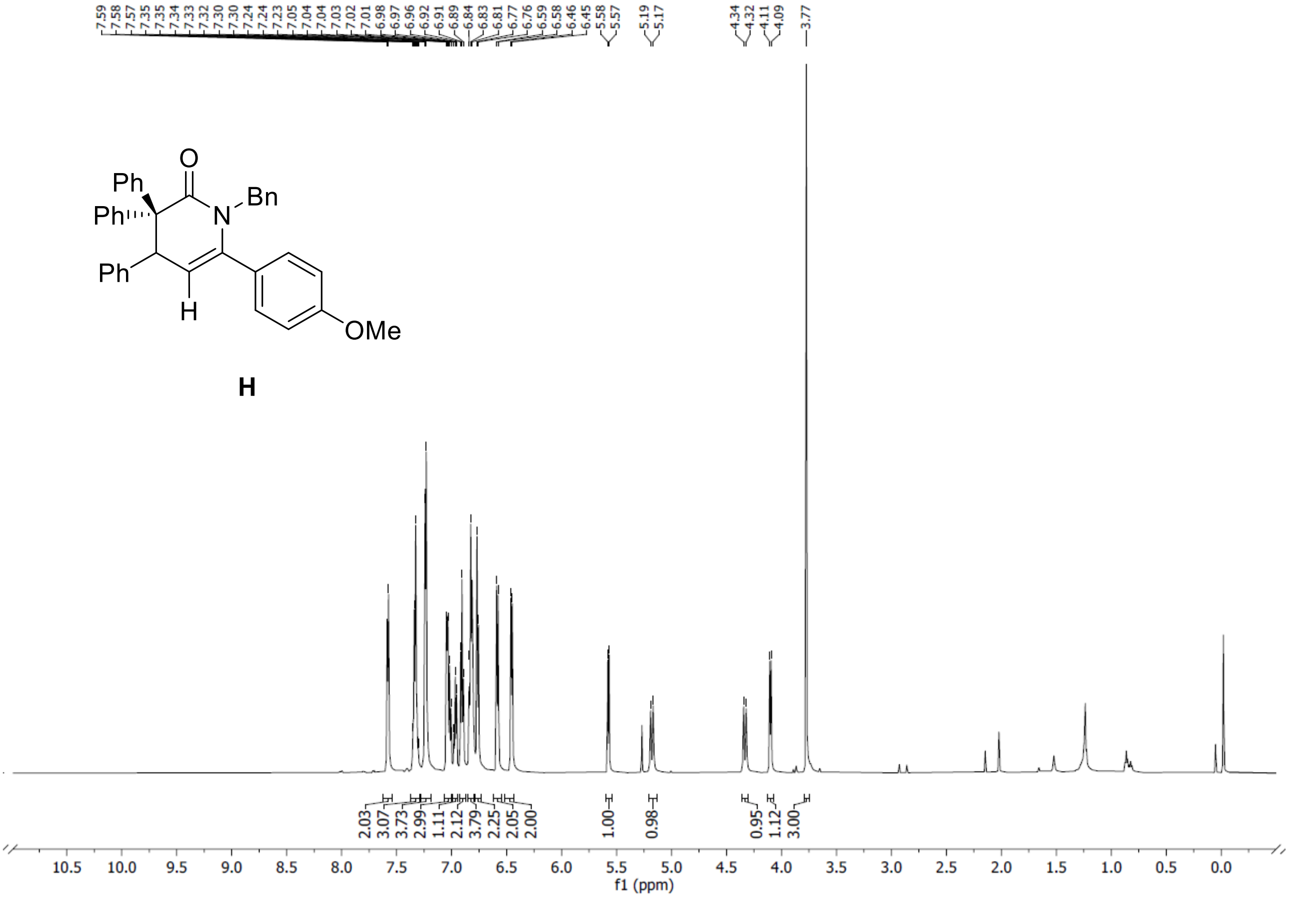

Photoisomerization of cinnamyl alcohol 3a, copies of NMR spectra

Photoisomerization with **Ir(dF(CF$_3$)ppy$_2$)bpy(PF$_6$)**, crude **A** + **B**, ^{1}H NMR (400 MHz, CDCl$_3$)

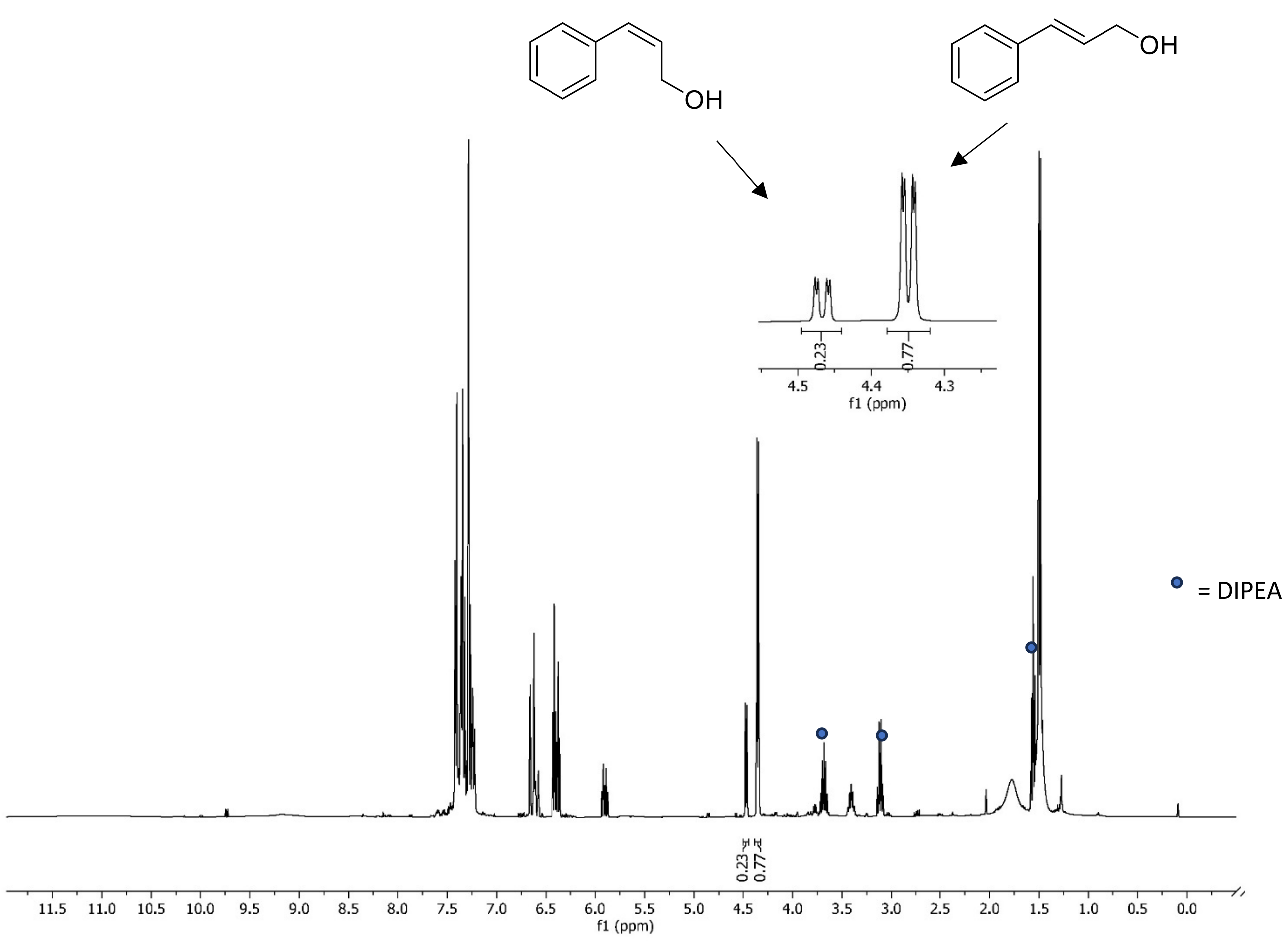

Photoisomerization with **Ir[(ppy)$_2$bpy](BF$_4$)**, crude **A** + **B**, ^{1}H NMR (400 MHz, $CDCl_3$)

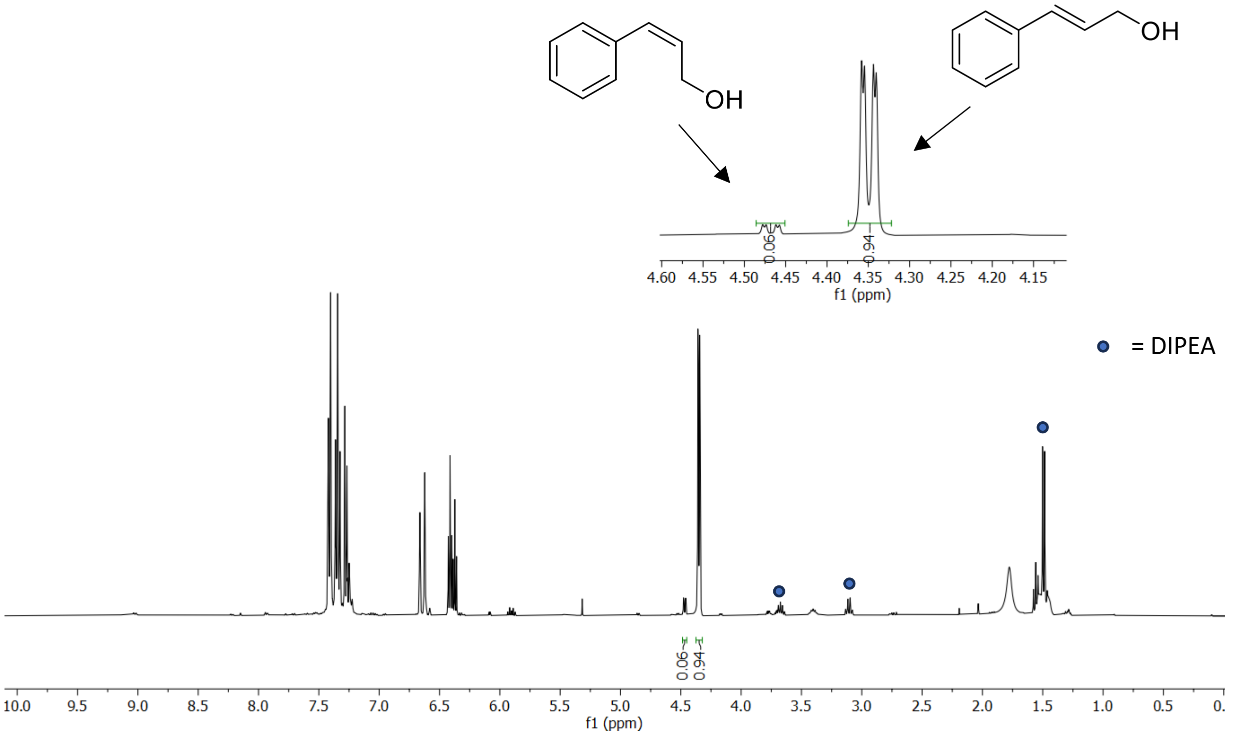

Photoisomerization with **PC1**, **A+B**, [1] H NMR (400 MHz, $CDCl_3$)

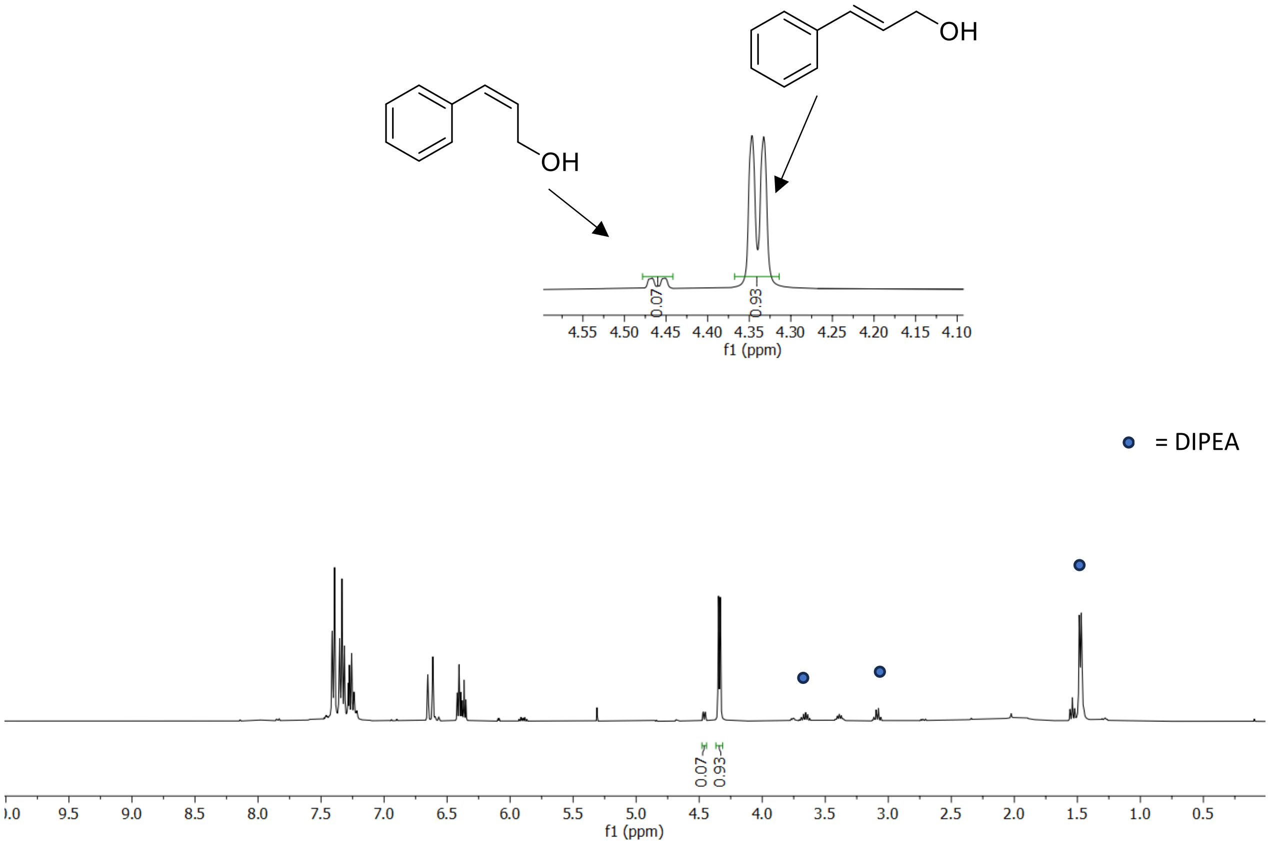

Photoisomerization with **PC2**, crude **A**+**B**, [1] H NMR (400 MHz, $CDCl_3$)

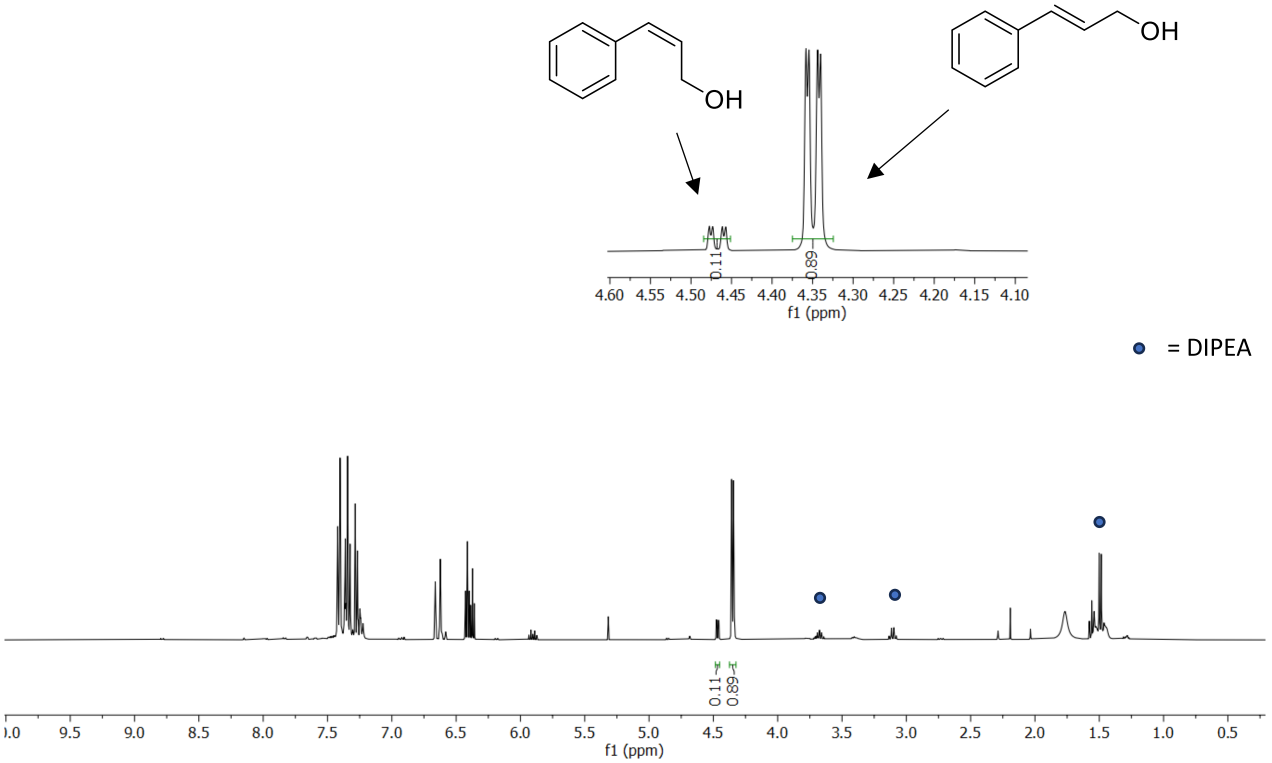

Photoisomerization with **PC3**, crude **A**+**B**, ^{1}H NMR (400 MHz, $CDCl_3$)

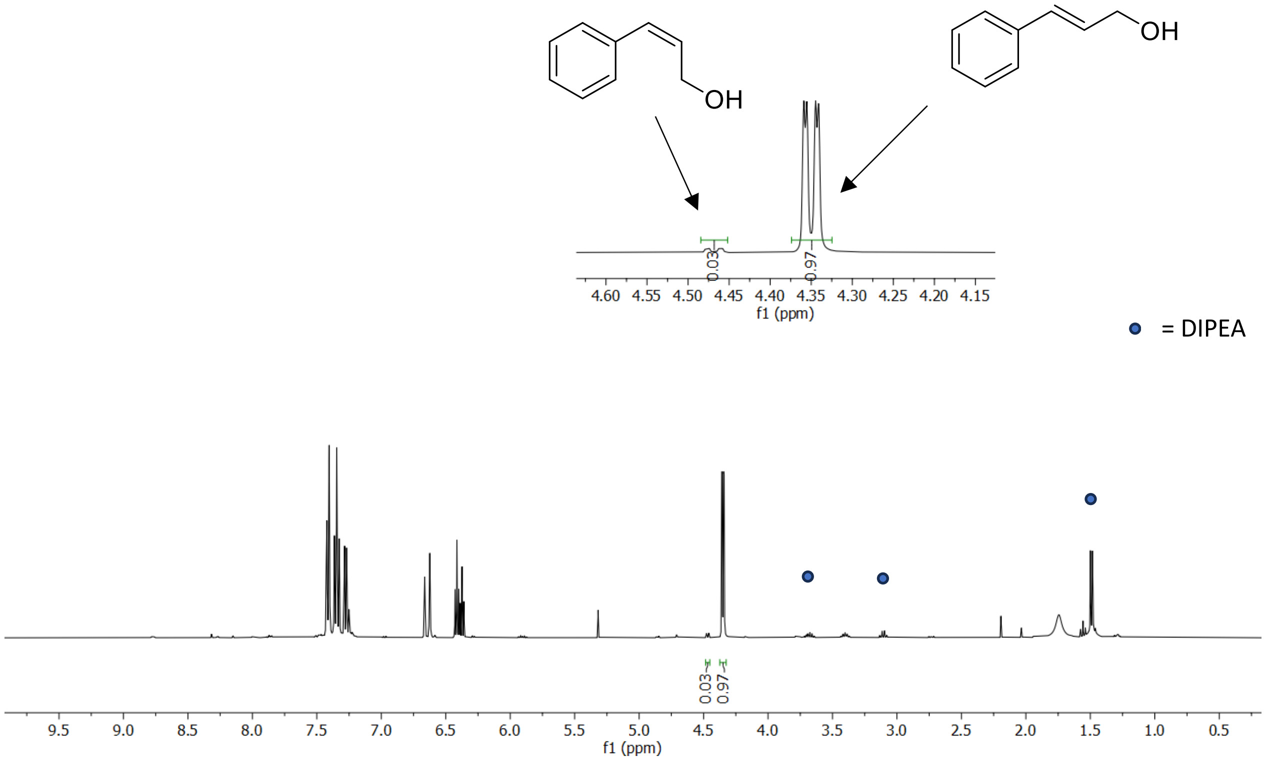

Photoisomerization with **PC4**, crude **A**+**B**, [1] H NMR (400 MHz, $CDCl_3$)

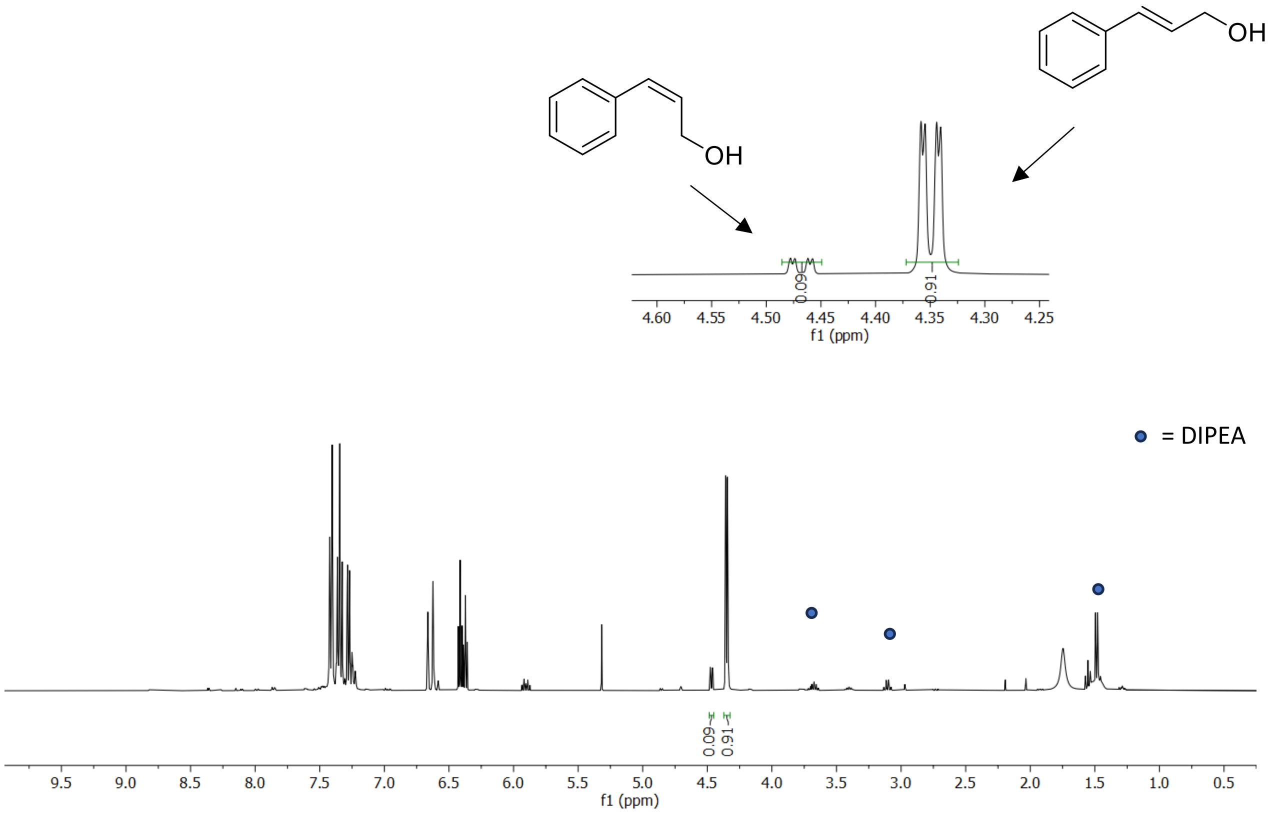

Photoisomerization with **PC5**, crude **A**+**B**, ^{1}H NMR (400 MHz, $CDCl_3$)

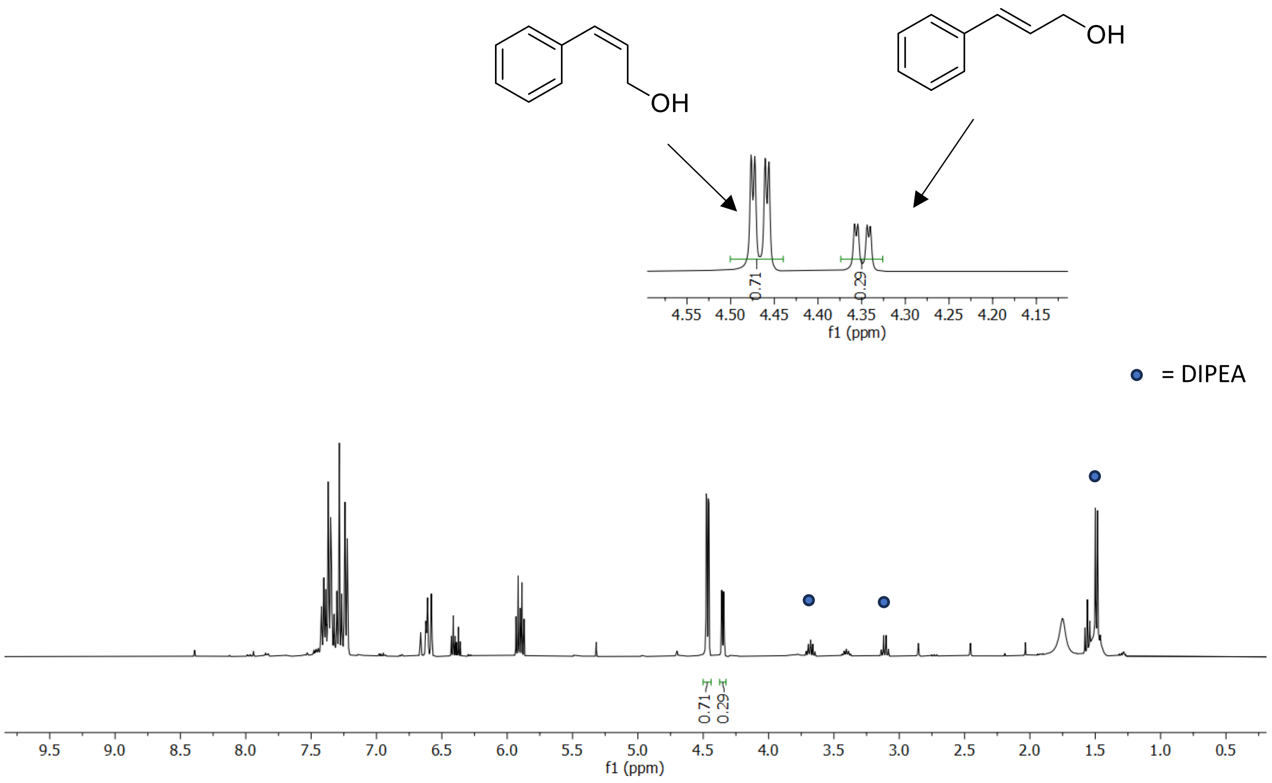

Photoisomerization with **PC6**, crude **A**+**B**, [1] H NMR (400 MHz, $CDCl_3$)

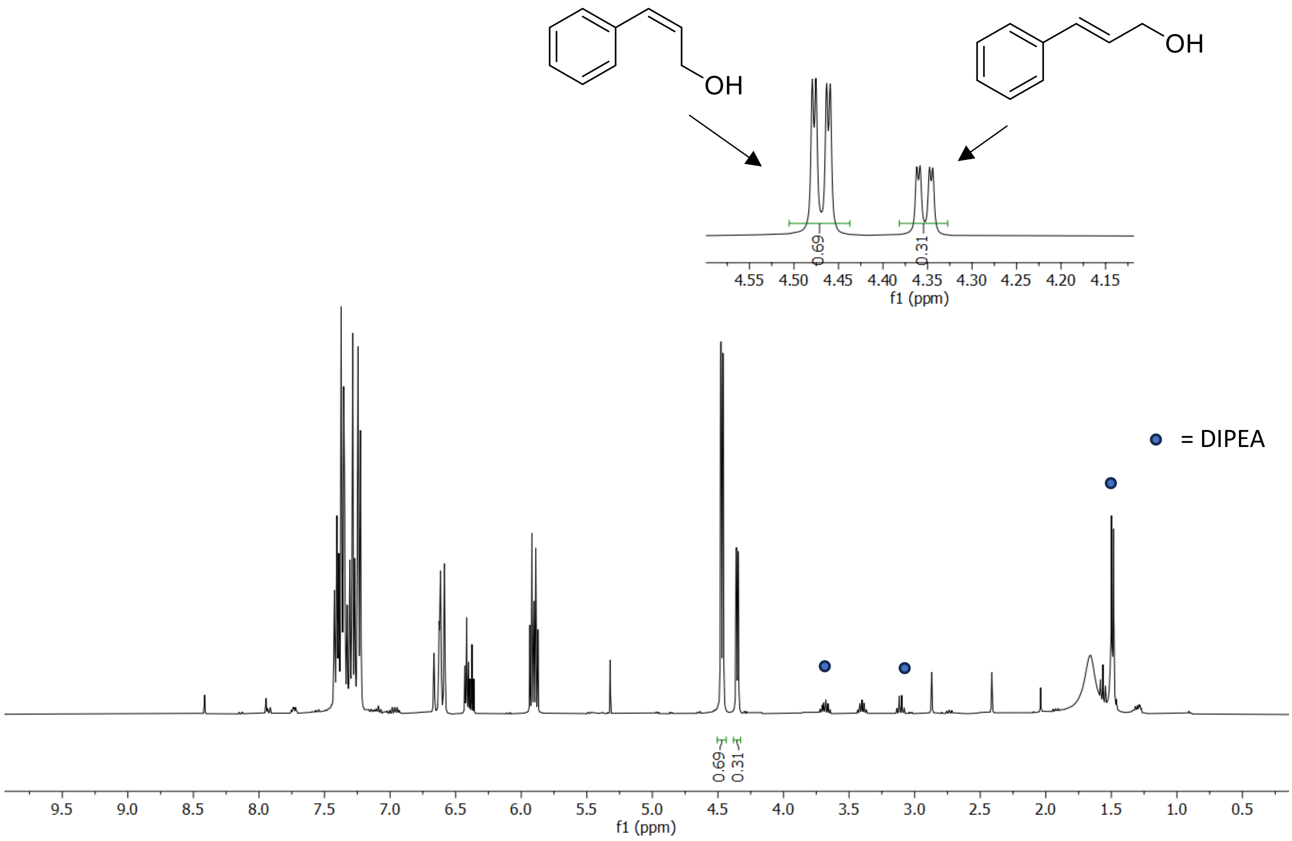

Photoisomerization with **PC7**, crude **A**+**B**, ^{1}H NMR (400 MHz, $CDCl_3$)

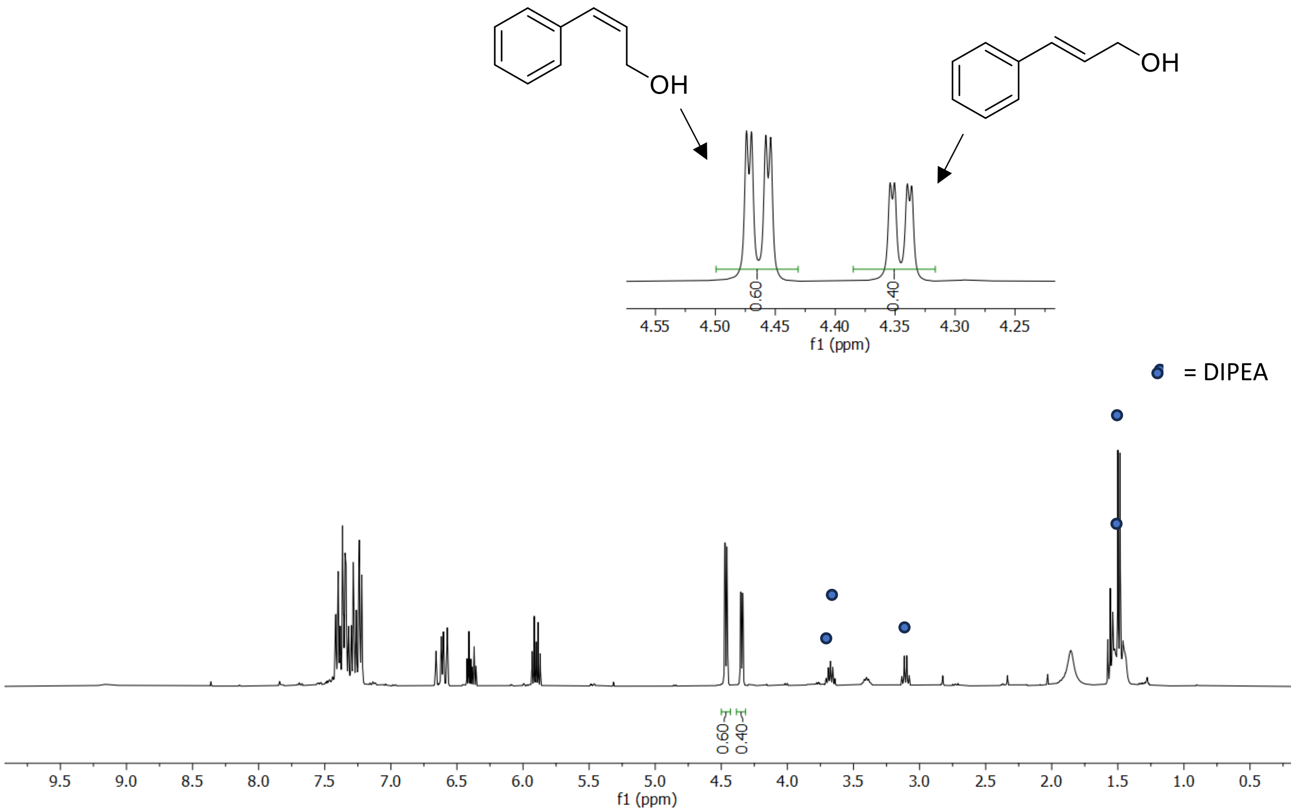

Photoisomerization with **PC8** crude **A**+**B**, ^{1}H NMR (400 MHz, $CDCl_3$)

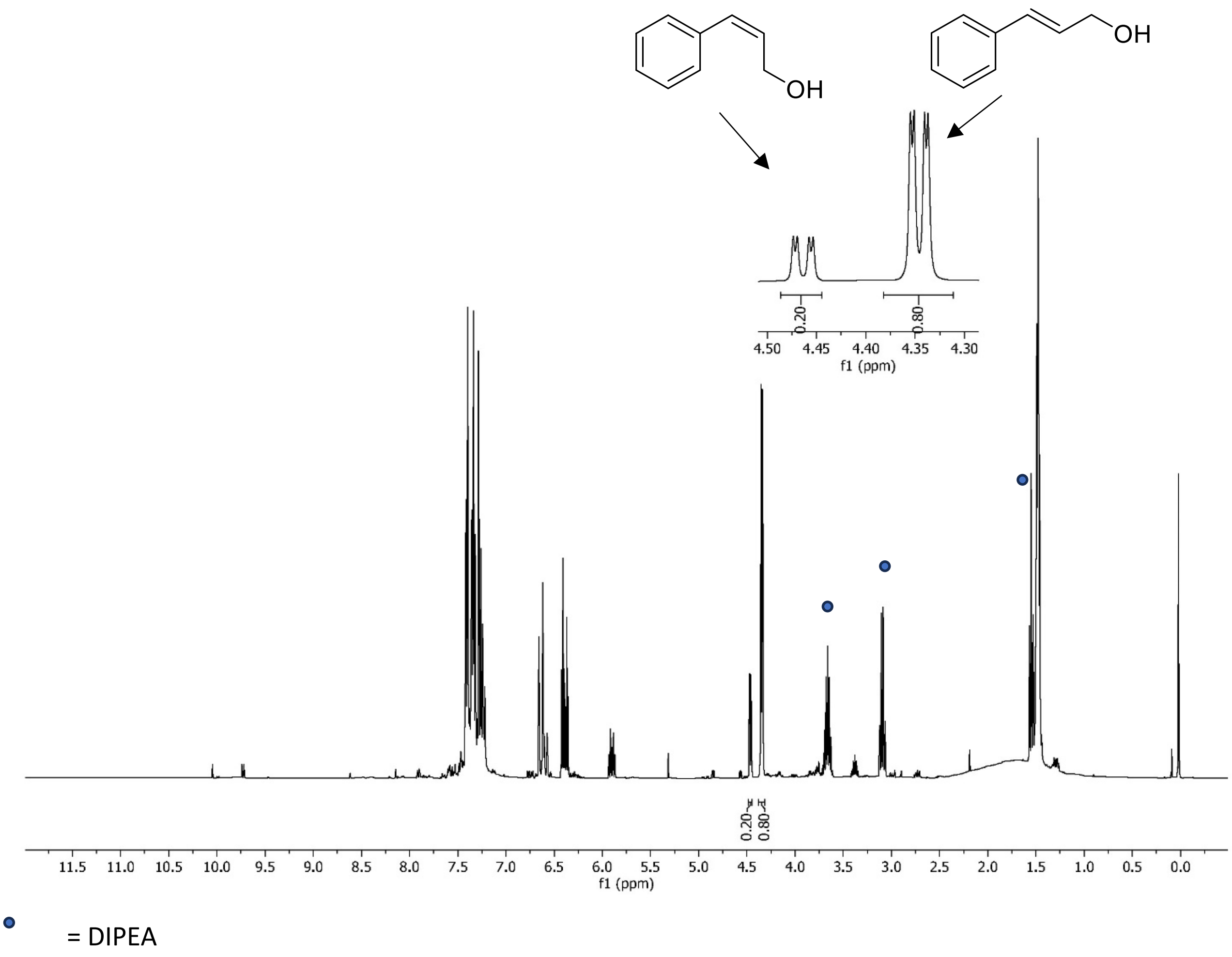

Photoisomerization with **PC9**, Blue LEDs, crude **A**+**B**, [1] H NMR (400 MHz, $CDCl_3$)

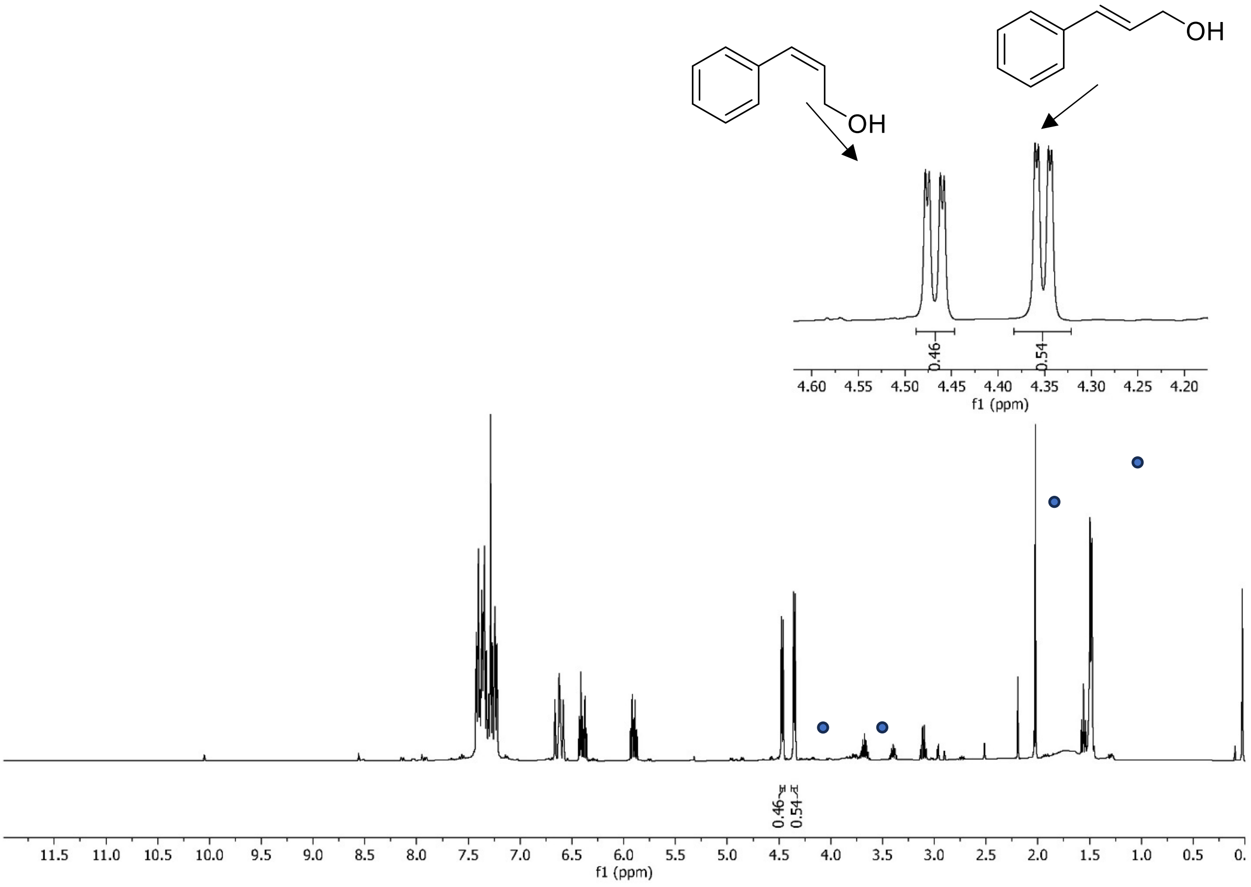

● = DIPEA

Photoisomerization with **PC9**, Violet LEDs crude **A**+**B**, [1] H NMR (400 MHz, $CDCl_3$)

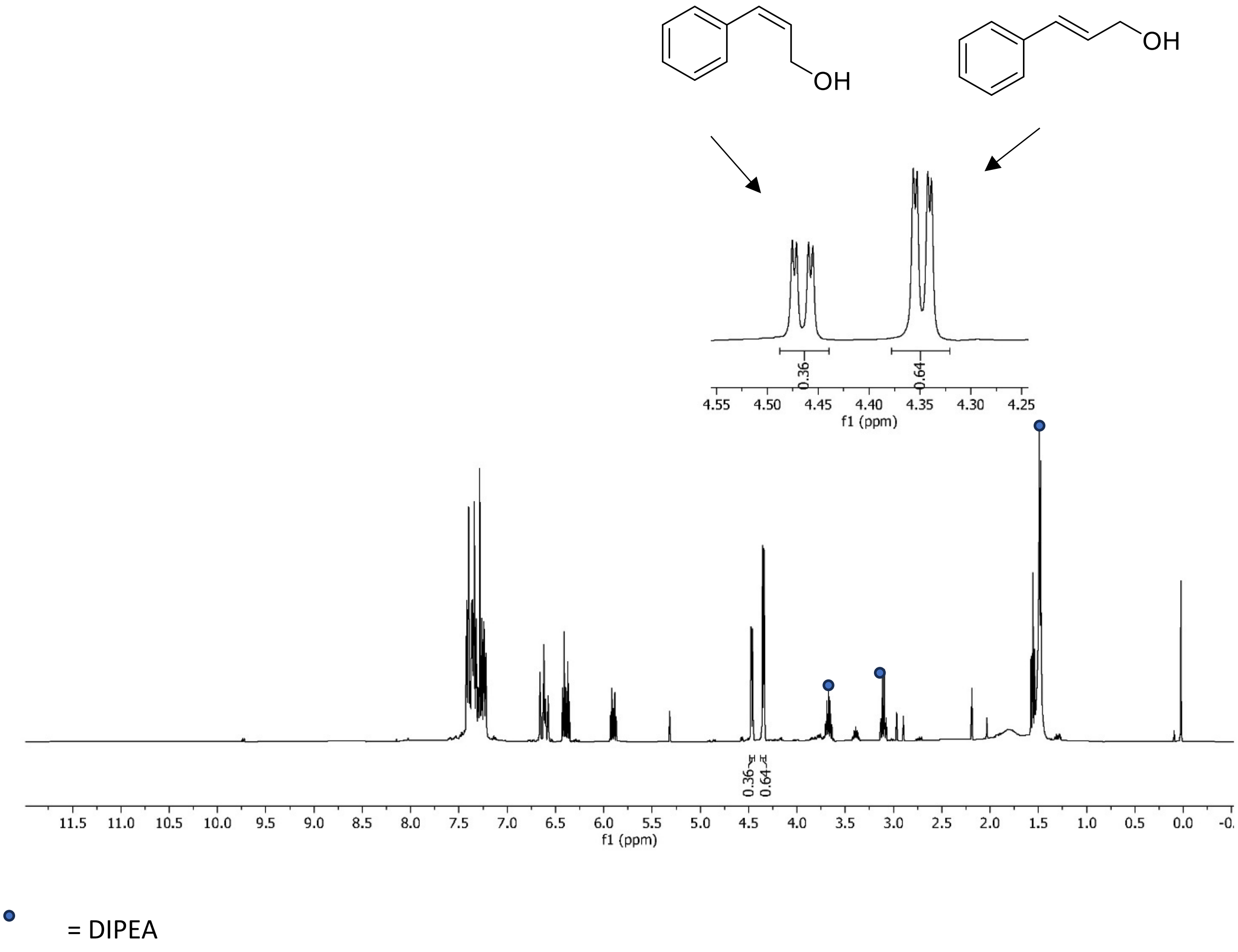

Photoisomerization with **PC10**, Blue LEDs crude **A**+**B**, ^{1}H NMR (400 MHz, $CDCl_3$)

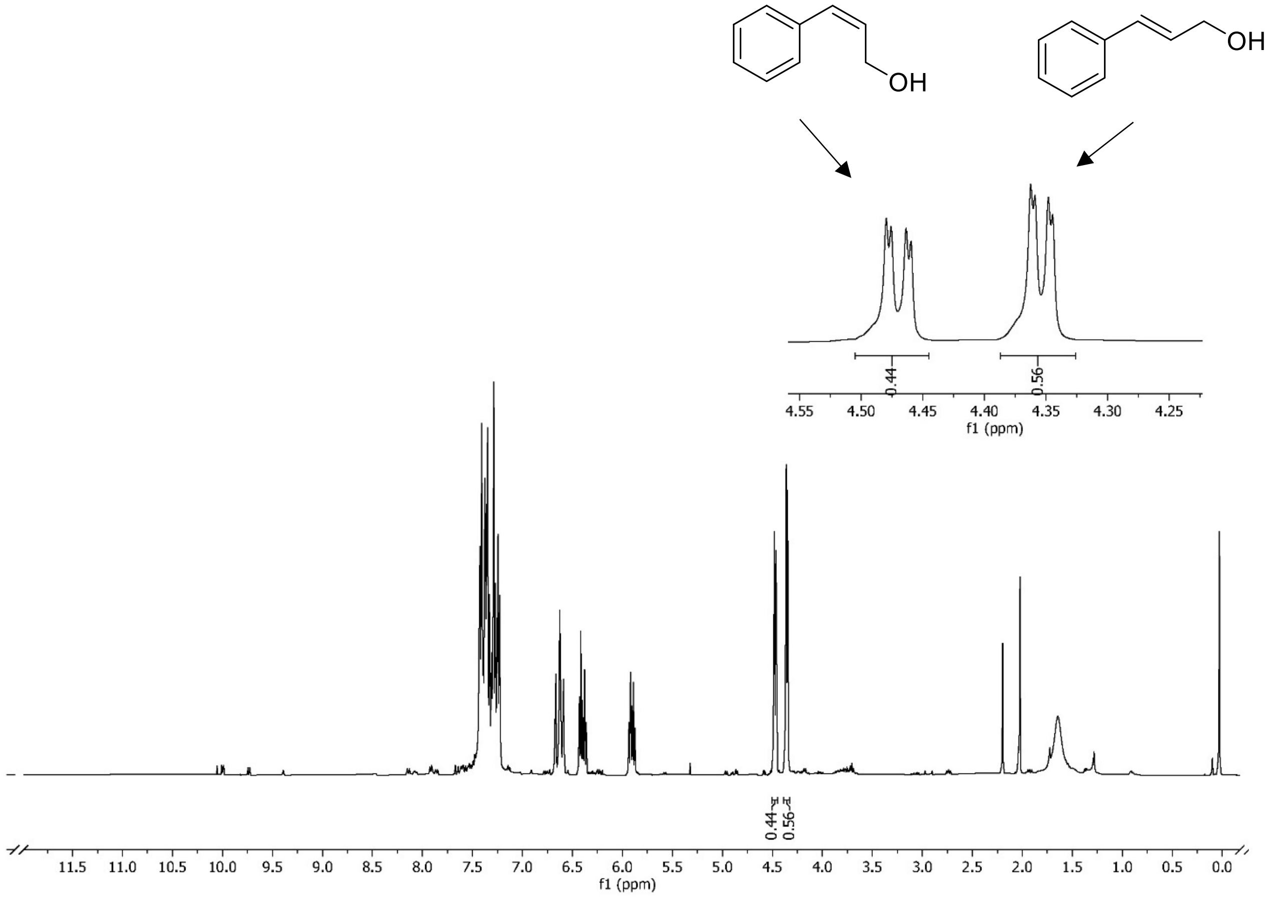

= DIPEA

Photoisomerization with **PC10**, Violet LEDs crude **A**+**B**, [1] H NMR (400 MHz, $CDCl_3$)

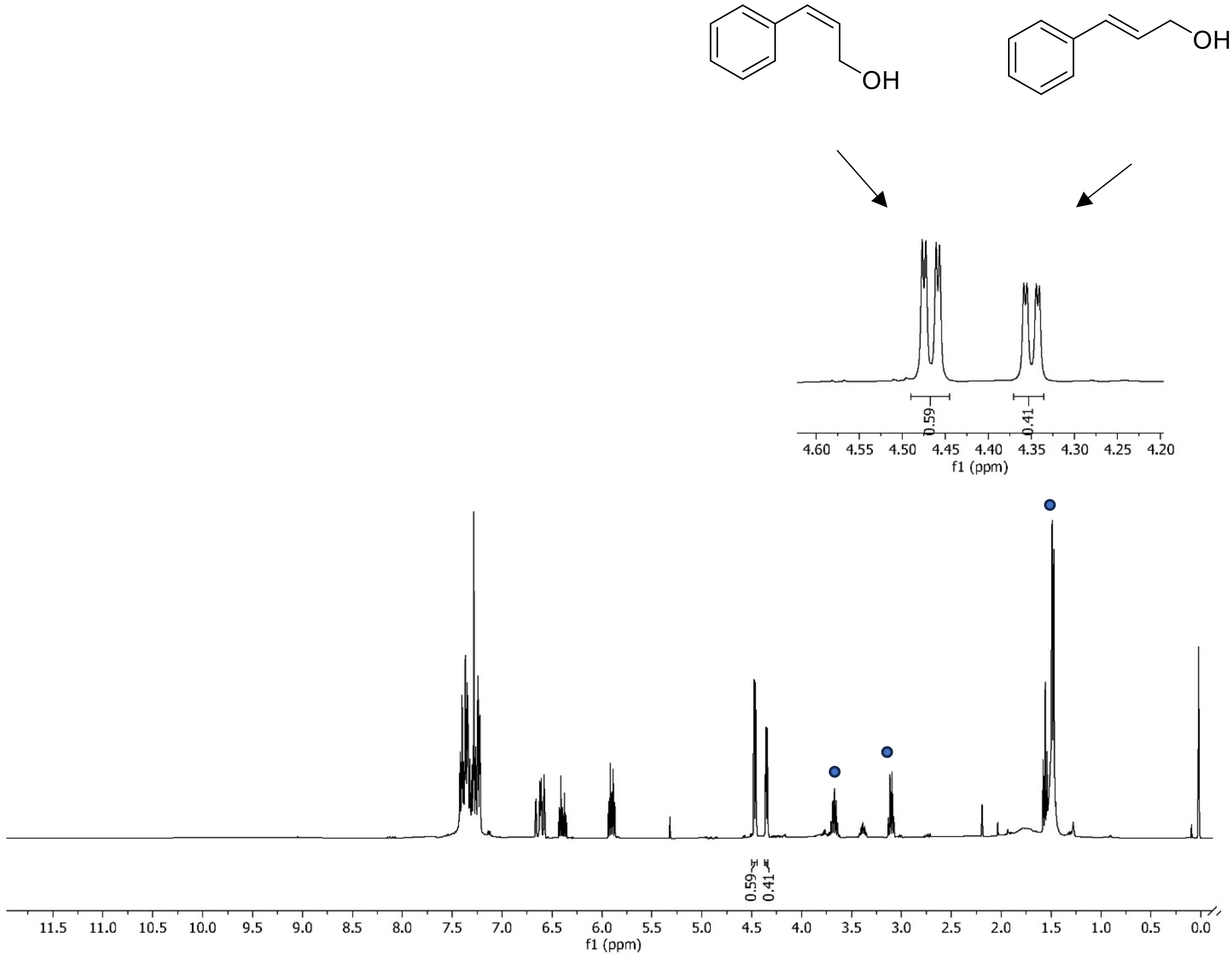

● = DIPEA

Photoisomerization with **PC5**, Violet LEDs crude **A**+**B**, **^{1}H NMR** (400 MHz, $CDCl_3$)

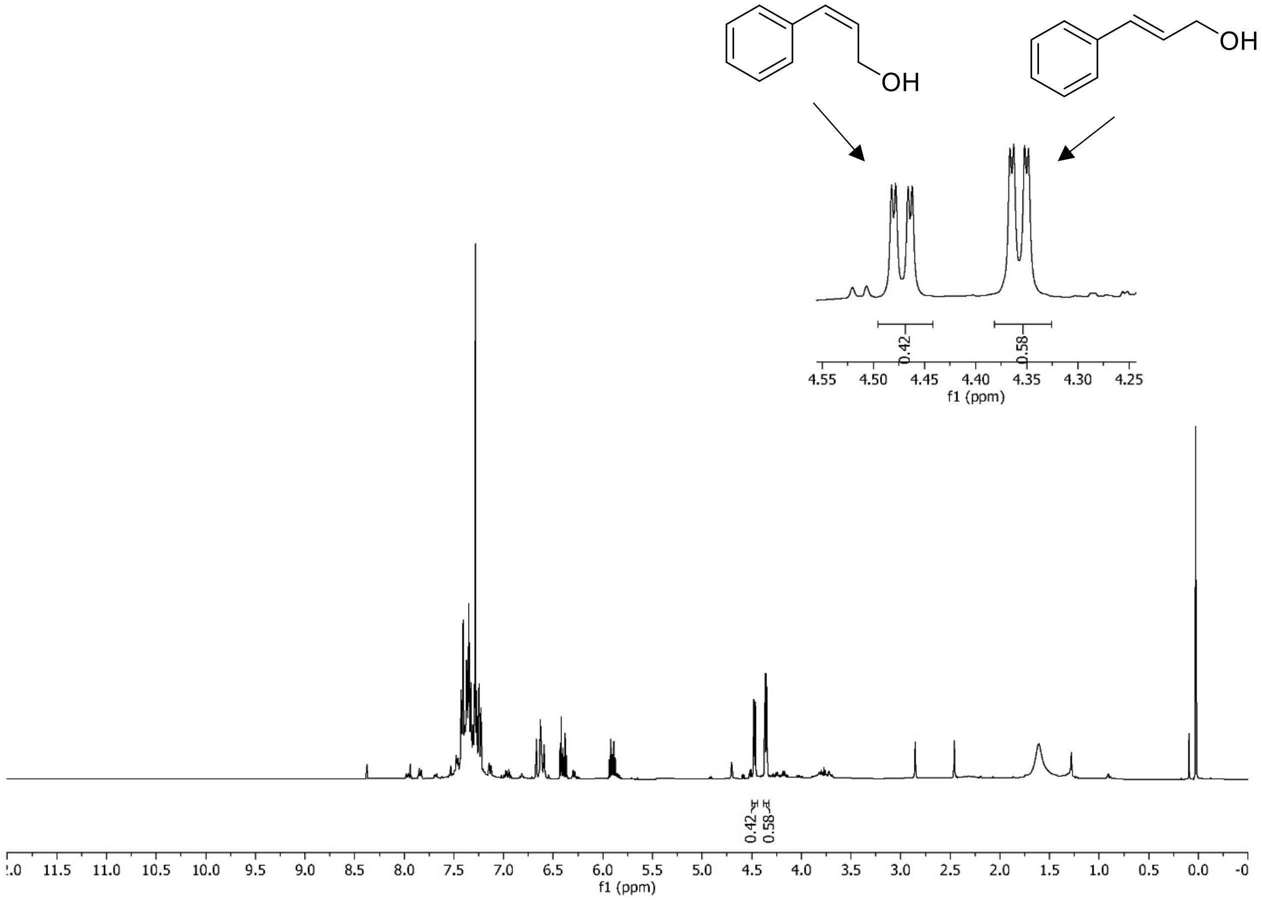

Photoisomerization with **PC11**, crude **A**+**B**, [1] H NMR (600 MHz, $CDCl_3$)

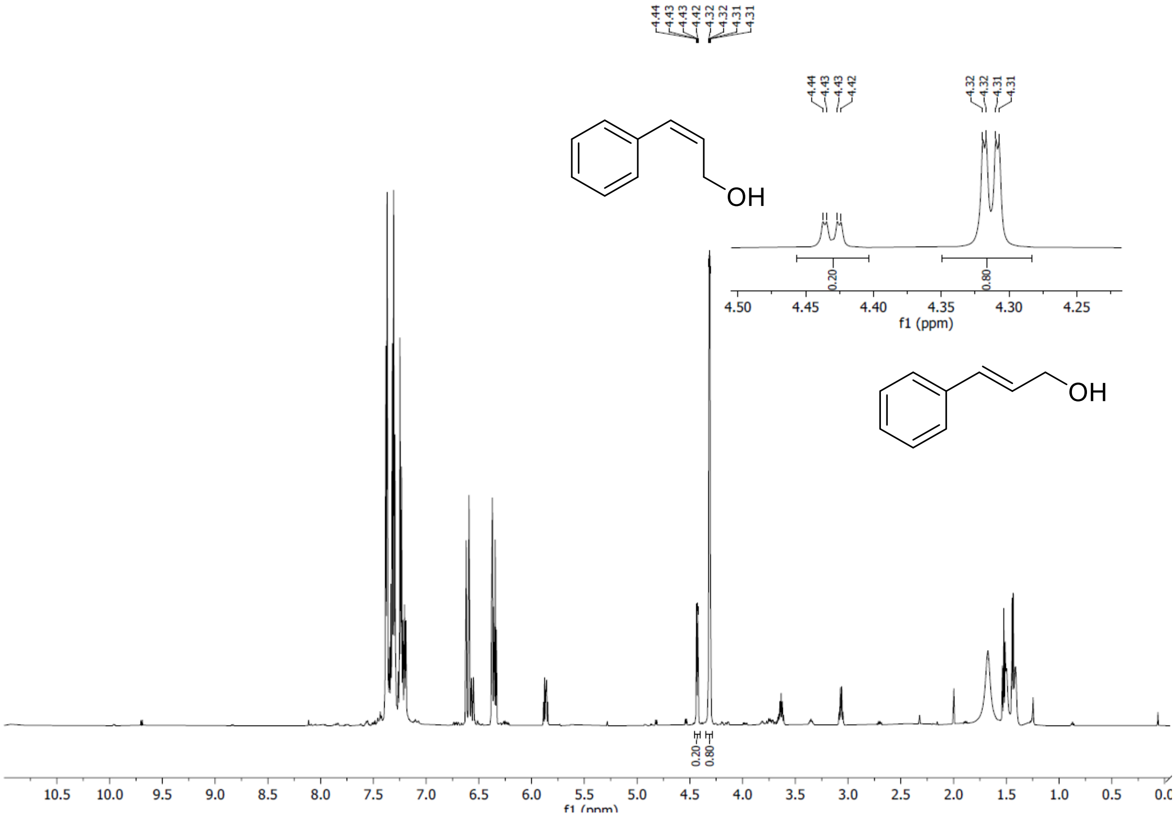

References

[41] A. Cerveri, G. Scarica, S. Sparascio, M. Hoch, M. Chiminelli, M. Tegoni, S. Protti, G. Maestri, *Chem. Eur. J.* **2024**, *30*, e202304010

[62] J. Lee, W. J. Song, *J. Am. Chem. Soc.* **2023**, *145*, 5211–5221.

[63] K. Kato, K. Furukawa, T. Mori, A. Osuka, *Chem. Weinh. Bergstr. Ger.* **2018**, *24*, 572–575.

[64] A. Roy, M. Oestreich, *Chem. – Eur. J.* **2021**, *27*, 8273–8276.

[65] Y. Fu, J. Huang, Y. Wu, X. Liu, F. Zhong, J. Wang, *J. Am. Chem. Soc.* **2021**, *143*, 617–622.

[66] C. E. McCusker, J. K. McCusker, *Inorg. Chem.* **2011**, *50*, 1656–1669.

[67] Z. Li, H. Li, B. J. Gifford, W. D. N. Peiris, S. Kilina, W. Sun, *RSC Adv.* **2016**, *6*, 41214–41228.

[68] M. Hitt, A. N. Vedernikov, *Org. Lett.* **2022**, *24*, 7737–7741.

[69] E. Sauvageot, P. Lafite, E. Duverger, R. Marion, M. Hamel, S. Gaillard, J.-L. Renaud, R. Daniellou, *J. Organomet. Chem.* **2016**, *808*, 122–127.

[70] H. Oh, D. G. Seo, H. C. Moon, *Org. Electron.* **2019**, *65*, 394–400.

[71] M. Graf, R. Czerwieniec, P. Mayer, H.-C. Böttcher, *Inorganica Chim. Acta* **2021**, *527*, 120554.

[72] K. Li, M.-L. Li, Q. Zhang, S.-F. Zhu, Q.-L. Zhou, *J. Am. Chem. Soc.* **2018**, *140*, 7458–7461.

[73] J. Hu, C. Zhang, *Anal. Chem.* **2013**, *85*, 2000–2004.

[74] W. E. Jr. Jones, M. A. Fox, *J. Phys. Chem.* **1994**, *98*, 5095–5099.

[75] H. Li, C. Wang, F. Glaser, N. Sinha, O. S. Wenger, *J. Am. Chem. Soc.* **2023**, *145*, 11402–11414.
